# A Stoichiometric Haloform Coupling for Ester Synthesis with Secondary Alcohols

**DOI:** 10.1002/anie.202400570

**Published:** 2024-04-18

**Authors:** Albert C. Rowett, Stephen G. Sweeting, David M. Heard, Alastair J. J. Lennox

**Affiliations:** ^1^ School of Chemistry University of Bristol Cantock's Close Bristol BS8 1TS UK

**Keywords:** Ketones, Haloform, Kinetics, Modelling, Esters

## Abstract

The haloform reaction from methyl ketones to carboxylic acids is one of the oldest known synthetic organic reactions, which has been used in myriad applications over the decades. The corresponding reaction to produce esters is, however, less developed, as the reaction is generally limited to simple, primary alcohols that are used in solvent‐level quantities, thereby restricting the complexity of esters that can be directly formed. Herein, we detail the development of a general ester‐forming haloform coupling reaction using one equivalent of alcohol. Mechanistic and kinetic modelling studies demonstrated that the key intermediates are formed under equilibrium, which facilitated the development of conditions that are amenable to secondary alcohols.

## Graphical abstract

A general haloform coupling reaction for the synthesis of esters from methyl ketones is reported using one equivalent of alcohol. Detailed kinetic modelling studies demonstrate that the key intermediates are formed under equilibrium, which led to the development of conditions that are amenable to secondary alcohols. TOC image: Georges Simon Serullas. Halftone after lithograph by Palley. Wellcome Collection, Public Domain Mark. Source: Wellcome Collection.

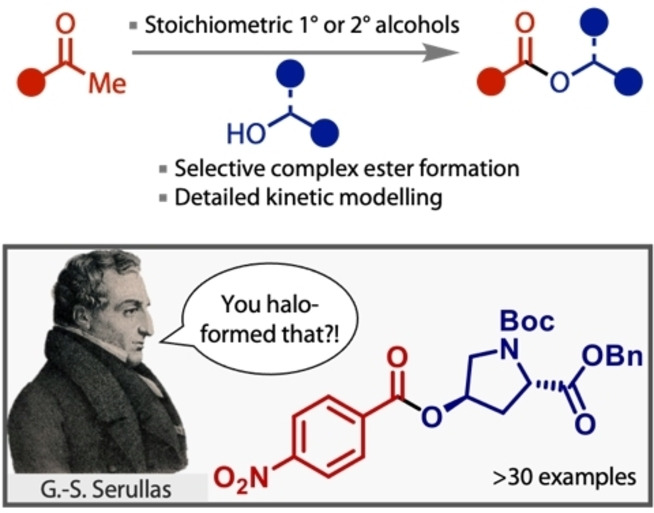

First reported in 1822 by G.‐S. Serullas, the haloform reaction is one of the oldest known synthetic organic reactions.[^1^, ^2^] The transformation of a methyl ketone to a carboxylic acid is conducted under such mild conditions that it has become routinely employed as a reliable method for preparing carboxylic acids in the synthesis of pharmaceutical agents,[^3^, ^4^, ^5^, ^6^] natural products,[^7^, ^8^, ^9^, ^10^, ^11^, ^12^, ^13^, ^14^, ^15^, ^16^] fragrances,[^17^, ^18^] and, more recently, for biomass valorisation.^[19]^ Exhaustive halogenation of the methyl ketone provides a sufficiently good leaving group that C−C bond cleavage is induced by subsequent attack from a hydroxide ion, Figure 1A. The by‐product of the reaction is a ‘haloform’, CHX_3_. The insolubility of iodoform (formed when iodine is used) led to an early application of the reaction as a diagnostic test for the presence of methyl ketones.[^20^, ^21^]

**Figure 1:**
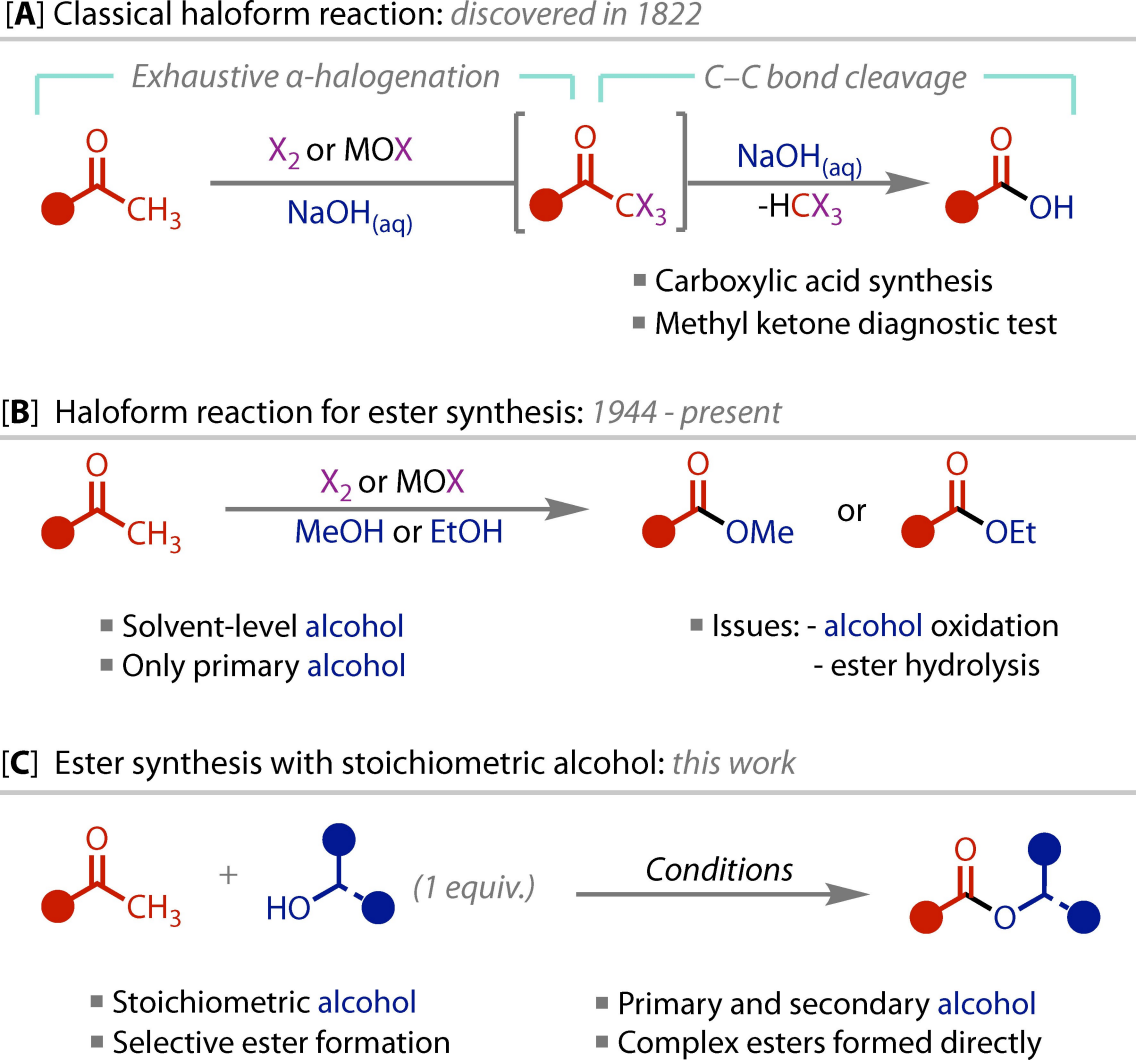
**A)** Classical haloform reaction for the synthesis of carboxylic acids, and **B)** esters. **C)** Structurally‐complex ester synthesis via haloform coupling with stoichiometric primary or secondary alcohols (this work).

In 1944, this strategy was discovered to be amenable for the preparation of methyl esters if the reaction was conducted in methanol.^[22]^ Since then, the reaction has been applied in natural product synthesis,[^23^, ^24^, ^25^] and has been demonstrated with other simple alcohols.[^26^, ^27^, ^28^, ^29^, ^30^, ^31^] However, the haloform reaction has not prospered in the synthesis of more complex esters. This constraint is rooted in the apparent requirement for solvent‐level alcohol to be used, which, for reasons of cost and practicality (physical state/solubility), clearly limits the scope of esters that can be prepared, Figure 1B. In particular, while the use of solvent‐level quantities of simple primary alcohols is well‐established (MeOH and EtOH),[^26^, ^27^, ^28^, ^29^, ^31^] the almost complete absence of examples using secondary alcohols is striking.^[30]^ By contrast, primary, secondary and tertiary amides have all been prepared from methyl ketones using only a small excess (2–10 equiv.) of ammonia or the pertinent amine.[^19^, ^31^, ^32^, ^33^, ^34^, ^35^, ^36^] The corresponding use of stoichiometric alcohol for ester synthesis remains elusive, however, with no reports targeting complex or secondary alcohols.^[37]^ Issues commonly encountered with alcohols, which are less nucleophilic than amines, include ester hydrolysis, and alcohol oxidation by the hypohalite formed in situ from dihalogen and hydroxide.[^2^, ^22^]

With these long‐standing limitations in mind, we sought to develop a general haloform reaction that is suitable for coupling methyl ketones with one equivalent of a wide range of alcohols, including both primary and the more elusive secondary alcohols. Herein, we describe the development of this system, which enables the direct construction of valuable, structurally‐complex esters that have been previously inaccessible via the haloform reaction, Figure 1C. We also report detailed kinetic modelling, which has provided key insight into the mechanism and demonstrate how it informed our optimisation efforts.

We began our investigation by briefly exploring how the classical conditions performed with acetophenone **1 a** and three simple alcohols, including secondary alcohol *i*‐PrOH. While a quantitative yield of the methyl ester was obtained, substituting methanol for ethanol resulted in a notable drop in yield, an effect that was even more pronounced with isopropyl alcohol, Figure 2A. These preliminary data highlight the difference in reactivity between different alcohols and the particular challenge posed by secondary alcohols.

**Figure 2:**
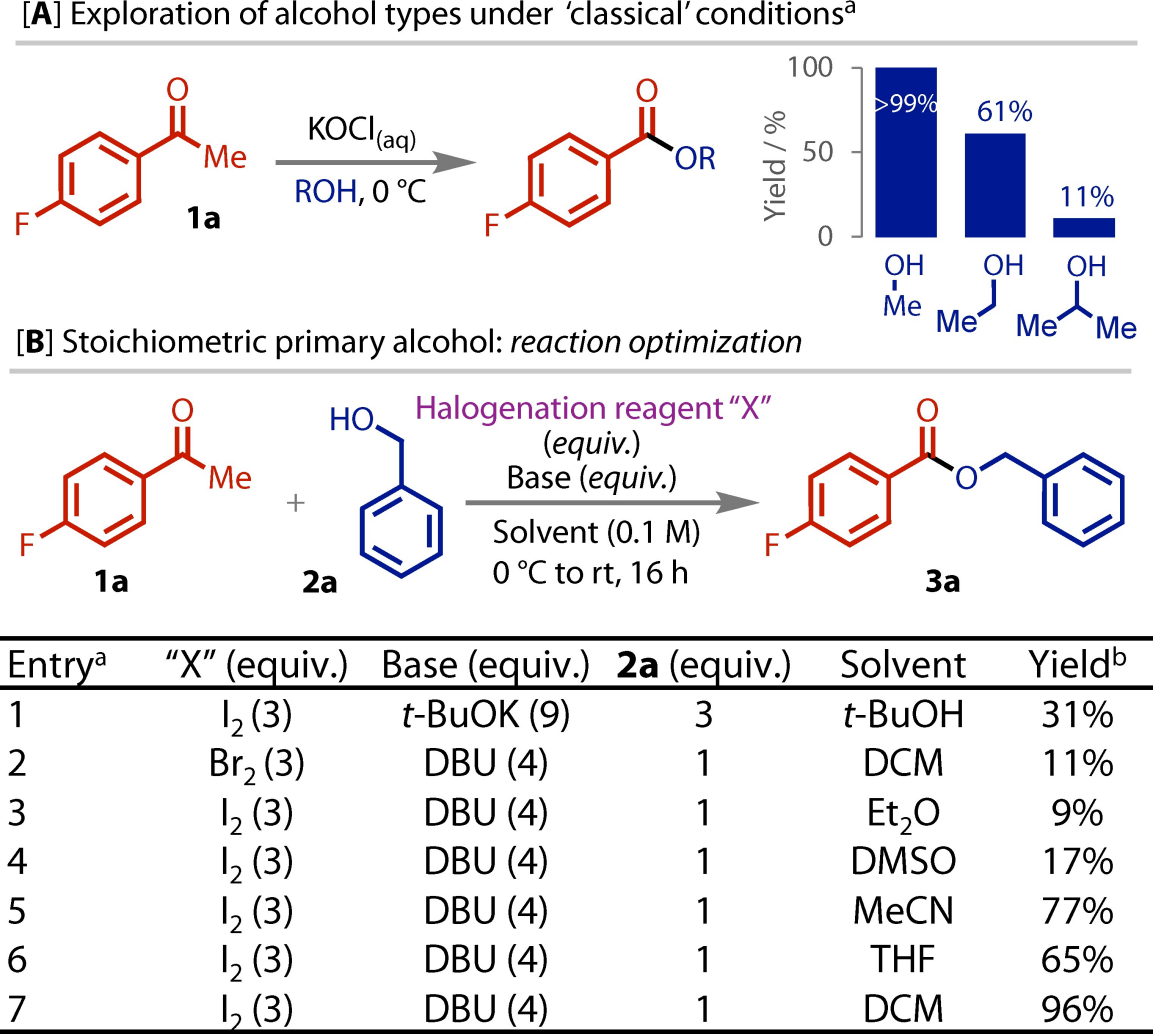
**A)** Classical conditions using solvent‐level alcohol. a: KOCl prepared immediately prior to use. **B)** Selected optimisation results with 1 equiv. of primary alcohol. a: 0.412 mmol scale; b: ^19^F NMR yield, calculated vs 4,4′‐difluorobiphenyl (internal standard).

With these insights in mind, we chose to first optimise the stoichiometric primary alcohol haloform reaction with benzyl alcohol. To avoid competing carboxylic acid formation, our optimisation focused on finding suitable anhydrous reagents. Initial attempts to build on our previous experience with electrochemical oxidation of anhydrous halide salts[^38^, ^39^, ^40^] were unsuccessful (see Supporting Information for details). Hence, our attention focused on organic‐soluble halogenation reagents. The use of iodine with *t*‐BuOK in *t*‐BuOH are conditions that have previously been employed for carboxylic acid synthesis from methyl ketones.^[19]^ However, despite using 3 equivalents of benzyl alcohol **2 a**, only a low yield of ester **3 a** was obtained in our case (entry 1, Figure 2B). A range of organic bases were therefore surveyed, along with a variety of solvents, equivalents and temperatures (see Supporting Information for full details). With 1 equivalent of alcohol, bromine was unsuitable, as extensive alcohol oxidation was observed (entry 2). A solvent screen with iodine and DBU found that DCM gave the highest yields (entries 3–7).

The scope of the haloform reaction with primary alcohols was then explored, Figure 3. Electron‐neutral (**3 a**, **3 b**, **3 e**), ‐poor (**3 c**), and ‐rich (**3 d**) primary benzyl alcohols were successfully coupled. Aliphatic primary alcohols (**3 f**–**h**) were also tolerated, as were *N*‐ and *O*‐heterocyclic alcohols (**3 i**–**j**). In addition to the electron‐poor nitroacetophenone used, a sterically‐hindered, electron‐rich acetophenone was tolerated (**3 k**). Finally, α,β‐unsaturated methyl ketones (**3 l**–**m**) were also suitable substrates.

**Figure 3:**
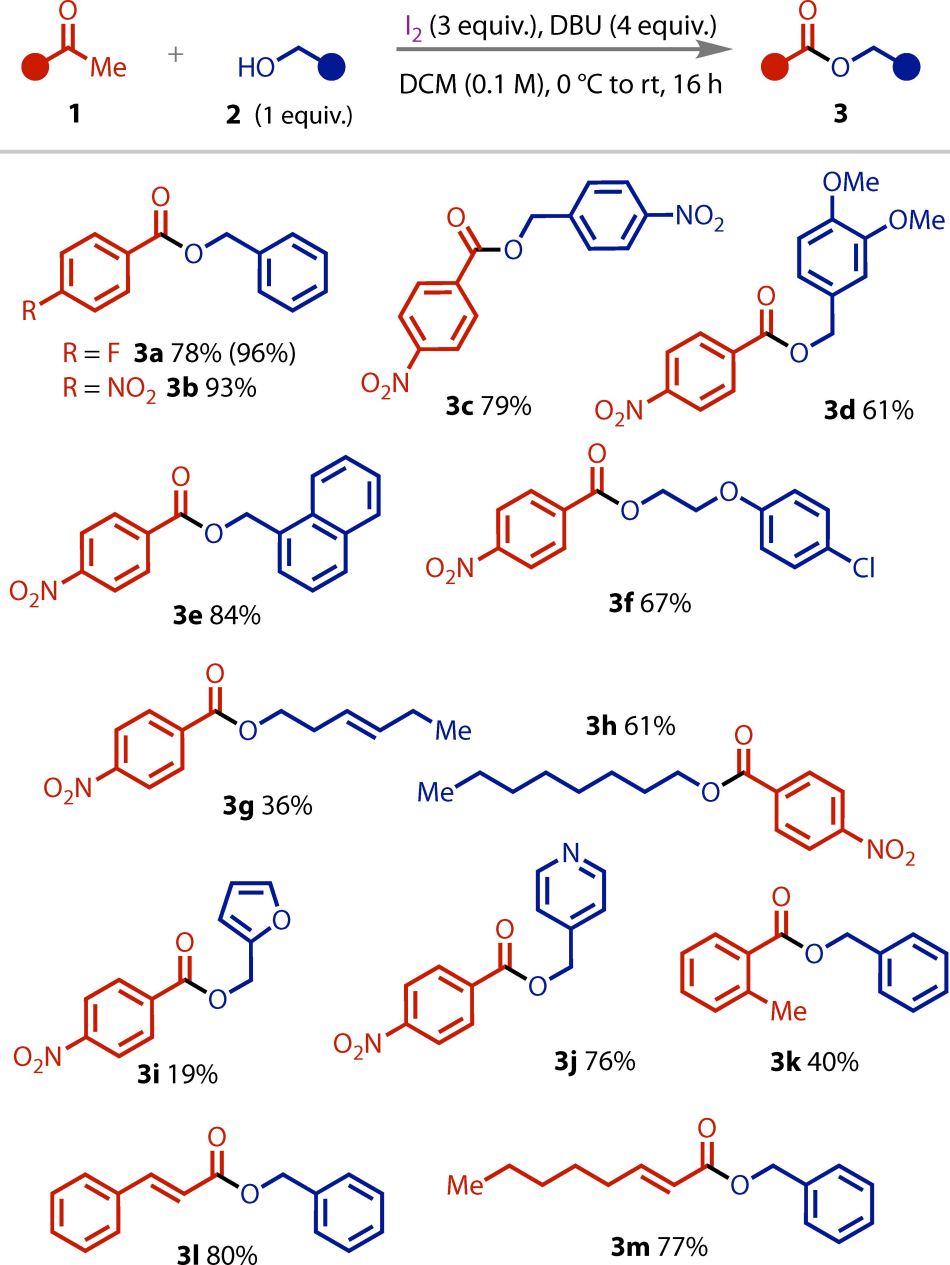
Scope of the stoichiometric haloform coupling with primary alcohols. Isolated yields, with ^19^F NMR yield shown in parentheses.

The use of secondary alcohols in haloform reactions is extremely limited^[30]^ and no examples of stoichiometric use have been reported. When we subjected secondary benzyl alcohol **4 a** to the optimised reaction conditions, a 32 % yield of ester **5 a** was observed, Figure 4A. Alternative reaction temperatures and concentrations, and controlling the addition rate of **1 a** and **4 a** had little impact on the yield (see Supporting Information for details). Therefore, to understand this deficiency and establish conditions suitable for the stoichiometric coupling of secondary alcohols, we conducted a series of mechanistic experiments.

**Figure 4:**
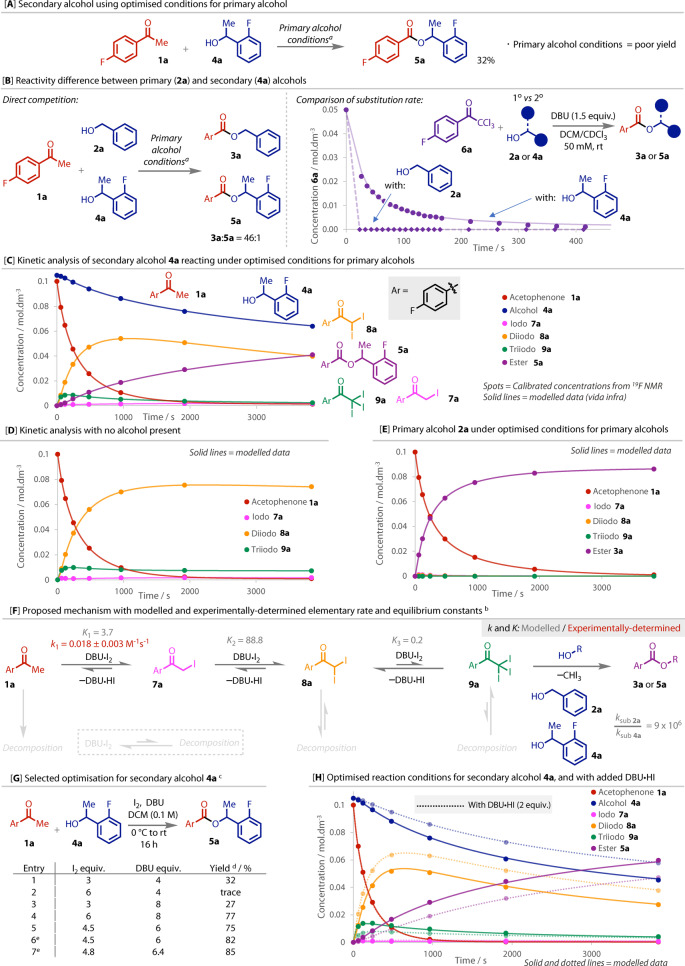
Mechanistic and kinetic modelling studies leading to reaction optimisation with secondary alcohol **4 a**. a: **1 a** (0.1 M) in DCM, I_2_ (3 equiv.), DBU (4 equiv.); b: See Supporting Information for the full list of kinetic constants and sensitivity analysis calculated throughthe parameter estimation workflow in COPASI. NB: the set of absolute values generated may be one of several possible solutions that fit the data. c: 0.412 mmol scale with anhydrous DCM in oven‐dried glassware under N_2_; d: ^19^F NMR yield, calculated vs 4,4′‐difluorobiphenyl (internal standard); e: 410 mg activated 3 Å molecular sieves added. For the plot in: [B], solid line is second order decay curve, dashed line is a guide for the eye only; [C], [D], [E] and [H], solid and dotted lines are all modelled data from the model shown in [F].

To quantitatively assess the difference in reactivity between a primary (**2 a**) and secondary (**4 a**) alcohol, we first conducted a competition experiment between **2 a** and **4 a** with limiting acetophenone **1 a**, Figure 4B. A large ratio (46 : 1) was observed between the two products (**3 a** : **5 a**), emphasising the difference in reactivity between the two alcohols and the sensitivity of the C−C bond cleavage step to steric hindrance.^[2]^ This difference in reactivity was supported by the comparison of time courses for the reaction of trichloroacetophenone **6 a** with primary alcohol **2 a** and with secondary alcohol **4 a**, Figure 4B. Using **6 a** instead of **1 a** allowed the relative rates of just the substitution step to be directly compared.^[41]^ With primary alcohol **2 a**, the reaction was complete within 20 seconds, before the first sample could be analysed by NMR. Secondary alcohol **4 a** was much slower and fitted a second order decay. However, although **4 a** was slower than **2 a**, both reactions reached full conversion and gave equally high yields of their respective products. This indicated that although steric hindrance affects the rate of substitution, it is not the substitution step that limits the yield.

To gain a deeper understanding of the mechanism and the cause of the reduced yield with secondary alcohol, we undertook a detailed kinetic modelling study.^[42]^ Data for the model was collected from reaction time courses, employing ^19^F NMR to analyse quenched aliquots.[^43^, ^44^]

We began our experimental studies by measuring the kinetics of a reaction with the poorly‐yielding secondary alcohol **4 a** under the optimised conditions for primary alcohols, Figure 4C. Acetophenone **1 a** reacted rapidly to complete consumption, consistent with our synthetic observations. Iodoacetophenone **7 a** was only observed at very low concentrations, indicating a rapid follow‐on reaction. Diiodoacetophenone **8 a** formed rapidly, resulting in a high concentration that slowly depleted. Triiodoacetophenone **9 a** also formed rapidly at the start of the reaction, but in much lower quantities, then slowly decayed, though not quite to completion. While ester **5 a** formed steadily throughout the reaction, its rate of formation decreased with conversion. This inverse correlation between conversion and reaction rate is also evident in the consumption rate of alcohol **4 a**. Hence, the reaction rate was attenuating despite significant quantities of intermediates and alcohol remaining.

When the alcohol was omitted from the reaction, Figure 4D, an equilibrium was reached with **1 a** reacting to give diiodo **8 a** as the major species and triiodo **9 a** as the minor species. In the reaction of primary alcohol **2 a**, Figure 4E, none of the intermediates were observed in significant concentrations, as ester **3 a** formed much more rapidly.

We then sought to develop a kinetic model which would fit the data collected and incorporate our experimental observations. Based on Figure 4D, we built the model to include the formation of **9 a** under equilibrium, which is a previously ‐unreported feature of the haloform reaction.^[45]^ A rate constant for the decay of acetophenone **1 a** was experimentally determined and then locked into the model, enabling others to vary from it. Meanwhile, ^1^H NMR analysis demonstrated that DBU and iodine form a strong adduct (see Supporting Information for full details), therefore these were represented as a single entity in the model. Finally, since mass balances with secondary alcohol **4 a** were consistently below 100 %, we proposed that decomposition pathways of the reactive iodinated intermediates may be significant. Indeed, haloacetophenones similar to **7 a**, **8 a** and **9 a** are known to be unstable, for example by light‐induced C−X bond homolysis.[^46^, ^47^, ^48^, ^49^] With these conditions in place, we considered possible kinetic scenarios (see Supporting Information for full details). Specifically, several different combinations of decomposition pathways were considered, which were assessed for their individual contribution to the objective value, which is the weighted sum of square residuals and assesses the overall accuracy of the model. Only those that made a significant improvement to the model were included, Figure 4F.

We first optimised the rate constants of the elementary steps using data from the reaction with no alcohol, Figure 4D, maintaining the experimentally‐determined acetophenone decay rate constant (*k*_1_) throughout. To incorporate the substitution steps, we then inputted these rate constants into the other two experiments, Figure 4C,E, and separately optimised the substitution rate constants of each alcohol without limits, while also allowing flexibility in the other rate constants. Optimisations were iteratively repeated until the objective values for each experiment remained constant and the coefficient of variation around the mean of the rate constants for each elementary step was minimised. A single set of rate constants was produced by taking the mean across the 3 experiments (Figure 4C–E). This process produced a model, co‐plotted on each of the plots, which accurately fits all the experimental datasets using a single set of rate constants.

Considering these insights, we returned to optimising the reaction conditions for secondary alcohols. DBU ⋅ HI is formed as a by‐product in the iodination of acetophenone with DBU ⋅ I_2_. Our mechanistic proposal and model, Figure 4F, suggests that an equilibrium is present; as DBU ⋅ HI forms, the deiodination of triiodo **9 a** back to diiodo **8 a** starts to compete with substitution of **9 a** with secondary alcohol **4 a** to **5 a**. This issue is only encountered with secondary alcohols because the substitution rate is so much slower than that with primary alcohols. We therefore reasoned that increasing the concentrations of iodine and/or DBU should bias the equilibria towards the triiodo species **9 a**, thereby helping the substitution to outcompete the reverse de‐iodination reaction.^[50]^

When the iodine concentration was doubled, ester **5 a** formed in trace quantities (entry 2, Figure 4G), with significant alcohol oxidation to the corresponding ketone instead, a reaction known to take place under similar conditions.^[51]^ Doubling the concentration of DBU did not improve the yield either, as more carboxylic acid was formed (entry 3). When we increased the concentrations of both, a significant increase in yield was observed (entries 4 and 5). Carboxylic acid remained as the only major side‐product but could be eliminated by the addition of molecular sieves (entries 6 and 7), leading to our optimised conditions. A time course under these conditions showed an enhanced yield within the 1‐hour measured timeframe, Figure 4H, compared to the primary alcohol conditions, Figure 4C. The model also accurately predicted that if DBU ⋅ HI was added to the reaction mixture, an attenuation of the reaction would be observed, due to the perturbed equilibrium, Figure 4H dotted lines.

With efficient conditions for the stoichiometric secondary alcohol coupling in hand, we explored their impact on various functional groups through a robustness screen (see Supporting Information for details).[^52^, ^53^] The majority of functionalities tested during the coupling of **1 a** with **4 a** (including aliphatic alkenes, nitriles and chlorides, electron‐poor aromatics, aldehydes and internal alkynes) had little to no negative impact on reaction yield and were themselves stable to the conditions. Those less tolerated were either susceptible to iodination (terminal alkynes, ketones, and electron‐rich aromatics), oxidation (amines) or were competitive nucleophiles (aliphatic amines and alcohols).

The scope was then explored using a single equivalent of each coupling partner. Electron‐rich and ‐poor acetophenones were successfully coupled (Figure 5, **5 a**–**c**), as were *N*‐heterocyclic methyl ketones (**5 d**–**e**). Although *ortho*‐methyl substitution was tolerated with primary alcohols, only *meta*‐methyl substitution was viable with secondary alcohols (**5 f**), reflecting the more challenging steric environment (see Supporting Information for other unsuccessful substrates). Electron‐poor (**5 g**) and ‐rich (**5 h**–**i**) benzyl alcohols were compatible, as were sterically–hindered benzyl alcohols with (bis)*ortho* substitution (**5 j**) and diphenylmethanol (**5 k**) both tolerated. Extended conjugation (**5 l**) and a bicyclic benzyl alcohol (**5 m**) were coupled in good yields. Aliphatic secondary alcohols were also tested and found to proceed successfully (**5 n**–**s**). These included several natural products, which were coupled without the need to increase their stoichiometries.

**Figure 5:**
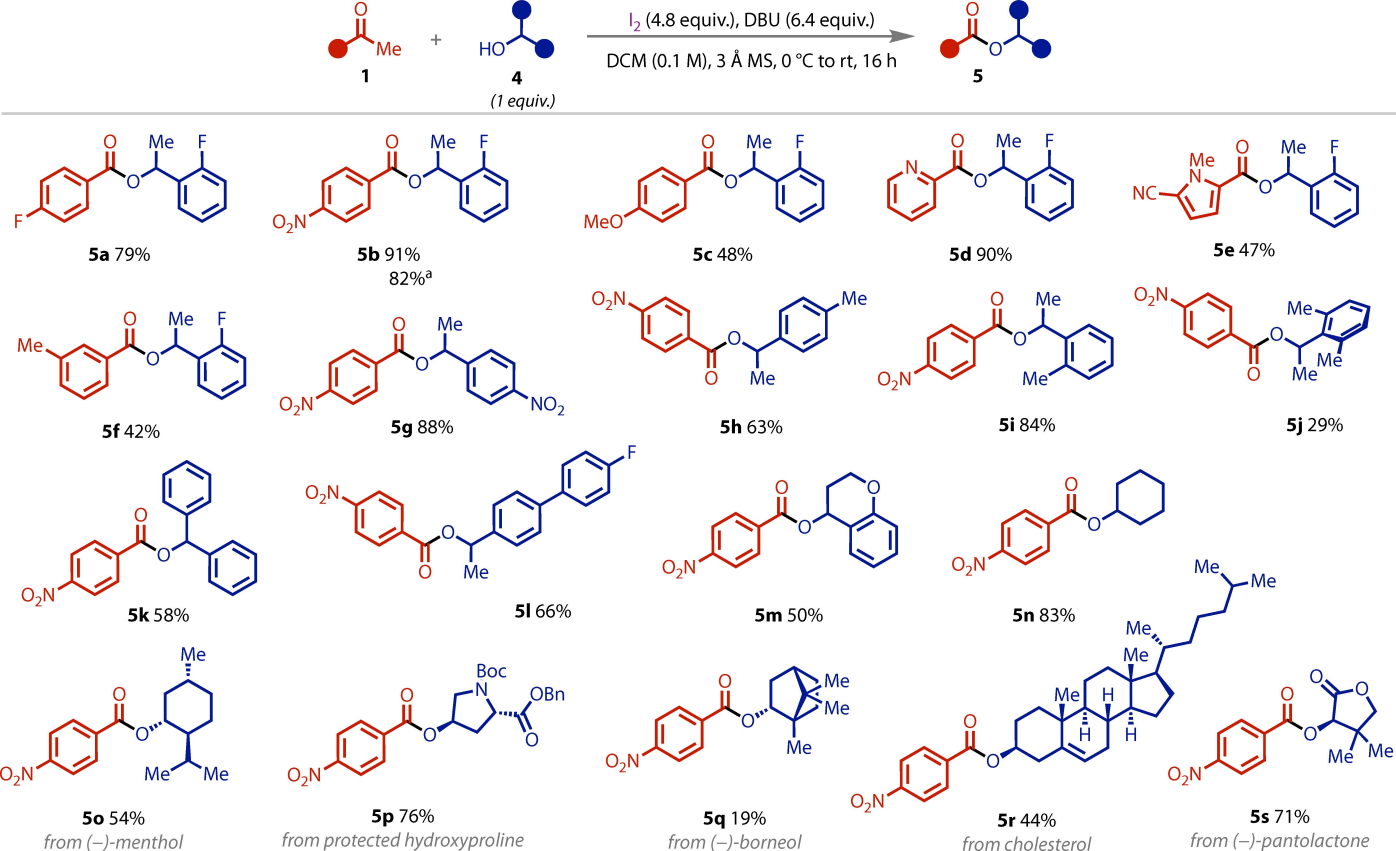
Scope of stoichiometric haloform coupling using 1 equivalent of secondary alcohol. a: 1 mmol scale. Isolated yields.

In summary, we have developed a general stoichiometric haloform coupling for ester synthesis with both primary and secondary alcohols. Mechanistic and kinetic modelling studies revealed that the secondary alcohol substitution step was substantially slower than that with primary alcohol. A previously–unrecognised equilibrium in the iodination of acetophenone was discovered. DBU ⋅ HI, formed as a by‐product of the iodinations, serves to inhibit ester formation when secondary alcohols are used. This is due to a competition for the triiodo intermediate (**9**) between DBU ⋅ HI (resulting in de‐iodination) and the slower reacting secondary alcohol. Conditions to overcome this were developed, enabling efficient couplings of secondary alcohols in stoichiometric quantities.

## Conflict of interests

The authors declare no conflict of interest.

## Supporting information

As a service to our authors and readers, this journal provides supporting information supplied by the authors. Such materials are peer reviewed and may be re‐organized for online delivery, but are not copy‐edited or typeset. Technical support issues arising from supporting information (other than missing files) should be addressed to the authors.

Supporting Information

## Data Availability

The data that support the findings of this study are available in the supplementary material of this article.
