# Supplementary material for: A Stoichiometric Haloform Coupling for Ester Synthesis with Secondary Alcohols
Source: Angew Chem Int Ed Engl. 2024 Apr 18;63(21):e202400570. doi: 10.1002/anie.202400570 (PMC11497235; doi:10.1002/anie.202400570)
Supplement: Supplementary file 1 — Supporting Information [file ANIE-63-e202400570-s001.pdf]

Angewandte Chemie
Eine Zeitschrift der Gesellschaft Deutscher Chemiker

Supporting Information

A Stoichiometric Haloform Coupling for Ester Synthesis with Secondary Alcohols

*A. C. Rowett, S. G. Sweeting, D. M. Heard, A. J. J. Lennox**

A Stoichiometric Haloform Coupling for Ester Synthesis with Secondary Alcohols

*Albert C. Rowett, Stephen G. Sweeting, David M. Heard and Alastair J. J. Lennox**

Table of Contents

General Experimental Details S3

Chemicals S3

Techniques S3

Chromatography S3

Analysis S3

Preliminary Experiments under Classical Conditions S4

Primary Alcohols S5

Electrochemical Attempts S5

Optimisation S6

Scope S7

Mechanistic Experiments and Kinetic Modelling S13

Competition Experiment S13

Trichloroacetophenone Substitution Time Courses S13

Haloform Reaction Time Courses S14

Evidence of DBU•I_2 Adduct Formation S21

Computational Study of Nucleophilic Attack S24

COPASI Modelling S33

Secondary Alcohols S62

Optimisation S62

Robustness Screen S67

Substrate Synthesis S70

Scope S72

Unsuccessful Couplings S82

NMR Spectra of Novel Compounds S84

References S129

General Experimental Details

Chemicals

Unless their synthesis is described, chemicals were obtained from commercial sources and were used without further purification, except for tetrabutylammonium hexafluorophosphate ($TBAPF_6$) and 1,8-diazabicyclo(5.4.0)undec-7-ene (DBU): $TBAPF_6$ was recrystallised from EtOH; DBU was dried over CaH_2, then distilled and stored in a Straus flask under nitrogen. Anhydrous solvents were only used where explicitly stated. Anhydrous DCM and THF were obtained from the Anhydrous Engineering double alumina drying system located at the University of Bristol and were stored in Straus flasks over activated 3 Å molecular sieves under nitrogen. Anhydrous EtOH was obtained from a commercial source and used without further drying. Anhydrous $CDCl_3$ was obtained by storage of commercially-obtained (non-anhydrous) $CDCl_3$ in a Straus flask over activated 3 Å molecular sieves under nitrogen.

Techniques

Unless stated otherwise, reactions were carried out at room temperature and open to air. Where procedures are described as having been carried out 'under nitrogen', standard Schlenk line (using vacuum lines attached to a double manifold, equipped with an oil pump) and glovebox techniques were employed, under an atmosphere of dry nitrogen. Oven-dried glassware was dried overnight in an oven at 180 °C and allowed to cool under vacuum (on a vacuum line, at room temperature and pressures up to ~0.1 mmHg). Molecular sieves were activated by drying overnight in an oven at 180 °C and then with a flame under vacuum (on a vacuum line, at pressures up to ~0.1 mmHg). Solvents were removed under vacuum using a rotary evaporator with water bath temperatures up to 40 °C and pressures up to ~10 mmHg (diaphragm pump), or on a vacuum line at room temperature and pressures up to ~0.1 mmHg (oil pump).

Chromatography

Thin layer chromatography (TLC) was performed using aluminium-backed silica gel 60 F_{254} plates and reagent grade solvents. Visualisation was achieved by UV fluorescence (254 nm), and/or basic potassium permanganate or phosphomolybdic acid stains. Flash column chromatography (FCC) was performed manually, using silica gel (40-63 µm, 230-400 mesh), or using a Biotage Selekt system with Biotage Sfär Silica (60 µm) or Biotage Sfär Silica HC (20 µm) pre-packed columns. Reagent grade solvents were used in both cases.

Analysis

^{1}H, ^{13}C and ^{19}F NMR spectra were recorded using Bruker Nano400, Jeol ECZ400, Jeol ECS400, Bruker 500 Cryo and Varian 500 spectrometers. Chemical shifts (δ) are reported in parts per million (ppm) and are referenced to residual solvent or tetramethylsilane (TMS) signals (^{1}H and ^{13}C only). Signals are described as singlets (s), doublets (d), triplets (t), quartets (q), quintets (quint), septets (sept) and multiplets (m), or combinations thereof, and are marked as 'apparent' (app) where relevant. Coupling constants (*J*) are quoted to the nearest 0.5 Hz. Spectra of novel compounds were assigned using 2D experiments (COSY, HSQC, HMBC) where required. Mass spectra were recorded by the University of Bristol Mass Spectrometry Service, using Thermo Scientific Q Exactive, Bruker ultrafleXtreme and Waters SYNAPT G2-S spectrometers. Infrared spectra were recorded using a PerkinElmer Spectrum Two spectrometer with an ATR accessory. Specific rotations ($[\alpha]_D^T$) were recorded with a Bellingham & Stanley ADP 220 polarimeter and are quoted in (° mL g^{-1} dm^{-1}). GC-MS analyses were performed with an Agilent 5977E GC/MSD system.

Preliminary Experiments under Classical Conditions

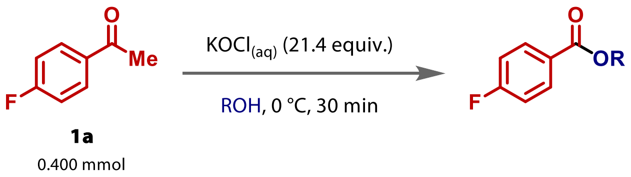

Based on literature procedures.[1,2] To a round-bottom flask was added 4'-fluoroacetophenone **1a** (50.0 µL, 0.412 mmol, 1 equiv.), 4,4'-difluorobiphenyl internal standard (23.5 mg, 0.124 mmol, 0.3 equiv.) and alcohol (MeOH/EtOH/*i*-PrOH; 2.5 mL). A small aliquot (~25 µL) of the resulting solution was taken for ^{19}F NMR analysis. To a separate round-bottom flask was added $Ca(OCl)_2$ (1.26 g, 8.83 mmol, 21.4 equiv.) and deionised water (5 mL), followed by addition of a solution of K_2CO_3 (884 mg, 6.39 mmol, 15.5 equiv.) and KOH (168 mg, 3.00 mmol, 7.28 equiv.) in deionised water (2.5 mL), which was prepared in another round-bottom flask. The mixture was stirred thoroughly, then filtered (Büchner filtration, washed with 1 mL deionised water) and the filtrate was added dropwise to the solution of **1a** and internal standard, which had been cooled to 0 °C (ice-water bath). On completion (~30 min, judged by TLC, by the consumption of **1a**), $NaHSO_3$ (360 mg, 3.46 mmol, 8.40 equiv.) was added to quench the reaction and an NMR sample was prepared from the reaction mixture (50 µL) and $CDCl_3$ (450 µL). ^{19}F NMR yields were calculated by comparison to the **1a**:internal standard ratio prior to the reaction. Ester formation was confirmed by comparison to literature ^{1}H and ^{19}F NMR data[3–5] after work-up: the quenched reaction mixture was filtered, diluted with deionised water, then extracted with Et_2O (×3), dried over anhydrous $MgSO_4$, filtered, and concentrated under vacuum.

Primary Alcohols

Electrochemical Attempts

Selected Representative Results

Table S1. Attempts at electrochemical haloform coupling via oxidation of halide salts.[a]

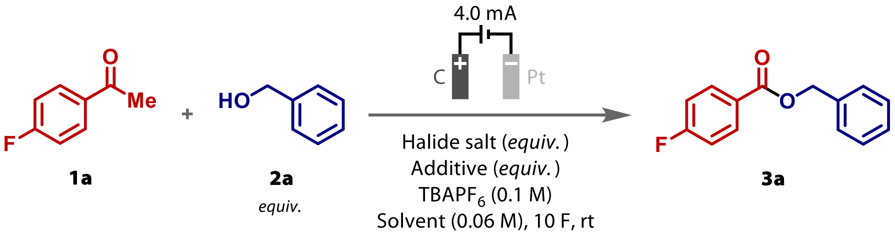

Entry	Halide salt (equiv.)	Additive (equiv.)	BnOH **2a** equiv.	Solvent	Yield[b]
1[c]	NaBr (3)	–	1	DMF	trace
2[c]	NaBr (3)	–	5	DMF	2%
3	TBACl (3)	–	2	DCM	0%
4	TBACl (3)	–	10	DCM	0%
5[d]	TBAI (4)	DBU (5)	2	DCM	12%

a: On 0.247 mmol scale; in undivided cell with graphite rod anode, platinum coil cathode. b: Calculated by ^{19}F NMR vs 4,4'-difluorobiphenyl internal standard. c: Reaction performed on 0.200 mmol scale, with graphite rod cathode (as well as anode). d: Reaction performed at 8.0 mA and 8 *F* passed.

Example Reaction Procedure (with TBACl, entry 3)

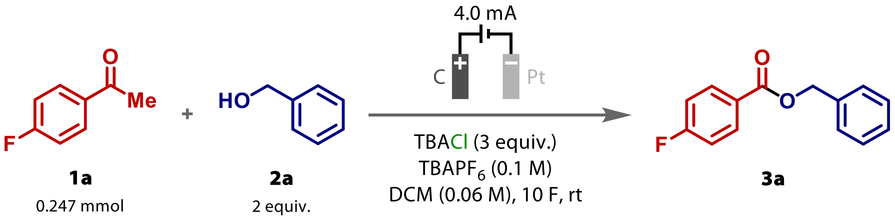

To a small, oven-dried, beaker-type, undivided electrolysis cell, equipped with a small stirrer bar, a graphite rod anode, a platinum coil cathode and a Ag/AgNO$_3$ reference electrode, connected to a potentiostat, was added 4'-fluoroacetophenone **1a** (30.0 µL, 0.247 mmol, 1 equiv.), benzyl alcohol **2a** (51.1 µL, 0.494 mmol, 2 equiv.), TBACl (206 mg, 0.741 mmol, 3 equiv.), TBAPF$_6$ (155 mg, 0.400 mmol, 0.1 M), 4,4'-difluorobiphenyl internal standard (23.5 mg, 0.123 mmol, 0.5 equiv.) and anhydrous DCM (4 mL). A small aliquot (~25 µL) of the resulting solution was taken for ^{19}F NMR analysis. The reaction mixture was then stirred and electrolysed at the 4 mA until 10 *F* had been passed. On completion, an NMR sample was prepared by diluting a 100-µL aliquot of reaction mixture with CDCl$_3$ (400 µL). ^{19}F NMR yield was calculated by comparison to the **1a**:internal standard ratio prior to the reaction.

Optimisation

Procedure

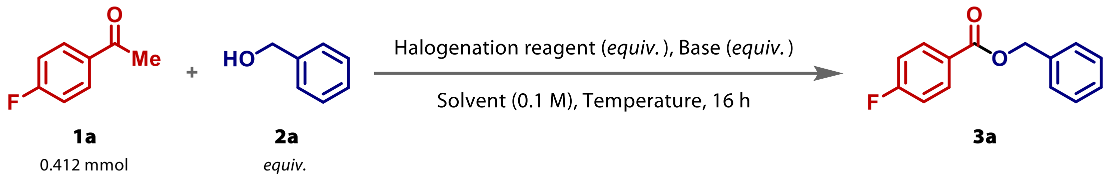

To an oven-dried Schlenk tube, which had been evacuated and refilled with nitrogen (×3), was added 4’-fluoroacetophenone **1a** (50.0 µL, 0.412 mmol, 1 equiv.), benzyl alcohol **2a**, 4,4’-difluorobiphenyl internal standard (23.5 mg, 0.124 mmol, 0.3 equiv.) and solvent (4.1 mL). A small aliquot (~25 µL) of the resulting solution was taken for ^{19}F NMR analysis, then the halonium source was added in one portion. The reaction mixture was cooled to 0 °C (ice-water bath),[a] then base was added dropwise.[b] The reaction mixture was stirred for 16 h, with the reaction allowed to warm to room temperature. On completion, an NMR sample was prepared by diluting a 100-µL aliquot of reaction mixture with $CDCl_3$ (400 µL). ^{19}F NMR yields were calculated by comparison to the **1a**:internal standard ratio prior to the reaction.

a: For reactions at room temperature (Table S2, entries 1 and 12): the reaction mixture was not cooled to 0 °C.

b: For reaction with *t*-BuOK (Table S2, entry 1): a different order of addition was followed: *t*-BuOK and *t*-BuOH (3 mL), followed by iodine. The reaction mixture was then stirred for 5 min, followed by addition of benzyl alcohol **2a**, and dropwise addition of 4’-fluoroacetophenone **1a** and 4,4’-difluorobiphenyl internal standard in *t*-BuOH (1.1 mL).

Selected Results

Table S2. Effect of varying conditions on yield of haloform coupling with primary alcohol **2a**.[a]

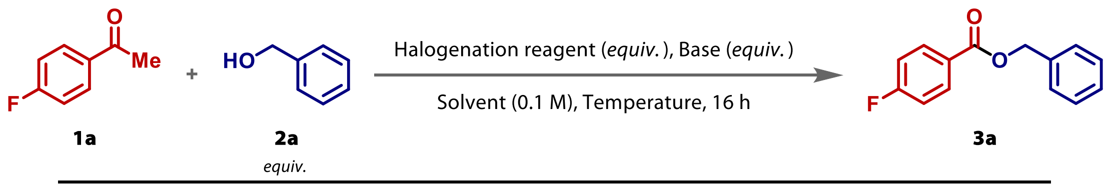

Entry	Halogenation reagent (equiv.)	Base (equiv.)	BnOH **2a** equiv.	Solvent	Temperature	Yield[b]
1	I_2 (3)	*t*-BuOK (9)	3	*t*-BuOH	rt	31%
2	I_2 (3.03)	Pyridine (4.04)	1.05	DCM	0 °C to rt	0%
3	I_2 (3.03)	2,6-Lutidine (4.04)	1.05	DCM	0 °C to rt	0%
4	I_2 (3.03)	DBU (4.04)	1.05	DCM	0 °C to rt	96%
5	Br_2 (3.03)	DBU (4.04)	1.05	DCM	0 °C to rt	11%
6	$TBAI_3$ (3.5)	DBU (4.5)	2	DCM	0 °C to rt	99%
7	$TBABr_3$ (3.5)	DBU (4.5)	2	DCM	0 °C to rt	99%
8	I_2 (3.03)	DBU (4.04)	1.05	Et_2O	0 °C to rt	9%
9	I_2 (3.03)	DBU (4.04)	1.05	DMSO	0 °C to rt	17%
10	I_2 (3.03)	DBU (4.04)	1.05	MeCN	0 °C to rt	77%
11	I_2 (3.03)	DBU (4.04)	1.05	THF	0 °C to rt	65%
12	I_2 (3.5)	DBU (4.5)	2	DCM	0 °C to rt	>99%
13	I_2 (3.5)	DBU (4.5)	1.05	DCM	0 °C to rt	>99%
14	I_2 (3.03)	DBU (4.04)	1.05	DCM	rt	87%

a: On 0.412 mmol scale. b: Calculated by ^{19}F NMR vs 4,4'-difluorobiphenyl internal standard.

Scope

General Procedure A

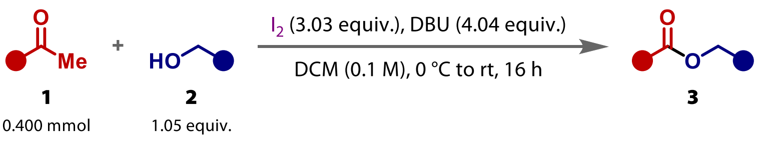

To an oven-dried Schlenk tube, which had been evacuated and refilled with nitrogen (×3), was added methyl ketone **1** (0.400 mmol, 1 equiv.), primary alcohol **2** (0.420 mmol, 1.05 equiv.) and anhydrous DCM (4 mL), followed by addition of iodine (308 mg, 1.21 mmol, 3.03 equiv.) in one portion. The reaction mixture was cooled to 0 °C (ice-water bath), then DBU (241 µL, 1.62 mmol, 4.04 equiv.) was added dropwise. The reaction mixture was stirred for 16 h and allowed to warm to room temperature. The reaction mixture was diluted with EtOAc, filtered, then added to sat. aq. $Na_2S_2O_3$. The mixture was separated and the aqueous layer was extracted a further two times with EtOAc. The combined organic fractions were dried over anhydrous $MgSO_4$, filtered, and concentrated under vacuum. The crude product was purified by FCC (eluted with EtOAc in pentane).

Benzyl 4-fluorobenzoate, **3a**

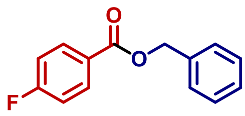

Ester **3a** was synthesised following Procedure A from 4'-fluoroacetophenone **1a** and benzyl alcohol **2a**, and was isolated as a colourless oil (71.6 mg, 78%).

^{1}H NMR (400 MHz, CDCl$_3$) δ 8.13 – 8.06 (m, 2H), 7.47 – 7.32 (m, 5H), 7.14 – 7.07 (m, 2H), 5.36 (s, 2H).

^{13}C NMR (101 MHz, CDCl$_3$) δ 166.0 (d, *J* = 254.0 Hz), 165.6, 136.1, 132.4 (d, *J* = 9.5 Hz), 128.8, 128.5, 128.4, 126.5 (d, *J* = 3.0 Hz), 115.7 (d, *J* = 22.0 Hz), 67.0.

^{19}F NMR (376 MHz, CDCl$_3$) δ −105.55 (tt, *J* = 8.5, 5.5 Hz).

Data are consistent with those previously reported (compound 10).[6]

Benzyl 4-nitrobenzoate, **3b**

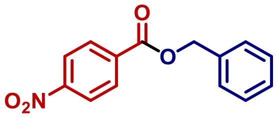

Ester **3b** was synthesised following Procedure A from 4'-nitroacetophenone and benzyl alcohol **2a**, and was isolated as a white solid (95.9 mg, 93%).

^{1}H NMR (400 MHz, CDCl$_3$) δ 8.31 – 8.21 (m, 4H), 7.49 – 7.35 (m, 5H), 5.41 (s, 2H).

^{13}C NMR (101 MHz, CDCl$_3$) δ 164.7, 150.7, 135.6, 135.4, 131.0, 128.9, 128.8, 128.6, 123.7, 67.8.

Data are consistent with those previously reported (compound 2h).[7]

4-Nitrobenzyl 4-nitrobenzoate, **3c**

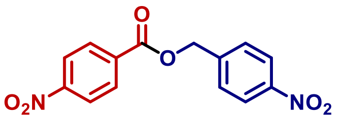

Ester **3c** was synthesised following Procedure A from 4'-nitroacetophenone and 4-nitrobenzyl alcohol, and was isolated as a white solid (95.1 mg, 79%).

^{1}H NMR (400 MHz, CDCl$_3$) δ 8.34 – 8.22 (m, 6H), 7.64 – 7.60 (m, 2H), 5.50 (s, 2H).

^{13}C NMR (101 MHz, CDCl$_3$) δ 164.4, 151.0, 148.1, 142.5, 135.0, 131.0, 128.8, 124.1, 123.9, 66.2.

Data are consistent with those previously reported (compound 4hh).[8]

3,4-Dimethoxybenzyl 4-nitrobenzoate, **3d**

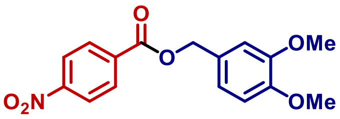

Ester **3d** was synthesised following Procedure A from 4'-nitroacetophenone and 3,4-dimethoxybenzyl alcohol, and was isolated as a yellow solid (77.0 mg, 61%).

^{1}H NMR (400 MHz, CDCl$_3$) δ 8.24 (d, J = 9.0 Hz, 2H), 8.19 (d, J = 9.0 Hz, 2H), 7.02 (dd, J = 8.0, 2.0 Hz, 1H), 6.96 (d, J = 2.0 Hz, 1H), 6.87 (d, J = 8.0 Hz, 1H), 5.32 (s, 2H), 3.89 (s, 3H), 3.87 (s, 3H).

^{13}C NMR (101 MHz, CDCl$_3$) δ 164.6, 150.6, 149.5, 149.1, 135.6, 130.8, 127.7, 123.6, 121.7, 112.0, 111.1, 67.9, 56.0, 56.0.

Data are consistent with those previously reported (compound 3al).[9]

Naphthalen-1-ylmethyl 4-nitrobenzoate, **3e**

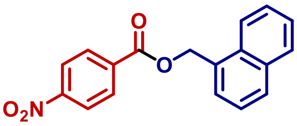

Ester **3e** was synthesised following Procedure A from 4'-nitroacetophenone and naphthalen-1-ylmethanol, and was isolated as a white solid (104 mg, 84%).

^{1}H NMR (400 MHz, CDCl$_3$) δ 8.25 – 8.16 (m, 4H), 8.11 (d, J = 9.0 Hz, 1H), 7.94 – 7.88 (m, 2H), 7.67 – 7.46 (m, 4H), 5.86 (s, 2H).

^{13}C NMR (101 MHz, CDCl$_3$) δ 164.7, 150.6, 135.5, 133.9, 131.8, 130.9, 130.8, 129.9, 129.0, 128.1, 126.9, 126.2, 125.4, 123.6, 123.5, 66.1.

Data are consistent with those previously reported (compound 2k).[10]

*2-(4-Chlorophenoxy)ethyl 4-nitrobenzoate, **3f***

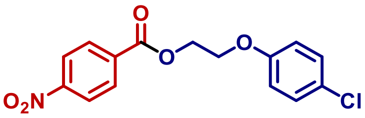

Ester **3f** was synthesised following Procedure A from 4'-nitroacetophenone and 2-(4-chlorophenoxy)ethan-1-ol, and was isolated as a colourless oil (86.8 mg, 67%).

^{1}H NMR (400 MHz, CDCl$_3$) δ 8.29 – 8.24 (m, 2H, Ar-H), 8.22 – 8.18 (m, 2H, Ar-H), 7.26 – 7.21 (m, 2H, Ar-H), 6.89 – 6.84 (m, 2H, Ar-H), 4.71 (t, J = 4.5 Hz, 2H, CH$_2$), 4.30 (t, J = 4.5 Hz, 2H, CH$_2$).

^{13}C NMR (101 MHz, CDCl$_3$) δ 164.7 (C=O), 157.1 (Ar), 150.7 (Ar), 135.2 (Ar), 131.0 (Ar), 129.6 (Ar), 126.4 (Ar), 123.7 (Ar), 116.0 (Ar), 66.1 (CH$_2$), 64.2 (CH$_2$).

HRMS (EI) m/z: [M]$^+$ Calcd for $C_{15}H_{12}NO_5Cl$ 321.0399; found 321.0397; 0.62 ppm error.

IR (neat) ν_{max} / cm^{-1}: 2989, 2968, 1728, 1525, 1492, 1349, 1271, 1244, 1102, 1058, 910, 718.

*(E)-Hex-3-en-1-yl 4-nitrobenzoate, **3g***

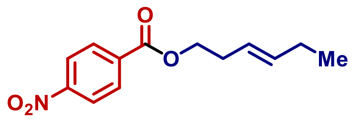

Ester **3g** was synthesised following Procedure A from 4'-nitroacetophenone and (*E*)-hex-3-en-1-ol, and was isolated as a yellow oil (35.8 mg, 36%).

^{1}H NMR (400 MHz, $CDCl_3$) δ 8.30 – 8.26 (m, 2H, Ar-H), 8.22 – 8.17 (m, 2H, Ar-H), 5.62 (dtt, *J* = 15.5, 6.5, 1.5 Hz, 1H, 3-H), 5.43 (dtt, *J* = 15.0, 7.0, 1.5 Hz, 1H, 4-H), 4.37 (t, *J* = 7.0 Hz, 2H, 1-H_2), 2.47 (qd, *J* = 7.0, 1.0 Hz, 2H, 5-H_2), 2.06 – 1.97 (m, 2H, 2-H_2), 0.95 (t, *J* = 7.5 Hz, 3H, 6-H_3).

^{13}C NMR (101 MHz, $CDCl_3$) δ 164.8 (C=O), 150.6 (Ar), 136.0 (C3), 135.8 (Ar), 130.8 (Ar), 123.8 (C4), 123.6 (Ar), 65.6 (C1), 32.1 (C5), 25.8 (C2), 13.9 (C6).

HRMS (EI) m/z: $[M+H]^+$ Calcd for $C_{13}H_{16}NO_4$ 250.1074; found 250.1070; 1.60 ppm error.

IR (neat) v_{max} / cm^{-1}: 3668, 2966, 2901, 1722, 1528, 1274, 1103, 908, 731.

Octyl 4-nitrobenzoate, **3h**

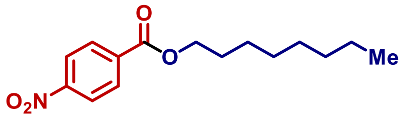

Ester **3h** was synthesised following Procedure A from 4'-nitroacetophenone and 1-octanol, and was isolated as a colourless oil (71.0 mg, 61%).

^{1}H NMR (400 MHz, $CDCl_3$) δ 8.31 – 8.26 (m, 2H), 8.23 – 8.18 (m, 2H), 4.37 (t, *J* = 6.5 Hz, 2H), 1.79 (app quint, *J* = 7.0 Hz, 2H), 1.49 – 1.22 (m, 10H), 0.88 (t, *J* = 7.0 Hz, 3H).

^{13}C NMR (101 MHz, $CDCl_3$) δ 164.9, 150.6, 136.1, 130.8, 123.7, 66.3, 31.9, 29.4, 29.3, 28.8, 26.1, 22.8, 14.2.

Data are consistent with those previously reported (compound 3au).[11]

Furan-2-ylmethyl 4-nitrobenzoate, **3i**

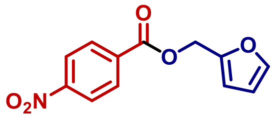

Ester **3i** was synthesised following Procedure A from 4'-nitroacetophenone and furan-2-ylmethanol, and was isolated as a yellow oil (19.2 mg, 19%).

^{1}H NMR (400 MHz, $CDCl_3$) δ 8.30 – 8.25 (m, 2H), 8.24 – 8.19 (m, 2H), 7.47 (dd, *J* = 2.0, 1.0 Hz, 1H), 6.52 (d, *J* = 3.5 Hz, 1H), 6.41 (dd, *J* = 3.5, 2.0 Hz, 1H), 5.35 (s, 2H).

^{13}C NMR (101 MHz, $CDCl_3$) δ 164.5, 150.7, 148.9, 143.8, 135.4, 131.0, 123.7, 111.6, 110.9, 59.4.

Data are consistent with those previously reported (compound 3at).[9]

Pyridin-4-ylmethyl 4-nitrobenzoate, **3j**

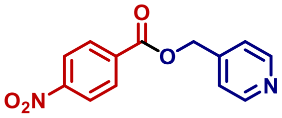

Ester **3j** was synthesised following Procedure A from 4'-nitroacetophenone and pyridin-4-ylmethanol, and was isolated as a pink solid (78.5 mg, 76%).

^{1}H NMR (400 MHz, $CDCl_3$) δ 8.65 – 8.60 (m, 2H), 8.31 – 8.27 (m, 2H), 8.26 – 8.22 (m, 2H), 7.34 – 7.30 (m, 2H), 5.40 (s, 2H).

^{13}C NMR (101 MHz, $CDCl_3$) δ 164.3, 150.8, 150.3, 144.2, 134.9, 131.0, 123.8, 122.1, 65.6.

Data are consistent with those previously reported (compound 4g).[12]

Benzyl 2-methylbenzoate, **3k**

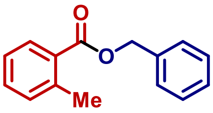

Ester **3k** was synthesised following Procedure A from 2-methylacetophenone and benzyl alcohol **2a**, and was isolated as a colourless oil (36.5 mg, 40%).

^{1}H NMR (400 MHz, $CDCl_3$) δ 7.97 (d, *J* = 8.0 Hz, 1H), 7.49 – 7.32 (m, 6H), 7.28 – 7.20 (m, 2H), 5.36 (s, 2H), 2.62 (s, 3H).

^{13}C NMR (101 MHz, $CDCl_3$) δ 167.5, 140.5, 136.3, 132.2, 131.8, 130.8, 129.6, 128.7, 128.3, 125.9, 66.6, 22.0.

Data are consistent with those previously reported (compound 3).[13]

Benzyl cinnamate, **3l**

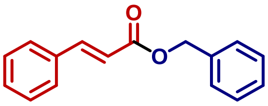

Ester **3l** was synthesised following Procedure A from (*E*)-4-phenylbut-3-en-2-one and benzyl alcohol **2a**, and was isolated as a colourless oil (76.4 mg, 80%).

^{1}H NMR (400 MHz, $CDCl_3$) δ 7.75 (d, *J* = 16.0 Hz, 1H), 7.56 – 7.50 (m, 2H), 7.46 – 7.33 (m, 8H), 6.51 (d, *J* = 16.0 Hz, 1H), 5.27 (s, 2H).

^{13}C NMR (101 MHz, $CDCl_3$) δ 166.9, 145.3, 136.2, 134.5, 130.5, 129.0, 128.7, 128.4, 128.4, 128.2, 118.0, 66.5.

Data are consistent with those previously reported (compound 1g).[14]

Benzyl (*E*)-oct-2-enoate, **3m**

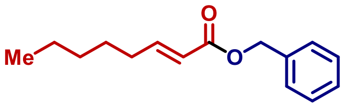

Ester **3m** was synthesised following Procedure A from (*E*)-non-3-en-2-one and benzyl alcohol **2a**, and was isolated as a colourless oil (71.2 mg, 77%).

^{1}H NMR (400 MHz, $CDCl_3$) δ 7.40 – 7.30 (m, 5H), 7.03 (dt, *J* = 15.5, 7.0 Hz, 1H), 5.87 (dt, *J* = 15.5, 1.5 Hz, 1H), 5.18 (s, 2H), 2.20 (app qd, *J* = 7.0, 1.5 Hz, 2H), 1.51 – 1.41 (m, 2H), 1.35 – 1.25 (m, 4H), 0.90 (t, *J* = 7.0 Hz, 3H).

^{13}C NMR (101 MHz, $CDCl_3$) δ 166.7, 150.4, 136.3, 128.7, 128.3, 128.3, 121.0, 66.1, 32.4, 31.4, 27.8, 22.5, 14.1.

Data are consistent with those previously reported (compound 3aa).[15]

Mechanistic Experiments and Kinetic Modelling

Competition Experiment

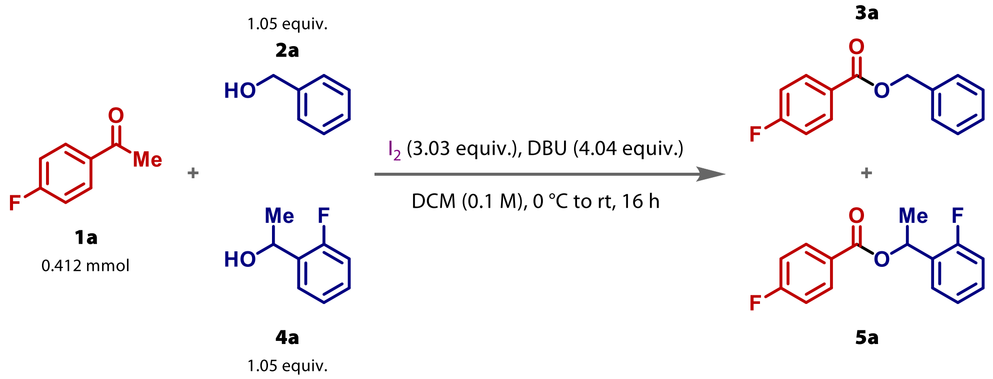

To an oven-dried Schlenk tube, which had been evacuated and refilled with nitrogen (×3), was added 4'-fluoroacetophenone **1a** (50.0 μL, 0.412 mmol, 1 equiv.), benzyl alcohol **2a** (44.8 μL, 0.432 mmol, 1.05 equiv.), 1-(2-fluorophenyl)ethanol **4a** (54.6 μL, 0.432 mmol, 1.05 equiv.), 4,4'-difluorobiphenyl internal standard (23.5 mg, 0.124 mmol, 0.3 equiv.) and anhydrous DCM (4.1 mL). A small aliquot (~25 μL) of the resulting solution was taken for ^{19}F NMR analysis, then iodine (319 mg, 1.26 mmol, 3.03 equiv.) was added in one portion. The reaction mixture was cooled to 0 °C (ice-water bath) and DBU (249 μL, 1.67 mmol, 4.04 equiv.) was added dropwise. The reaction mixture was stirred for 16 h and allowed to warm to room temperature. On completion, an NMR sample was prepared by diluting a 100-μL aliquot of reaction mixture with $CDCl_3$ (400 μL). ^{19}F NMR yields were calculated by comparison to the **1a**:internal standard ratio prior to the reaction.

Trichloroacetophenone Substitution Time Courses

Reaction Procedure

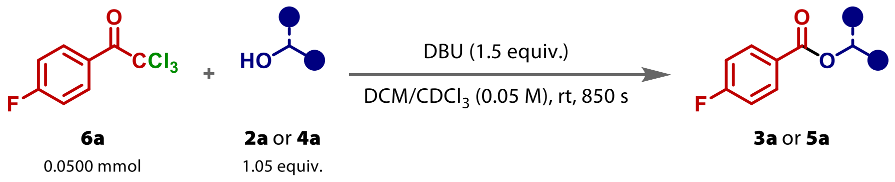

To an oven-dried Schlenk tube containing activated 3 Å molecular sieves (300 mg), which had been evacuated and refilled with nitrogen (×3), was added 2,2,2-trichloro-4'-fluoroacetophenone **6a** (36.2 mg, 0.150 mmol, 3 equiv.), alcohol **2a**/**4a** (0.158 mmol, 3.15 equiv.), 4,4'-difluorobiphenyl internal standard (14.3 mg, 0.0750 mmol, 1.5 equiv.), anhydrous DCM (1.2 mL) and anhydrous $CDCl_3$ (1.8 mL). A 1-mL aliquot of the resulting solution (corresponding to 0.0500 mmol **6a**, 0.0525 mmol **2a**/**4a** and 0.0250 mmol internal standard) was transferred to an NMR tube under nitrogen and analysed by ^{19}F NMR to determine the **6a**:internal standard ratio prior to reaction. DBU (11.2 μL, 0.0750 mmol, 1.5 equiv.) was added to the NMR tube in one portion (open to air), which was shaken to mix and then monitored by ^{19}F NMR (spectra acquired at 10 s intervals).

Substrate Synthesis

2,2,2-Trichloro-4'-fluoroacetophenone, **6a**

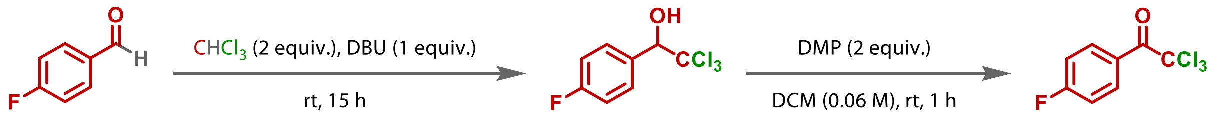

Synthesised by modified literature procedures.[16,17] To a round-bottom flask, which had been evacuated and refilled with nitrogen (×3), was added 4-fluorobenzaldehyde (600 µL, 5.59 mmol, 1 equiv.) and $CHCl_3$ (895 µL, 11.2 mmol, 2 equiv.), followed by dropwise addition of DBU (836 µL, 5.59 mmol, 1 equiv.). The reaction mixture was stirred for 15 h, then diluted with $CHCl_3$ and washed with 2 M aq. HCl. The organic layer was dried over anhydrous $MgSO_4$, filtered, and concentrated under vacuum to obtain crude 2,2,2-trichloro-1-(4-fluorophenyl)ethanol as a yellow oil (1.31 g), which was used in the following step without further purification.

To a round-bottom flask containing crude 2,2,2-trichloro-1-(4-fluorophenyl)ethanol (1.31 g) in DCM (100 mL) was added Dess-Martin periodinane (DMP; 4.74 g, 11.2 mmol, 2 equiv.). The reaction mixture was stirred for 1 h, then the reaction mixture was filtered through Celite and the filtrate was concentrated under vacuum. The crude product was purified by FCC (eluted with 20% EtOAc in hexane) to obtain the title compound as a pale-yellow oil (1.15 g, 85%).

^{1}H NMR (400 MHz, $CDCl_3$) δ 8.35 – 8.28 (m, 2H), 7.21 – 7.13 (m, 2H).

^{13}C NMR (101 MHz, $CDCl_3$) δ 179.9, 166.3 (d, J = 258.5 Hz), 134.6 (d, J = 9.5 Hz), 125.3 (d, J = 3.0 Hz), 115.9 (d, J = 22.0 Hz), 95.4.

^{19}F NMR (376 MHz, $CDCl_3$) δ −101.77 (tt, J = 8.0, 5.0 Hz).

Data are consistent with those previously reported (compound 4b).[18] *As ^{19}F NMR data have not previously been reported, a copy of the spectrum is included.*

Haloform Reaction Time Courses

Reaction Procedure

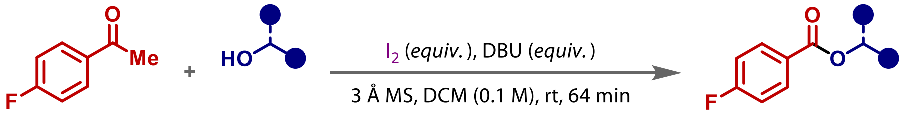

To an oven-dried Schlenk tube containing activated 3 Å molecular sieves (400 mg), which had been evacuated and refilled with nitrogen (×3), was added 4’-fluoroacetophenone **1a** (48.6 µL, 0.400 mmol, 1 equiv.), alcohol **2a**/**4a** (if added; 0.420 mmol, 1.05 equiv.), 4,4’-difluorobiphenyl internal standard (23.5 mg, 0.124 mmol, 0.3 equiv.) and anhydrous DCM (4 mL).[a] A small aliquot (~25 µL) of the resulting solution was taken for ^{19}F NMR analysis, then iodine was added in one portion. DBU was then added in one portion and ~250-µL aliquots of the reaction mixture were taken after 1, 2, 4, 8, 16, 32 and 64 min. Aliquots were immediately quenched with sat. aq. NH_4Cl (~0.7 mL) and NMR samples were prepared by diluting a 100-µL aliquot of the quenched reaction mixture with $CDCl_3$ (400 µL). ^{19}F NMR yields were calculated by comparison to the **1a**:internal standard ratio prior to reaction. NB: NMR samples were protected from exposure to light, due to the light sensitivity of reaction intermediates.

a: For reactions with DBU•HI added: DBU•HI was added to the reaction mixture in one portion immediately after the addition of DBU.

Synthesis of DBU•HI Salt

1,8-Diazabicyclo[5.4.0]undec-7-enium iodide

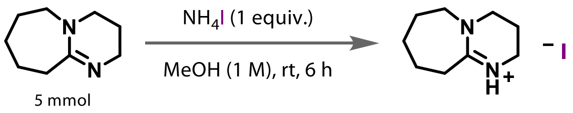

Synthesised and isolated following a literature procedure[19] from DBU (747 μL, 5.00 mmol) as a pale-yellow solid (1.40 g, 100%).

^{1}H NMR (400 MHz, CDCl$_3$) δ 9.57 (br s, 1H), 3.65 – 3.58 (m, 4H), 3.52 – 3.46 (m, 2H), 3.03 – 2.98 (m, 2H), 2.12 (quint, *J* = 6.0 Hz, 2H), 1.84 – 1.74 (m, 6H).

^{13}C NMR (101 MHz, CDCl$_3$) δ 166.0, 54.8, 48.8, 37.8, 32.7, 28.9, 26.6, 23.7, 19.3.

Data are consistent with those previously reported (compound 1a•HI).[19]

Synthesis of Intermediates and Other Haloacetophenones

2-Iodo-4'-fluoroacetophenone, **7a**

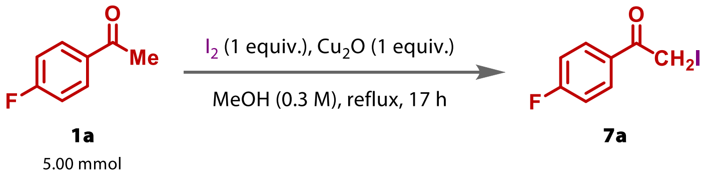

Synthesised by a modified literature procedure.[20] To a round-bottom flask was added 4'-fluoroacetophenone **1a** (607 μL, 5.00 mmol, 1 equiv.) and MeOH (20 mL), followed by finely-powdered Cu_2O (398 mg, 5.00 mmol, 1 equiv.) and iodine (1.27 g, 5.00 mmol, 1 equiv.). The reaction mixture was stirred for 17 h under reflux, then allowed to cool to room temperature, filtered, and concentrated under vacuum. The residue was redissolved in sat. aq. $Na_2S_2O_3$, then extracted with EtOAc (×3), dried over anhydrous $MgSO_4$, filtered, and concentrated under vacuum. The crude product was purified by FCC (eluted with 0-6% EtOAc in pentane) to obtain the title compound as a yellow oil (829 mg, 63%), which became and remained a yellow solid on freezing (at −20 °C). NB: **7a** is light-sensitive, but storage at −20 °C is not believed to be necessary.

^{1}H NMR (400 MHz, CDCl$_3$) δ 8.05 – 7.98 (m, 2H), 7.19 – 7.11 (m, 2H), 4.33 (s, 2H).

^{13}C NMR (101 MHz, CDCl$_3$) δ 191.4, 166.2 (d, *J* = 256.5 Hz), 131.9 (d, *J* = 9.5 Hz), 130.0 (d, *J* = 3.0 Hz), 116.2 (d, *J* = 22.0 Hz), 1.3.

^{19}F NMR (376 MHz, CDCl$_3$) δ −103.50 (tt, *J* = 8.5, 5.5 Hz).

Data are consistent with those previously reported (compound 2x).[21]

2,2-Diiodo-1-(4-fluorophenyl)ethanol

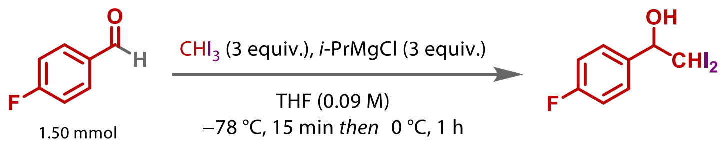

Synthesised by a modified literature procedure.[22] To an oven-dried 2-neck round-bottom flask, which had been evacuated and refilled with nitrogen (×3), was added CHI_3 (1.77 g, 4.50 mmol, 3 equiv.) and anhydrous THF (15 mL). The resulting solution was cooled to −78 °C (dry ice-acetone bath), then *i*-PrMgCl (2.0 M in THF; 2.25 mL, 4.50 mmol, 3 equiv.) was added dropwise, followed by dropwise addition of a solution of 4-fluorobenzaldehyde (162 µL, 1.50 mmol, 1 equiv.) in anhydrous THF (1.5 mL), prepared in a separate oven-dried Schlenk tube, which had been evacuated and refilled with nitrogen (×3). The reaction mixture was stirred for 15 min at −78 °C, then allowed to warm to 0 °C (ice-water bath) and stirred for a further 1 h. After confirming complete conversion (by TLC), sat. aq. NH_4Cl was added and the mixture was extracted with DCM (×3). The combined organic fractions were dried over anhydrous $MgSO_4$, filtered, and concentrated under vacuum. The crude product was purified by FCC (eluted with 0-15% EtOAc in pentane) to obtain the title compound as a yellow oil (378 mg, 64%). NB: 2,2-Diiodo-1-(4-fluorophenyl)ethanol may be light-sensitive.

^{1}H NMR (400 MHz, $CDCl_3$) δ 7.44 – 7.36 (m, 2H, Ar-H), 7.10 – 7.02 (m, 2H, Ar-H), 5.28 (d, *J* = 4.5 Hz, 1H, CHI_2), 4.68 (app t, *J* = 4.0 Hz, 1H, C*H*OH), 2.88 (d, *J* = 4.0 Hz, 1H, OH).

^{13}C NMR (101 MHz, $CDCl_3$) δ 162.9 (d, *J* = 248.0 Hz, Ar), 134.9 (d, *J* = 3.0 Hz, Ar), 128.5 (d, *J* = 8.5 Hz, Ar), 115.6 (d, *J* = 21.5 Hz, Ar), 79.1 (CHOH), −10.8 (d, *J* = 2.0 Hz, CHI_2).

^{19}F NMR (376 MHz, $CDCl_3$) δ −112.46 (tt, *J* = 8.5, 5.0 Hz).

HRMS (EI) m/z: $[M]^+$ Calcd for $C_8H_7OFI_2$ 391.8565; found 391.8565; 0.00 ppm error.

IR (neat) ν_{max} / cm^{-1}: 3443, 1603, 1508, 1227, 1073, 836.

2,2-Diiodo-4'-fluoroacetophenone, **8a**

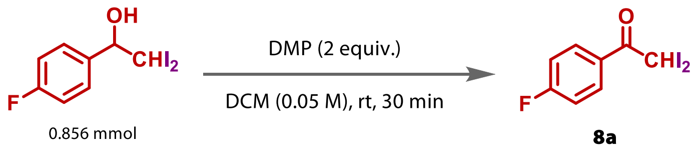

8a

To a round-bottom flask was added 2,2-diiodo-1-(4-fluorophenyl)ethanol (336 mg, 0.856 mmol, 1 equiv.), DMP (727 mg, 1.71 mmol, 2 equiv.) and DCM (17 mL). The reaction mixture was stirred for 30 min, then filtered through a short plug of silica (washed with DCM) and the filtrate was concentrated under vacuum to obtain the title compound as a yellow oil (254 mg, 76%). NB: **8a** is light-sensitive.

^{1}H NMR (400 MHz, $CDCl_3$) δ 8.12 – 8.05 (m, 2H, Ar-H), 7.19 – 7.12 (m, 2H, Ar-H), 6.44 (s, 1H, CHI_2).

^{13}C NMR (101 MHz, $CDCl_3$) δ 186.9 (C=O), 166.3 (d, *J* = 257.5 Hz, Ar), 132.5 (d, *J* = 9.5 Hz, Ar), 125.1 (d, *J* = 3.0 Hz, Ar), 116.4 (d, *J* = 22.0 Hz, Ar), −29.7 (CHI_2).

^{19}F NMR (376 MHz, $CDCl_3$) δ −102.42 (tt, J = 8.0, 5.0 Hz).

HRMS (EI) m/z: $[M]^+$ Calcd for $C_8H_5OFI_2$ 389.8408; found 389.8406; 0.51 ppm error.

IR (neat) ν_{max} / cm^{-1}: 1677, 1599, 1505, 1253, 1159, 980, 847, 577.

*^{13}C NMR data are inconsistent with those in the only previous report (compound 2aa),[23] so **8a** was fully characterised. Copies of NMR spectra are included.*

Comparison of Haloacetophenone ^{19}F NMR Shifts:

Table S3. Measured ^{19}F NMR shifts (in ppm) of halo-substituted 4'-fluoroacetophenones in 4:1 $CDCl_3$:DCM, referenced to 4,4'-difluorobiphenyl (−115.96 ppm).

	Unsubstituted (–CH_3)	Monosubstituted (–CH_2X)	Disubstituted (–CHX_2)	Trisubstituted (–CX_3)
X = Cl	−105.63	−103.36	−101.93	−102.17
X = Br	−105.63	−103.44	−102.12	−102.62
X = I	−105.63	−103.75	−102.68	*−102.07*[a]

a: Shift of signal observed in haloform time course experiments assigned as 2,2,2-triiodo-4'-fluoroacetophenone **9a**.

2-Chloro-4'-fluoroacetophenone

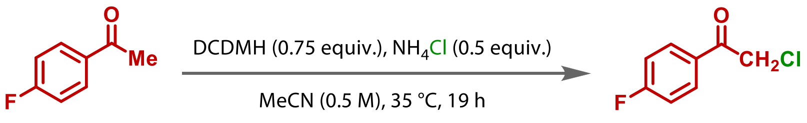

Synthesis based on a literature procedure.[24] To a round-bottom flask was added 4'-fluoroacetophenone **1a** (607 µL, 5.00 mmol, 1 equiv.), NH_4Cl (134 mg, 2.50 mmol, 0.5 equiv.) and MeCN (10 mL). The resulting solution was stirred for 10 min, then 1,3-dichloro-5,5-dimethylhydantoin (DCDMH; 739 mg, 3.75 mmol, 0.75 equiv.) was added in five portions over 50 min. After the final addition, the reaction mixture was stirred for 19 h at 35 °C. On completion, the reaction mixture was concentrated under vacuum, then re-dissolved in EtOAc, washed with deionised water (×2), dried over anhydrous $MgSO_4$, filtered, and concentrated under vacuum. The crude product was purified by FCC (eluted with 20% Et_2O in hexane) to obtain the title compound as a white solid (607 mg, 70%).

^{1}H NMR (400 MHz, $CDCl_3$) δ 8.05 – 7.96 (m, 2H), 7.22 – 7.14 (m, 2H), 4.67 (s, 2H).

^{13}C NMR (101 MHz, $CDCl_3$) δ 189.8, 166.3 (d, *J* = 256.5 Hz), 131.5 (d, *J* = 9.5 Hz), 130.8, 116.3 (d, *J* = 22.0 Hz), 45.8.

^{19}F NMR (376 MHz, $CDCl_3$) δ −102.93 (tt, *J* = 8.5, 5.5 Hz).

Data are consistent with those previously reported (compound 3g).[25]

2,2-Dichloro-4'-fluoroacetophenone

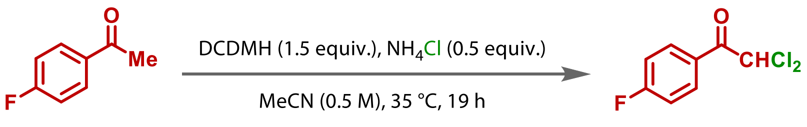

Synthesised by a modified literature procedure.[24] To a round-bottom flask was added 4'-fluoroacetophenone **1a** (607 µL, 5.00 mmol, 1 equiv.), NH_4Cl (134 mg, 2.50 mmol, 0.5 equiv.) and MeCN (10 mL). The resulting solution was stirred for 10 min, then 1,3-dichloro-5,5-dimethylhydantoin (DCDMH; 1.48 g, 7.5 mmol, 1.5 equiv.) was added in four portions over 35 min. After the final addition, the reaction mixture was stirred for 19 h at 35 °C. On completion, the reaction mixture was concentrated under vacuum, then re-dissolved in EtOAc, washed with deionised water (×2), dried over anhydrous $MgSO_4$, filtered, and concentrated under vacuum. The crude product was purified by FCC (eluted with 5-10% Et_2O in hexane) to obtain the title compound as a pale-yellow oil (913 mg, 88%).

^{1}H NMR (400 MHz, $CDCl_3$) δ 8.17 – 8.10 (m, 2H), 7.21 – 7.14 (m, 2H), 6.63 (s, 1H).

^{13}C NMR (101 MHz, $CDCl_3$) δ 184.7, 166.6 (d, *J* = 258.0 Hz), 132.9 (d, *J* = 9.5 Hz), 127.7 (d, *J* = 3.0 Hz), 116.4 (d, *J* = 22.5 Hz), 68.0.

^{19}F NMR (376 MHz, $CDCl_3$) δ −101.68 (tt, *J* = 8.5, 5.0 Hz).

Data are consistent with those previously reported (compound 2e).[26] *As ^{19}F NMR data have not previously been reported, a copy of the spectrum is included.*

2-Bromo-4'-fluoroacetophenone

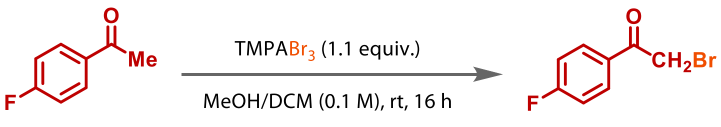

To a round-bottom flask was added 4'-fluoroacetophenone **1a** (121 µL, 1.00 mmol, 1 equiv.), trimethylphenylammonium tribromide ($TMPABr_3$; 414 mg, 1.10 mmol, 1.1 equiv.), MeOH (2 mL) and DCM (5 mL). The resulting solution was stirred for 16 h, then deionised water was added and the mixture was extracted with DCM (×3), washed with brine, dried over anhydrous $MgSO_4$, filtered, and concentrated under vacuum. The crude product contained a 91:9 mixture of the mono- and dibrominated products and was combined with the crude product from an attempted dibromination (containing a 60:40 mixture of the mono- and dibrominated products). The combined crudes were purified by FCC (eluted with 5% EtOAc in hexane) to obtain the title compound as a white solid (312 mg, yield incalculable).

^{1}H NMR (400 MHz, $CDCl_3$) δ 8.07 – 7.99 (m, 2H), 7.21 – 7.14 (m, 2H), 4.41 (s, 2H).

^{13}C NMR (101 MHz, $CDCl_3$) δ 190.0, 166.3 (d, *J* = 256.5 Hz), 131.9 (d, *J* = 9.5 Hz), 130.5 (d, *J* = 3.0 Hz), 116.2 (d, *J* = 22.0 Hz), 30.5.

^{19}F NMR (376 MHz, $CDCl_3$) δ −103.06 (tt, *J* = 8.5, 5.0 Hz).

Data are consistent with those previously reported (compound 2f/s7).[27,28]

2,2-Dibromo-4'-fluoroacetophenone

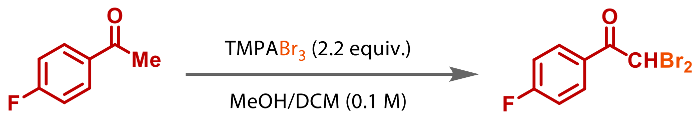

1a
5.00 mmol

To a round-bottom flask was added 4'-fluoroacetophenone **1a** (607 µL, 5.00 mmol, 1 equiv.), trimethylphenylammonium tribromide (TMPABr$_3$; 4.14 g, 11.0 mmol, 2.2 equiv.), MeOH (15 mL) and DCM (37.5 mL) and the resulting solution was stirred for 27 h. TLC showed complete conversion of **1a**, but predominantly to the monobrominated product, so stirring was continued for a further 45 h under reflux. After this time, despite incomplete dibromination, the reaction was cooled to room temperature and deionised water was added. The mixture was extracted with DCM (×3), washed with brine, dried over anhydrous MgSO$_4$, filtered, and concentrated under vacuum. The crude product was purified by FCC (eluted with 5% EtOAc in hexane) to obtain the title compound as a pale-yellow oil (941 mg, 64%).

^{1}H NMR (400 MHz, CDCl$_3$) δ 8.19 – 8.12 (m, 2H), 7.23 – 7.15 (m, 2H), 6.61 (s, 1H).

^{13}C NMR (101 MHz, CDCl$_3$) δ 184.7, 166.4 (d, J = 258.0 Hz), 132.8 (d, J = 9.5 Hz), 127.2 (d, J = 3.0 Hz), 116.3 (d, J = 22.0 Hz), 39.5.

^{19}F NMR (376 MHz, CDCl$_3$) δ −101.77 (tt, J = 8.5, 5.0 Hz).

Data are consistent with those previously reported (compound 3e).[29]

2,2,2-Tribromo-4'-fluoroacetophenone

Method 1:

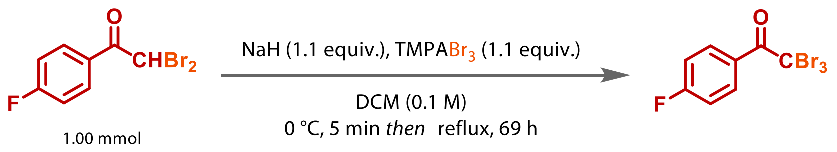

To a round-bottom flask, containing NaH (60% in mineral oil; 44.0 mg, 1.10 mmol, 1.1 equiv.) and DCM (4 mL) at 0 °C (ice-water bath), was added dropwise a solution of 2,2-dibromo-4'-fluoroacetophenone (296 mg, 1.00 mmol, 1 equiv.) in DCM (6 mL). The reaction mixture was stirred for 5 min at 0 °C, then the reaction was allowed to warm to room temperature. After 30 min, trimethylphenylammonium tribromide ($TMPABr_3$; 414 mg, 1.10 mmol, 1.1 equiv.) was added in one portion and the reaction mixture was stirred for 69 h under reflux. After this time, despite incomplete conversion, the reaction was cooled to room temperature, filtered, and concentrated under vacuum. The crude product was purified by FCC (eluted with 0-1% EtOAc in hexane) to obtain the title compound as a yellow oil (136 mg, 36%).

Method 2:

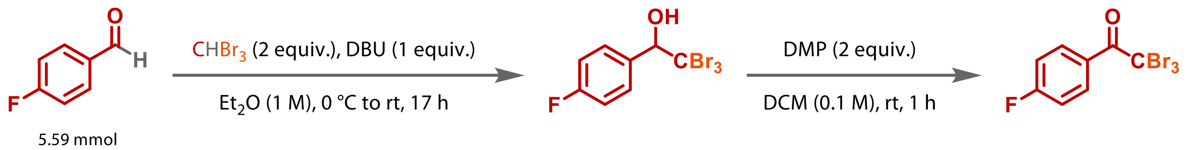

Synthesis based on literature procedures.[16,17] To a round-bottom flask, which had been evacuated and refilled with nitrogen (×3), was added 4-fluorobenzaldehyde (600 µL, 5.59 mmol, 1 equiv.), $CHBr_3$ (978 µL, 11.2 mmol, 2 equiv.) and Et_2O (6 mL). The resulting solution was cooled to 0 °C (ice-water bath), then DBU (836 µL, 5.59 mmol, 1 equiv.) was added dropwise. The reaction mixture was stirred for 17 h and allowed to warm to room temperature, then diluted with DCM and washed with 2 M aq. HCl. The organic layer was dried over anhydrous $MgSO_4$, filtered, and concentrated under vacuum. The crude product was partially purified by FCC (eluted with 10% EtOAc in hexane) to remove high R_f impurities, giving crude 2,2,2-tribromo-1-(4-fluorophenyl)ethanol as a yellow oil (938 mg).

To a round-bottom flask containing crude 2,2,2-tribromo-1-(4-fluorophenyl)ethanol (912 mg) in DCM (45 mL) was added DMP (2.05 g, 4.84 mmol, 2 equiv.). The reaction mixture was stirred for 1 h, then filtered through Celite and the filtrate was concentrated under vacuum. The crude product was purified by FCC (eluted with 1% Et_2O in hexane) to obtain the title compound as a colourless oil (398 mg, 19%).

^{1}H NMR (400 MHz, $CDCl_3$) δ 8.43 – 8.37 (m, 2H), 7.19 – 7.12 (m, 2H).

^{13}C NMR (101 MHz, $CDCl_3$) δ 180.2, 166.1 (d, J = 258.0 Hz), 135.0 (d, J = 9.5 Hz), 124.5 (d, J = 3.5 Hz), 115.7 (d, J = 22.0 Hz), 41.6.

^{19}F NMR (376 MHz, $CDCl_3$) δ −102.43 (tt, J = 8.0, 5.0 Hz).

Data are consistent with those previously reported (compound 2o).[30] As ^{19}F NMR data have not previously been reported, a copy of the spectrum is included.

Evidence of DBU•I_2 Adduct Formation

Spectroscopic Observation

On addition of iodine, the ^{1}H NMR signals of DBU were observed to shift downfield, indicating an interaction between DBU and iodine in solution, i.e. formation of a DBU•I_2 adduct (Figures S1-S2). Furthermore, reaction mixtures containing iodine were observed to rapidly turn from purple to brown on addition of DBU, also suggesting a loss of 'free' iodine in solution.

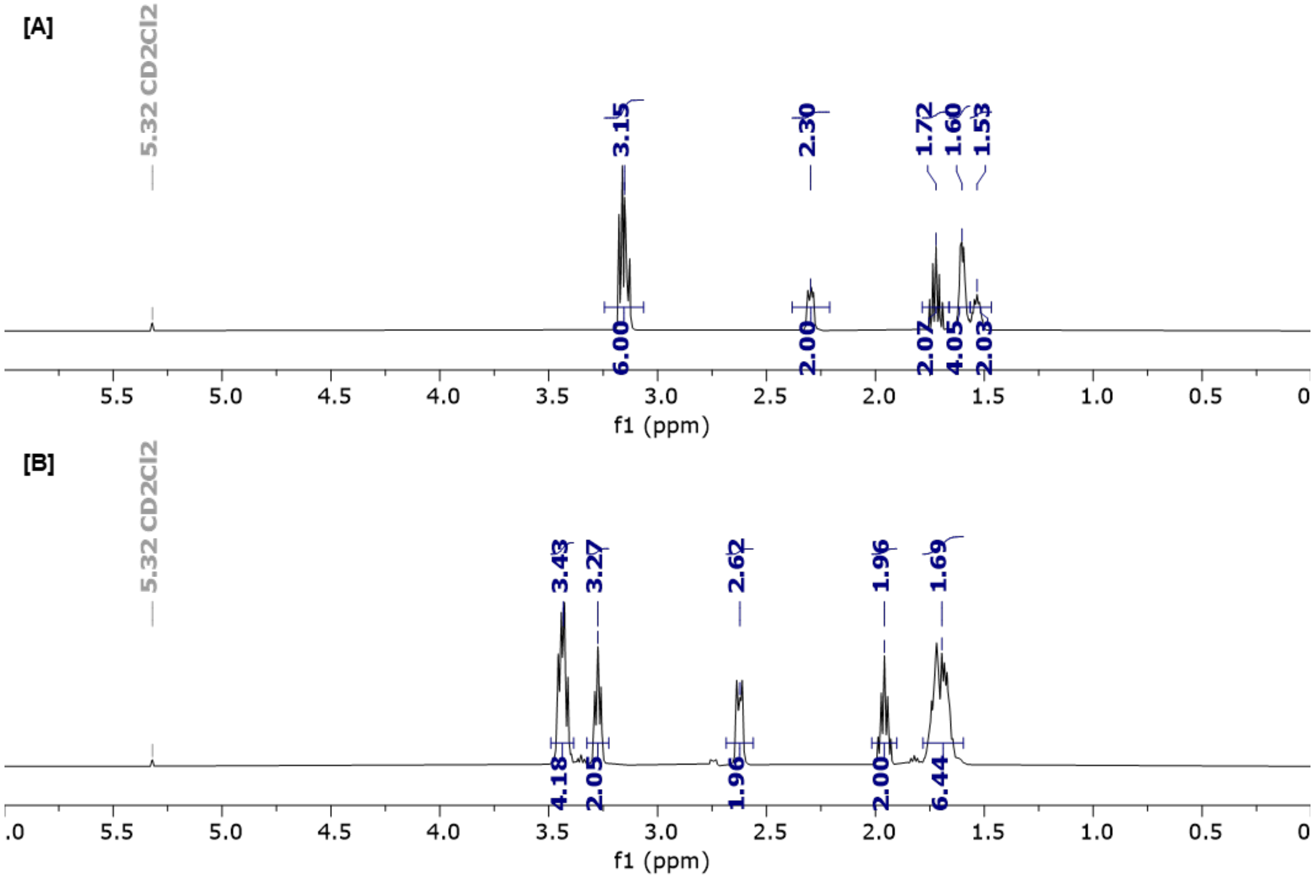

Figure S1. ^{1}H NMR spectra of **A)** DBU and **B)** a 4:3 mixture of DBU and iodine in CD_2Cl_2.

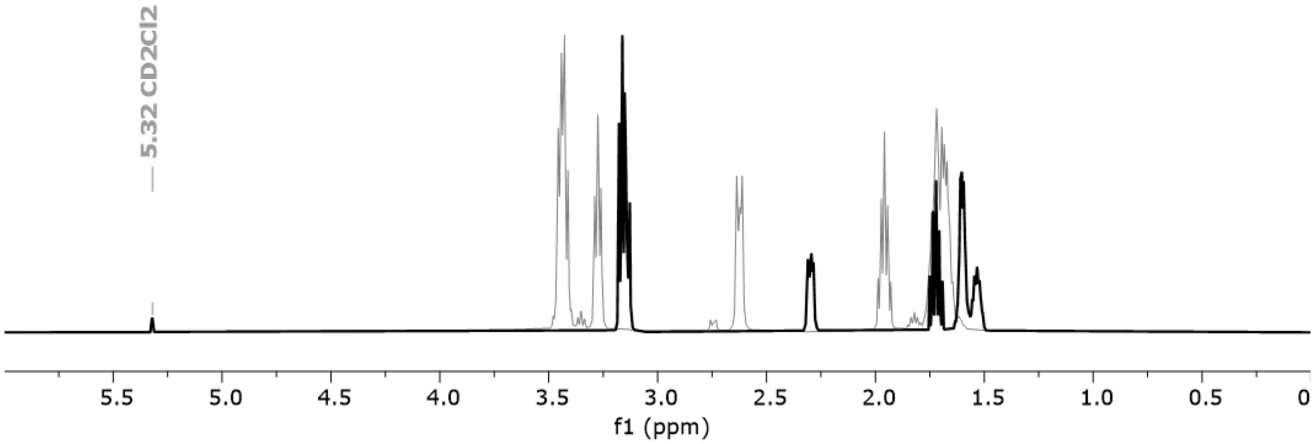

Figure S2. Superimposed ^{1}H NMR spectra of DBU (foreground, black) and a 4:3 mixture of DBU and iodine (background, grey) in CD_2Cl_2.

Other Reaction Component Interactions

Analysis of ^{1}H NMR spectra of other combinations of reaction components supports the formation of a DBU•I_2 adduct in solution, which is distinct from both DBU and DBU•H (Table S4). The interactions of (primary) 4-fluorobenzyl alcohol **F-2a** and (secondary) 1-(2-fluorophenyl)ethanol **4a** with a combination of DBU and iodine (i.e. DBU•I_2 in solution) are also distinct from their interactions with either with DBU, iodine or DBU•H alone (Tables S5-S6).

Table S4. ^{1}H NMR shifts of DBU signals (in ppm) in CD_2Cl_2 solutions of DBU and other reaction components.

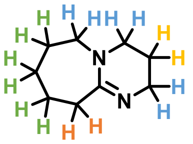

DBU						3.15 (6H)			2.30 (2H)			1.72 (2H)				1.60 (4H)	1.54 (2H)
DBU:I_2 (4:3)			3.43 (4H)		3.27 (2H)			2.62 (2H)			1.96 (2H)			1.71 (6H)			
DBU:**F-2a**(4:1)						3.15 (6H)			2.30 (2H)			1.72 (2H)				1.60 (4H)	1.54 (2H)
DBU:**4a** (4:1)						3.15 (6H)			2.29 (2H)			1.71 (2H)				1.59 (4H)	1.54 (2H)
DBU:I_2:**F-2a** (4:3:1)			3.44 (4H)		3.28 (2H)			2.64 (2H)			1.97 (2H)			1.70 (6H)			
DBU:I_2:**4a** (4:3:1)			3.45 (4H)		3.28 (2H)			2.65 (2H)			1.97 (2H)			1.71 (6H)			
DBU•H	3.54 (2H)	3.50 (2H)		3.41 (2H)			2.68 (2H)			2.05 (2H)			1.77 (4H)		1.70 (2H)		
DBU•H:I_2 (4:3)	3.54 (2H)	3.50 (2H)		3.41 (2H)			2.68 (2H)			2.05 (2H)			1.77 (4H)		1.71 (2H)		
DBU•H:**F-2a** (1:1)	3.53 (2H)	3.49 (2H)		3.39 (2H)			2.67 (2H)			2.04 (2H)			1.76 (4H)		1.70 (2H)		
DBU•H:**F-2a** (2:1)	3.53 (2H)	3.49 (2H)		3.38 (2H)			2.66 (2H)			2.03 (2H)			1.75 (4H)		1.70 (2H)		
DBU•H:**F-2a** (3:1)	3.53 (2H)	3.49 (2H)		3.37 (2H)			2.65 (2H)			2.03 (2H)			1.74 (4H)		1.68 (2H)		
DBU•H:**4a** (1:1)	3.53 (2H)	3.49 (2H)		3.40 (2H)			2.68 (2H)			2.04 (2H)			1.76 (4H)		1.70 (2H)		
DBU•H:**4a** (2:1)	3.54 (2H)	3.49 (2H)		3.38 (2H)			2.67 (2H)			2.04 (2H)			1.75 (4H)		1.70 (2H)		
DBU•H:**4a** (3:1)	3.54 (2H)	3.50 (2H)		3.37 (2H)			2.66 (2H)			2.03 (2H)			1.75 (4H)		1.70 (2H)		

Table S5. ^{1}H NMR shifts of (primary) 4-fluorobenzyl alcohol **F-2a** signals (in ppm) in CD_2Cl_2 solutions of **F-2a** and other reaction components.

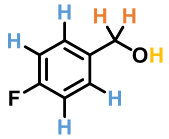

**F-2a**	7.34 (2H)		7.05 (2H)				4.63 (2H)						1.84 (1H)
**F-2a**:DBU (1:4)		7.31 (2H)			6.98 (2H)				4.53 (2H)		6.22 (1.2H)		
**F-2a**:I_2 (1:3)	7.34 (2H)		7.05 (2H)			4.69 (2H)						2.35 (1.3H)	
**F-2a**:DBU:I_2 (1:4:3)	7.33 (2H)			7.02 (2H)				4.60 (2H)					
**F-2a**:DBU•H (1:1)	7.34 (2H)		7.04 (2H)				4.63 (2H)			8.17 (0.6H)			
**F-2a**:DBU•H (1:2)	7.34 (2H)		7.03 (2H)				4.62 (2H)			8.11 (1.2H)			
**F-2a**:DBU•H (1:3)	7.34 (2H)		7.03 (2H)				4.61 (2H)			8.07 (1.8H)			

Table S6. ^{1}H NMR shifts of (secondary) 1-(2-fluorophenyl)ethanol **4a** signals (in ppm) in CD_2Cl_2 solutions of **4a** and other reaction components.

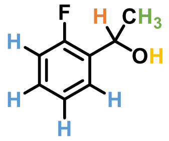

**4a**		7.49 (1H)	7.26 (1H)		7.16 (1H)			7.03 (1H)			5.17 (1H)					1.99 (1H)		1.48 (3H)		
**4a**:DBU (1:4)	7.58 (1H)				7.17 (1H)		7.11 (1H)			6.94 (1H)			5.08 (1H)		6.17 (1.2H)					1.38 (3H)
**4a**:I_2 (1:3)		7.49 (1H)	7.26 (1H)		7.16 (1H)			7.03 (1H)			5.17 (1H)						1.90 (1.4H)	1.49 (3H)		
**4a**:DBU:I_2 (1:4:3)		7.50 (1H)		7.23 (1H)		7.13 (1H)			6.99 (1H)			5.14 (1H)							1.44 (3H)	
**4a**:DBU•H (1:1)		7.50 (1H)	7.26 (1H)		7.16 (1H)			7.02 (1H)			5.16 (1H)			8.10 (0.3H)				1.48 (3H)		
**4a**:DBU•H (1:2)		7.50 (1H)	7.25 (1H)		7.15 (1H)			7.01 (1H)			5.16 (1H)			8.05 (0.9H)				1.47 (3H)		
**4a**:DBU•H (1:3)		7.49 (1H)	7.24 (1H)		7.15 (1H)			7.01 (1H)			5.15 (1H)			8.02 (1.6H)				1.46 (3H)		

NMR Sample Preparation

Solutions of one or more of DBU (0.400 mmol), iodine (0.300 mmol), 4-fluorobenzyl alcohol **F-2a** (0.100 mmol), 1-(2-fluorophenyl)ethanol **4a** (0.100 mmol) and DBU•HBF_4 (0.100-0.300 mmol) were prepared in CD_2Cl_2 (1 mL), according to the combinations shown in Tables S4-S6.

Synthesis of DBU•HBF$_4$ Salt

1,8-Diazabicyclo[5.4.0]undec-7-enium tetrafluoroborate

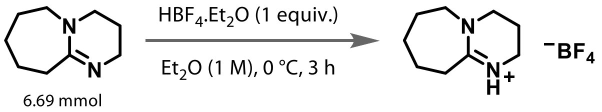

To a round-bottom flask was added DBU (1.00 mL, 6.69 mmol, 1 equiv.) and Et_2O (6.7 mL). The resulting solution was cooled to 0 °C (ice-water bath), then $HBF_4.Et_2O$ (0.911 mL, 6.69 mmol, 1 equiv.) was added dropwise. After 3 h, the precipitate formed was collected by filtration (Büchner, washed with cold Et_2O) and dried under vacuum overnight to obtain the title compound as an off-white solid (1.18 g, 74%).

^{1}H NMR (400 MHz, D$_2$O) δ 3.65 – 3.49 (m, 4H), 3.35 (t, *J* = 6.0 Hz, 2H), 2.69 – 2.62 (m, 2H), 2.05 (app quint, *J* = 6.0 Hz, 2H), 1.84 – 1.65 (m, 6H).

^{13}C NMR (101 MHz, D$_2$O) δ 166.0, 54.1, 48.2, 38.0, 32.8, 28.4, 25.8, 23.3, 18.9.

^{19}F NMR (376 MHz, D$_2$O) δ −150.42 (s, 0.8F), −150.47 (s, 3.2F).

Data are consistent with those previously reported.[31] NB: Only 8 of the 9 ^{13}C signals were reported, so, based on the otherwise corroborating data, it is believed that the signal at 25.8 ppm was accidentally omitted. As ^{19}F NMR data have not previously been reported, a copy of the spectrum is included.

Computational Study of Nucleophilic Attack

Method

The reactions between 2,2,2-triiodoacetophenone and two alkoxides (methoxide and isopropoxide) were investigated computationally. Geometries were optimised to the ground state, using DFT to determine the relevant stationary points for the reaction pathways considered. The hybrid metafunctional M06-2X[32] was employed for all ground state optimisations, along with the def2-QZVPPD Karlsruhe basis set, which features def2-ECPs for iodine atoms within the structures, two sets of polarisation functions and a set of diffuse functions.[33–36] Stationary points were characterised through their harmonic vibrational frequencies as either minima or saddle points. Ground states can be identified through a lack of imaginary vibrational frequencies. Transition states only feature a single imaginary vibrational frequency. Calculations were performed in Gaussian 16[37] and structures were visualised using GaussView 6.1.[38]

Solvent effects for DCM were modelled using the polarisable continuum model and the self-consistent reaction field approach, as implemented in Gaussian 16.[39] Thermal corrections to the Gibbs free energies were calculated from zero-point vibrational energies, assuming ideal gas behaviour for the enthalpy and entropy correction calculations.[37]

Results

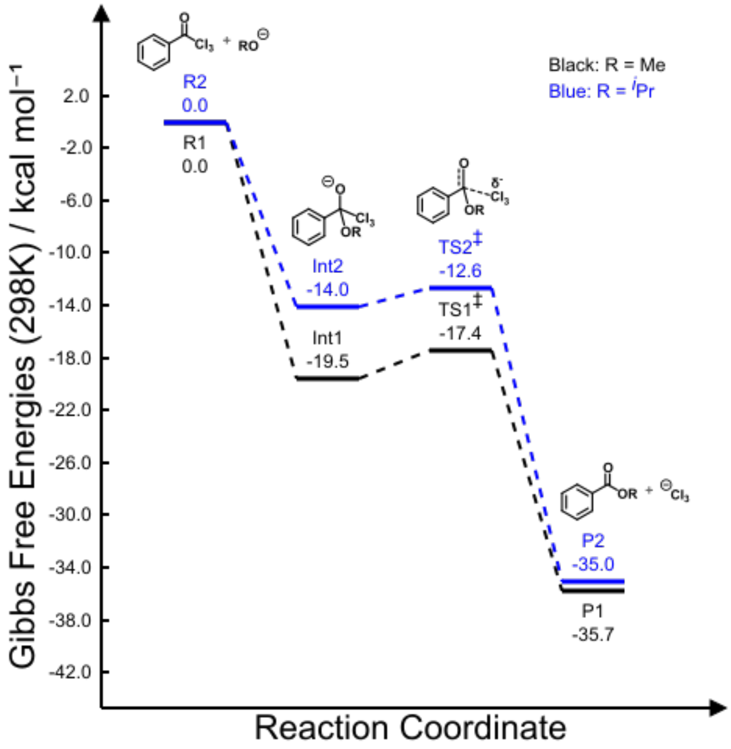

Scheme S1. Reaction pathways calculated for alkoxide-initiated C–CI_3 bond cleavage in 2,2,2-triiodoacetophenone. Blue: attack of isopropoxide anion; black: attack of methoxide anion. Gibbs free energies at 298 K are shown relative to the initial conditions pre-alkoxide attack on the ketone for each reaction pathway.

Table S7. Calculated ground state energies (E), zero-point vibrational energies (ZPVE), thermal corrections to enthalpy (ΔH_c), absolute entropies (ΔS) and thermally-corrected Gibbs energies (ΔG_c) for all species involved in the alkoxide-initiated C–CI_3 bond cleavage of 2,2,2 -triiodoacetophenone in DCM.

Species	E / Ha	ZPVE / (kcal mol^{-1})	ΔH_c (298 K) / Ha	ΔS (298 K) / (cal mol^{-1} K^{-1})	ΔG_c (298 K) / Ha
PhC(O)CI_3	−1275.979	67.6159	0.121	120.525	−1275.915
MeO^-	−115.208	23.286	0.041	54.933	−115.193
i-PrO^-	−193.840	59.218	0.100	69.004	−193.773
Int1	−1391.243	93.875	0.166	130.652	−1391.139
Int2	−1469.867	129.106	0.224	143.254	−1469.711
TS1	−1391.237	92.676	0.163	126.530	−1391.129
TS2	−1469.862	128.126	0.223	146.069	−1469.708
P1	−460.158	90.874	0.154	92.592	−460.047
P2	−538.790	126.198	0.213	105.835	−538.626
$^-CI_3$	−931.086	2.294	0.010	88.699	−931.117

Cartesian Coordinates of M06-2X/def2-QZVPPD-Optimised Geometries

2,2,2-Triiodoacetophenone

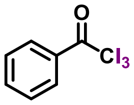

0 1

C	-2.13596800	0.72924300	-0.15688800
O	-0.95980300	0.66036000	-0.39038800
C	-2.96271300	-0.52120900	-0.07359200
C	-4.35578800	-0.57835200	-0.11070400
C	-2.23834600	-1.70971900	0.03522000
C	-5.00474200	-1.80104300	-0.04753500
H	-4.95071100	0.31547500	-0.19910000
C	-2.88939900	-2.92850900	0.11004300
H	-1.16059200	-1.66283300	0.06513300
C	-4.27595500	-2.97656200	0.06575500
H	-6.08347700	-1.83264200	-0.08626700
H	-2.31556600	-3.83884200	0.20170500
H	-4.78755600	-3.92668000	0.11964600
C	-2.75935100	2.13412300	0.06136100
I	-3.89448600	2.20526800	1.88578600
I	-3.96124000	2.58623000	-1.67278900
I	-1.20016700	3.60741600	0.19477300

Methoxide anion

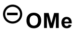

-1 1

C	-2.94523900	-0.21211400	0.06131900
H	-2.62053000	-1.27386400	-0.02098300
H	-2.62042000	0.24749100	-0.89942200
H	-4.05448100	-0.25977800	-0.02112300
O	-2.49081200	0.43034600	1.17414800

Isopropoxide anion

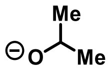


```
-1 1
 C                 -3.68743200   -0.28269200   -0.00702600
 C                 -2.14932300   -0.23796800    0.05580400
 H                 -1.82991800    0.23260200   -0.90583100
 H                 -4.08727400    0.73148200    0.00992500
 H                 -4.05853000   -0.78623700   -0.90392500
 H                 -4.07142600   -0.81067000    0.86975400
 C                 -1.61912500   -1.68243600   -0.00329200
 H                 -0.52853300   -1.67853300   -0.00652100
 H                 -1.95198700   -2.22419600    0.88604500
 H                 -1.96552700   -2.22304500   -0.88853000
 O                 -1.69030200    0.44247600    1.14046500
```

2,2,2-Triiodo-1-methoxy-1-phenylethanolate anion, Int1

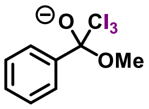


```
-1 1
 C                 -0.68088600   -1.82044800   -0.05140000
 O                 -0.17339100   -1.63628700   -1.21760800
 C                 -2.22937200   -1.83827300   -0.02748000
 C                 -2.90961400   -2.17234600   -1.19454200
 C                 -2.97083600   -1.46134100    1.08835100
 C                 -4.29645300   -2.16658500   -1.23915800
 H                 -2.32724100   -2.42180800   -2.06931600
 C                 -4.36006300   -1.44764600    1.04805200
 H                 -2.45304000   -1.16197700    1.98694600
 C                 -5.02813700   -1.80898200   -0.11347600
 H                 -4.80739100   -2.43313700   -2.15415200
 H                 -4.91966100   -1.14865800    1.92368000
 H                 -6.10872200   -1.79982800   -0.14570500
 C                 -0.10598900   -3.21987800    0.58815800
 I                 -0.66698300   -3.62575000    2.64773300
 I                  2.05624100   -3.11666400    0.50205000
 I                 -0.74050400   -4.89546800   -0.61253700
 O                 -0.24575400   -0.88962900    0.97998500
 C                 -0.29308600    0.44849500    0.55336200
 H                  0.07097300    1.06089000    1.37538800
 H                  0.33191700    0.60520700   -0.32512300
 H                 -1.31660900    0.75423400    0.31197000
```

2,2,2-Triiodo-1-isopropoxy-1-phenylethanolate anion, Int2

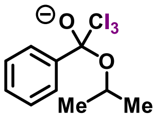

```
-1 1
 C                 -0.86962000   -1.77935500   -0.17565400
 O                 -0.46037300   -1.59518600   -1.37565700
 C                 -2.40658100   -1.90659400   -0.02700100
 C                 -3.15434200   -2.25031100   -1.14786800
 C                 -3.08003500   -1.62391100    1.15779600
 C                 -4.53878300   -2.33105300   -1.08745100
 H                 -2.62721700   -2.43311100   -2.07247000
 C                 -4.46538000   -1.70273300    1.22546800
 H                 -2.51508400   -1.31425000    2.02362200
 C                 -5.20049800   -2.06202700    0.10376000
 H                 -5.10247600   -2.59684700   -1.97125400
 H                 -4.97136800   -1.47374800    2.15338800
 H                 -6.27889400   -2.11995000    0.15434500
 C                 -0.17548200   -3.17681300    0.41922900
 I                 -0.55834900   -3.65013500    2.50940300
 I                  1.96857800   -2.96908400    0.18644000
 I                 -0.80472800   -4.87088300   -0.76787100
 O                 -0.40794200   -0.84722900    0.83282600
 C                 -0.28003500    0.52642400    0.47603100
 H                 -0.18596700    1.01898000    1.44711500
 C                  0.99406000    0.81197400   -0.30810900
 H                  1.84890400    0.35816400    0.19004600
 H                  0.92223900    0.40304300   -1.31154300
 H                  1.15605600    1.88936200   -0.36320200
 C                 -1.50979800    1.10303200   -0.21495800
 H                 -1.66659600    0.62291600   -1.17873400
 H                 -2.40253000    0.96721500    0.39233400
 H                 -1.36037800    2.17115600   -0.37308200
```

2,2,2-Triiodo-1-methoxy-1-phenylethanolate anion to methyl benzoate, TS1

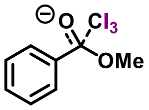

-1 1

C	1.10859200	-2.63122500	-0.40718600
C	1.22526000	-2.52583700	-1.79042400
C	2.24848800	-2.49949900	0.38024700
C	2.46576600	-2.31230400	-2.37597300
H	0.34140000	-2.60574400	-2.40376300
C	3.48873900	-2.28739500	-0.20394500
H	2.14367300	-2.55605200	1.45355700
C	3.60142500	-2.19740200	-1.58546500
H	2.54460100	-2.23127200	-3.45110200
H	4.36693800	-2.18688400	0.41839800
H	4.56666800	-2.02989700	-2.04235500
C	-0.30039800	-4.84759300	0.19269900
I	-0.17736100	-5.78571900	-1.77925900
I	-2.23916600	-5.38984000	1.04254600
I	1.23019300	-5.74556500	1.46399500
O	-0.35392700	-2.55625100	1.48055000
C	-0.22197000	-2.83898700	0.28183100
O	-1.26778600	-2.57790300	-0.60616800
C	-2.36864200	-1.87842700	-0.05393600
H	-2.06966600	-0.88163200	0.27157000
H	-3.10273700	-1.79660600	-0.85052600
H	-2.80195800	-2.40719100	0.79018200

2,2,2-Triiodo-1-methoxy-1-phenylethanolate anion to isopropyl benzoate, TS2

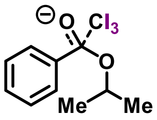

-1 1

C	-1.10061100	-1.40487800	-0.32981500
O	-0.63751300	-1.31908000	-1.46528300
C	-2.55646600	-1.73815300	-0.11933100
C	-3.28152300	-2.25723500	-1.18800100
C	-3.20391800	-1.50471700	1.09078000
C	-4.62864800	-2.55530900	-1.04683300
H	-2.77243800	-2.41518200	-2.12693300
C	-4.55260300	-1.80135300	1.23262000
H	-2.64560500	-1.08628600	1.91405700
C	-5.26760100	-2.33066500	0.16610100
H	-5.18171000	-2.95924300	-1.88327000
H	-5.04706600	-1.61560000	2.17582700
H	-6.31801700	-2.56042300	0.27716300
C	-0.32553400	-3.21365100	0.42835100
I	-0.77179200	-3.76027200	2.50321000
I	1.84159000	-2.99361600	0.25664100
I	-0.90169700	-4.89704700	-0.84561300
O	-0.58573400	-0.64346300	0.71240500
C	-0.21665400	0.71627900	0.42713400
H	-0.11688900	1.14294500	1.42580200
C	1.12513800	0.83387300	-0.27545400
H	1.88473200	0.27153800	0.26298000
H	1.06297600	0.44845400	-1.28927700
H	1.42338400	1.88225200	-0.30599500
C	-1.32288600	1.46487500	-0.30108500
H	-1.43631000	1.09486200	-1.31815900
H	-2.27158700	1.35238300	0.22181600
H	-1.07621800	2.52482600	-0.34443100

Methyl benzoate, P1

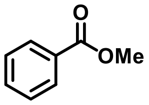

0 1

C	-1.60373800	0.28565600	-0.00004200
O	-0.39805500	0.27130300	-0.00015300
C	-2.45320300	-0.93731500	0.00006400
C	-3.84313000	-0.86545600	0.00019500
C	-1.81774700	-2.17512300	0.00003400
C	-4.59217800	-2.03201300	0.00029700
H	-4.33013700	0.09752400	0.00021700
C	-2.56919100	-3.33852100	0.00013700
H	-0.73827800	-2.20961500	-0.00006800
C	-3.95676000	-3.26681700	0.00026900
H	-5.67098500	-1.97825700	0.00040000
H	-2.07610900	-4.29952500	0.00011500
H	-4.54341100	-4.17428700	0.00035100
O	-2.31626800	1.41199400	0.00000300
C	-1.56152800	2.62479900	-0.00009100
H	-2.28886300	3.42725700	-0.00004000
H	-0.93533600	2.67724600	0.88636600
H	-0.93550000	2.67721100	-0.88666500

Isopropyl benzoate, P2

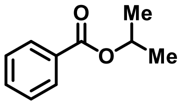

0 1

C	0.16240600	-0.90977600	-0.22021000
O	1.32023700	-0.88435200	0.11885400
C	-0.70362900	0.30243700	-0.28940100
C	-2.03370600	0.23313200	-0.69313800
C	-0.14503000	1.52701000	0.06302000
C	-2.79933700	1.38792700	-0.74350500
H	-2.46179100	-0.71934700	-0.96519700
C	-0.91253400	2.67891300	0.01179700
H	0.88866000	1.56045500	0.37400900
C	-2.24019100	2.60942100	-0.39180700
H	-3.83164900	1.33520800	-1.05709700
H	-0.47821900	3.62931000	0.28543800
H	-2.83927400	3.50784900	-0.43206900
O	-0.47874900	-2.01982300	-0.57885300
C	0.25847400	-3.26454900	-0.54075600
C	-0.46098800	-4.20613000	-1.48016400
C	0.30821500	-3.77779900	0.88435400
H	1.26449600	-3.05848900	-0.90061000
H	-0.49431100	-3.79872800	-2.48793200
H	0.05875800	-5.16153800	-1.50876000
H	-1.48007600	-4.37796200	-1.13640100
H	0.80040300	-3.06279300	1.53849300
H	-0.70138200	-3.96087300	1.25023300
H	0.86204700	-4.71418200	0.91710900

Triiodomethyl anion

$^{\ominus}CI_3$

```
-1 1
 C                 -1.77735900    0.05359400   -0.11970500
 I                 -1.10836600   -2.08088200    0.01838900
 I                 -1.10831500    1.00122700    1.79815300
 I                 -4.01247200   -0.02580100    0.01780600
```

COPASI Modelling

Manuscript Model

Reaction modelling was performed with COPASI.[40] The model presented in the manuscript was built using the elementary steps shown in Scheme S2. The initial concentrations of reaction species in the kinetics reactions were defined as shown in Tables S8-S9.

Model A: Model Presented in Manuscript

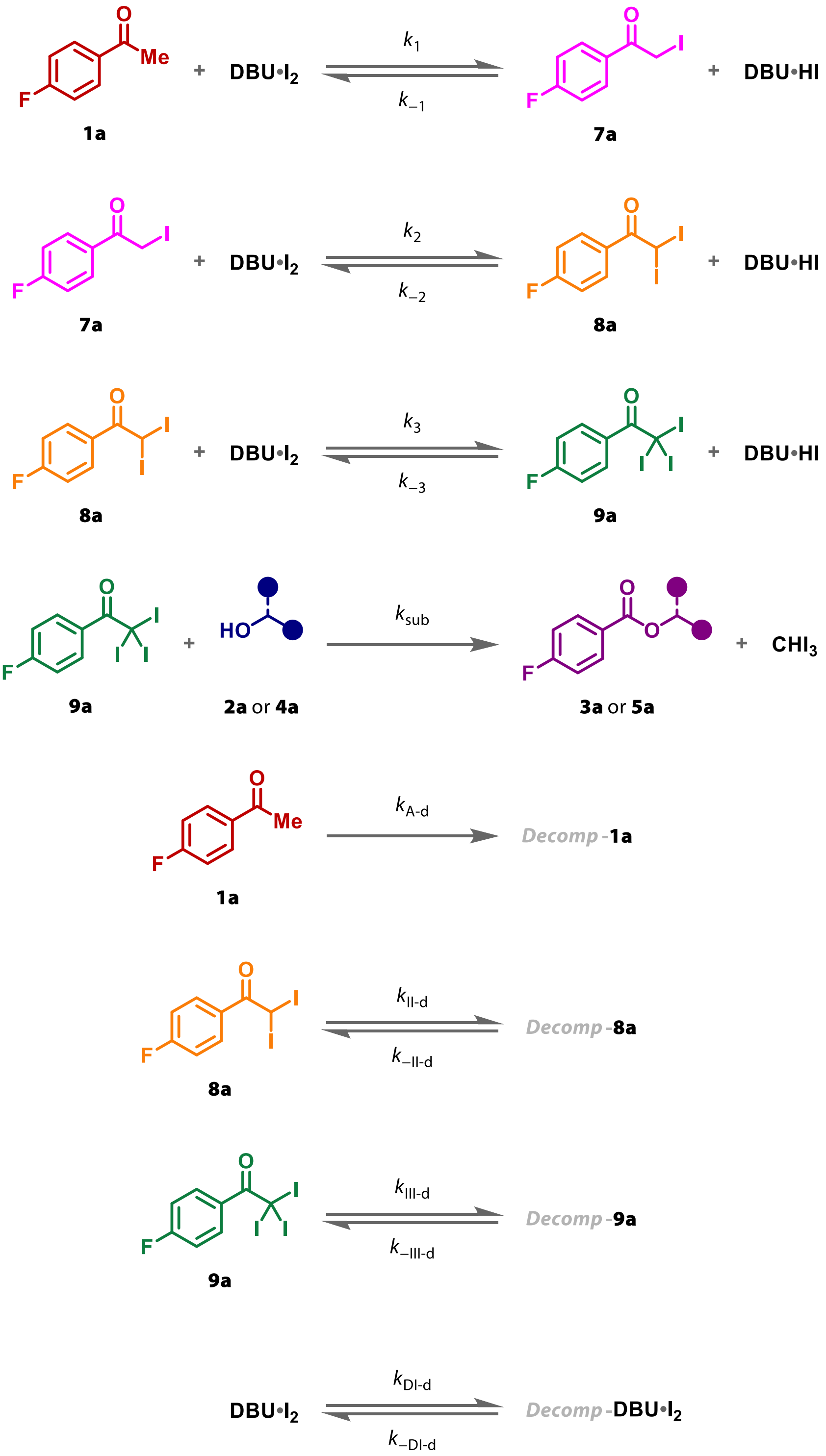

Scheme S2. Elementary steps used in COPASI to build the model of the haloform reaction presented in the manuscript. The 'decomp' terms were included to enable modelling of the side-reactions without needing to specify the identities of the decomposition products.

Table S8. Initial concentrations (in mmol mL^{-1}) set for reaction species in kinetics reactions not featuring DBU•HI.

Species	Without alcohol (primary alcohol conditions)	With primary alcohol **2a** (primary alcohol conditions)	With secondary alcohol **4a** (primary alcohol conditions)	With secondary alcohol **4a** (optimised secondary alcohol conditions)
**1a**	0.100	0.100	0.100	0.100
**7a**	0	0	0	0
**8a**	0	0	0	0
**9a**	0	0	0	0
**2a**/**4a**	–	0.105	0.105	0.105
**3a**/**5a**	–	0	0	0
DBU•I_2	0.303	0.303	0.303	0.480
DBU•HI	0	0	0	0
CHI_3	0	0	0	0
Decomp-**1a**	0	0	0	0
Decomp-**8a**	0	0	0	0
Decomp-**9a**	0	0	0	0
Decomp-DBU•I_2	0	0	0	0

Table S9. Initial concentrations (in mmol mL^{-1}) set for reaction species in kinetics reactions featuring DBU•HI.

Species	With primary alcohol **2a** and DBU•HI (1 equiv.)	With primary alcohol **2a** and DBU•HI (2 equiv.)	With secondary alcohol **4a** and DBU•HI (1 equiv.)	With secondary alcohol **4a** and DBU•HI (2 equiv.)
**1a**	0.100	0.100	0.100	0.100
**7a**	0	0	0	0
**8a**	0	0	0	0
**9a**	0	0	0	0
**2a**/**4a**	0.105	0.105	0.105	0.105
**3a**/**5a**	0	0	0	0
DBU•I_2	0.303	0.303	0.480	0.480
DBU•HI	0.100	0.200	0.100	0.200
CHI_3	0	0	0	0
Decomp-**1a**	0	0	0	0
Decomp-**8a**	0	0	0	0
Decomp-**9a**	0	0	0	0
Decomp-DBU•I_2	0	0	0	0

The rate constant of the first elementary step in Scheme S2, k_1, was determined by fitting the appropriate bimolecular rate law to the decay of 4'fluoroacetophenone **1a** in the alcohol-free reaction. The value of k_1 was determined from the first 4 min of the reaction (see Figure S3). The concentration of DBU•I_2 was calculated from the amount of **1a** remaining.

Subsequent rate constants were calculated using the 'parameter estimation' tool in COPASI, using 20,000 generations of population size 20. The concentrations of acetophenone **1a**, iodoacetophenone **7a**, diiodoacetophenone **8a** and triiodoacetophenone **9a** over 64 min in the alcohol-free reaction were imported and matched to their corresponding terms in the elementary reactions. Rate constants were then determined using the 'genetic algorithm' method with the default upper and lower bounds, and with k_1 held constant (Table S10). Rate constants for the substitution step, k_{sub}, with primary **2a** and secondary alcohol **4a** were, separately, calculated in the same manner, using experimental data from the reactions with these alcohols and using the rate constants determined for the alcohol-free reaction as starting points for the genetic algorithm (Table S10).

The fit of each reaction's model was assessed using the 'objective value' provided by COPASI, which is the weighted sum of square residuals (Table S12). COPASI also provided the root mean square of the fit, which is the square root of the sum of square residuals divided by the number of datapoints (Table S12).

Once all three reactions had been modelled, mean rate constants were calculated, which were used as the values of the rate constants in the model (Table S10). The coefficient of variation, which is the standard deviation divided by the mean, was also determined for each rate constant (Table S13). Coefficients of variation indicate the degree of variance between the values used to calculate each mean rate constant.

Sensitivities were calculated for rate constants using the 'time series' subtask in COPASI (Tables S14-15). The summarised sensitivities were calculated using a delta factor of 0.001 and a delta minimum of 1×10^{-12}. All variables of the model were affected by all parameter values in the analysis.

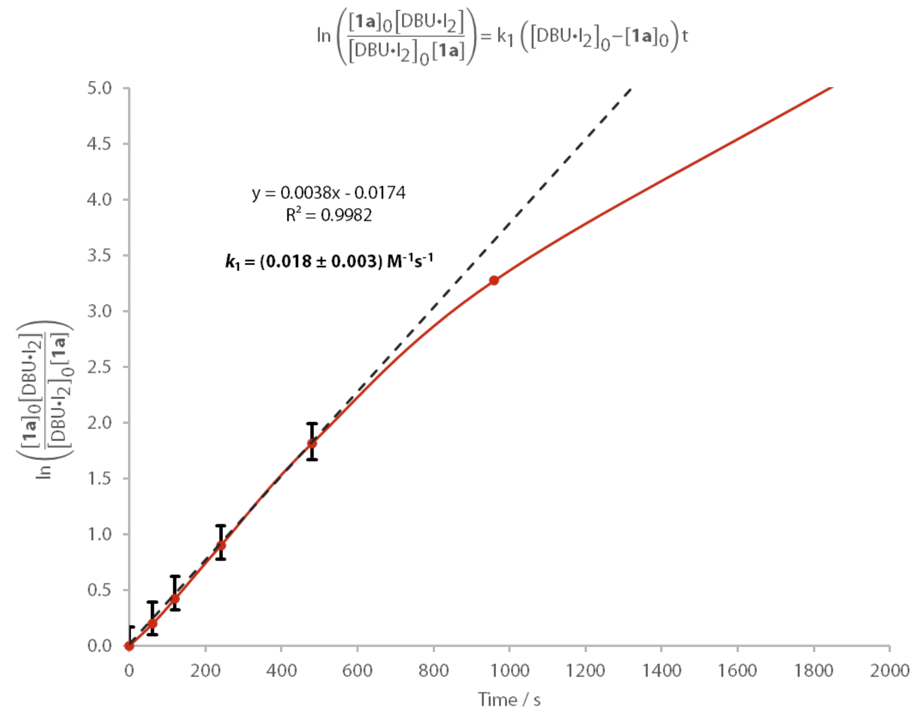

Figure S3. Experimental data fitted to the bimolecular rate law (red) to determine the rate constant for 4'-fluoroacetophenone **1a** decay, k_1. The gradient used to determine k_1 is shown by the dashed black line. The value of k_1 is shown with its associated uncertainty.

Table S10. Calculated rate constants (to 3 significant figures) for reactions which contributed to the mean rate constants used in model A. Mean values also shown.

Rate constant / units	Without alcohol (primary alcohol conditions)	With primary alcohol **2a** (primary alcohol conditions)	With secondary alcohol **4a** (primary alcohol conditions)	Mean
k_{-1} / mL mmol^{-1} s^{-1}	4.92E-03	5.00E-03	4.92E-03	4.94E-03
k_2 / mL mmol^{-1} s^{-1}	1.17E+00	1.20E+00	1.20E+00	1.19E+00
k_{-2} / mL mmol^{-1} s^{-1}	1.30E-02	1.35E-02	1.36E-02	1.34E-02
k_3 / mL mmol^{-1} s^{-1}	1.90E+00	1.97E+00	1.96E+00	1.94E+00
k_{-3} / mL mmol^{-1} s^{-1}	8.79E+00	8.89E+00	8.96E+00	8.88E+00
$k_{\text{sub 2a}}$ / mL mmol^{-1} s^{-1}	–	2.67E+05	–	2.67E+05
$k_{\text{sub 4a}}$ / mL mmol^{-1} s^{-1}	–	–	3.00E-02	3.00E-02
$k_{\text{A-d}}$ / s^{-1}	2.45E-04	2.50E-04	2.45E-04	2.46E-04
$k_{\text{II-d}}$ / s^{-1}	1.70E-05	2.00E-05	1.70E-05	1.80E-05
$k_{-\text{II-d}}$ / s^{-1}	1.00E-06	3.35E-06	1.00E-06	1.78E-06
$k_{\text{III-d}}$ / s^{-1}	2.04E+04	2.05E+04	2.04E+04	2.04E+04
$k_{-\text{III-d}}$ / s^{-1}	8.74E+04	8.74E+04	8.74E+04	8.74E+04
$k_{\text{DI-d}}$ / s^{-1}	1.02E+01	1.05E+01	1.02E+01	1.03E+01
$k_{-\text{DI-d}}$ / s^{-1}	2.66E+01	2.60E+01	2.66E+01	2.64E+01

Table S11. Calculated rate constants (to 3 significant figures) for reactions which did not contribute to the mean rate constants used in model A.

Rate constant / units	With secondary alcohol **4a** (optimised secondary alcohol conditions)	With primary alcohol **2a** and DBU•HI (1 equiv.)	With primary alcohol **2a** and DBU•HI (2 equiv.)	With secondary alcohol **4a** and DBU•HI (1 equiv.)	With secondary alcohol **4a** and DBU•HI (2 equiv.)
k_{-1} / mL mmol^{-1} s^{-1}	4.90E-03	4.98E-03	4.98E-03	4.87E-03	4.93E-03
k_2 / mL mmol^{-1} s^{-1}	1.19E+00	1.18E+00	1.18E+00	1.16E+00	1.18E+00
k_{-2} / mL mmol^{-1} s^{-1}	1.30E-02	1.35E-02	1.36E-02	1.32E-02	1.31E-02
k_3 / mL mmol^{-1} s^{-1}	2.11E+00	2.05E+00	2.02E+00	1.92E+00	1.98E+00
k_{-3} / mL mmol^{-1} s^{-1}	8.95E+00	8.95E+00	8.94E+00	9.01E+00	9.04E+00
$k_{\text{sub 2a}}$ / mL mmol^{-1} s^{-1}	–	2.67E+05	2.68E+05	–	–
$k_{\text{sub 4a}}$ / mL mmol^{-1} s^{-1}	2.90E-02	–	–	2.94E-02	3.04E-02
$k_{\text{A-d}}$ / s^{-1}	2.45E-04	2.47E-04	2.47E-04	2.53E-04	2.57E-04
$k_{\text{II-d}}$ / s^{-1}	1.33E-05	1.50E-05	1.34E-05	1.99E-05	1.69E-05
$k_{-\text{II-d}}$ / s^{-1}	1.00E-06	3.35E-06	3.69E-06	1.70E-06	2.19E-06
$k_{\text{III-d}}$ / s^{-1}	2.04E+04	2.05E+04	2.03E+04	2.04E+04	2.02E+04
$k_{-\text{III-d}}$ / s^{-1}	8.75E+04	8.74E+04	8.74E+04	8.86E+04	8.80E+04
$k_{\text{DI-d}}$ / s^{-1}	1.04E+01	1.08E+01	1.02E+01	1.10E+01	1.09E+01
$k_{-\text{DI-d}}$ / s^{-1}	2.65E+01	2.62E+01	2.66E+01	2.70E+01	2.62E+01

Table S12. Objective value, root mean square error and standard deviation (all to 3 significant figures) for each reaction.

Reaction	Objective value	Root mean square error	Standard deviation
Without alcohol (primary alcohol conditions)	1.20E-08	1.94E-05	1.97E-05
With primary alcohol **2a** (primary alcohol conditions)	1.44E-09	6.01E-06	6.09E-06
With secondary alcohol **4a** (primary alcohol conditions)	1.18E-08	1.57E-05	1.59E-05
With secondary alcohol **4a** (optimised secondary alcohol conditions)	1.78E-08	1.92E-05	1.94E-05
With primary alcohol **2a** and DBU•HI (1 equiv.)	1.15E-09	5.36E-06	5.43E-06
With primary alcohol **2a** and DBU•HI (2 equiv.)	1.12E-09	5.28E-06	5.35E-06
With secondary alcohol **4a** and DBU•HI (1 equiv.)	2.37E-08	2.22E-05	2.25E-05
With secondary alcohol **4a** and DBU•HI (2 equiv.)	1.24E-08	1.61E-05	1.63E-05

Table S13. Calculated mean, standard deviation and coefficient of variation (all to 3 significant figures) for rate constants used in model A.

Rate constant / units	Mean	Standard deviation	Coefficient of variation
k_1 / mL mmol^{-1} s^{-1}	1.81E-02	0.00E+00	0.00E+00
k_{-1} / mL mmol^{-1} s^{-1}	4.94E-03	3.99E-05	8.07E-03
k_2 / mL mmol^{-1} s^{-1}	1.19E+00	1.27E-02	1.07E-02
k_{-2} / mL mmol^{-1} s^{-1}	1.34E-02	2.50E-04	1.87E-02
k_3 / mL mmol^{-1} s^{-1}	1.94E+00	3.40E-02	1.75E-02
k_{-3} / mL mmol^{-1} s^{-1}	8.88E+00	7.08E-02	7.97E-03
$k_{\text{sub }\mathbf{2a}}$ / mL mmol^{-1} s^{-1}	2.67E+05	0.00E+00	0.00E+00
$k_{\text{sub }\mathbf{4a}}$ / mL mmol^{-1} s^{-1}	3.00E-02	0.00E+00	0.00E+00
$k_{\text{A-d}}$ / s^{-1}	2.46E-04	2.56E-06	1.04E-02
$k_{\text{II-d}}$ / s^{-1}	1.80E-05	1.39E-06	7.72E-02
$k_{-\text{II-d}}$ / s^{-1}	1.78E-06	1.11E-06	6.21E-01
$k_{\text{III-d}}$ / s^{-1}	2.04E+04	3.46E+01	1.69E-03
$k_{-\text{III-d}}$ / s^{-1}	8.74E+04	5.23E+00	5.98E-05
$k_{\text{DI-d}}$ / s^{-1}	1.03E+01	1.34E-01	1.30E-02
$k_{-\text{DI-d}}$ / s^{-1}	2.64E+01	2.94E-01	1.11E-02

Table S14. Calculated rate constant sensitivities (to 3 decimal places) for reactions which contributed to the mean rate constants used in model A.

Rate constant	Without alcohol (primary alcohol conditions)	With primary alcohol **2a** (primary alcohol conditions)	With secondary alcohol **4a** (primary alcohol conditions)
k_1	1.084	2.058	1.138
k_{-1}	0.870	0.196	0.791
k_2	1.211	1.058	1.121
k_{-2}	1.212	0.281	1.119
k_3	1.163	1.453	0.989
k_{-3}	1.163	0.175	0.987
$k_{\text{sub } \mathbf{2a}}$	–	1.028	–
$k_{\text{sub } \mathbf{4a}}$	–	–	1.342
$k_{\text{A-d}}$	1.126	3.113	1.242
$k_{\text{II-d}}$	0.981	1.035	0.983
$k_{-\text{II-d}}$	0.004	1.763	0.004
$k_{\text{III-d}}$	0.966	1.206	1.002
$k_{-\text{III-d}}$	0.965	0.828	1.001
$k_{\text{DI-d}}$	0.974	1.299	0.965
$k_{-\text{DI-d}}$	0.974	1.302	0.965

Table S15. Calculated rate constant sensitivities (to 3 decimal places) for reactions which did not contribute to the mean rate constants used in model A.

Rate constant	With secondary alcohol **4a** (optimised secondary alcohol conditions)	With primary alcohol **2a** and DBU•HI (1 equiv.)	With primary alcohol **2a** and DBU•HI (2 equiv.)	With secondary alcohol **4a** and DBU•HI (1 equiv.)	With secondary alcohol **4a** and DBU•HI (2 equiv.)
k_1	1.183	2.119	2.077	1.179	1.170
k_{-1}	0.978	0.068	0.089	0.964	0.949
k_2	1.361	1.881	1.036	1.335	1.307
k_{-2}	1.363	0.089	0.093	1.335	1.307
k_3	1.117	1.431	1.428	1.114	1.129
k_{-3}	1.116	0.108	0.001	1.114	1.128
$k_{\text{sub } \mathbf{2a}}$	–	1.429	1.404	–	–
$k_{\text{sub } \mathbf{4a}}$	1.520	–	–	1.419	1.353
$k_{\text{A-d}}$	1.038	3.780	3.751	1.007	0.988
$k_{\text{II-d}}$	0.985	1.000	1.000	0.984	0.984
$k_{-\text{II-d}}$	0.004	0.447	0.010	0.004	0.004
$k_{\text{III-d}}$	1.000	0.836	0.995	0.997	0.995
$k_{-\text{III-d}}$	0.999	1.015	0.999	0.995	0.995
$k_{\text{DI-d}}$	1.079	1.209	1.216	1.080	1.079
$k_{-\text{DI-d}}$	1.077	1.344	2.631	1.079	1.079

Other Models

To demonstrate the importance of including the side reactions in the model, ‘time courses’ were simulated in COPASI with elementary steps added or removed. The elementary steps included in each model are shown in Schemes S3-S7. By adding or removing side reactions one at a time, their individual impact on the accuracy of the model could be assessed by its objective value, root mean square error and standard deviation. The effects of these changes on the model fits can be seen in Figures S4-S11 and their impact on the objective value, root mean square error and standard deviation of each model is shown in Figures S12-S17.

Model B: Inclusion of Iodoacetophenone Decomposition

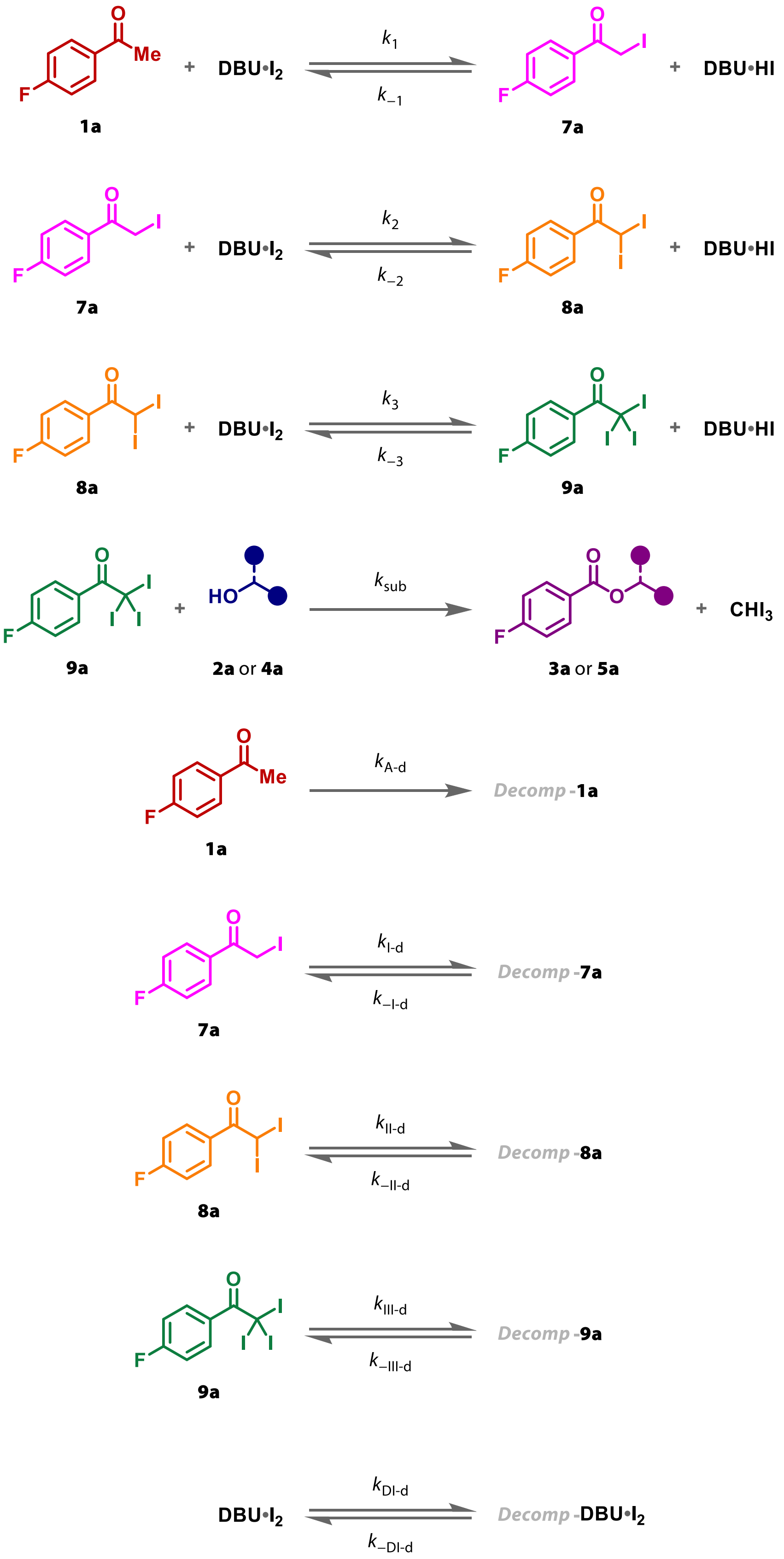

Scheme S3. Elementary steps used in COPASI to build a model of the haloform reaction which includes reversible iodoacetophenone **7a** decomposition. The 'decomp' terms were included to enable modelling of the side-reactions without needing to specify the identities of the decomposition products.

Table S16. Calculated rate constants (to 3 significant figures) for reactions which contributed to the mean rate constants used in model B. Mean values also shown.

Rate constant / units	Without alcohol (primary alcohol conditions)	With primary alcohol **2a** (primary alcohol conditions)	With secondary alcohol **4a** (primary alcohol conditions)	Mean
k_{-1} / mL mmol^{-1} s^{-1}	5.82E-03	5.82E-03	7.38E-03	6.34E-03
k_2 / mL mmol^{-1} s^{-1}	1.16E+00	1.17E+00	1.21E+00	1.18E+00
k_{-2} / mL mmol^{-1} s^{-1}	1.29E-02	1.19E-04	1.43E-02	9.12E-03
k_3 / mL mmol^{-1} s^{-1}	1.40E+00	1.93E+00	1.95E+00	1.76E+00
k_{-3} / mL mmol^{-1} s^{-1}	6.57E+00	3.32E-02	9.17E+00	5.26E+00
$k_{\mathrm{sub\ 2a}}$ / mL mmol^{-1} s^{-1}	–	1.32E+04	–	1.32E+04
$k_{\mathrm{sub\ 4a}}$ / mL mmol^{-1} s^{-1}	–	–	3.03E-02	3.03E-02
$k_{\mathrm{A\text{-}d}}$ / s^{-1}	2.27E-04	3.30E-04	2.85E-04	2.81E-04
$k_{\mathrm{I\text{-}d}}$ / s^{-1}	9.31E-02	2.69E-02	1.89E-06	4.00E-02
$k_{\mathrm{-I\text{-}d}}$ / s^{-1}	2.39E-01	1.51E-02	9.01E+00	3.09E+00
$k_{\mathrm{II\text{-}d}}$ / s^{-1}	1.69E-05	3.33E-06	1.30E-03	4.41E-04
$k_{\mathrm{-II\text{-}d}}$ / s^{-1}	1.82E-06	4.56E+05	1.93E+01	1.52E+05
$k_{\mathrm{III\text{-}d}}$ / s^{-1}	4.17E+04	1.22E-04	2.84E+01	1.39E+04
$k_{\mathrm{-III\text{-}d}}$ / s^{-1}	2.25E+05	2.51E+05	2.09E+02	1.59E+05
$k_{\mathrm{DI\text{-}d}}$ / s^{-1}	4.60E+00	1.76E+03	6.34E+02	8.00E+02
$k_{\mathrm{-DI\text{-}d}}$ / s^{-1}	1.24E+01	4.58E+03	1.51E+03	2.04E+03

Table S17. Calculated rate constants (to 3 significant figures) for reactions which did not contribute to the mean rate constants used in model B.

Rate constant / units	With secondary alcohol **4a** (optimised secondary alcohol conditions)	With primary alcohol **2a** and DBU•HI (1 equiv.)	With primary alcohol **2a** and DBU•HI (2 equiv.)	With secondary alcohol **4a** and DBU•HI (1 equiv.)	With secondary alcohol **4a** and DBU•HI (2 equiv.)
k_{-1} / mL mmol^{-1} s^{-1}	3.50E-03	7.30E-03	7.69E-03	4.23E-03	6.07E-03
k_2 / mL mmol^{-1} s^{-1}	1.20E+00	1.14E+00	1.14E+00	1.18E+00	1.21E+00
k_{-2} / mL mmol^{-1} s^{-1}	1.30E-02	3.41E-04	2.69E-04	1.37E-02	1.39E-02
k_3 / mL mmol^{-1} s^{-1}	1.06E+00	2.09E+00	2.11E+00	9.58E-01	1.27E+00
k_{-3} / mL mmol^{-1} s^{-1}	4.45E+00	3.30E-02	2.95E+01	4.50E+00	5.85E+00
$k_{\mathrm{sub\ 2a}}$ / mL mmol^{-1} s^{-1}	–	1.13E+04	7.80E+03	–	–
$k_{\mathrm{sub\ 4a}}$ / mL mmol^{-1} s^{-1}	2.91E-02	–	–	2.95E-02	3.03E-02
$k_{\mathrm{A\text{-}d}}$ / s^{-1}	3.18E-04	2.41E-04	2.43E-04	4.07E-04	4.14E-04
$k_{\mathrm{I\text{-}d}}$ / s^{-1}	1.15E-06	1.39E+00	1.86E-03	1.10E-05	2.52E-06
$k_{\mathrm{-I\text{-}d}}$ / s^{-1}	2.92E+01	9.82E+02	4.56E+02	2.10E+01	5.20E+02
$k_{\mathrm{II\text{-}d}}$ / s^{-1}	5.12E-05	4.00E-06	2.76E-03	1.60E-05	1.68E-05
$k_{\mathrm{-II\text{-}d}}$ / s^{-1}	1.82E+03	7.26E+05	8.79E+05	8.71E-04	1.50E+05
$k_{\mathrm{III\text{-}d}}$ / s^{-1}	1.31E+01	8.06E-05	4.27E-04	2.55E-06	1.61E-06
$k_{\mathrm{-III\text{-}d}}$ / s^{-1}	8.80E+01	1.55E+05	1.20E+05	1.09E+02	1.55E+05
$k_{\mathrm{DI\text{-}d}}$ / s^{-1}	3.26E+02	1.25E+03	1.27E+03	5.09E+02	4.80E+02
$k_{\mathrm{-DI\text{-}d}}$ / s^{-1}	7.37E+02	3.03E+03	3.17E+03	1.06E+03	1.03E+03

Table S18. Objective value, root mean square error and standard deviation (all to 3 significant figures) for each reaction.

Reaction	Objective value	Root mean square error	Standard deviation
Without alcohol (primary alcohol conditions)	4.87E-09	9.87E-10	8.88E-09
With primary alcohol **2a** (primary alcohol conditions)	1.23E-05	4.97E-06	1.36E-05
With secondary alcohol **4a** (primary alcohol conditions)	1.64E-05	6.28E-06	1.64E-05
With secondary alcohol **4a** (optimised secondary alcohol conditions)	4.87E-09	9.87E-10	8.88E-09
With primary alcohol **2a** and DBU•HI (1 equiv.)	1.23E-05	4.97E-06	1.36E-05
With primary alcohol **2a** and DBU•HI (2 equiv.)	1.64E-05	6.28E-06	1.64E-05
With secondary alcohol **4a** and DBU•HI (1 equiv.)	4.87E-09	9.87E-10	8.88E-09
With secondary alcohol **4a** and DBU•HI (2 equiv.)	1.23E-05	4.97E-06	1.36E-05

Table S19. Calculated mean, standard deviation and coefficient of variation (all to 3 significant figures) for rate constants used in model B.

Rate constant / units	Mean	Standard deviation	Coefficient of variation
k_1 / mL mmol^{-1} s^{-1}	1.81E-02	0.00E+00	0.00E+00
k_{-1} / mL mmol^{-1} s^{-1}	6.34E-03	7.35E-04	1.16E-01
k_2 / mL mmol^{-1} s^{-1}	1.18E+00	2.37E-02	2.01E-02
k_{-2} / mL mmol^{-1} s^{-1}	9.12E-03	6.39E-03	7.01E-01
k_3 / mL mmol^{-1} s^{-1}	1.76E+00	2.54E-01	1.44E-01
k_{-3} / mL mmol^{-1} s^{-1}	5.26E+00	3.84E+00	7.31E-01
$k_{\mathrm{sub}\ \mathbf{2a}}$ / mL mmol^{-1} s^{-1}	1.32E+04	0.00E+00	0.00E+00
$k_{\mathrm{sub}\ \mathbf{4a}}$ / mL mmol^{-1} s^{-1}	3.03E-02	0.00E+00	0.00E+00
$k_{\mathrm{A\text{-}d}}$ / s^{-1}	2.81E-04	4.21E-05	1.50E-01
$k_{\mathrm{I\text{-}d}}$ / s^{-1}	4.00E-02	3.91E-02	9.79E-01
$k_{-\mathrm{I\text{-}d}}$ / s^{-1}	3.09E+00	4.19E+00	1.36E+00
$k_{\mathrm{II\text{-}d}}$ / s^{-1}	4.41E-04	6.09E-04	1.38E+00
$k_{-\mathrm{II\text{-}d}}$ / s^{-1}	1.52E+05	2.15E+05	1.41E+00
$k_{\mathrm{III\text{-}d}}$ / s^{-1}	1.39E+04	1.97E+04	1.41E+00
$k_{-\mathrm{III\text{-}d}}$ / s^{-1}	1.59E+05	1.13E+05	7.09E-01
$k_{\mathrm{DI\text{-}d}}$ / s^{-1}	8.00E+02	7.26E+02	9.08E-01
$k_{-\mathrm{DI\text{-}d}}$ / s^{-1}	2.04E+03	1.90E+03	9.35E-01

Model C: Exclusion of Triiodoacetophenone Decomposition

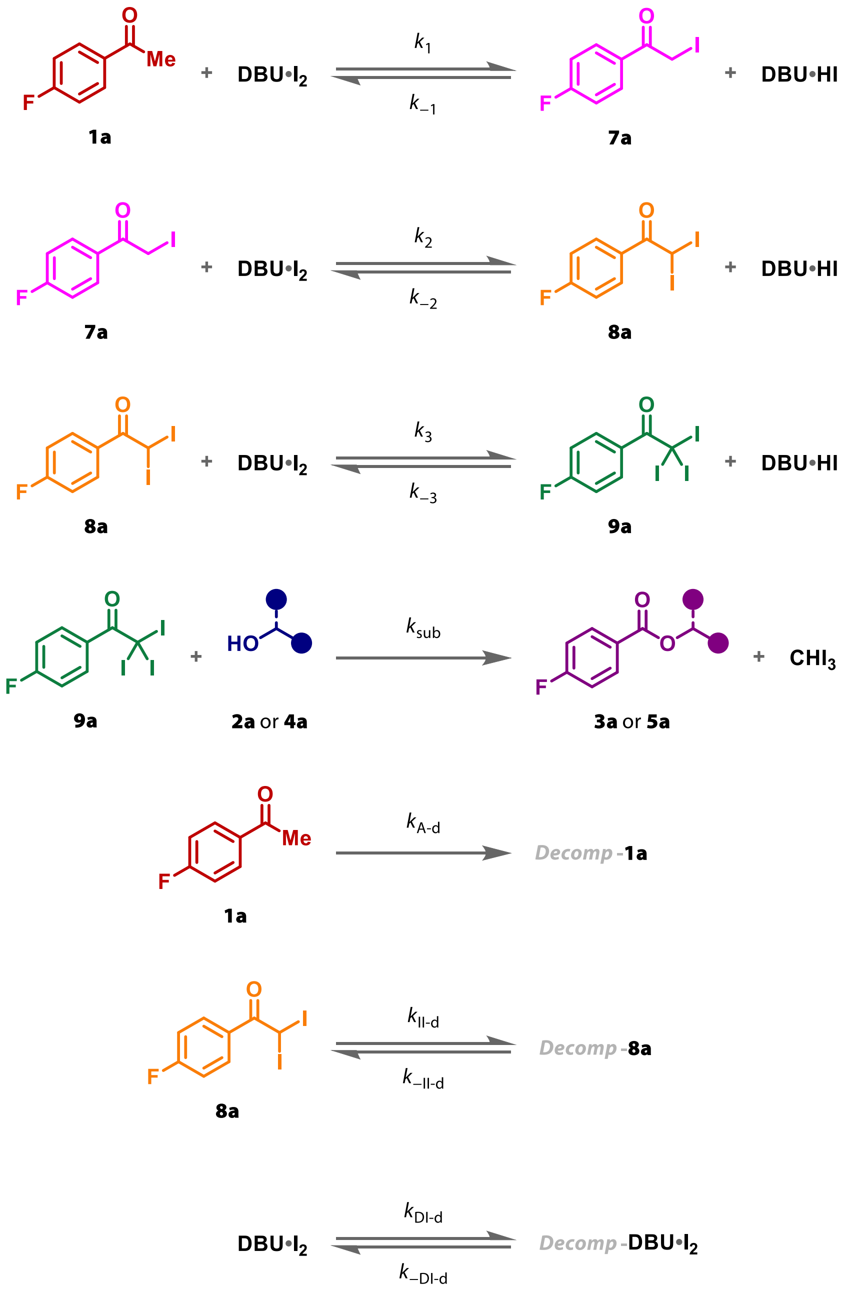

Scheme S4. Elementary steps used in COPASI to build a model of the haloform reaction which excludes triiodoacetophenone **9a** decomposition. The ‘decomp’ terms were included to enable modelling of the side-reactions without needing to specify the identities of the decomposition products.

Table S20. Calculated rate constants (to 3 significant figures) for reactions which contributed to the mean rate constants used in model C. Mean values also shown.

Rate constant / units	Without alcohol (primary alcohol conditions)	With primary alcohol **2a** (primary alcohol conditions)	With secondary alcohol **4a** (primary alcohol conditions)	Mean
k_{-1} / mL mmol^{-1} s^{-1}	3.86E-03	1.20E-05	7.07E-03	3.65E-03
k_2 / mL mmol^{-1} s^{-1}	1.18E+00	1.22E+00	1.25E+00	1.22E+00
k_{-2} / mL mmol^{-1} s^{-1}	1.37E-02	2.54E-04	1.48E-02	9.57E-03
k_3 / mL mmol^{-1} s^{-1}	6.58E-01	2.02E+00	1.30E+00	1.33E+00
k_{-3} / mL mmol^{-1} s^{-1}	3.15E+00	3.38E-02	6.13E+00	3.10E+00
$k_{\mathrm{sub}\ \mathbf{2a}}$ / mL mmol^{-1} s^{-1}	–	2.13E+04	–	2.13E+04
$k_{\mathrm{sub}\ \mathbf{4a}}$ / mL mmol^{-1} s^{-1}	–	–	3.03E-02	3.03E-02
$k_{\mathrm{A\text{-}d}}$ / s^{-1}	3.48E-04	3.15E-04	3.06E-04	3.23E-04
$k_{\mathrm{II\text{-}d}}$ / s^{-1}	9.34E-06	3.71E-06	9.49E-04	3.21E-04
$k_{-\mathrm{II\text{-}d}}$ / s^{-1}	4.75E-06	3.59E+05	2.19E+02	1.20E+05
$k_{\mathrm{DI\text{-}d}}$ / s^{-1}	6.06E+00	2.14E+03	3.69E+02	8.39E+02
$k_{-\mathrm{DI\text{-}d}}$ / s^{-1}	1.26E+01	4.73E+03	7.59E+02	1.83E+03

Table S21. Calculated rate constants (to 3 significant figures) for reactions which did not contribute to the mean rate constants used in model C.

Rate constant / units	With secondary alcohol **4a** (optimised secondary alcohol conditions)	With primary alcohol **2a** and DBU•HI (1 equiv.)	With primary alcohol **2a** and DBU•HI (2 equiv.)	With secondary alcohol **4a** and DBU•HI (1 equiv.)	With secondary alcohol **4a** and DBU•HI (2 equiv.)
k_{-1} / mL mmol^{-1} s^{-1}	1.18E-03	4.66E-03	7.69E-03	4.23E-03	6.07E-03
k_2 / mL mmol^{-1} s^{-1}	1.20E+00	1.15E+00	1.14E+00	1.18E+00	1.21E+00
k_{-2} / mL mmol^{-1} s^{-1}	1.31E-02	4.43E-04	2.69E-04	1.37E-02	1.39E-02
k_3 / mL mmol^{-1} s^{-1}	1.13E+00	2.09E+00	2.11E+00	9.58E-01	1.27E+00
k_{-3} / mL mmol^{-1} s^{-1}	4.87E+00	1.94E+00	2.95E+01	4.50E+00	5.85E+00
$k_{\mathrm{sub}\ \mathbf{2a}}$ / mL mmol^{-1} s^{-1}	–	9.78E+03	7.80E+03	–	–
$k_{\mathrm{sub}\ \mathbf{4a}}$ / mL mmol^{-1} s^{-1}	2.92E-02	–	–	2.95E-02	3.03E-02
$k_{\mathrm{A\text{-}d}}$ / s^{-1}	4.06E-04	2.42E-04	2.43E-04	4.07E-04	4.14E-04
$k_{\mathrm{II\text{-}d}}$ / s^{-1}	6.10E-05	5.99E-06	2.76E-03	1.60E-05	1.68E-05
$k_{-\mathrm{II\text{-}d}}$ / s^{-1}	1.78E+03	4.89E+05	8.79E+05	8.71E-04	1.50E+05
$k_{\mathrm{DI\text{-}d}}$ / s^{-1}	2.97E+02	1.25E+03	1.27E+03	5.09E+02	4.80E+02
$k_{-\mathrm{DI\text{-}d}}$ / s^{-1}	6.05E+02	2.98E+03	3.17E+03	1.06E+03	1.03E+03

Table S22. Objective value, root mean square error and standard deviation (all to 3 significant figures) for each reaction.

Reaction	Objective value	Root mean square error	Standard deviation
Without alcohol (primary alcohol conditions)	8.07E-09	1.14E-09	1.29E-08
With primary alcohol **2a** (primary alcohol conditions)	1.59E-05	5.35E-06	1.64E-05
With secondary alcohol **4a** (primary alcohol conditions)	1.92E-05	6.28E-06	1.87E-05
With secondary alcohol **4a** (optimised secondary alcohol conditions)	8.07E-09	1.14E-09	1.29E-08
With primary alcohol **2a** and DBU•HI (1 equiv.)	1.59E-05	5.35E-06	1.64E-05
With primary alcohol **2a** and DBU•HI (2 equiv.)	1.92E-05	6.28E-06	1.87E-05
With secondary alcohol **4a** and DBU•HI (1 equiv.)	8.07E-09	1.14E-09	1.29E-08
With secondary alcohol **4a** and DBU•HI (2 equiv.)	1.59E-05	5.35E-06	1.64E-05

Table S23. Calculated mean, standard deviation and coefficient of variation (all to 3 significant figures) for rate constants used in model C.

Rate constant / units	Mean	Standard deviation	Coefficient of variation
k_1 / mL mmol^{-1} s^{-1}	1.81E-02	0.00E+00	0.00E+00
k_{-1} / mL mmol^{-1} s^{-1}	3.65E-03	2.89E-03	7.91E-01
k_2 / mL mmol^{-1} s^{-1}	1.22E+00	2.71E-02	2.23E-02
k_{-2} / mL mmol^{-1} s^{-1}	9.57E-03	6.61E-03	6.90E-01
k_3 / mL mmol^{-1} s^{-1}	1.33E+00	5.58E-01	4.20E-01
k_{-3} / mL mmol^{-1} s^{-1}	3.10E+00	2.49E+00	8.02E-01
$k_{\mathrm{sub}\ \mathbf{2a}}$ / mL mmol^{-1} s^{-1}	2.13E+04	0.00E+00	0.00E+00
$k_{\mathrm{sub}\ \mathbf{4a}}$ / mL mmol^{-1} s^{-1}	3.03E-02	0.00E+00	0.00E+00
$k_{\mathrm{A\text{-}d}}$ / s^{-1}	3.23E-04	1.81E-05	5.59E-02
$k_{\mathrm{II\text{-}d}}$ / s^{-1}	3.21E-04	4.44E-04	1.39E+00
$k_{-\mathrm{II\text{-}d}}$ / s^{-1}	1.20E+05	1.69E+05	1.41E+00
$k_{\mathrm{DI\text{-}d}}$ / s^{-1}	8.39E+02	9.33E+02	1.11E+00
$k_{-\mathrm{DI\text{-}d}}$ / s^{-1}	1.83E+03	2.07E+03	1.13E+00

Model D: Exclusion of Di- and Triiodoacetophenone Decomposition

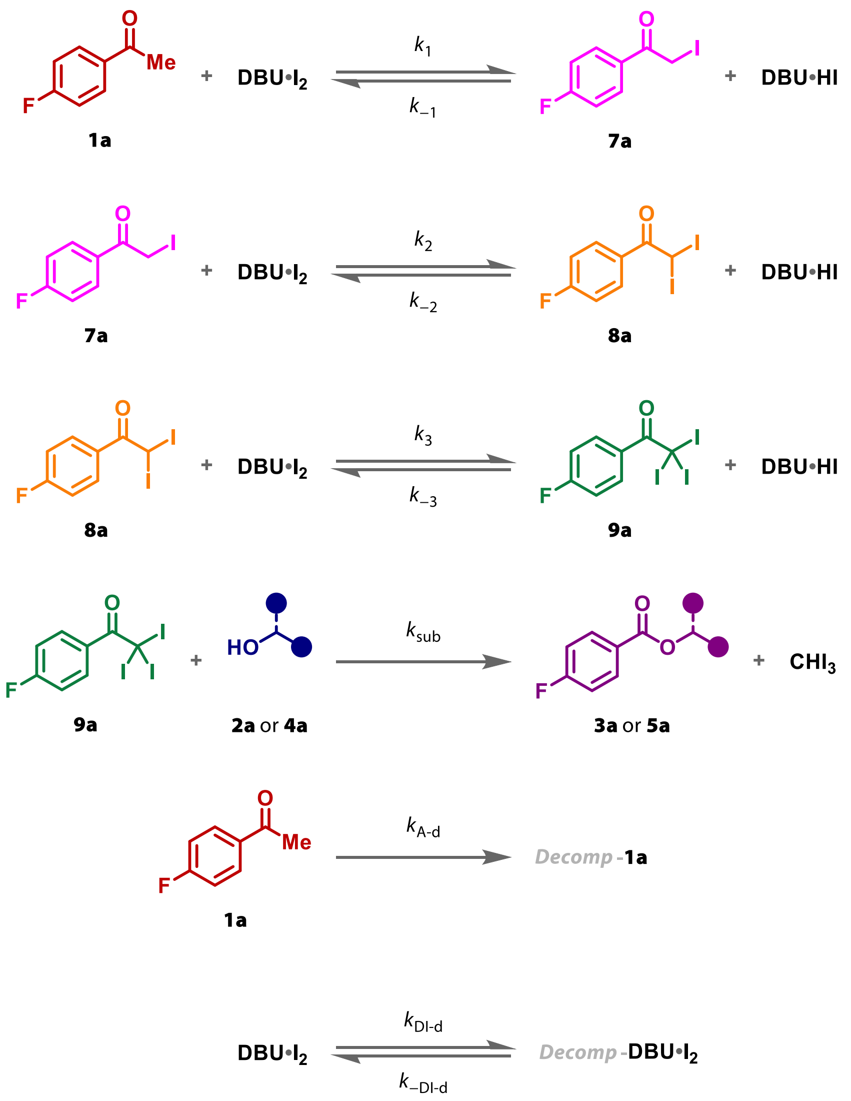

Scheme S5. Elementary steps used in COPASI to build a model of the haloform reaction which excludes di- **8a** and triiodoacetophenone **9a** decomposition. The 'decomp' terms were included to enable modelling of the side-reactions without needing to specify the identities of the decomposition products.

Table S24. Calculated rate constants (to 3 significant figures) for reactions which contributed to the mean rate constants used in model D. Mean values also shown.

Rate constant / units	Without alcohol (primary alcohol conditions)	With primary alcohol **2a** (primary alcohol conditions)	With secondary alcohol **4a** (primary alcohol conditions)	Mean
k_{-1} / mL mmol^{-1} s^{-1}	7.34E-03	7.81E-06	5.99E-03	4.44E-03
k_2 / mL mmol^{-1} s^{-1}	1.18E+00	1.22E+00	1.22E+00	1.21E+00
k_{-2} / mL mmol^{-1} s^{-1}	1.40E-02	3.27E-04	1.48E-02	9.70E-03
k_3 / mL mmol^{-1} s^{-1}	6.10E-01	2.03E+00	9.98E-01	1.21E+00
k_{-3} / mL mmol^{-1} s^{-1}	2.97E+00	2.19E-02	4.67E+00	2.56E+00
$k_{\text{sub }\mathbf{2a}}$ / mL mmol^{-1} s^{-1}	–	2.17E+04	–	2.17E+04
$k_{\text{sub }\mathbf{4a}}$ / mL mmol^{-1} s^{-1}	–	–	3.03E-02	3.03E-02
$k_{\text{A-d}}$ / s^{-1}	3.59E-04	3.12E-04	3.24E-04	3.32E-04
$k_{\text{DI-d}}$ / s^{-1}	7.50E+00	2.61E+03	3.30E+02	9.82E+02
$k_{-\text{DI-d}}$ / s^{-1}	1.53E+01	5.74E+03	6.99E+02	2.15E+03

Table S25. Calculated rate constants (to 3 significant figures) for reactions which did not contribute to the mean rate constants used in model D.

Rate constant / units	With secondary alcohol **4a** (optimised secondary alcohol conditions)	With primary alcohol **2a** and DBU•HI (1 equiv.)	With primary alcohol **2a** and DBU•HI (2 equiv.)	With secondary alcohol **4a** and DBU•HI (1 equiv.)	With secondary alcohol **4a** and DBU•HI (2 equiv.)
k_{-1} / mL mmol^{-1} s^{-1}	1.18E-03	7.84E-03	7.69E-03	4.44E-03	5.58E-03
k_2 / mL mmol^{-1} s^{-1}	1.20E+00	1.15E+00	1.14E+00	1.19E+00	1.20E+00
k_{-2} / mL mmol^{-1} s^{-1}	1.31E-02	5.41E-04	2.69E-04	1.35E-02	1.36E-02
k_3 / mL mmol^{-1} s^{-1}	1.13E+00	2.09E+00	2.11E+00	8.84E-01	1.87E+00
k_{-3} / mL mmol^{-1} s^{-1}	4.87E+00	1.79E+00	2.95E+01	4.13E+00	8.52E+00
$k_{\text{sub }\mathbf{2a}}$ / mL mmol^{-1} s^{-1}	–	1.13E+04	7.80E+03	–	–
$k_{\text{sub }\mathbf{4a}}$ / mL mmol^{-1} s^{-1}	2.92E-02	–	–	2.96E-02	3.07E-02
$k_{\text{A-d}}$ / s^{-1}	4.06E-04	2.39E-04	2.43E-04	4.06E-04	3.95E-04
$k_{\text{DI-d}}$ / s^{-1}	2.97E+02	1.03E+03	1.27E+03	7.60E+02	5.08E+02
$k_{-\text{DI-d}}$ / s^{-1}	6.05E+02	2.50E+03	3.17E+03	1.56E+03	1.08E+03

Table S26. Objective value, root mean square error and standard deviation (all to 3 significant figures) for each reaction.

Reaction	Objective value	Root mean square error	Standard deviation
Without alcohol (primary alcohol conditions)	1.25E-08	1.14E-09	1.21E-08
With primary alcohol **2a** (primary alcohol conditions)	1.97E-05	5.35E-06	1.59E-05
With secondary alcohol **4a** (primary alcohol conditions)	2.28E-05	6.07E-06	1.76E-05
With secondary alcohol **4a** (optimised secondary alcohol conditions)	1.25E-08	1.14E-09	1.21E-08
With primary alcohol **2a** and DBU•HI (1 equiv.)	1.97E-05	5.35E-06	1.59E-05
With primary alcohol **2a** and DBU•HI (2 equiv.)	2.28E-05	6.07E-06	1.76E-05
With secondary alcohol **4a** and DBU•HI (1 equiv.)	1.25E-08	1.14E-09	1.21E-08
With secondary alcohol **4a** and DBU•HI (2 equiv.)	1.97E-05	5.35E-06	1.59E-05

Table S27. Calculated mean, standard deviation and coefficient of variation (all to 3 significant figures) for rate constants used in model D.

Rate constant / units	Mean	Standard deviation	Coefficient of variation
k_1 / mL mmol^{-1} s^{-1}	1.81E-02	0.00E+00	0.00E+00
k_{-1} / mL mmol^{-1} s^{-1}	4.44E-03	3.18E-03	7.17E-01
k_2 / mL mmol^{-1} s^{-1}	1.21E+00	1.78E-02	1.48E-02
k_{-2} / mL mmol^{-1} s^{-1}	9.70E-03	6.63E-03	6.84E-01
k_3 / mL mmol^{-1} s^{-1}	1.21E+00	5.98E-01	4.94E-01
k_{-3} / mL mmol^{-1} s^{-1}	2.56E+00	1.92E+00	7.52E-01
$k_{\text{sub }\mathbf{2a}}$ / mL mmol^{-1} s^{-1}	2.17E+04	0.00E+00	0.00E+00
$k_{\text{sub }\mathbf{4a}}$ / mL mmol^{-1} s^{-1}	3.03E-02	0.00E+00	0.00E+00
$k_{\text{A-d}}$ / s^{-1}	3.32E-04	1.98E-05	5.96E-02
$k_{\text{DI-d}}$ / s^{-1}	9.82E+02	1.16E+03	1.18E+00
$k_{-\text{DI-d}}$ / s^{-1}	2.15E+03	2.55E+03	1.19E+00

Model E: Exclusion of Diiodo-, Triiodo- and Acetophenone Decomposition

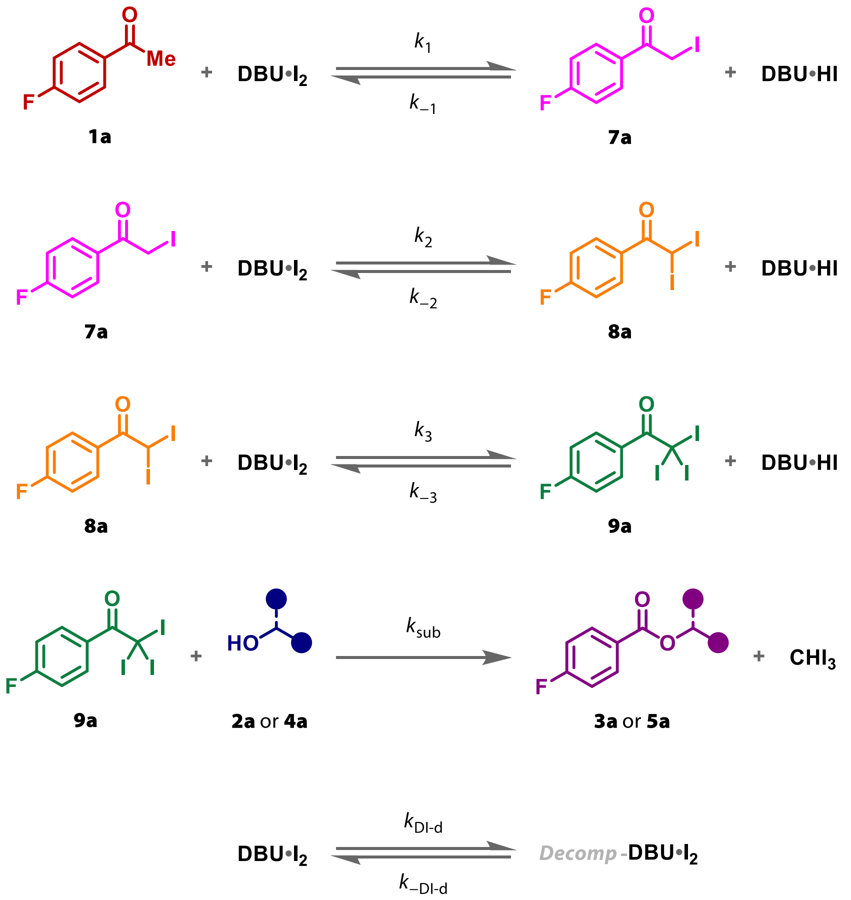

Scheme S6. Elementary steps used in COPASI to build a model of the haloform reaction which excludes diiodo- **8a**, triiodo- **9a** and acetophenone **1a** decomposition. The 'decomp' terms were included to enable modelling of the side-reactions without needing to specify the identities of the decomposition products.

Model F: Exclusion of All Side-Reactions

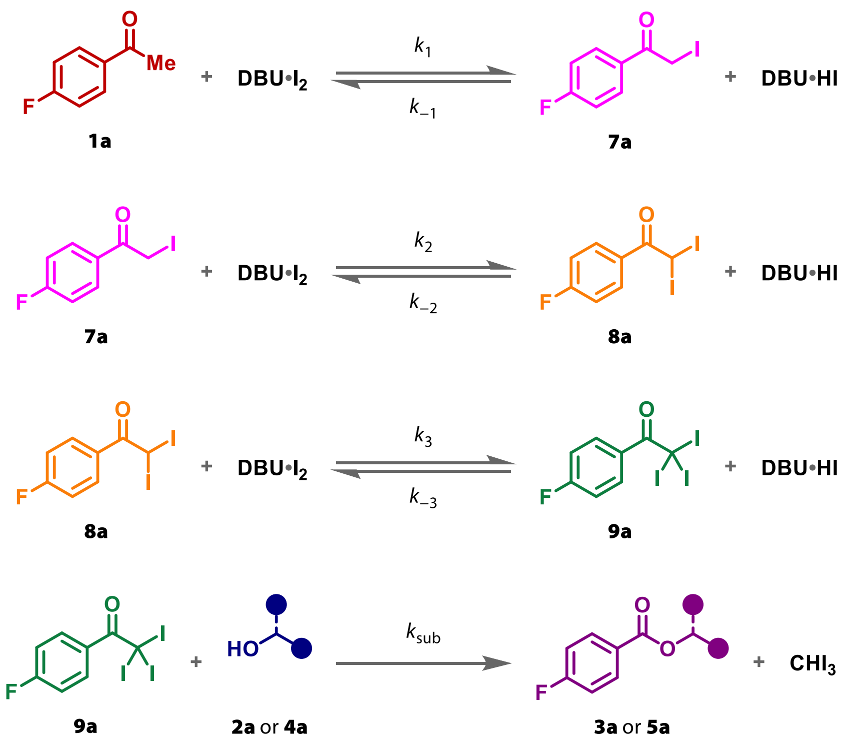

Scheme S7. Elementary steps used in COPASI to build a model of the haloform reaction which excludes diiodo- **8a**, triiodo- **9a** and acetophenone **1a**, and DBU•I_2 decomposition. The 'decomp' terms were included to enable modelling of the side-reactions without needing to specify the identities of the decomposition products.

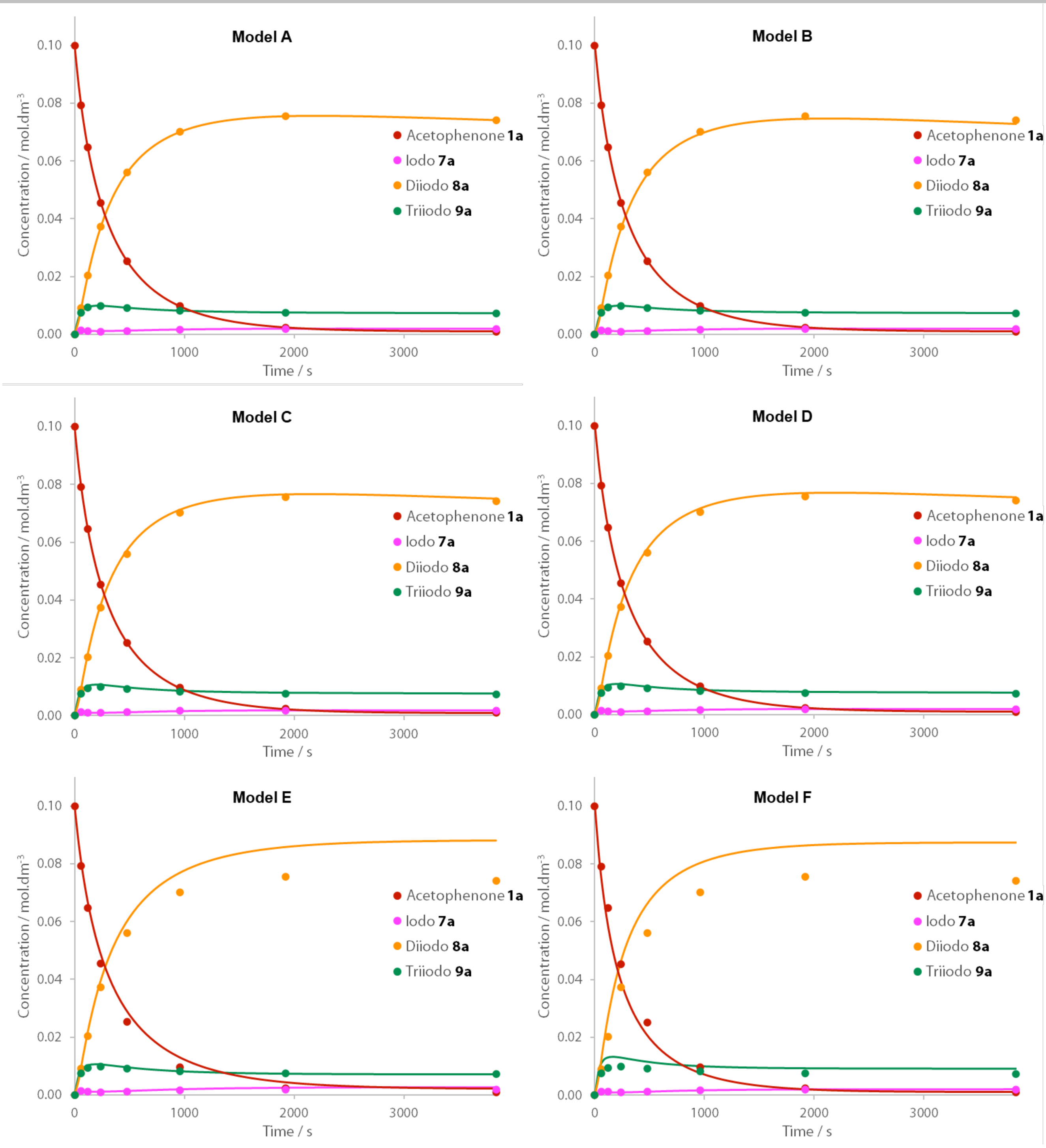

Figure S4. Time course plots for the kinetic reaction without alcohol using different COPASI models. Experimental concentrations shown as points; simulated data shown as solid lines. **Model A:** model presented in the manuscript (Scheme S2). **Model B:** including iodoacetophenone **7a** decomposition (Scheme S3). **Model C:** excluding triiodoacetophenone **9a** decomposition (Scheme S4). **Model D:** excluding di- **8a** and triiodoacetophenone **9a** decomposition (Scheme S5). **Model E:** excluding diiodo- **8a**, triiodo- **9a** and acetophenone **1a** decomposition (Scheme S6). **Model F:** excluding diiodo- **8a**, triiodo- **9a** and acetophenone **1a**, and DBU•I_2 decomposition (Scheme S7).

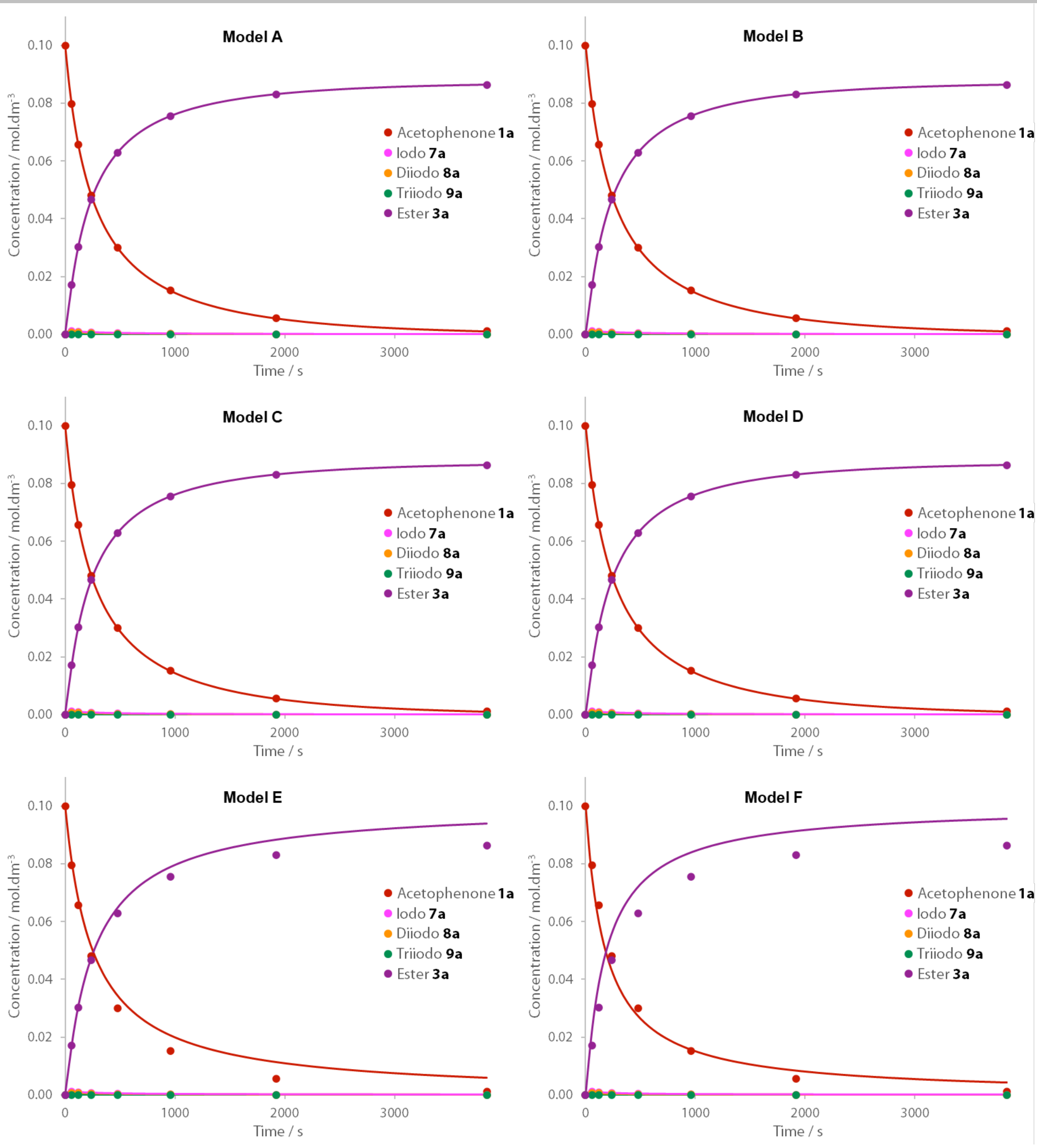

Figure S5. Time course plots for the kinetic reaction with primary alcohol **2a** (under primary alcohol conditions) using different COPASI models. Experimental concentrations shown as points; simulated data shown as solid lines. **Model A:** model presented in the manuscript (Scheme S2). **Model B:** including iodoacetophenone **7a** decomposition (Scheme S3). **Model C:** excluding triiodoacetophenone **9a** decomposition (Scheme S4). **Model D:** excluding di- **8a** and triiodoacetophenone **9a** decomposition (Scheme S5). **Model E:** excluding diiodo- **8a**, triiodo- **9a** and acetophenone **1a** decomposition (Scheme S6). **Model F:** excluding diiodo- **8a**, triiodo- **9a** and acetophenone **1a**, and DBU•I_2 decomposition (Scheme S7).

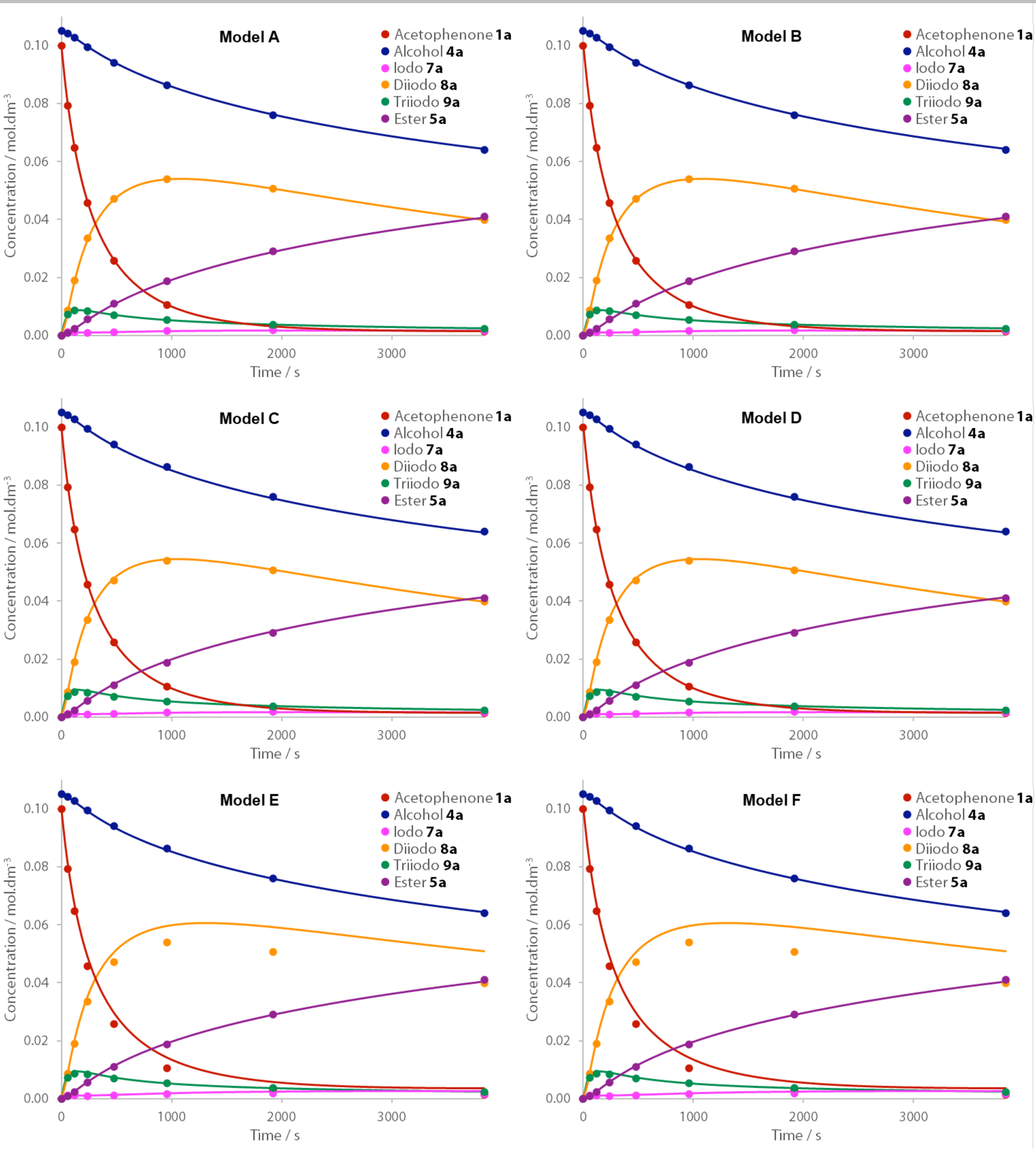

Figure S6. Time course plots for the kinetic reaction with secondary alcohol **4a** (under primary alcohol conditions) using different COPASI models. Experimental concentrations shown as points; simulated data shown as solid lines. **Model A:** model presented in the manuscript (Scheme S2). **Model B:** including iodoacetophenone **7a** decomposition (Scheme S3). **Model C:** excluding triiodoacetophenone **9a** decomposition (Scheme S4). **Model D:** excluding di- **8a** and triiodoacetophenone **9a** decomposition (Scheme S5). **Model E:** excluding diiodo- **8a**, triiodo- **9a** and acetophenone **1a** decomposition (Scheme S6). **Model F:** excluding diiodo- **8a**, triiodo- **9a** and acetophenone **1a**, and DBU•I_2 decomposition (Scheme S7).

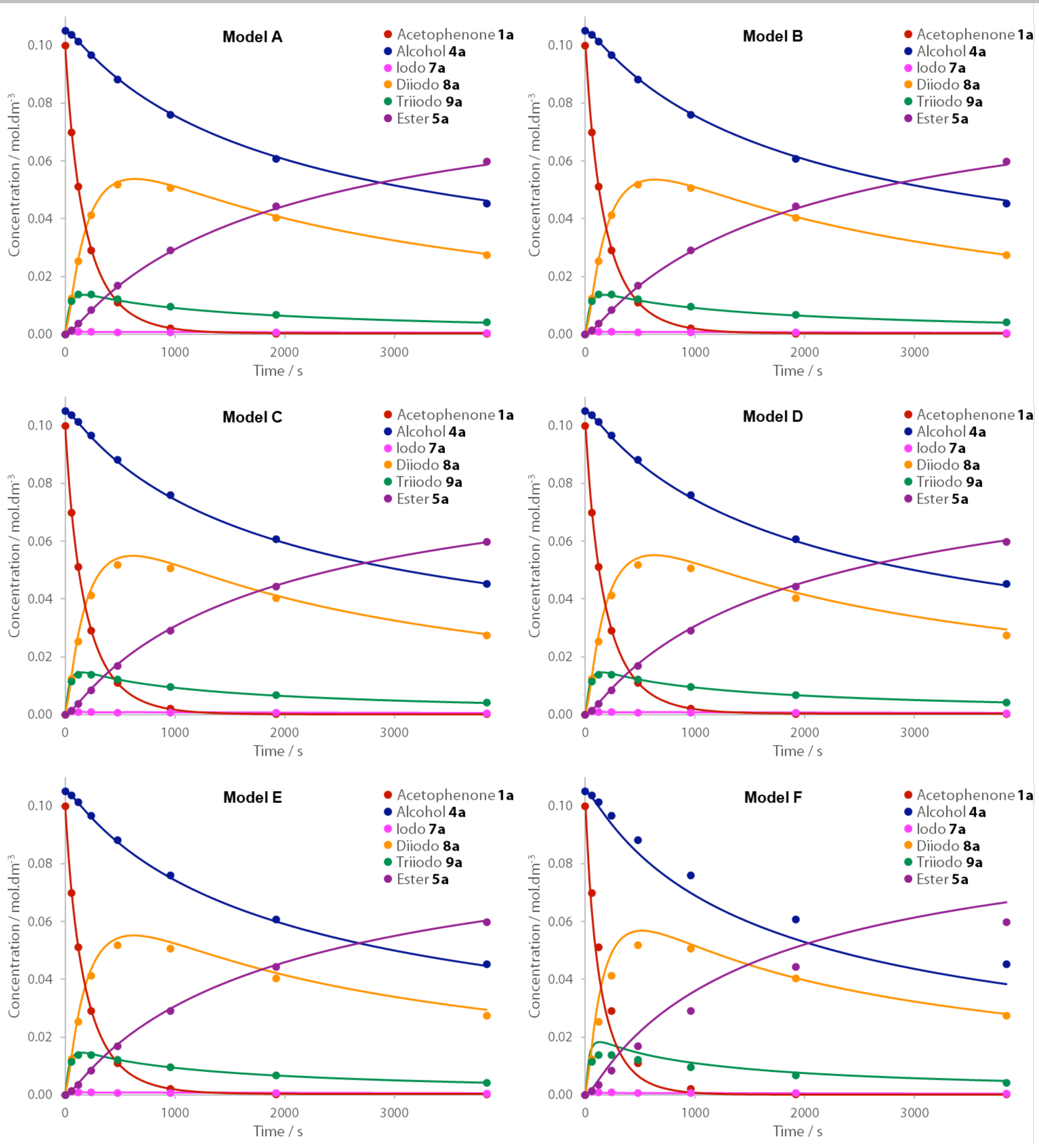

Figure S7. Time course plots for the kinetic reaction with secondary alcohol **4a** (under optimised secondary alcohol conditions) using different COPASI models. Experimental concentrations shown as points; simulated data shown as solid lines. **Model A:** model presented in the manuscript (Scheme S2). **Model B:** including iodoacetophenone **7a** decomposition (Scheme S3). **Model C:** excluding triiodoacetophenone **9a** decomposition (Scheme S4). **Model D:** excluding di- **8a** and triiodoacetophenone **9a** decomposition (Scheme S5). **Model E:** excluding diiodo- **8a**, triiodo- **9a** and acetophenone **1a** decomposition (Scheme S6). **Model F:** excluding diiodo- **8a**, triiodo- **9a** and acetophenone **1a**, and DBU•I_2 decomposition (Scheme S7).

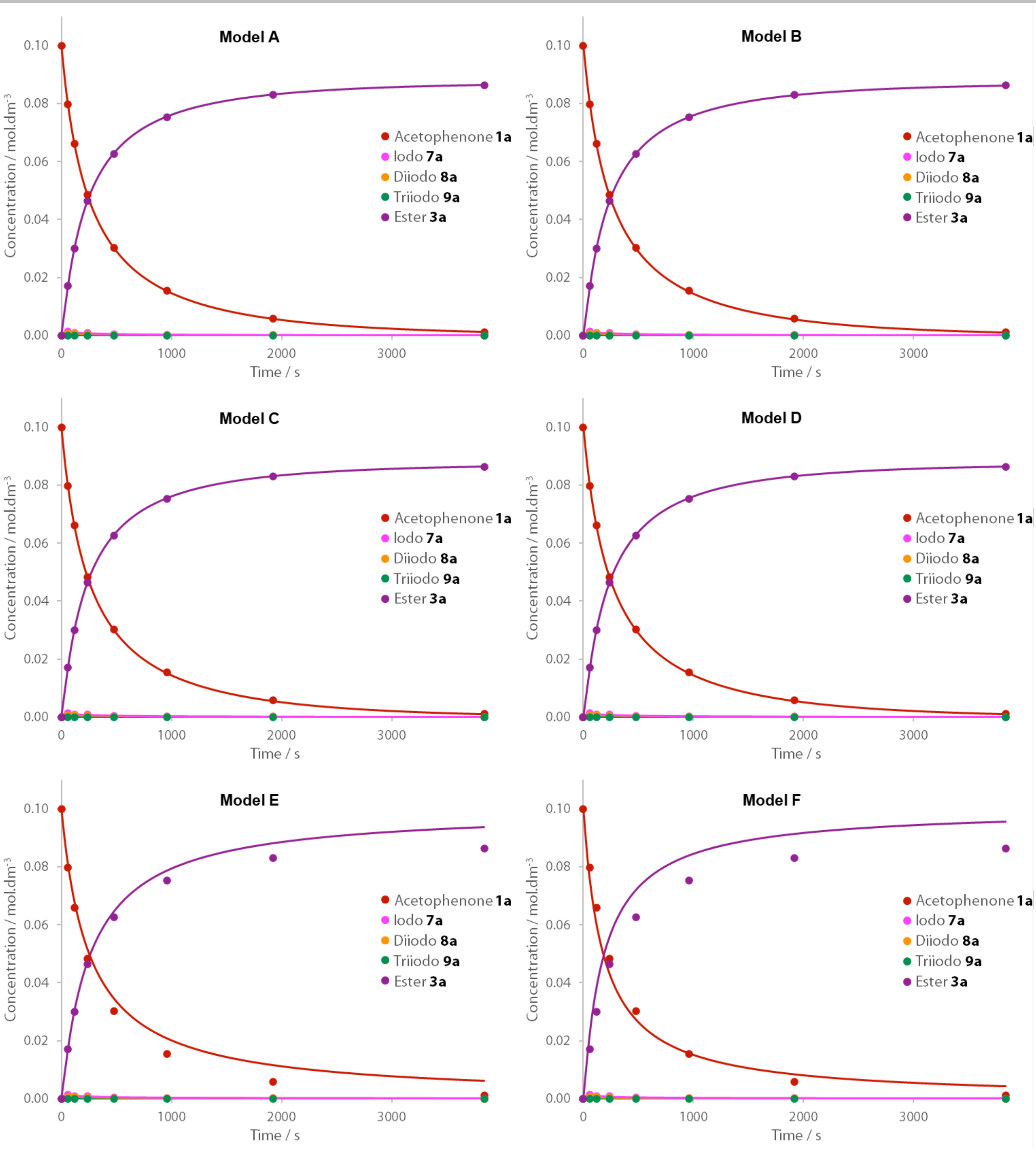

Figure S8. Time course plots for the kinetic reaction with primary alcohol **2a** and DBU•HI (1 equiv.) using different COPASI models. Experimental concentrations shown as points; simulated data shown as solid lines. **Model A:** model presented in the manuscript (Scheme S2). **Model B:** including iodoacetophenone **7a** decomposition (Scheme S3). **Model C:** excluding triiodoacetophenone **9a** decomposition (Scheme S4). **Model D:** excluding di- **8a** and triiodoacetophenone **9a** decomposition (Scheme S5). **Model E:** excluding diiodo- **8a**, triiodo- **9a** and acetophenone **1a** decomposition (Scheme S6). **Model F:** excluding diiodo- **8a**, triiodo- **9a** and acetophenone **1a**, and DBU•I_2 decomposition (Scheme S7).

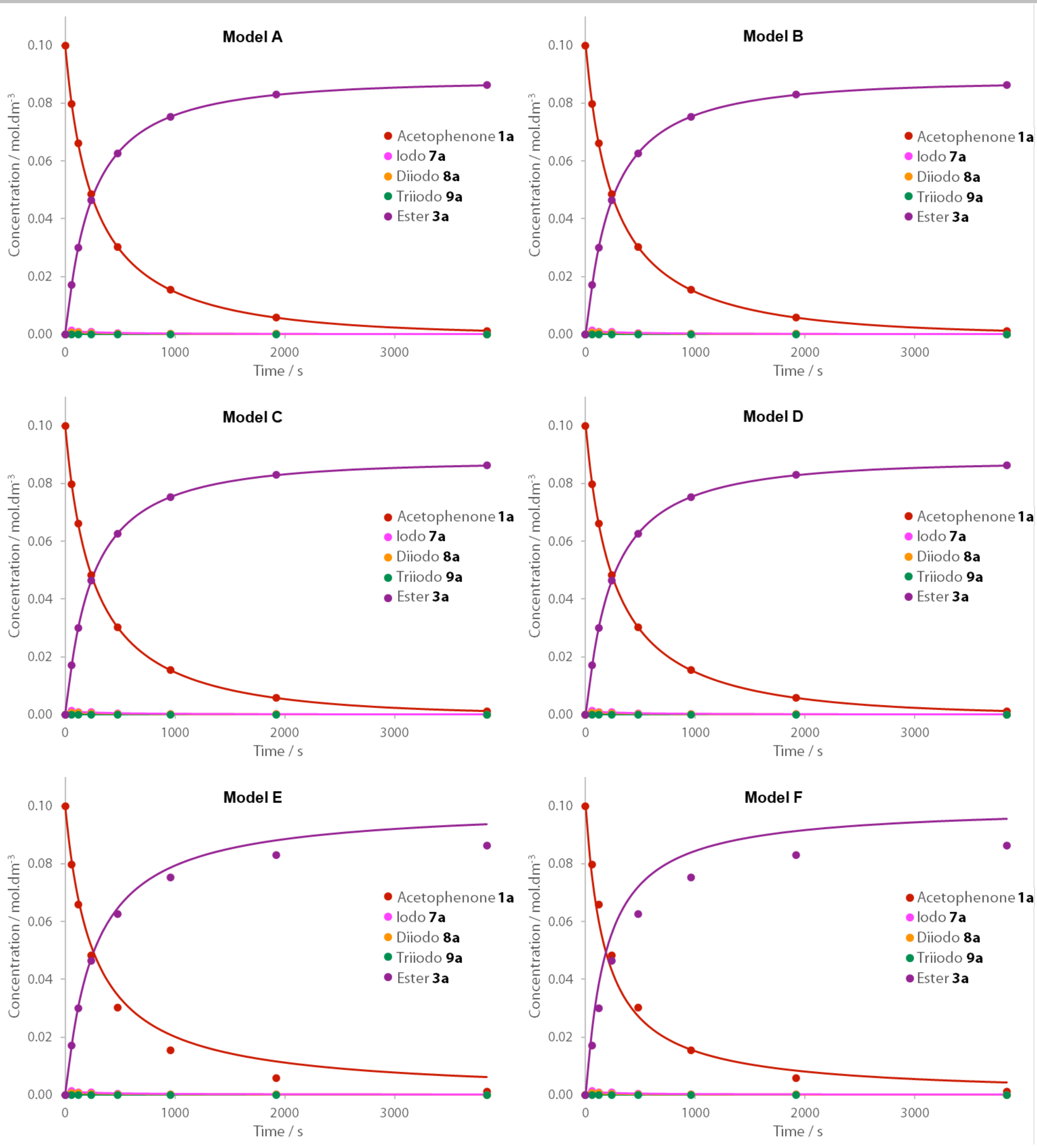

Figure S9. Time course plots for the kinetic reaction with primary alcohol **2a** and DBU•HI (2 equiv.) using different COPASI models. Experimental concentrations shown as points; simulated data shown as solid lines. **Model A:** model presented in the manuscript (Scheme S2). **Model B:** including iodoacetophenone **7a** decomposition (Scheme S3). **Model C:** excluding triiodoacetophenone **9a** decomposition (Scheme S4). **Model D:** excluding di- **8a** and triiodoacetophenone **9a** decomposition (Scheme S5). **Model E:** excluding diiodo- **8a**, triiodo- **9a** and acetophenone **1a** decomposition (Scheme S6). **Model F:** excluding diiodo- **8a**, triiodo- **9a** and acetophenone **1a**, and DBU•I_2 decomposition (Scheme S7).

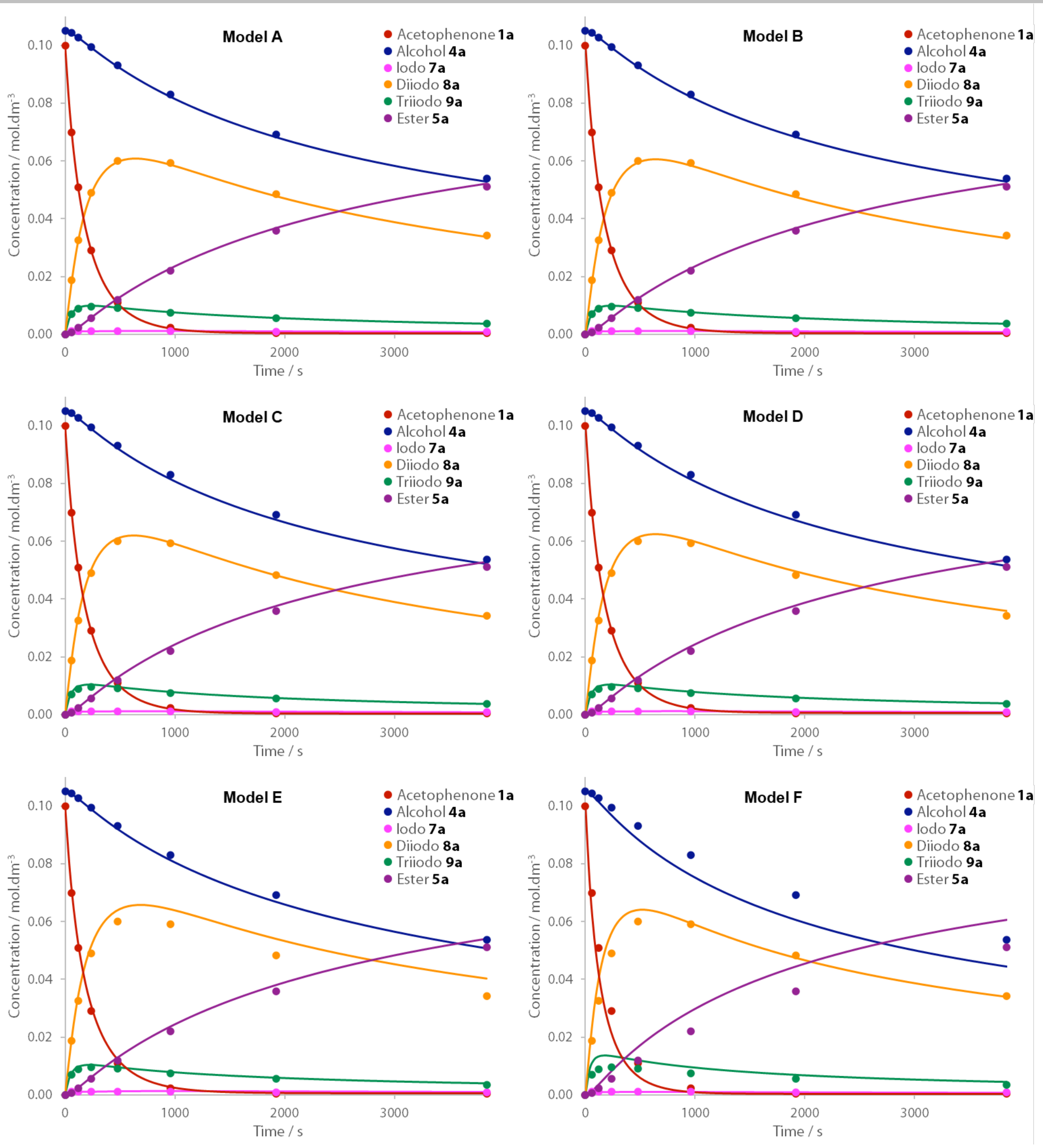

Figure S10. Time course plots for the kinetic reaction with secondary alcohol **4a** and DBU•HI (1 equiv.) using different COPASI models. Experimental concentrations shown as points; simulated data shown as solid lines. **Model A:** model presented in the manuscript (Scheme S2). **Model B:** including iodoacetophenone **7a** decomposition (Scheme S3). **Model C:** excluding triiodoacetophenone **9a** decomposition (Scheme S4). **Model D:** excluding di- **8a** and triiodoacetophenone **9a** decomposition (Scheme S5). **Model E:** excluding diiodo- **8a**, triiodo- **9a** and acetophenone **1a** decomposition (Scheme S6). **Model F:** excluding diiodo- **8a**, triiodo- **9a** and acetophenone **1a**, and DBU•I_2 decomposition (Scheme S7).

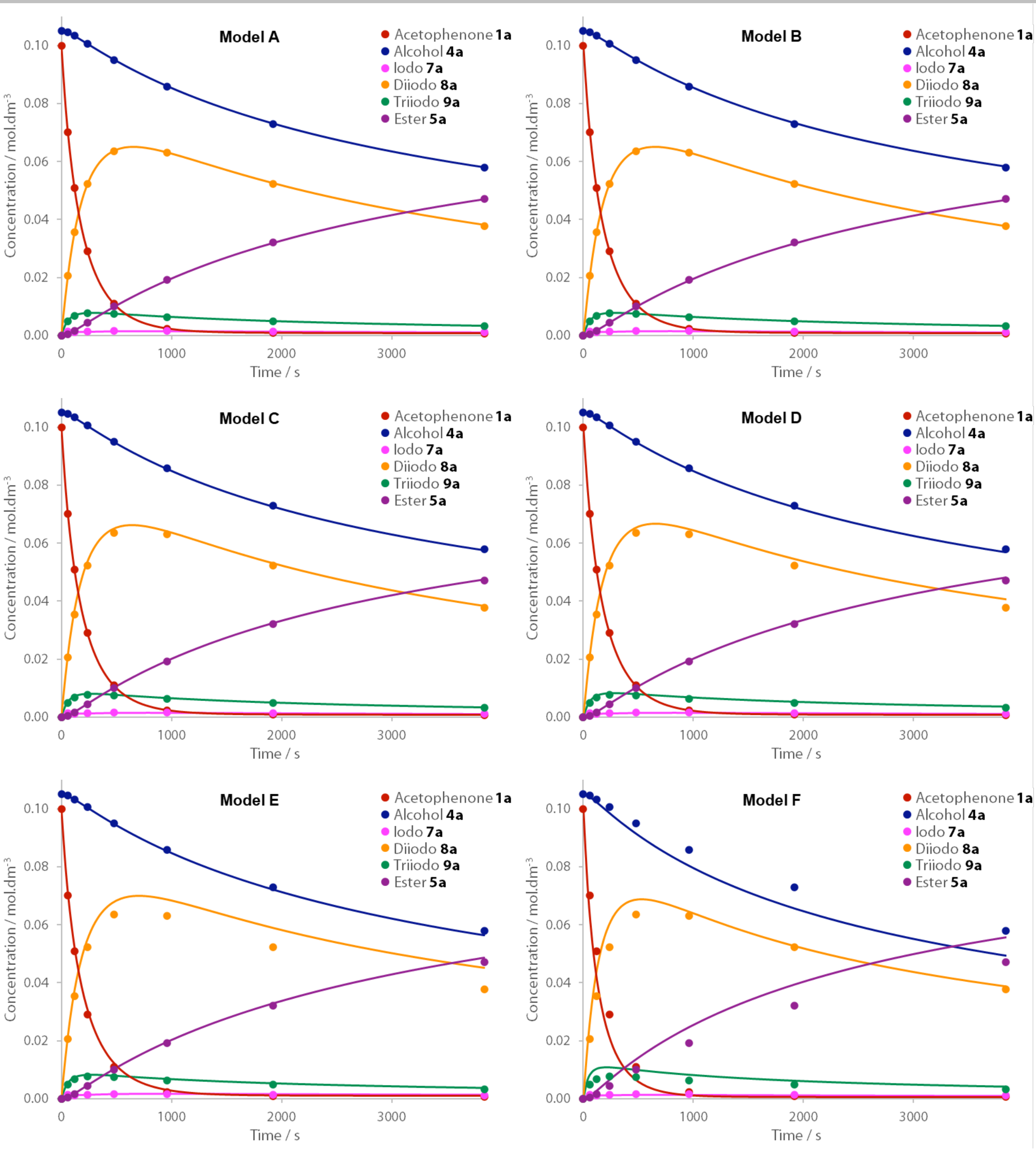

Figure S11. Time course plots for the kinetic reaction with secondary alcohol **4a** and DBU•HI (2 equiv.) using different COPASI models. Experimental concentrations shown as points; simulated data shown as solid lines. **Model A:** model presented in the manuscript (Scheme S2). **Model B:** including iodoacetophenone **7a** decomposition (Scheme S3). **Model C:** excluding triiodoacetophenone **9a** decomposition (Scheme S4). **Model D:** excluding di- **8a** and triiodoacetophenone **9a** decomposition (Scheme S5). **Model E:** excluding diiodo- **8a**, triiodo- **9a** and acetophenone **1a** decomposition (Scheme S6). **Model F:** excluding diiodo- **8a**, triiodo- **9a** and acetophenone **1a**, and DBU•I_2 decomposition (Scheme S7).

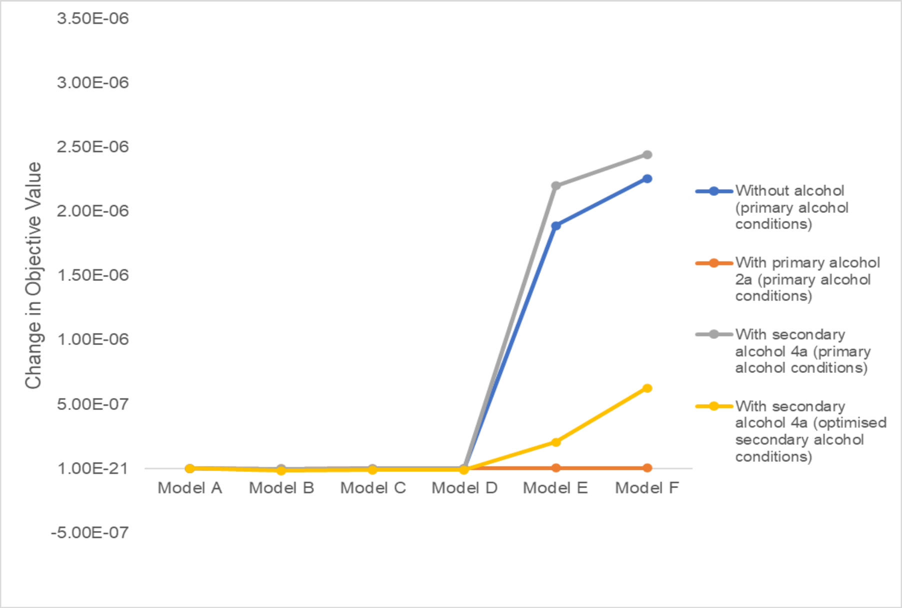

Figure S12. Calculated changes in objective values (relative to model A) for COPASI models of kinetics reactions not featuring DBU•HI. **Model A:** model presented in the manuscript (Scheme S2). **Model B:** including iodoacetophenone **7a** decomposition (Scheme S3). **Model C:** excluding triiodoacetophenone **9a** decomposition (Scheme S4). **Model D:** excluding di- **8a** and triiodoacetophenone **9a** decomposition (Scheme S5). **Model E:** excluding diiodo- **8a**, triiodo- **9a** and acetophenone **1a** decomposition (Scheme S6). **Model F:** excluding diiodo- **8a**, triiodo- **9a** and acetophenone **1a**, and DBU•I_2 decomposition (Scheme S7).

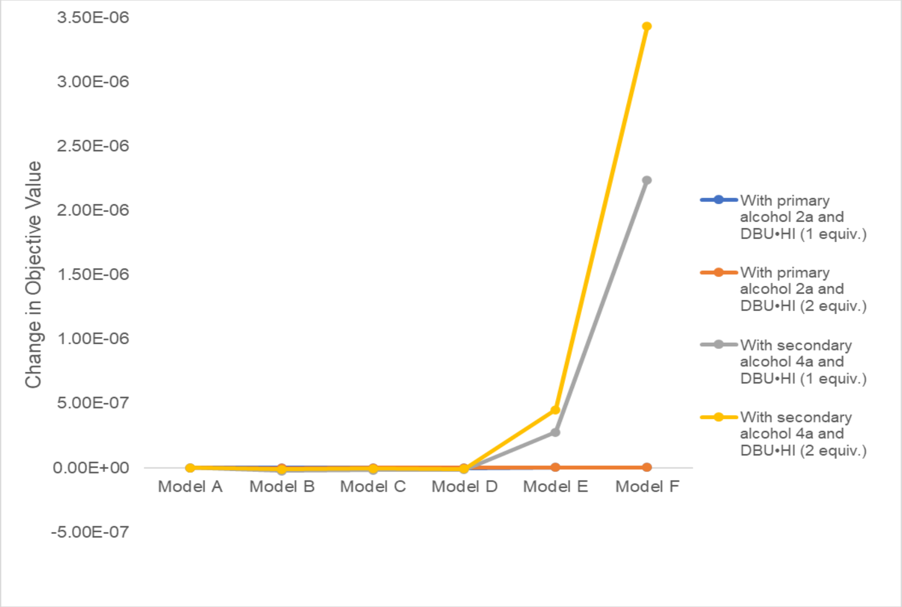

Figure S13. Calculated changes in objective values (relative to model A) for COPASI models of kinetics reactions featuring DBU•HI. **Model A:** model presented in the manuscript (Scheme S2). **Model B:** including iodoacetophenone **7a** decomposition (Scheme S3). **Model C:** excluding triiodoacetophenone **9a** decomposition (Scheme S4). **Model D:** excluding di- **8a** and triiodoacetophenone **9a** decomposition (Scheme S5). **Model E:** excluding diiodo- **8a**, triiodo- **9a** and acetophenone **1a** decomposition (Scheme S6). **Model F:** excluding diiodo- **8a**, triiodo- **9a** and acetophenone **1a**, and DBU•I_2 decomposition (Scheme S7).

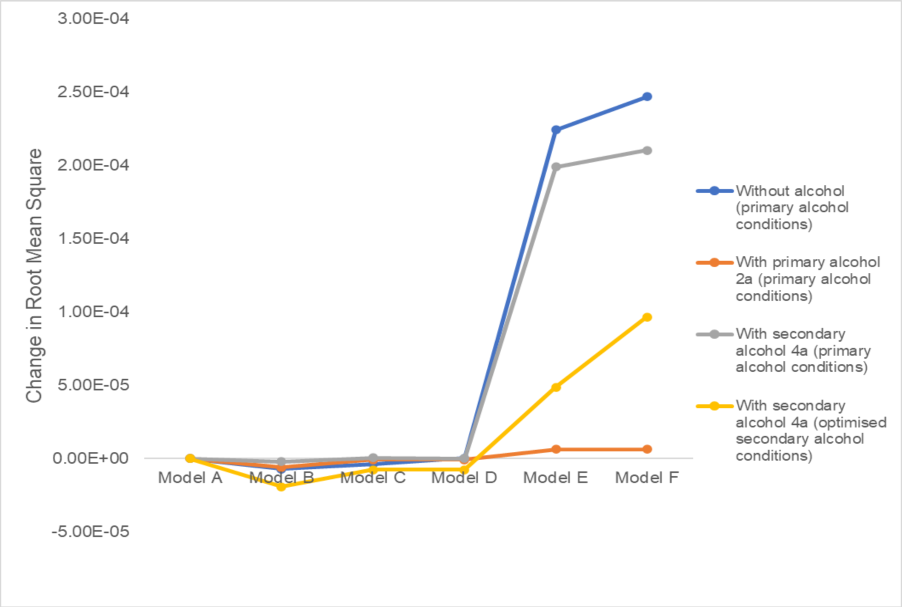

Figure S14. Calculated changes in root mean squares (relative to model A) for COPASI models of kinetics reactions not featuring DBU•HI. **Model A:** model presented in the manuscript (Scheme S2). **Model B:** including iodoacetophenone **7a** decomposition (Scheme S3). **Model C:** excluding triiodoacetophenone **9a** decomposition (Scheme S4). **Model D:** excluding di- **8a** and triiodoacetophenone **9a** decomposition (Scheme S5). **Model E:** excluding diiodo- **8a**, triiodo- **9a** and acetophenone **1a** decomposition (Scheme S6). **Model F:** excluding diiodo- **8a**, triiodo- **9a** and acetophenone **1a**, and DBU•I_2 decomposition (Scheme S7).

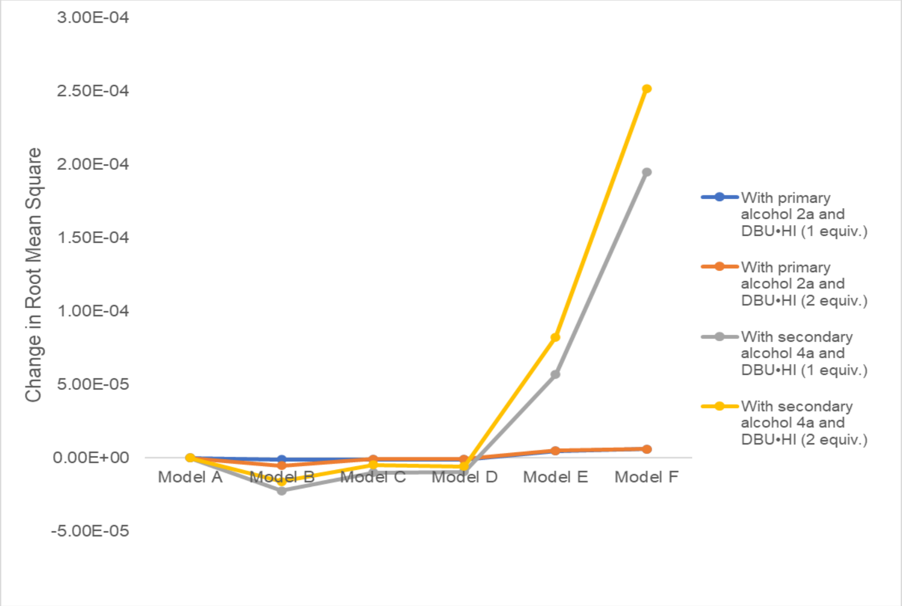

Figure S15. Calculated changes in root mean squares (relative to model A) for COPASI models of kinetics reactions featuring DBU•HI. **Model A:** model presented in the manuscript (Scheme S2). **Model B:** including iodoacetophenone **7a** decomposition (Scheme S3). **Model C:** excluding triiodoacetophenone **9a** decomposition (Scheme S4). **Model D:** excluding di- **8a** and triiodoacetophenone **9a** decomposition (Scheme S5). **Model E:** excluding diiodo- **8a**, triiodo- **9a** and acetophenone **1a** decomposition (Scheme S6). **Model F:** excluding diiodo- **8a**, triiodo- **9a** and acetophenone **1a**, and DBU•I_2 decomposition (Scheme S7).

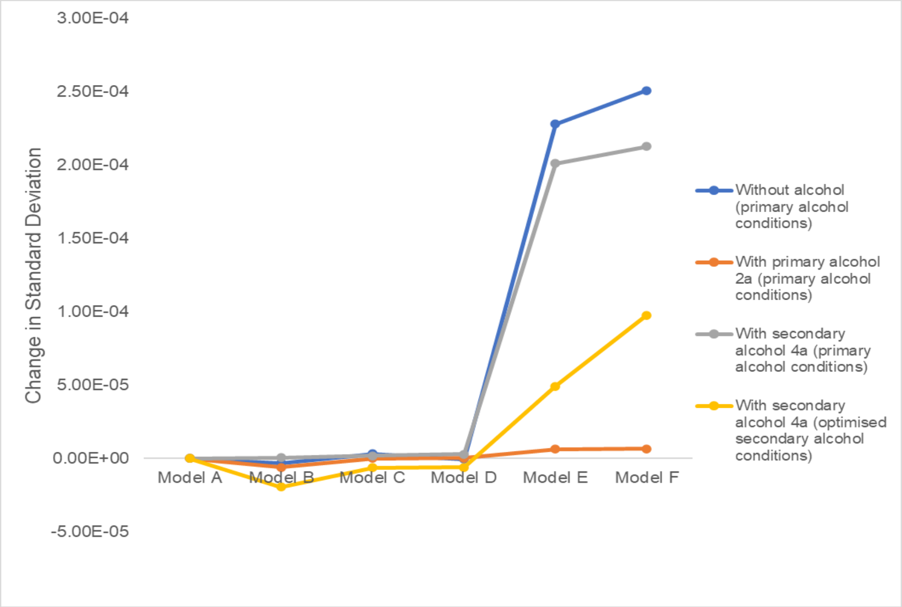

Figure S16. Calculated changes in standard deviations (relative to model A) for COPASI models of kinetics reactions not featuring DBU•HI. **Model A:** model presented in the manuscript (Scheme S2). **Model B:** including iodoacetophenone **7a** decomposition (Scheme S3). **Model C:** excluding triiodoacetophenone **9a** decomposition (Scheme S4). **Model D:** excluding di- **8a** and triiodoacetophenone **9a** decomposition (Scheme S5). **Model E:** excluding diiodo- **8a**, triiodo- **9a** and acetophenone **1a** decomposition (Scheme S6). **Model F:** excluding diiodo- **8a**, triiodo- **9a** and acetophenone **1a**, and DBU•I_2 decomposition (Scheme S7).

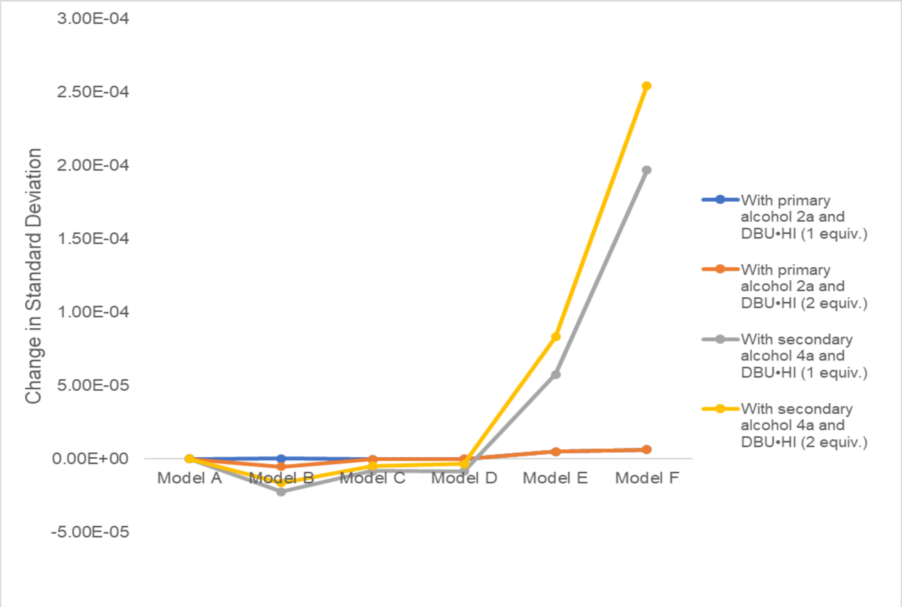

Figure S17. Calculated changes in standard deviations (relative to model A) for COPASI models of kinetics reactions featuring DBU•HI. **Model A:** model presented in the manuscript (Scheme S2). **Model B:** including iodoacetophenone **7a** decomposition (Scheme S3). **Model C:** excluding triiodoacetophenone **9a** decomposition (Scheme S4). **Model D:** excluding di- **8a** and triiodoacetophenone **9a** decomposition (Scheme S5). **Model E:** excluding diiodo- **8a**, triiodo- **9a** and acetophenone **1a** decomposition (Scheme S6). **Model F:** excluding diiodo- **8a**, triiodo- **9a** and acetophenone **1a**, and DBU•I_2 decomposition (Scheme S7).

Secondary Alcohols

Optimisation

Procedure

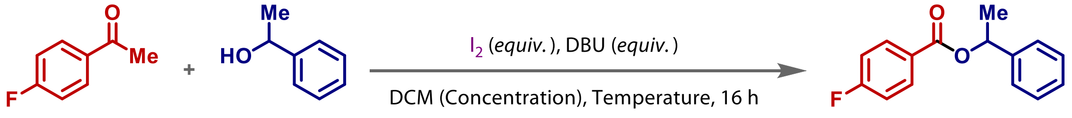

1a 0.412 mmol | **4a** 1.05 equiv. | **5a**

To an oven-dried Schlenk tube, which had been evacuated and refilled with nitrogen (×3), was added 4'-fluoroacetophenone **1a** (50.0 μL, 0.412 mmol, 1 equiv.), 1-(2-fluorophenyl)ethanol **4a** (54.6 μL, 0.432 mmol, 1.05 equiv.), 4,4'-difluorobiphenyl internal standard (23.5 mg, 0.124 mmol, 0.3 equiv.) and anhydrous DCM.[a,b,c] A small aliquot (~25 μL) of the resulting solution was taken for ^{19}F NMR analysis, then iodine was added in one portion. The reaction mixture was cooled to the desired temperature in an appropriate cooling bath, then DBU was added dropwise. The reaction mixture was stirred for 16 h, with the reaction allowed to warm to room temperature. On completion, an NMR sample was prepared by diluting a 100-μL aliquot of reaction mixture with $CDCl_3$ (400 μL). ^{19}F NMR yields were calculated by comparison to the **1a**:internal standard ratio prior to the reaction.

a: Stock solutions were used for most reactions, prepared as follows: to an oven-dried Schlenk tube with a Young's tap, which had been evacuated and refilled with nitrogen (×3), was added 4'-fluoroacetophenone **1a** (1.00 mL, 8.24 mmol, 1 equiv.), 1-(2-fluorophenyl)ethanol **4a** (1.09 mL, 8.63 mmol, 1.05 equiv.), 4,4'-difluorobiphenyl internal standard (470 mg, 2.47 mmol, 0.3 equiv.) and anhydrous DCM (17.9 mL). A 1-mL aliquot of the stock solution (corresponding to 0.412 mmol **1a**, 0.432 mmol **4a**, 0.124 mmol internal standard) was diluted with anhydrous DCM (3.1 mL) for each reaction, with no aliquot taken for pre-reaction ^{19}F NMR analysis (this was carried out on a separate aliquot of the stock solution).

b: For reactions with controlled addition of **1a** and **4a** (Table S28, entries 6-8): to an oven-dried Schlenk tube, which had been evacuated and refilled with nitrogen (×3), was added 4'-fluoroacetophenone **1a** (62.5 μL, 0.515 mmol, 1.25 equiv.), 1-(2-fluorophenyl)ethanol **2c** (68.3 μL, 0.541 mmol, 1.31 equiv.), 4,4'-difluorobiphenyl internal standard (29.4 mg, 0.155 mmol, 0.375 equiv.) and anhydrous DCM (1.12 mL). This solution was drawn into a 1-mL syringe, from which 1 mL (corresponding to 0.412 mmol **1a**, 0.432 mmol **4a**, 0.124 mmol internal standard) was dispensed into the mixture of iodine and DBU in DCM (3.1 mL) over the desired time, starting immediately after addition of DBU.

c: For reactions with molecular sieves (Table S29, entries 11-16): 3 Å molecular sieves (410 mg) were activated in the Schlenk tube prior to use.

Results

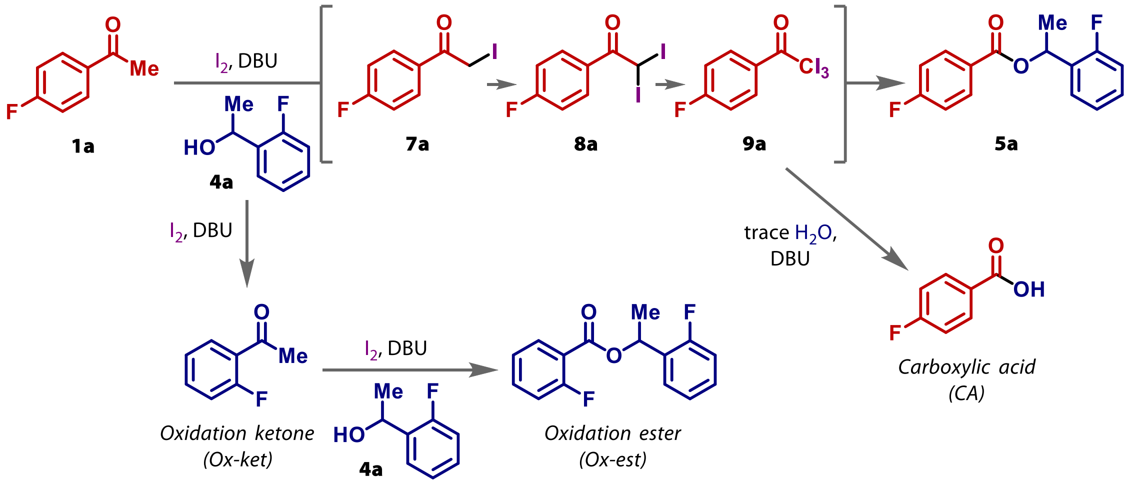

Scheme S8. Overview of routes to possible side-products in haloform coupling between acetophenone **1a** and secondary alcohol **4a**.

Table S28. Effect of varying temperatures, concentrations, and addition rates on product distributions in haloform coupling with secondary alcohol **4a**.[a]

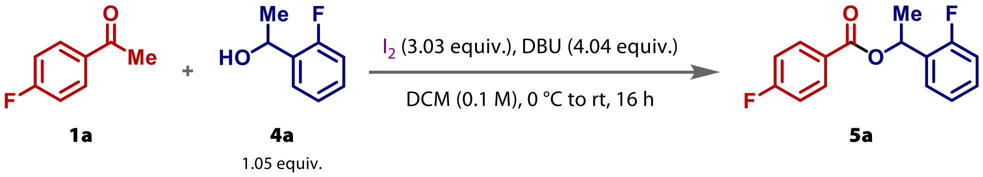

Entry	Change from conditions above	Acetophenone **1a**[b]	Iodo **7a**[b]	Diiodo **8a**[b]	Alcohol **4a**[b]	Ox-ket[b]	Ox-est[b]	CA[b]	Ester **5a**[b]
1	–	trace	13%	trace	59%	0%	trace	1%	**32%**
2	−20 °C to rt	0%	0%	0%	52%	0%	0%	15%	**40%**
3	rt	0%	27%	trace	60%	0%	trace	trace	**23%**
4	0.05 M	1%	19%	0%	58%	0%	trace	trace	**34%**
5	0.2 M	0%	12%	trace	55%	0%	trace	1%	**35%**
6	**1a** and **4a** added over 2 h	1%	17%	trace	69%	0%	trace	1%	**27%**
7	**1a** and **4a** added over 5 h	0%	7%	0%	51%	0%	trace	8%	**42%**
8	**1a** and **4a** added over 8 h	18%	8%	0%	76%	0%	trace	4%	**22%**

a: On 0.412 mmol scale. b: Percentage relative to acetophenone **1a** in mixture prior to reaction; calculated by ^{19}F NMR vs 4,4’-difluorobiphenyl internal standard.

Table S29. Effect of varying reagent stoichiometries on product distributions in haloform coupling with secondary alcohol **4a**.[a]

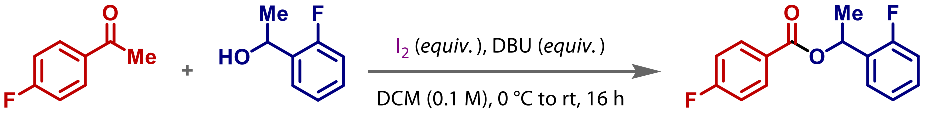

Entry	I_2 equiv.	DBU equiv.	Additive	Acetophenone **1a**[b]	Iodo **8a**[b]	Diiodo **9a**[b]	Alcohol **4a**[b]	Ox-ket[b]	Ox-est[b]	CA[b]	Ester **5a**[b]
1	3.03	4.04	–	trace	13%	trace	59%	0%	trace	1%	**32%**
2	6	4.04	–	74%	14%	11%	4%	59%	0%	0%	**trace**
3	3.03	8	–	0%	0%	0%	95%	0%	trace	12%	**29%**
4	6	8	–	0%	trace	0%	12%	0%	2%	11%	**77%**
5	3.3	4.4	–	0%	7%	0%	54%	0%	trace	1%	**42%**
6	3.6	4.8	–	0%	trace	0%	39%	0%	trace	5%	**57%**
7	3.9	5.2	–	0%	0%	0%	33%	0%	trace	9%	**64%**
8	4.2	5.6	–	0%	0%	0%	31%	0%	trace	15%	**69%**
9	4.5	6	–	0%	0%	0%	23%	0%	1%	13%	**75%**
10	7.5	10	–	0%	trace	0%	12%	0%	2%	10%	**79%**
11	4.5	6	3 Å MS	0%	0%	0%	13%	0%	1%	trace	**82%**
12	4.8	6.4	3 Å MS	0%	0%	0%	11%	0%	1%	trace	**85%**
13	5.1	6.8	3 Å MS	0%	0%	0%	11%	0%	1%	trace	**85%**
14	5.4	7.2	3 Å MS	0%	0%	0%	13%	0%	1%	1%	**81%**
15	5.7	7.6	3 Å MS	0%	trace	0%	11%	0%	1%	1%	**86%**
16	6	8	3 Å MS	0%	trace	0%	11%	0%	1%	2%	**84%**

a: On 0.412 mmol scale. b: Percentage relative to acetophenone **1a** in mixture prior to reaction; calculated by ^{19}F NMR vs 4,4'-difluorobiphenyl internal standard.

Synthesis of Side-Products

1-(2-Fluorophenyl)ethyl 2-fluorobenzoate

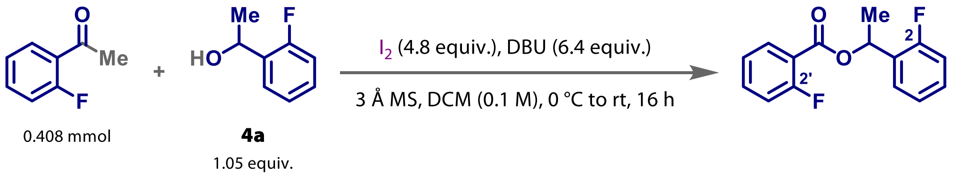

To an oven-dried Schlenk tube containing activated 3 Å molecular sieves (410 mg), which had been evacuated and refilled with nitrogen (×3), was added 2'-fluoroacetophenone (56.3 mg, 0.408 mmol, 1 equiv.), 1-(2-fluorophenyl)ethanol **4a** (54.0 µL, 0.428 mmol, 1.05 equiv.) and anhydrous DCM (4.1 mL), followed by addition of iodine (496 mg, 1.96 mmol, 4.8 equiv.) in one portion. The reaction mixture was cooled to 0 °C (ice-water bath), then DBU (390 µL, 2.61 mmol, 6.4 equiv.) was added dropwise. The reaction mixture was stirred for 16 h and allowed to warm to room temperature. The reaction mixture was diluted with EtOAc, filtered, then added to sat. aq. $Na_2S_2O_3$. The mixture was separated and the aqueous layer was extracted a further two times with EtOAc. The combined organic fractions were dried over anhydrous $MgSO_4$, filtered, and concentrated under vacuum. The crude product was purified by FCC (eluted with 5% EtOAc in pentane) to obtain the title compound as a colourless oil (81.2 mg, 76%).

^{1}H NMR (400 MHz, $CDCl_3$) δ 7.88 (ddd, *J* = 7.5, 1.5, 1.0 Hz, 1H, Ar-H), 7.76 (ddd, *J* = 9.5, 2.5, 1.5 Hz, 1H, Ar-H), 7.50 – 7.38 (m, 2H, Ar-H), 7.32 – 7.22 (m, 2H, Ar-H), 7.15 (app td, *J* = 7.5, 1.0 Hz, 1H, Ar-H), 7.07 (ddd, *J* = 10.5, 8.0, 1.0 Hz, 1H, Ar-H), 6.37 (q, *J* = 6.5 Hz, 1H, C*H*Me), 1.69 (d, *J* = 6.5 Hz, 3H, Me).

^{13}C NMR (101 MHz, $CDCl_3$) δ 164.6 (d, *J* = 3.0 Hz, C=O), 162.7 (d, *J* = 247.0 Hz, Ar), 160.0 (d, *J* = 247.5 Hz, Ar), 132.7 (d, *J* = 7.5 Hz, Ar), 130.2 (d, *J* = 8.0 Hz, Ar), 129.7 (d, *J* = 8.5 Hz, Ar), 128.8 (d, *J* = 13.5 Hz, Ar), 127.3 (d, *J* = 4.0 Hz, Ar), 125.6 (d, *J* = 3.0 Hz, Ar), 124.5 (d, *J* = 3.5 Hz, Ar), 120.2 (d, *J* = 21.0 Hz, Ar), 116.7 (d, *J* = 23.0 Hz, Ar), 115.9 (d, *J* = 21.5 Hz, Ar), 68.1 (d, *J* = 3.0 Hz, CHMe), 21.4 (d, *J* = 1.0 Hz, Me).

^{19}F NMR (376 MHz, $CDCl_3$) δ −112.35 (ddd, *J* = 9.5, 8.5, 5.5 Hz, 1F, 2'-F), −118.21 (ddd, *J* = 10.5, 7.5, 5.0 Hz, 1F, 2-F).

HRMS (EI) m/z: $[M]^+$ Calcd for $C_{15}H_{12}O_2F_2$ 262.0800; found 262.0798; 0.76 ppm error.

IR (neat) ν_{max} / cm^{-1}: 1716, 1613, 1489, 1455, 1294, 1249, 1230, 1124, 1064, 753.

Investigation of Inorganic Base Additives to Form Metal Iodide Salts

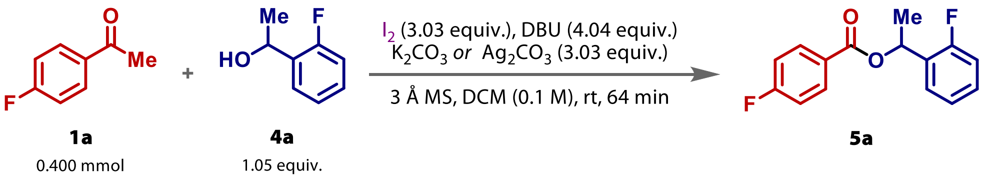

To an oven-dried Schlenk tube containing activated 3 Å molecular sieves (400 mg), which had been evacuated and refilled with nitrogen (×3), was added 4'-fluoroacetophenone **1a** (48.6 μL, 0.400 mmol, 1 equiv.), 1-(2-fluorophenyl)ethanol **4a** (53.0 μL, 0.420 mmol, 1.05 equiv.), 4,4'-difluorobiphenyl internal standard (23.5 mg, 0.124 mmol, 0.3 equiv.), anhydrous K_2CO_3/Ag_2CO_3 (1.21 mmol, 3.03 equiv.) and anhydrous DCM (4 mL). A small aliquot (~25 μL) of the resulting solution was taken for ^{19}F NMR analysis, then iodine (308 mg, 1.21 mmol, 3.03 equiv.) was added in one portion. DBU (241 μL, 1.62 mmol, 4.04 equiv.) was then added in one portion and the reaction was monitored in the same manner as the time course experiments, i.e. ~250-μL aliquots of the reaction mixture were taken after 1, 2, 4, 8, 16, 32 and 64 min. Aliquots were immediately quenched with sat. aq. NH_4Cl (~0.7 mL) and NMR samples were prepared by diluting a 100-μL aliquot of the quenched reaction mixture with $CDCl_3$ (400 μL). ^{19}F NMR yields were calculated by comparison to the **1a**:internal standard ratio prior to reaction.

Addition of K_2CO_3 resulted in a small increase in the yield of ester **5a** over 64 min: 45% with K_2CO_3 vs 41% without (see Figure S18 for overlayed time course plots). Addition of Ag_2CO_3 caused the reaction mixture to solidify, preventing samples being taken for analysis.

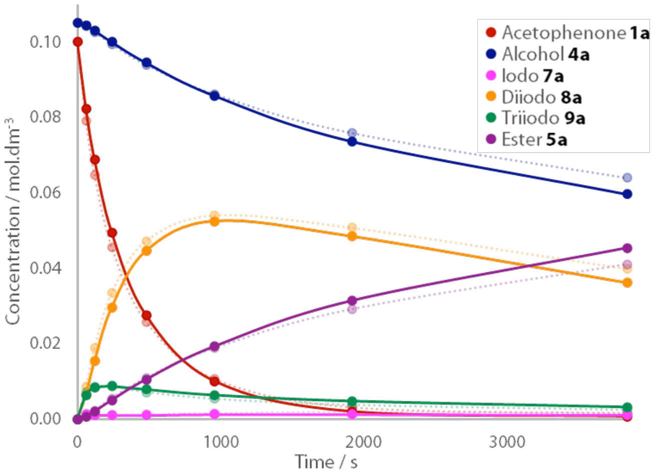

Figure S18. Time course plots for the reaction of secondary alcohol **4a** under primary alcohol conditions with (solid lines, foreground) and without (dotted lines, background) K_2CO_3 added. NB: lines are not based on modelled data; they are merely intended to guide the eye by connecting the datapoints.

Robustness Screen

The robustness screen was based on the protocol reported by Glorius and co-workers,[41] and used the 'Group A' functional group additives from their initial report.[42]

Calibration Procedure

The robustness screen results were calculated by GC-MS, using single-point calibrations for each of the additives, as well as the reaction product, 1-(2-fluorophenyl)ethyl 4-fluorobenzoate **5a**. Based on analyte retention times and incompatibilities, analytes (additives and reaction components) were calibrated in batches: Group 1 (additives): 1-dodecene, decanenitrile, 1-chlorododecane, acetanilide, methyl benzoate, benzonitrile, chlorobenzene, benzaldehyde; Group 2 (additives): 1-decyne, 6-undecanone, 1-nonanol, 2-vinylnaphthalene, 4-octyne, phenol, *N*-methylacetanilide; Group 3 (reaction components): 1-(2-fluorophenyl)ethanol **4a**, 2-iodo-4'-fluoroacetophenone **7a**, 1-(2-fluorophenyl)ethyl 4-fluorobenzoate **5a**, iodoform; Group 4 (reaction components): 4'-fluoroacetophenone **1a**, 1-(2-fluorophenyl)ethyl 2-fluorobenzoate. Dodecylamine and aniline were calibrated individually.

Calibration stock solutions were prepared containing the analytes (0.200 mmol, 1 equiv. of each) and mesitylene (as a standard; 27.8 µL, 0.200 mmol, 1 equiv.) in DCM (20 mL). For each calibration solution, a 30-µL aliquot was diluted with DCM (1.5 mL) and filtered through silica into a vial for analysis. The dodecylamine solution was prepared by filtering through Celite, to avoid removing the additive. Additional Group 1 and Group 2 samples were prepared by filtering through Celite, to avoid removing acetanilide and *N*-methylacetanilide, respectively.

Reaction Procedure

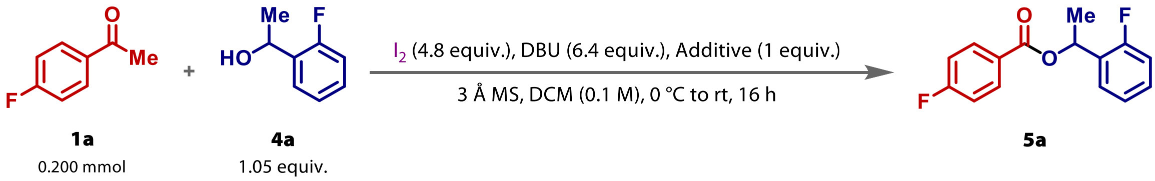

A stock solution was used, prepared as follows: to an oven-dried Schlenk tube with a Young's tap, which had been evacuated and refilled with nitrogen (×3), was added 4'-fluoroacetophenone **1a** (510 µL, 4.20 mmol, 1 equiv.), 1-(2-fluorophenyl)ethanol **4a** (557 µL, 4.41 mmol, 1.05 equiv.) and anhydrous DCM (19.9 mL). A fresh stock solution was prepared for running repeat reactions to check reproducibility.

To an oven-dried Schlenk tube containing activated 3 Å molecular sieves (200 mg), which had been evacuated and refilled with nitrogen (×3), was added a 1-mL aliquot of the stock solution (corresponding to 0.200 mmol **1a** and 0.210 mmol **4a**), an additive (0.200 mmol, 1 equiv.) and anhydrous DCM (1 mL), followed by addition of iodine (243 mg, 0.960 mmol, 4.8 equiv.) in one portion. The reaction mixture was cooled to 0 °C (ice-water bath), then DBU (191 µL, 1.28 mmol, 6.4 equiv.) was added dropwise. The reaction mixture was stirred for 16 h and allowed to warm to room temperature. On completion, sat. aq. $Na_2S_2O_3$ (1 mL) was added to the reaction mixture and stirred vigorously to quench any unreacted iodine. Mesitylene (GC-MS standard; 27.8 µL, 0.200 mmol, 1 equiv.) was then added to the stirring mixture. The biphasic mixture was allowed to partition, then a 100-µL aliquot of the DCM layer was diluted with DCM (900 µL). A 30-µL aliquot of this solution was then further diluted with DCM (1.5 mL) and filtered through silica into a vial for analysis by the same GC-MS method as used for calibration. Solutions from reactions with dodecylamine, acetanilide and *N*-methylacetanilide as additives were filtered through Celite, to avoid removing the additives.

Results

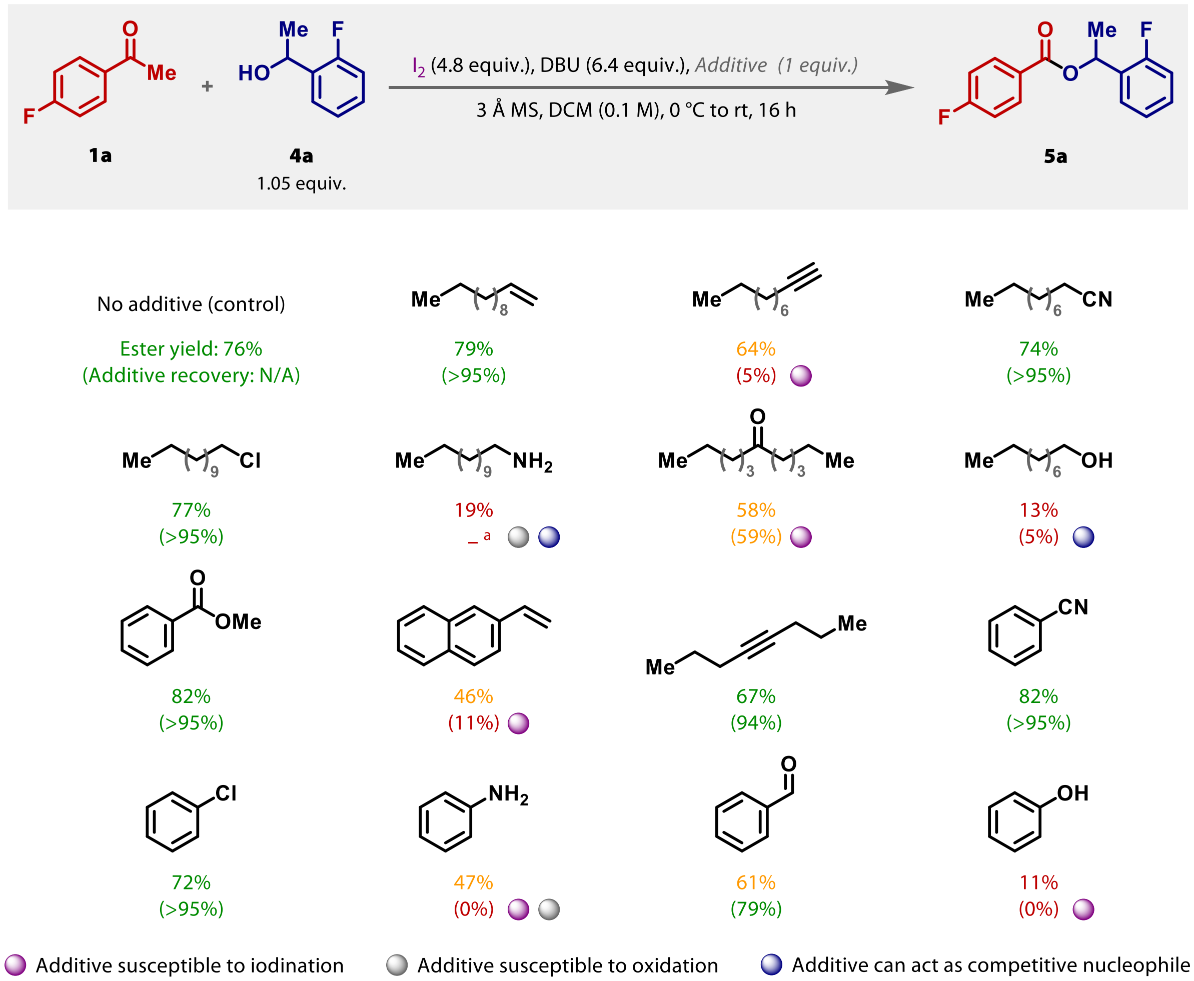

Scheme S9. Summary of robustness screen results. GC-MS yields and additive recoveries shown. Some results shown as an average from two reactions (see Table S30 for details). a: Additive recovery could not be quantified, due to co-elution with DBU.

Table S30. Complete robustness screen results.

Additive	Ester **5a** yield[a] (repeat)[b]	Additive recovery[a] (repeat)[b]	Side-products identified[c]
None (control)	79% (74%)	N/A	N/A
1-Dodecene	79%	99%	None
1-Decyne	64%	5%	Iododecyne
Decanenitrile	72% (77%)	103% (101%)	None
1-Chlorododecane	77%	107%	None
Dodecylamine[d]	20% (19%)	– (–)	Dodecanenitrile, dodecyl amide
6-Undecanone	55% (61%)	52% (66%)	None
1-Nonanol	13%	5%	Nonyl ester
Acetanilide[e]	96% (87%)	250% (235%)	None
Methyl benzoate	81% (83%)	95% (96%)	None
2-Vinylnaphthalene	46%	11%	None
4-Octyne	67%	94%	None
Benzonitrile	82%	100%	None
Chlorobenzene	72%	97%	None
Aniline	47%	0%	Iodoaniline, azobenzene, iodoazobenzene
Benzaldehyde	61%	79%	None
Phenol	11%	0%	Triiodophenol
N-Methylacetanilide[e]	87% (69%)	303% (359%)	None

a: Ester yields and additive recoveries calculated by comparison of analyte:mesitylene ratios in reaction mixtures and calibration solutions. b: Some reactions were repeated to verify reproducibility. c: Side-products identified by comparison of mass spectra of peaks in chromatogram to NERC database. d: Additive recovery could not be quantified, due to co-elution with DBU (which was not removed by filtering through Celite). e: Additive recoveries were reproducibly >>100%. The issue appeared to be calibration-related, but individual re-calibration of these additives did not resolve it. These results were therefore not included in the summary (Scheme S9) or discussed in the manuscript.

Substrate Synthesis

5-Acetyl-1-methyl-1H-pyrrole-2-carbonitrile

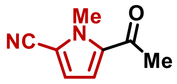

This substrate was provided by Luke Elliot (University of Bristol).

^{1}H NMR (400 MHz, CDCl$_3$) δ 7.39 (d, *J* = 2.0 Hz, 1H, Ar-H), 7.17 (d, *J* = 2.0 Hz, 1H, Ar-H), 3.82 (s, 3H, NMe), 2.40 (s, 3H, COMe).

^{13}C NMR (101 MHz, CDCl$_3$) δ 192.1 (C=O), 130.5 (Ar), 126.2 (Ar), 120.2 (Ar), 112.5 (CN), 106.5 (Ar), 36.1 (NMe), 27.4 (CO*Me*).

HRMS (EI) m/z: [M]$^+$ Calcd for $C_8H_8N_2O$ 148.0631; found 148.0628; 2.03 ppm error.

IR (neat) ν_{max} / cm^{-1}: 3115, 2217, 1667, 1542, 1240, 1195, 861, 649.

1-(2,6-Dimethylphenyl)ethanol

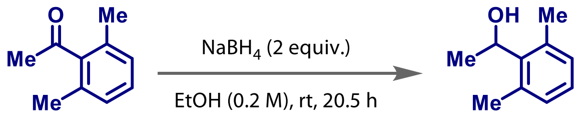

To an oven-dried round-bottom flask, which had been evacuated and refilled with nitrogen (×3), was added $NaBH_4$ (254 mg, 6.71 mmol, 2 equiv.) and anhydrous EtOH (16.8 mL), followed by addition of 2,6-dimethylacetophenone (497 mg, 3.35 mmol). The reaction mixture was stirred for 20.5 h, then concentrated under vacuum, dissolved in deionised water and extracted with DCM (×3). The combined organic fractions were concentrated under vacuum and the crude product was purified by FCC (eluted with 0-20% EtOAc in pentane) to obtain the title compound as a white solid (250 mg, 50%).

^{1}H NMR (400 MHz, CDCl$_3$) δ 7.05 (dd, *J* = 8.5, 6.0 Hz, 1H), 7.02 – 6.97 (m, 2H), 5.40 (qd, *J* = 7.0, 3.0 Hz, 1H), 2.45 (s, 6H), 1.74 (d, *J* = 3.0 Hz, 1H), 1.54 (d, *J* = 7.0 Hz, 3H).

^{13}C NMR (101 MHz, CDCl$_3$) δ 140.7, 135.8, 129.6, 127.1, 67.8, 21.6, 20.8.

Data are consistent with those previously reported (compound 16h).[43]

1-(4'-Fluoro-[1,1'-biphenyl]-4-yl)ethanol

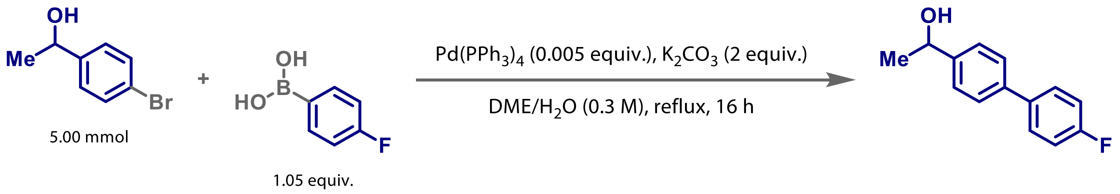

To a round-bottom flask was added 1-(4-bromophenyl)ethanol (685 µL, 5.00 mmol, 1 equiv.), (4-fluorophenyl)boronic acid (735 mg, 5.25 mmol, 1.05 equiv.), $Pd(PPh_3)_4$ (28.9 mg, 0.0250 mmol, 0.005 equiv.), DME (12.5 mL) and 2 M aq. K_2CO_3 (5 mL). The reaction mixture was stirred for 16 h under reflux, then allowed to cool to room temperature. Sat. aq. NH_4Cl was added, then the mixture was separated and the aqueous layer was extracted a further two times with DCM. The combined organic fractions were dried over anhydrous $MgSO_4$, filtered, and concentrated under vacuum. The crude product was purified by FCC (eluted with 25% EtOAc in pentane) to obtain the title compound as an off-white solid (777 mg, 72%).

^{1}H NMR (400 MHz, $CDCl_3$) δ 7.57 – 7.51 (m, 4H), 7.47 – 7.43 (m, 2H), 7.16 – 7.09 (m, 2H), 4.96 (q, *J* = 6.5 Hz, 1H), 1.54 (d, *J* = 6.5 Hz, 3H).

^{13}C NMR (151 MHz, $CDCl_3$) δ 162.6 (d, *J* = 246.5 Hz), 145.0, 139.6, 137.1 (d, *J* = 3.0 Hz), 128.8 (d, *J* = 8.0 Hz), 127.3, 126.1, 115.8 (d, *J* = 21.5 Hz), 70.3, 25.3.

^{19}F NMR (376 MHz, $CDCl_3$) δ −115.67 (tt, *J* = 8.5, 5.5 Hz).

Data are consistent with those previously reported (compound 5e).[44] *As ^{19}F NMR data have not previously been reported, a copy of the spectrum is included.*

2-Benzyl 1-(*tert*-butyl) (2*S*,4*R*)-4-hydroxypyrrolidine-1,2-dicarboxylate

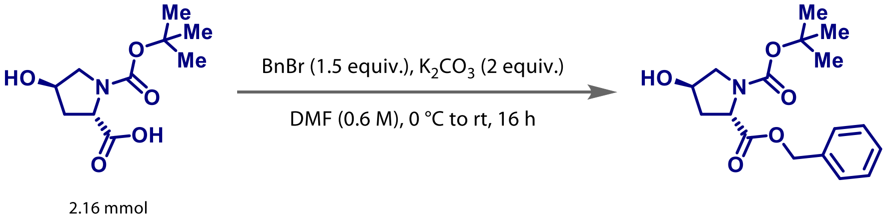

Synthesised following a literature procedure[45] from (2*S*,4*R*)-1-(*tert*-butoxycarbonyl)-4-hydroxypyrrolidine-2-carboxylic acid (500 mg, 2.16 mmol) and isolated by FCC (eluted with 0-56% EtOAc in pentane) as a pale-yellow oil (641 mg, 92%, 2:1 mixture of rotamers *A*:*B*).

^{1}H NMR (400 MHz, $CDCl_3$): δ 7.38 – 7.28 (m, 5H, *A*+*B*), 5.28 – 5.05 (m, 2H, *A*+*B*), 4.53 – 4.39 (m, 2H, *A*+*B*), 3.63 (d, *J* = 4.5 Hz, 0.33H, *B*), 3.60 (d, *J* = 4.5 Hz, 0.67H, *A*), 3.54 (d, *J* = 11.5 Hz, 0.67H, *A*), 3.44 (d, *J* = 11.5 Hz, 0.33H, *B*), 2.52 – 2.15 (m, 2H, *A*+*B*), 2.05 (app ddd, *J* = 13.0, 8.0, 5.0 Hz, 1H, *A*+*B*), 1.45 (s, 3H, *B*), 1.33 (s, 6H, *A*).

^{13}C NMR (101 MHz, $CDCl_3$): δ 173.1 (*A*), 172.8 (*B*), 154.7 (*B*), 154.1 (*A*), 135.8 (*B*), 135.6 (*A*), 128.7 (*A*), 128.6 (*B*), 128.6 (*B*), 128.5 (*A*), 128.3 (*B*), 128.2 (*A*), 80.6 (*A*), 80.4 (*B*), 70.2 (*B*), 69.5 (*A*), 66.9 (*A*+*B*), 58.1 (*A*), 57.8 (*B*), 54.8 (*A*), 53.5 (*B*), 39.3 (*A*), 38.5 (*B*), 28.5 (*B*), 28.3 (*A*).

Data are consistent with those previously reported (compound P-2).[45]

Scope

General Procedure B

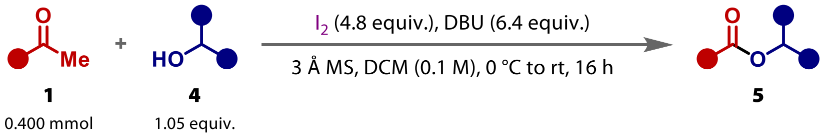

To an oven-dried Schlenk tube containing activated 3 Å molecular sieves (400 mg), which had been evacuated and refilled with nitrogen (×3), was added methyl ketone **1** (0.400 mmol, 1 equiv.), secondary alcohol **4** (0.420 mmol, 1.05 equiv.) and anhydrous DCM (4 mL), followed by addition of iodine (487 mg, 1.92 mmol, 4.8 equiv.) in one portion. The reaction mixture was cooled to 0 °C (ice-water bath), then DBU (382 µL, 2.56 mmol, 6.4 equiv.) was added dropwise. The reaction mixture was stirred for 16 h and allowed to warm to room temperature. The reaction mixture was diluted with EtOAc, filtered, then added to sat. aq. $Na_2S_2O_3$. The mixture was separated and the aqueous layer was extracted a further two times with EtOAc. The combined organic fractions were dried over anhydrous $MgSO_4$, filtered, and concentrated under vacuum. The crude product was purified by FCC (eluted with EtOAc in pentane).

*1-(2-Fluorophenyl)ethyl 4-fluorobenzoate, **5a***

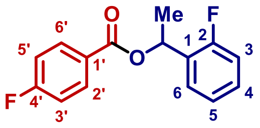

Ester **5a** was synthesised following Procedure B from 4'-fluoroacetophenone **1a** and 1-(2-fluorophenyl)ethanol **4a**, and was isolated as a colourless oil (82.5 mg, 79%).

^{1}H NMR (400 MHz, $CDCl_3$) δ 8.14 – 8.08 (m, 2H, 2'-H, 6'-H), 7.47 (app td, *J* = 7.5, 2.0 Hz, 1H, 6-H), 7.32 – 7.25 (m, 1H, 4-H), 7.18 – 7.04 (m, 4H, 3-H, 5-H, 3'-H, 5'-H), 6.37 (q, *J* = 6.5 Hz, 1H, C*H*Me), 1.69 (d, *J* = 6.5 Hz, 3H, Me).

^{13}C NMR (101 MHz, $CDCl_3$) δ 166.0 (d, *J* = 254.0 Hz, C4'), 164.7 (C=O), 160.0 (d, *J* = 247.5 Hz, C2), 132.3 (d, *J* = 9.0 Hz, C2', C6'), 129.6 (d, *J* = 8.5 Hz, C4), 129.0 (d, *J* = 13.5 Hz, C1), 127.3 (d, *J* = 4.0 Hz, C6), 126.7 (d, *J* = 3.0 Hz, C1'), 124.4 (d, *J* = 3.5 Hz, C5), 115.8 (d, *J* = 21.5 Hz, C3), 115.7 (d, *J* = 22.0 Hz, C3', C5'), 67.9 (d, *J* = 3.0 Hz, CHMe), 21.5 (Me).

^{19}F NMR (376 MHz, $CDCl_3$) δ −105.59 (tt, *J* = 8.5, 5.5 Hz, 1F, 4'-F), −118.21 (ddd, *J* = 10.5, 7.5, 5.0 Hz, 1F, 2-F).

HRMS (EI) m/z: $[M]^+$ Calcd for $C_{15}H_{12}O_2F_2$ 262.0800; found 262.0801; 0.38 ppm error.

IR (neat) ν_{max} / cm^{-1}: 2985, 1717, 1605, 1507, 1492, 1265, 1231, 1153, 1108, 1090, 1063, 853, 756.

*1-(2-Fluorophenyl)ethyl 4-nitrobenzoate, **5b***

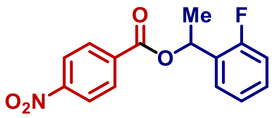

Ester **5b** was synthesised following Procedure B from 4'-nitroacetophenone and 1-(2-fluorophenyl)ethanol **4a**, and was isolated as a pale-yellow solid (105 mg, 91%).

1 mmol scale reaction: To an oven-dried 2-neck round-bottom flask containing activated 3 Å molecular sieves (1.00 g), which had been evacuated and refilled with nitrogen (×3), was added 4'-nitroacetophenone (165 mg, 1.00 mmol, 1 equiv.), 1-(2-fluorophenyl)ethanol **4a** (133 µL, 1.05 mmol, 1.05 equiv.) and anhydrous DCM (10 mL), followed by addition of iodine (1.22 g, 4.80 mmol, 4.8 equiv.) in one portion. The reaction mixture was cooled to 0 °C (ice-water bath), then DBU (956 µL, 6.40 mmol, 6.4 equiv.) was added dropwise. The reaction mixture was stirred for 16 h and allowed to warm to room temperature. Work-up and purification were carried out following Procedure A to obtain the title compound as a pale-yellow solid (238 mg, 82%).

^{1}H NMR (400 MHz, $CDCl_3$) δ 8.31 – 8.22 (m, 4H, Ar-H), 7.48 (app td, *J* = 7.5, 2.0 Hz, 1H, Ar-H), 7.34 – 7.27 (m, 1H, Ar-H), 7.17 (app td, *J* = 7.5, 1.0 Hz, 1H, Ar-H), 7.08 (ddd, *J* = 10.5, 8.5, 1.5 Hz, 1H, Ar-H), 6.40 (q, *J* = 6.5 Hz, 1H, C*H*Me), 1.73 (d, *J* = 6.5 Hz, 3H, Me).

^{13}C NMR (101 MHz, $CDCl_3$) δ 163.8 (C=O), 160.0 (d, *J* = 247.5 Hz, Ar), 150.7 (Ar), 135.8 (Ar), 130.9 (Ar), 129.9 (d, *J* = 8.5 Hz, Ar), 128.3 (d, *J* = 13.5 Hz, Ar), 127.3 (d, *J* = 4.0 Hz, Ar), 124.5 (d, *J* = 3.5 Hz, Ar), 123.6 (Ar), 115.9 (d, *J* = 21.5 Hz, Ar), 68.9 (d, *J* = 3.0 Hz, *C*HMe), 21.2 (Me).

^{19}F NMR (376 MHz, $CDCl_3$) δ −117.92 (app dt, *J* = 11.0, 6.0 Hz).

HRMS (EI) m/z: $[M]^+$ Calcd for $C_{15}H_{12}NO_4F$ 289.0745; found 289.0745; 0.00 ppm error.

IR (neat) ν_{max} / cm^{-1}: 3112, 3076, 2987, 1723, 1523, 1276, 1232, 1106, 760, 720.

*1-(2-Fluorophenyl)ethyl 4-methoxybenzoate, **5c***

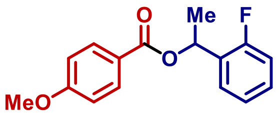

Ester **5c** was synthesised following Procedure B from 4'-methoxyacetophenone and 1-(2-fluorophenyl)ethanol **4a**, and was isolated as a colourless oil (52.3 mg, 48%).

^{1}H NMR (400 MHz, $CDCl_3$) δ 8.08 – 8.02 (m, 2H, Ar-H), 7.48 (app td, *J* = 7.5, 2.0 Hz, 1H, Ar-H), 7.30 – 7.23 (m, 1H, Ar-H), 7.14 (app td, *J* = 7.5, 1.0 Hz, 1H, Ar-H), 7.06 (ddd, *J* = 10.5, 8.0, 1.0 Hz, 1H, Ar-H), 6.96 – 6.90 (m, 2H, Ar-H), 6.36 (q, *J* = 6.5 Hz, 1H, C*H*Me), 3.86 (s, 3H, OMe), 1.67 (d, *J* = 6.5 Hz, 3H, CH*Me*).

^{13}C NMR (101 MHz, $CDCl_3$) δ 165.4 (C=O), 163.6 (Ar), 159.9 (d, *J* = 247.0 Hz, Ar), 131.8 (Ar), 129.5 (Ar), 129.4 (d, *J* = 8.0 Hz, Ar), 127.3 (d, *J* = 4.0 Hz, Ar), 124.4 (d, *J* = 3.5 Hz, Ar), 122.9 (Ar), 115.7 (d, *J* = 21.5 Hz, Ar), 113.8 (Ar), 67.3 (d, *J* = 3.0 Hz, *C*HMe), 55.6 (OMe), 21.6 (CH*Me*).

^{19}F NMR (376 MHz, $CDCl_3$) δ −118.32 (ddd, *J* = 10.5, 7.5, 5.5 Hz).

HRMS (EI) m/z: $[M]^+$ Calcd for $C_{16}H_{15}O_3F$ 274.1000; found 274.0998; 0.73 ppm error.

IR (neat) ν_{max} / cm^{-1}: 2982, 2935, 1709, 1605, 1511, 1251, 1230, 1166, 1098, 1062, 1028, 846, 755.

1-(2-Fluorophenyl)ethyl picolinate, ***5d***

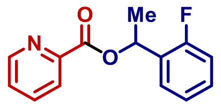

Ester **5d** was synthesised following Procedure B from 2-acetylpyridine and 1-(2-fluorophenyl)ethanol **4a**, and was isolated as a yellow oil (88.2 mg, 90%).

^{1}H NMR (400 MHz, CDCl$_3$) δ 8.77 (ddd, *J* = 4.5, 2.0, 1.0 Hz, 1H, Ar-H), 8.12 (app dt, *J* = 8.0, 1.0 Hz, 1H, Ar-H), 7.81 (app td, *J* = 8.0, 2.0 Hz, 1H, Ar-H), 7.53 (app td, *J* = 7.5, 2.0 Hz, 1H, Ar-H), 7.45 (ddd, *J* = 7.5, 4.5, 1.0 Hz, 1H, Ar-H), 7.29 – 7.22 (m, 1H, Ar-H), 7.12 (app td, *J* = 7.5, 1.0 Hz, 1H, Ar-H), 7.04 (ddd, *J* = 10.5, 8.0, 1.0 Hz, 1H, Ar-H), 6.45 (q, *J* = 6.5 Hz, 1H, C*H*Me), 1.73 (d, *J* = 6.5 Hz, 3H, Me).

^{13}C NMR (101 MHz, CDCl$_3$) δ 164.3 (C=O), 159.9 (d, *J* = 247.5 Hz, Ar), 150.1 (Ar), 148.2 (Ar), 137.0 (Ar), 129.6 (d, *J* = 8.5 Hz, Ar), 128.7 (d, *J* = 13.5 Hz, Ar), 127.3 (d, *J* = 4.0 Hz, Ar), 127.0 (Ar), 125.4 (Ar), 124.4 (d, *J* = 3.5 Hz, Ar), 115.7 (d, *J* = 21.5 Hz, Ar), 68.3 (d, *J* = 3.0 Hz, *C*HMe), 21.3 (Me).

^{19}F NMR (376 MHz, CDCl$_3$) δ −118.07 (app dt, *J* = 11.0, 6.0 Hz).

HRMS (EI) m/z: [M]$^+$ Calcd for $C_{14}H_{12}NO_2F$ 245.0847; found 245.0846; 0.41 ppm error.

IR (neat) ν_{max} / cm^{-1}: 3058, 2986, 1717, 1492, 1302, 1278, 1244, 1230, 1131, 1063, 994, 745, 705.

1-(2-Fluorophenyl)ethyl 5-cyano-1-methyl-1H-pyrrole-2-carboxylate, ***5e***

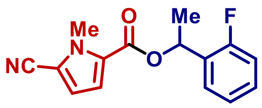

Ester **5e** was synthesised following Procedure B from 5-acetyl-1-methyl-1*H*-pyrrole-2-carbonitrile and 1-(2-fluorophenyl)ethanol **4a**, and was isolated as a white solid (50.9 mg, 47%).

^{1}H NMR (400 MHz, CDCl$_3$) δ 7.45 – 7.38 (m, 2H, Ar-H), 7.30 – 7.22 (m, 2H, Ar-H), 7.13 (app td, *J* = 7.5, 1.0 Hz, 1H, Ar-H), 7.04 (ddd, *J* = 10.5, 8.0, 1.0 Hz, 1H, Ar-H), 6.28 (q, *J* = 6.5 Hz, 1H, C*H*Me), 3.80 (s, 3H, NMe), 1.62 (d, *J* = 6.5 Hz, 3H, CH*Me*).

^{13}C NMR (101 MHz, CDCl$_3$) δ 161.9 (C=O), 159.9 (d, *J* = 247.5 Hz, Ar), 131.3 (Ar), 129.5 (d, *J* = 8.0 Hz, Ar), 129.0 (d, *J* = 13.5 Hz, Ar), 127.2 (d, *J* = 4.0 Hz, Ar), 124.4 (d, *J* = 3.5 Hz, Ar), 121.2 (Ar), 117.2 (Ar), 115.8 (d, *J* = 21.5 Hz, Ar), 112.5 (CN), 106.1 (Ar), 67.0 (d, *J* = 3.0 Hz, *C*HMe), 36.0 (NMe), 21.4 (CH*Me*).

^{19}F NMR (376 MHz, CDCl$_3$) δ −118.17 (app dt, *J* = 11.0, 6.0 Hz).

HRMS (EI) m/z: [M]$^+$ Calcd for $C_{15}H_{13}N_2O_2F$ 272.0956; found 272.0955; 0.37 ppm error.

IR (neat) ν_{max} / cm^{-1}: 2961, 2901, 2224, 1711, 1551, 1492, 1223, 1192, 1065, 981, 909, 760, 731.

*1-(2-Fluorophenyl)ethyl 3-methylbenzoate, **5f***

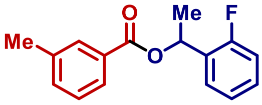

Ester **5f** was synthesised following Procedure B from 3'-methylacetophenone and 1-(2-fluorophenyl)ethanol **4a**, and was isolated as a colourless oil (43.0 mg, 42%).

^{1}H NMR (400 MHz, CDCl$_3$) δ 7.92 – 7.88 (m, 2H, Ar-H), 7.49 (app td, *J* = 7.5, 2.0 Hz, 1H, Ar-H), 7.40 – 7.24 (m, 3H, Ar-H), 7.15 (app td, *J* = 7.5, 1.0 Hz, 1H, Ar-H), 7.07 (ddd, *J* = 10.5, 8.0, 1.0 Hz, 1H, Ar-H), 6.39 (q, *J* = 6.5 Hz, 1H, C*H*Me), 2.41 (s, 3H, Ar-Me), 1.69 (d, *J* = 6.5 Hz, 3H, CH*Me*).

^{13}C NMR (101 MHz, CDCl$_3$) δ 165.9 (C=O), 159.9 (d, *J* = 247.5 Hz, Ar), 138.3 (Ar), 133.9 (Ar), 130.4 (Ar), 130.3 (Ar), 129.5 (d, *J* = 8.0 Hz, Ar), 129.2 (d, *J* = 13.5 Hz, Ar), 128.4 (Ar), 127.3 (d, *J* = 4.0 Hz, Ar), 126.9 (Ar), 124.4 (d, *J* = 3.5 Hz, Ar), 115.8 (d, *J* = 21.5 Hz, Ar), 67.5 (d, *J* = 3.0 Hz, *C*HMe), 21.6 (Ar-*Me*), 21.4 (CH*Me*).

^{19}F NMR (376 MHz, CDCl$_3$) δ −118.17 (app dt, *J* = 11.0, 6.0 Hz).

HRMS (EI) m/z: $[M]^+$ Calcd for $C_{16}H_{15}O_2F$ 258.1051; found 258.1051; 0.00 ppm error.

IR (neat) ν_{max} / cm^{-1}: 2984, 1717, 1492, 1271, 1232, 1195, 1106, 1064, 757, 742.

*1-(4-Nitrophenyl)ethyl 4-nitrobenzoate, **5g***

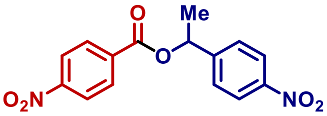

Ester **5g** was synthesised following Procedure B from 4'-nitroacetophenone and 1-(4-nitrophenyl)ethanol, and was isolated as a white solid (111 mg, 88%).

^{1}H NMR (400 MHz, CDCl$_3$) δ 8.32 – 8.27 (m, 2H, Ar-H), 8.26 – 8.21 (m, 4H, Ar-H), 7.64 – 7.58 (m, 2H, Ar-H), 6.20 (q, *J* = 6.5 Hz, 1H, C*H*Me), 1.74 (d, *J* = 6.5 Hz, 3H, Me).

^{13}C NMR (101 MHz, CDCl$_3$) δ 163.9 (C=O), 150.9 (Ar), 148.3 (Ar), 147.8 (Ar), 135.3 (Ar), 130.9 (Ar), 127.0 (Ar), 124.2 (Ar), 123.8 (Ar), 73.1 (*C*HMe), 22.3 (Me).

HRMS (MALDI) m/z: $[M]^-$ Calcd for $C_{15}H_{12}N_2O_6$ 316.0701; found 316.0705; 1.27 ppm error.

IR (neat) ν_{max} / cm^{-1}: 3112, 2986, 1722, 1519, 1344, 1264, 1101, 1060, 1013, 854, 841, 718, 697.

1-(4-Methylphenyl)ethyl 4-nitrobenzoate, **5h**

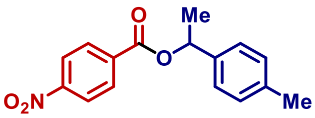

Ester **5h** was synthesised following Procedure B from 4'-nitroacetophenone and 1-(4-methylphenyl)ethanol, and was isolated as a white solid (71.7 mg, 63%).

^{1}H NMR (400 MHz, CDCl$_3$) δ 8.28 (d, *J* = 9.0 Hz, 2H, Ar-H), 8.23 (d, *J* = 9.0 Hz, 2H, Ar-H), 7.35 (d, *J* = 8.0 Hz, 2H, Ar-H), 7.20 (d, *J* = 8.0 Hz, 2H, Ar-H), 6.13 (q, *J* = 6.5 Hz, 1H, C*H*Me), 2.36 (s, 3H, Ar-Me), 1.70 (d, *J* = 6.5 Hz, 3H, CH*Me*).

^{13}C NMR (101 MHz, CDCl$_3$) δ 164.1 (C=O), 150.6 (Ar), 138.3 (Ar), 138.1 (Ar), 136.1 (Ar), 130.9 (Ar), 129.5 (Ar), 126.3 (Ar), 123.6 (Ar), 74.3 (*C*HMe), 22.2 (CH*Me*), 21.3 (Ar-*Me*).

HRMS (EI) m/z: $[M]^+$ Calcd for $C_{16}H_{15}NO_4$ 285.0996; found 285.0995; 0.35 ppm error.

IR (neat) ν_{max} / cm^{-1}: 2982, 1719, 1525, 1266, 1115, 1101, 1056, 1014, 815, 717.

Only 11 of the 12 ^{13}C signals were reported in the only previous report and ^{1}H signal integrals were not reported at all (compound 1),[46] *so **5h** was fully characterised. Copies of NMR spectra are included.*

*1-(2-Methylphenyl)ethyl 4-nitrobenzoate, **5i***

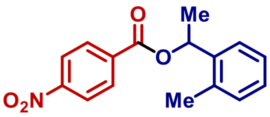

Ester **5i** was synthesised following Procedure B from 4'-nitroacetophenone and 1-(2-methylphenyl)ethanol, and was isolated as a white solid (96.1 mg, 84%).

^{1}H NMR (400 MHz, CDCl$_3$) δ 8.31 – 8.27 (m, 2H, Ar-H), 8.26 – 8.22 (m, 2H, Ar-H), 7.52 – 7.46 (m, 1H, Ar-H), 7.28 – 7.17 (m, 3H, Ar-H), 6.36 (q, *J* = 6.5 Hz, 1H, C*H*Me), 2.46 (s, 3H, Ar-Me), 1.69 (d, *J* = 6.5 Hz, 3H, CH*Me*).

^{13}C NMR (101 MHz, CDCl$_3$) δ 164.1 (C=O), 150.7 (Ar), 139.5 (Ar), 136.0 (Ar), 134.9 (Ar), 130.8 (Ar), 130.7 (Ar), 128.1 (Ar), 126.6 (Ar), 125.4 (Ar), 123.7 (Ar), 71.3 (*C*HMe), 21.6 (CH*Me*), 19.2 (Ar-*Me*).

HRMS (MALDI) m/z: $[M]^-$ Calcd for $C_{16}H_{15}NO_4$ 285.1007; found 285.1004; 1.05 ppm error.

IR (neat) ν_{max} / cm^{-1}: 2981, 1719, 1524, 1268, 1115, 1101, 1062, 1049, 872, 841, 760, 717.

*1-(2,6-Dimethylphenyl)ethyl 4-nitrobenzoate, **5j***

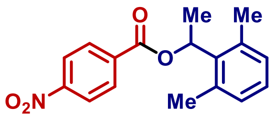

Ester **5j** was synthesised following Procedure B from 4'-nitroacetophenone and 1-(2,6-dimethylphenyl)ethanol, and was isolated as a white solid (34.4 mg, 29%).

^{1}H NMR (400 MHz, $CDCl_3$) δ 8.32 – 8.27 (m, 2H, Ar-H), 8.25 – 8.20 (m, 2H, Ar-H), 7.09 (dd, *J* = 8.5, 6.5 Hz, 1H, Ar-H), 7.03 (d, *J* = 7.5 Hz, 2H, Ar-H), 6.58 (q, *J* = 7.0 Hz, 1H, C*H*Me), 2.55 (s, 6H, Ar-Me), 1.75 (d, *J* = 7.0 Hz, 3H, CH*Me*).

^{13}C NMR (101 MHz, $CDCl_3$) δ 164.1 (C=O), 150.6 (Ar), 136.7 (Ar), 136.0 (Ar), 136.0 (Ar), 130.8 (Ar), 129.5 (Ar), 127.9 (Ar), 123.7 (Ar), 71.7 (CHMe), 20.8 (Ar-*Me*), 19.7 (CH*Me*).

HRMS (MALDI) m/z: $[M]^-$ Calcd for $C_{17}H_{17}NO_4$ 299.1163; found 299.1169; 2.01 ppm error.

IR (neat) ν_{max} / cm^{-1}: 2977, 1718, 1524, 1347, 1271, 1101, 1057, 1014, 872, 841, 772, 718.

Benzhydryl 4-nitrobenzoate, **5k**

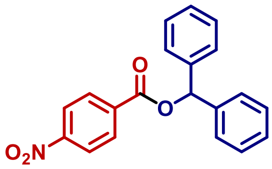

Ester **5k** was synthesised following Procedure B from 4'-nitroacetophenone and benzhydrol, and was isolated as a white solid (77.7 mg, 58%).

^{1}H NMR (400 MHz, $CDCl_3$) δ 8.31 (app s, 4H), 7.48 – 7.43 (m, 4H), 7.42 – 7.37 (m, 4H), 7.37 – 7.31 (m, 2H), 7.17 (s, 1H).

^{13}C NMR (101 MHz, $CDCl_3$) δ 163.9, 150.7, 139.6, 135.7, 131.0, 128.8, 128.4, 127.2, 123.7, 78.6.

Data are consistent with those previously reported (compound 5g).[47]

*1-(4'-Fluoro-[1,1'-biphenyl]-4-yl)ethyl 4-nitrobenzoate, **5l***

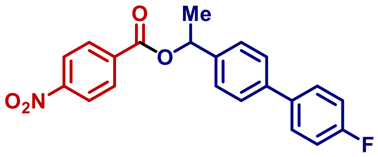

Ester **5l** was synthesised following Procedure B from 4'-nitroacetophenone and 1-(4'-fluoro-[1,1'-biphenyl]-4-yl)ethanol, and was isolated as a white solid (92.4 mg, 66%).

^{1}H NMR (400 MHz, $CDCl_3$) δ 8.32 – 8.23 (m, 4H, Ar-H), 7.59 – 7.50 (m, 6H, Ar-H), 7.16 – 7.09 (m, 2H, Ar-H), 6.21 (q, *J* = 6.5 Hz, 1H, C*H*Me), 1.76 (d, *J* = 6.5 Hz, 3H, Me).

^{13}C NMR (101 MHz, $CDCl_3$) δ 164.1 (C=O), 162.6 (d, *J* = 247.0 Hz, Ar), 150.6 (Ar), 140.4 (Ar), 140.1 (Ar), 136.8 (d, *J* = 3.5 Hz, Ar), 135.9 (Ar), 130.9 (Ar), 128.8 (d, *J* = 8.0 Hz, Ar), 127.4 (Ar), 126.8 (Ar), 123.6 (Ar), 115.8 (d, *J* = 21.5 Hz, Ar), 74.0 (*C*HMe), 22.2 (Me).

^{19}F NMR (376 MHz, $CDCl_3$) δ −115.15 (tt, *J* = 8.5, 5.5 Hz).

HRMS (MALDI) m/z: $[M]^-$ Calcd for $C_{21}H_{16}NO_4F$ 365.1063; found 365.1068; 1.37 ppm error.

IR (neat) ν_{max} / cm^{-1}: 2984, 1721, 1527, 1498, 1271, 1103, 908, 823, 732.

*Chroman-4-yl 4-nitrobenzoate, **5m***

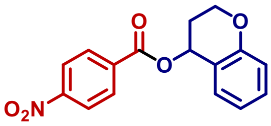

Ester **5m** was synthesised following Procedure B from 4'-nitroacetophenone and chroman-4-ol, and was isolated as a white solid (59.6 mg, 50%).

^{1}H NMR (400 MHz, $CDCl_3$) δ 8.29 – 8.24 (m, 2H, Ar-H), 8.23 – 8.19 (m, 2H, Ar-H), 7.36 (dd, *J* = 7.5, 1.5 Hz, 1H, Ar-H), 7.27 (ddd, *J* = 9.0, 7.5, 2.0 Hz, 1H, Ar-H), 6.97 – 6.88 (m, 2H, Ar-H), 6.23 (app t, *J* = 4.0 Hz, 1H, C*H*CH$_2$), 4.44 – 4.31 (m, 2H, CH$_2$O), 2.37 (app ddt, *J* = 15.5, 11.0, 4.5 Hz, 1H, CHC*H*H), 2.26 (app dq, *J* = 14.5, 3.5 Hz, 1H, CHCH*H*).

^{13}C NMR (101 MHz, $CDCl_3$) δ 164.2 (C=O), 155.5 (Ar), 150.7 (Ar), 135.7 (Ar), 130.9 (Ar), 130.9 (Ar), 130.7 (Ar), 123.7 (Ar), 120.8 (Ar), 119.6 (Ar), 117.4 (Ar), 67.2 (*C*HCH$_2$), 62.2 (CH$_2$O), 28.5 (CH*C*H$_2$).

HRMS (MALDI) m/z: $[M]^-$ Calcd for $C_{16}H_{13}NO_5$ 299.0794; found 299.0798; 1.34 ppm error.

IR (neat) ν_{max} / cm^{-1}: 2970, 1715, 1608, 1523, 1487, 1345, 1263, 1228, 1103, 1057, 865, 758, 719.

Cyclohexyl 4-nitrobenzoate, **5n**

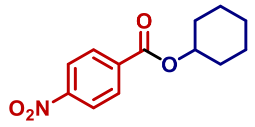

Ester **5n** was synthesised following Procedure B from 4'-nitroacetophenone and cyclohexanol, and was isolated as a pale-yellow oil (83.0 mg, 83%).

^{1}H NMR (400 MHz, CDCl$_3$) δ 8.30 – 8.25 (m, 2H), 8.23 – 8.18 (m, 2H), 5.06 (tt, *J* = 9.0, 4.0 Hz, 1H), 2.02 – 1.92 (m, 2H), 1.85 – 1.75 (m, 2H), 1.66 – 1.55 (m, 3H), 1.52 – 1.41 (m, 2H), 1.41 – 1.30 (m, 1H).

^{13}C NMR (101 MHz, CDCl$_3$) δ 164.2, 150.6, 136.5, 130.8, 123.6, 74.5, 31.7, 25.5, 23.8.

Data are consistent with those previously reported (compound 6).[48]

(−)-Menthyl 4-nitrobenzoate, **5o**

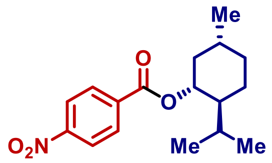

Ester **5o** was synthesised following Procedure B from 4'-nitroacetophenone and (−)-menthol, and was isolated as a yellow solid (66.3 mg, 54%).

^{1}H NMR (400 MHz, CDCl$_3$) δ 8.30 – 8.25 (m, 2H), 8.22 – 8.17 (m, 2H), 4.96 (app td, *J* = 11.0, 4.5 Hz, 1H), 2.16 – 2.08 (m, 1H), 1.91 (app septd, *J* = 7.0, 3.0 Hz, 1H), 1.78 – 1.69 (m, 2H), 1.62 – 1.51 (m, 2H), 1.20 – 1.06 (m, 2H), 0.99 – 0.87 (m, 7H), 0.79 (d, *J* = 7.0 Hz, 3H).

^{13}C NMR (101 MHz, CDCl$_3$) δ 164.3, 150.5, 136.3, 130.8, 123.6, 76.2, 47.3, 40.9, 34.3, 31.6, 26.7, 23.7, 22.1, 20.8, 16.6.
Optical Rotation: $[\alpha]_D^{22}$ −34 (*c* 1.0, CHCl$_3$).

Data are consistent with those previously reported (compound 4).[49]

2-Benzyl 1-(tert-butyl) (2S,4R)-4-((4-nitrobenzoyl)oxy)pyrrolidine-1,2-dicarboxylate, ***5p***

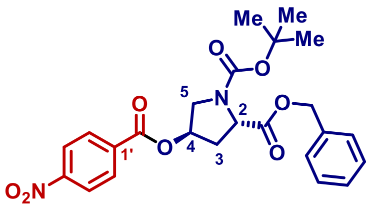

Ester **5p** was synthesised following Procedure B from 4'-nitroacetophenone and 2-benzyl 1-(*tert*-butyl) (2*S*,4*R*)-4-hydroxypyrrolidine-1,2-dicarboxylate, and was isolated as a white solid (143 mg, 76%, 3:2 mixture of rotamers *A*:*B*- calculated from C2-H signals, as these were clearly resolved).

^{1}H NMR (400 MHz, CDCl$_3$): *cf.* diastereomer[50]; δ 8.29 (d, *J* = 9.0 Hz, 2H, Ar-H, *A*+*B*), 8.17 (d, *J* = 9.0 Hz, 2H, Ar-H, *A*+*B*), 7.41 – 7.30 (m, 5H, Ar-H, *A*+*B*), 5.58 – 5.52 (m, 1H, 4-H, *A*+*B*), 5.32 – 5.10 (m, 2H, C*H*$_2$Ph, *A*+*B*), 4.59 (app t, *J* = 8.0 Hz, 0.4H, 2-H, *B*), 4.48 (app t, *J* = 8.0 Hz, 0.6H, 2-H, *A*), 3.91 – 3.81 (m, 1.2H, 5-H, *A*), 3.71 (app d, *J* = 12.5 Hz, 0.8H, 5-H, *B*), 2.63 – 2.50 (m, 1H, 3-H, *A*+*B*), 2.41 – 2.28 (m, 1H, 3-H, *A*+*B*), 1.46 (s, 3.6H, Me, *B*), 1.37 (s, 5.4H, Me, *A*).

^{13}C NMR (101 MHz, CDCl$_3$): δ 172.3 (2-C=O, *A*), 171.9 (2-C=O, *B*), 164.1 (1'-C=O, *B*), 164.0 (1'-C=O, *A*), 154.2 (N-C=O, *B*), 153.7 (N-C=O, *A*), 150.8 (Ar, *B*), 150.7 (Ar, *A*), 135.5 (Ar, *B*), 135.3 (Ar, *A*), 135.0 (Ar, *A*), 134.9 (Ar, *B*), 130.9 (Ar, *A*+*B*), 128.7 (Ar, *A*), 128.6 (Ar, *B*), 128.6 (Ar, *B*), 128.5 (Ar, *A*), 128.4 (Ar, *B*), 128.2 (Ar, *A*), 123.7 (Ar, *B*), 123.6 (Ar, *A*), 80.9 (CMe$_3$, *A*), 80.8 (CMe$_3$, *B*), 74.4 (C4, *B*), 73.7 (C4, *A*), 67.1 (CH$_2$Ph, *A*+*B*), 58.0 (C2, *A*), 57.7 (C2, *B*), 52.3 (C5, *B*), 52.0 (C5, *A*), 36.6 (C3, *A*), 35.6 (C3, *B*), 28.4 (Me, *B*), 28.2 (Me, *A*).

HRMS (ESI) m/z: $[M+H]^+$ Calcd for $C_{24}H_{27}N_2O_8$ 471.1767; found 471.1765; 0.42 ppm error.

IR (neat) ν_{max} / cm^{-1}: 2976, 2901, 1726, 1698, 1528, 1398, 1270, 1157, 1115, 1102, 732, 720.

Optical Rotation: $[\alpha]_D^{22}$ −13 (*c* 1.0, CHCl$_3$).

(−)-Bornyl 4-nitrobenzoate, **5q**

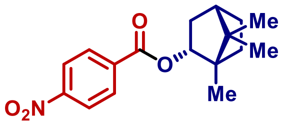

Ester **5q** was synthesised following Procedure B from 4'-nitroacetophenone and (−)-borneol, and was isolated as a white solid (23.6 mg, 19%).

^{1}H NMR (400 MHz, CDCl$_3$) δ 8.32 – 8.27 (m, 2H), 8.24 – 8.19 (m, 2H), 5.15 (ddd, *J* = 10.0, 3.5, 2.0 Hz, 1H), 2.55 – 2.45 (m, 1H), 2.08 (ddd, *J* = 13.5, 9.5, 4.5 Hz, 1H), 1.88 – 1.78 (m, 1H), 1.77 (app t, *J* = 4.5 Hz, 1H), 1.49 – 1.39 (m, 1H), 1.32 (ddd, *J* = 12.0, 9.5, 4.5 Hz, 1H), 1.13 (dd, *J* = 14.0, 3.5 Hz, 1H), 0.97 (s, 3H), 0.93 (s, 3H), 0.92 (s, 3H).

^{13}C NMR (101 MHz, CDCl$_3$) δ 165.1, 150.6, 136.4, 130.7, 123.7, 82.0, 49.3, 48.1, 45.0, 37.0, 28.2, 27.5, 19.8, 19.0, 13.8.

Optical Rotation: $[\alpha]_D^{22}$ −16 (*c* 0.5, CHCl$_3$).

Data are consistent with those previously reported (compound 11e).[51]

Cholesteryl 4-nitrobenzoate, **5r**

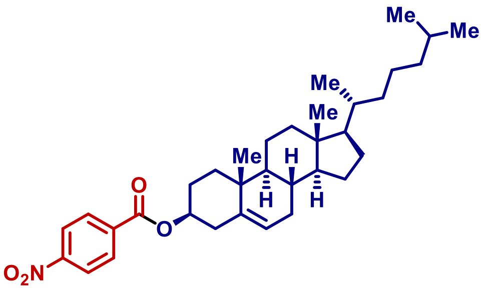

Ester **5r** was synthesised following Procedure B from 4'-nitroacetophenone and cholesterol, and was isolated as a white solid (93.2 mg, 44%).

^{1}H NMR (400 MHz, $CDCl_3$) δ 8.30 – 8.25 (m, 2H), 8.22 – 8.18 (m, 2H), 5.43 (app d, J = 4.5 Hz, 1H), 4.94 – 4.83 (m, 1H), 2.51 – 2.44 (m, 2H), 2.06 – 1.70 (m, 6H), 1.65 – 0.95 (m, 23H), 0.92 (d, J = 6.5 Hz, 3H), 0.87 (d, J = 2.0 Hz, 3H), 0.85 (d, J = 2.0 Hz, 3H), 0.68 (s, 3H).

^{13}C NMR (101 MHz, $CDCl_3$) δ 164.2, 150.5, 139.4, 136.3, 130.8, 123.6, 123.3, 75.9, 56.8, 56.2, 50.1, 42.4, 39.8, 39.6, 38.2, 37.1, 36.7, 36.2, 35.9, 32.0, 32.0, 28.4, 28.1, 27.9, 24.4, 24.0, 23.0, 22.7, 21.2, 19.5, 18.8, 12.0.

Optical Rotation: $[\alpha]_D^{22}$ +5 (c 0.2, $CHCl_3$).

Data are consistent with those previously reported (compound 3m).[52]

O-(4-Nitrobenzoyl)-D-pantolactone, ***5s***

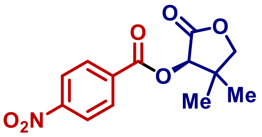

Ester **5s** was synthesised following Procedure B from 4'-nitroacetophenone and D-pantolactone, and was isolated as a white solid (79.7 mg, 71%).

^{1}H NMR (400 MHz, $CDCl_3$) δ 8.33 – 8.28 (m, 2H, Ar-H), 8.27 – 8.22 (m, 2H, Ar-H), 5.63 (s, 1H, CH), 4.14 (app s, 2H, CH_2), 1.29 (s, 3H, Me), 1.23 (s, 3H, Me).

^{13}C NMR (101 MHz, $CDCl_3$) δ 171.9 (CH$\underline{C}$=O), 163.7 (Ar$\underline{C}$=O), 151.0 (Ar), 134.2 (Ar), 131.3 (Ar), 123.8 (Ar), 76.4 (CH), 76.3 (CH_2), 40.6 ($\underline{C}Me_2$), 23.1 (Me), 20.1 (Me).

HRMS (ESI) m/z: $[M]^+$ Calcd for $C_{13}H_{13}NO_6$ 279.0743; found 279.0750; 2.51 ppm error.

IR (neat) v_{max} / cm^{-1}: 2970, 1789, 1735, 1527, 1266, 1120, 1105, 717.

Optical Rotation: $[\alpha]_D^{22}$ +2 (c 1.0, $CHCl_3$).

Unsuccessful Couplings

Unless stated otherwise, products were not observed, despite consumption of the methyl ketone (and in some cases also the alcohol) coupling partner.

Methyl ketones with other enolisable positions:

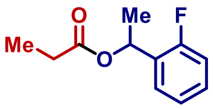
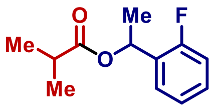
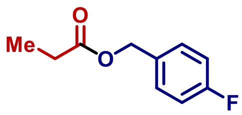
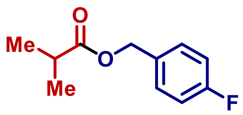
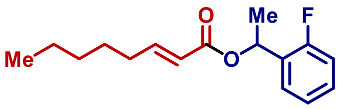

Significantly sterically-hindered methyl ketones:

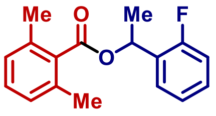
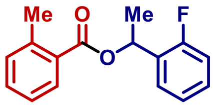
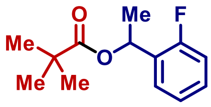
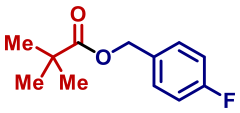

Other incompatible methyl ketones:

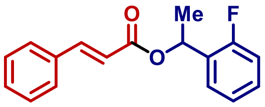

some product formed, but inseparable from side-products

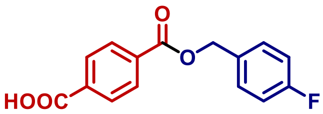

Significantly sterically-hindered alcohols:

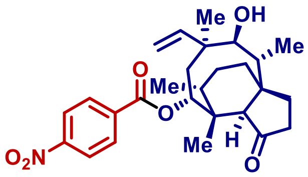

from (+)-mutilin

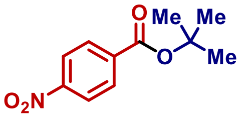
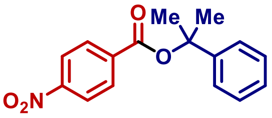
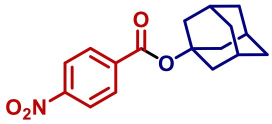

Alcohols bearing electron-rich aromatic rings:

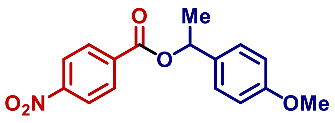

some product formed, but inseparable from ring-iodinated side-products

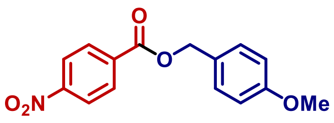

some product formed, but inseparable from ring-iodinated side-products

Other incompatible alcohols:

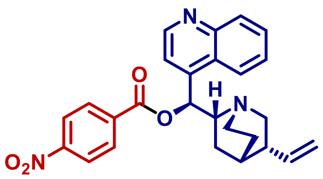

from (+)-cinchonine

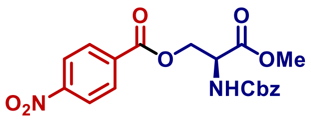

from protected L-serine

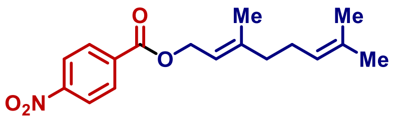

from geraniol

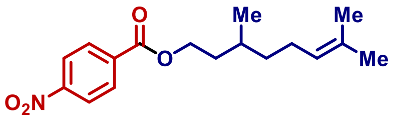

from citronellol
some product formed, but inseparable from side-products

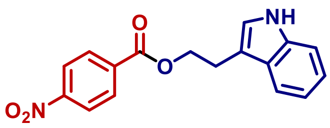

NMR Spectra of Novel Compounds

Spectra are also included for known compounds where ^{19}F NMR data have not previously been reported (^{19}F NMR spectra only included) and for compounds where the NMR data reported here are not fully consistent with those in the only previous report. See characterisation data for further details.

2-(4-Chlorophenoxy)ethyl 4-nitrobenzoate, ***3f***

^{1}H NMR (400 MHz, $CDCl_3$)

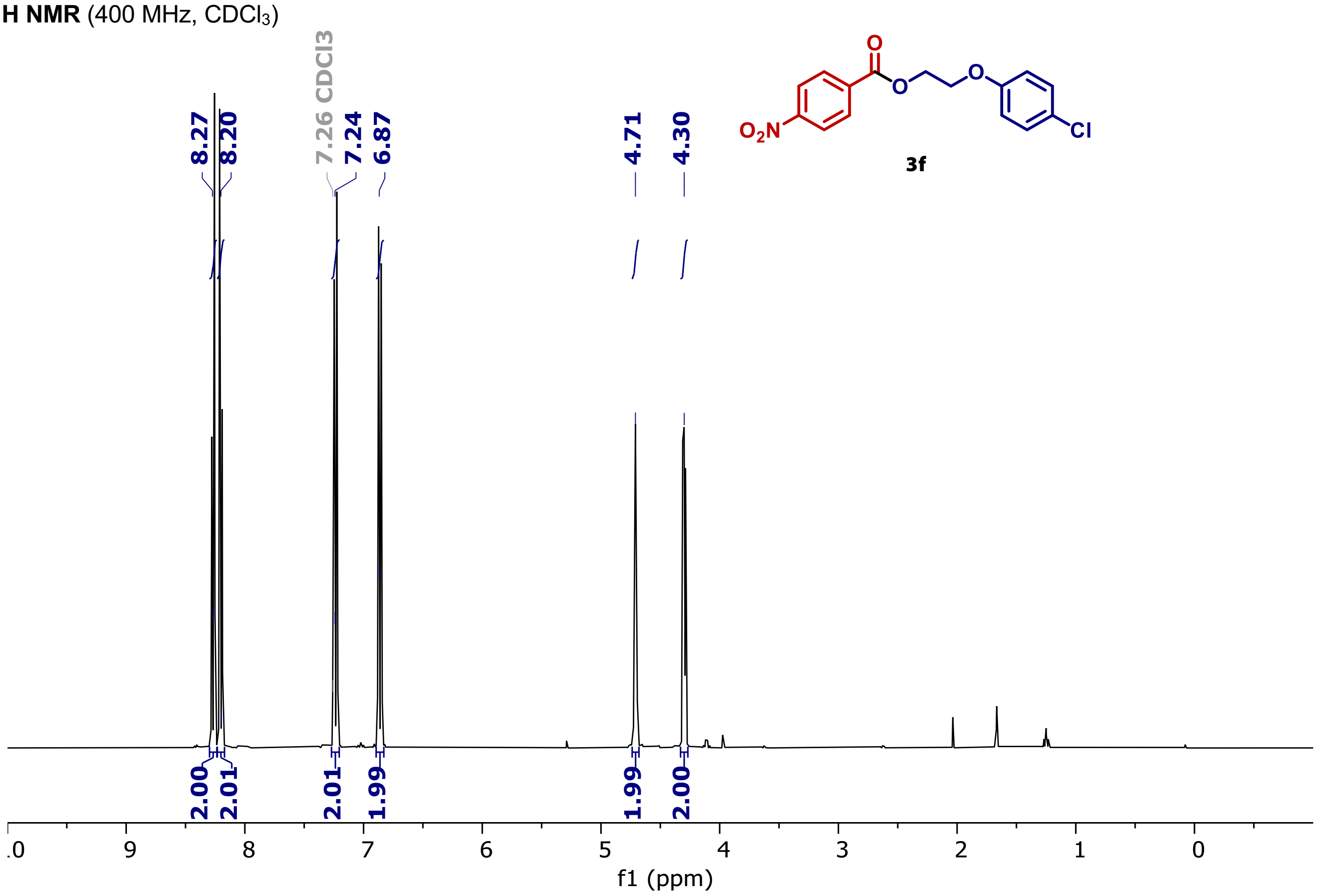

^{13}C NMR (101 MHz, $CDCl_3$)

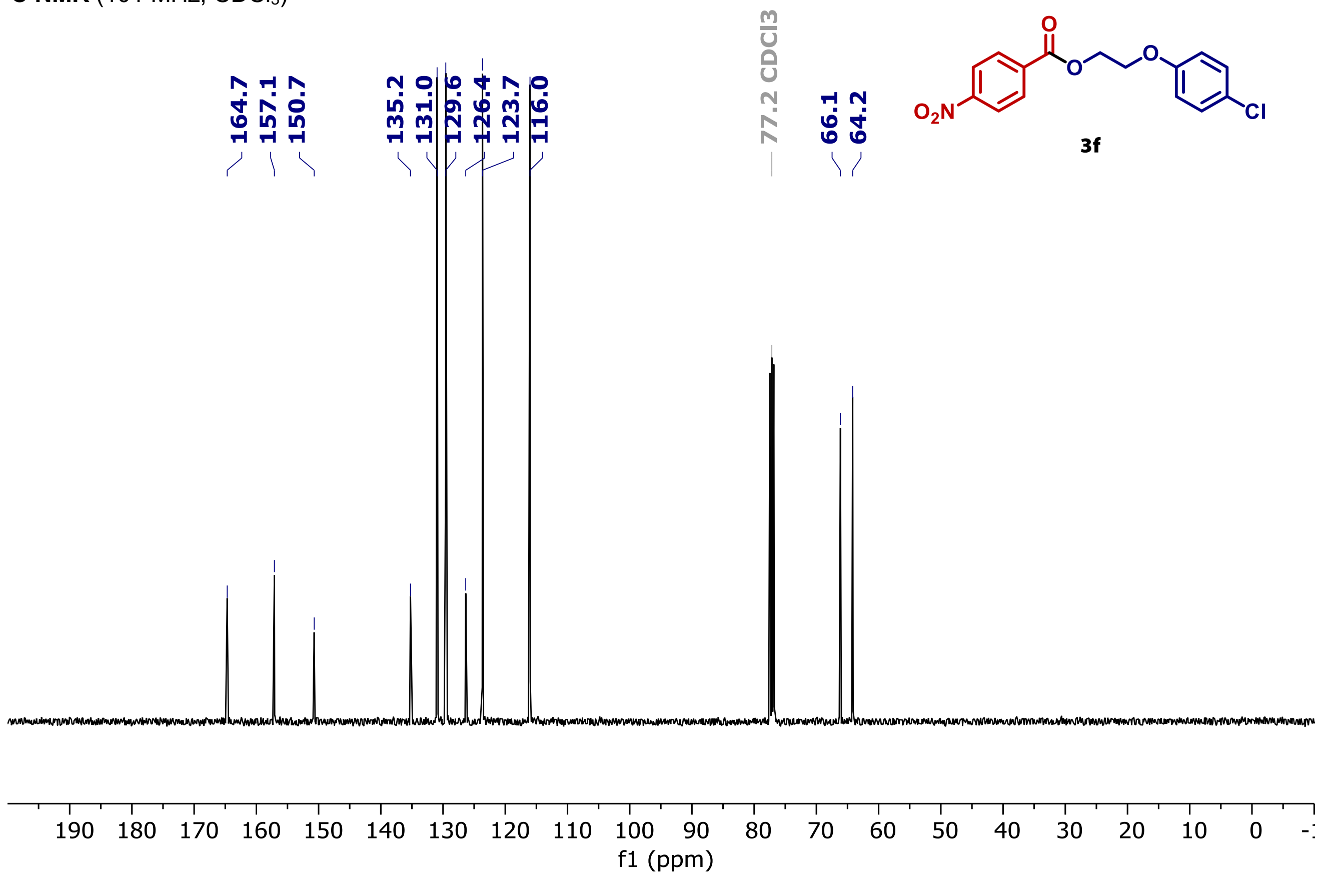

(E)-Hex-3-en-1-yl 4-nitrobenzoate, ***3g***

^{1}H NMR (400 MHz, $CDCl_3$)

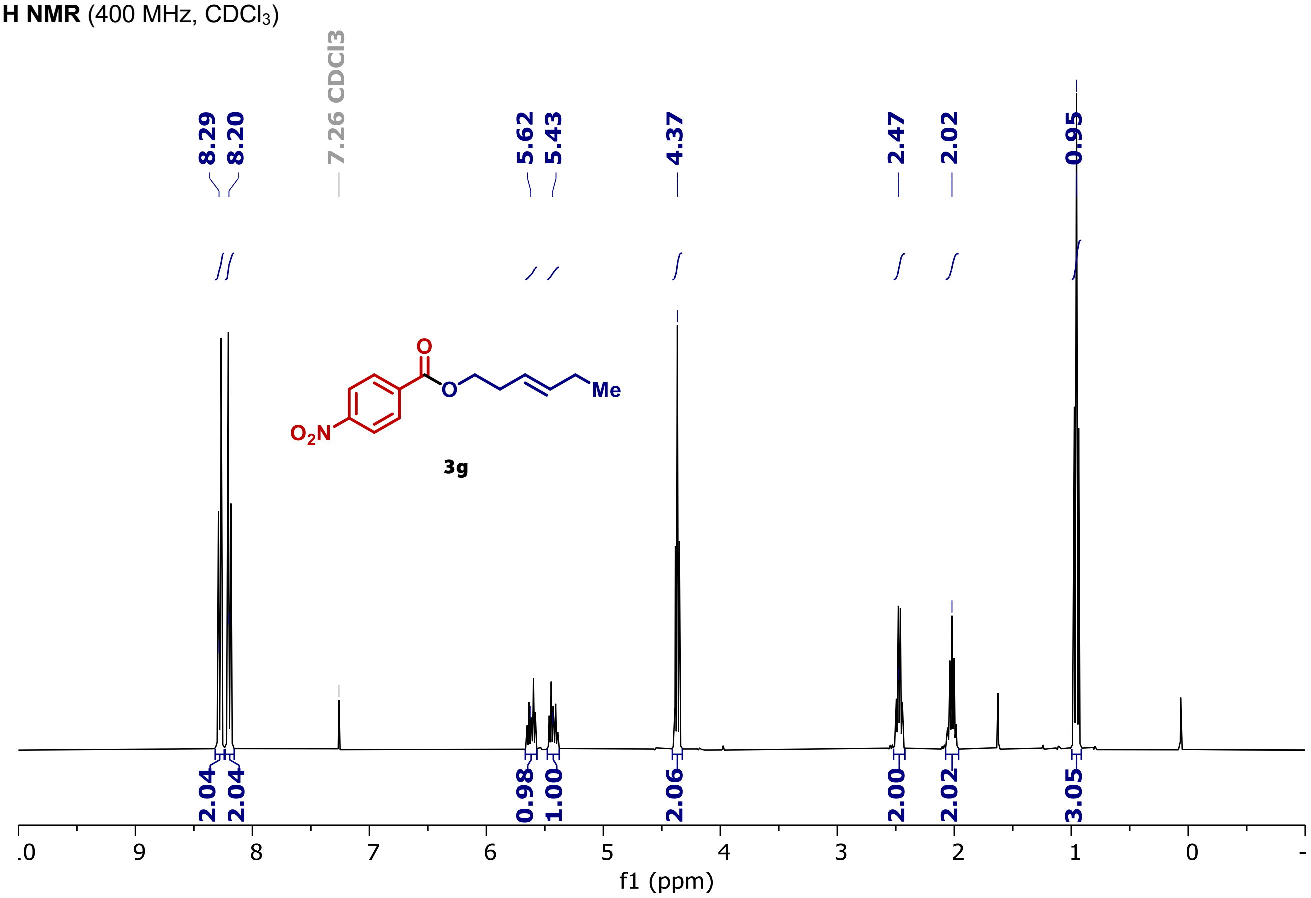

^{13}C NMR (101 MHz, $CDCl_3$)

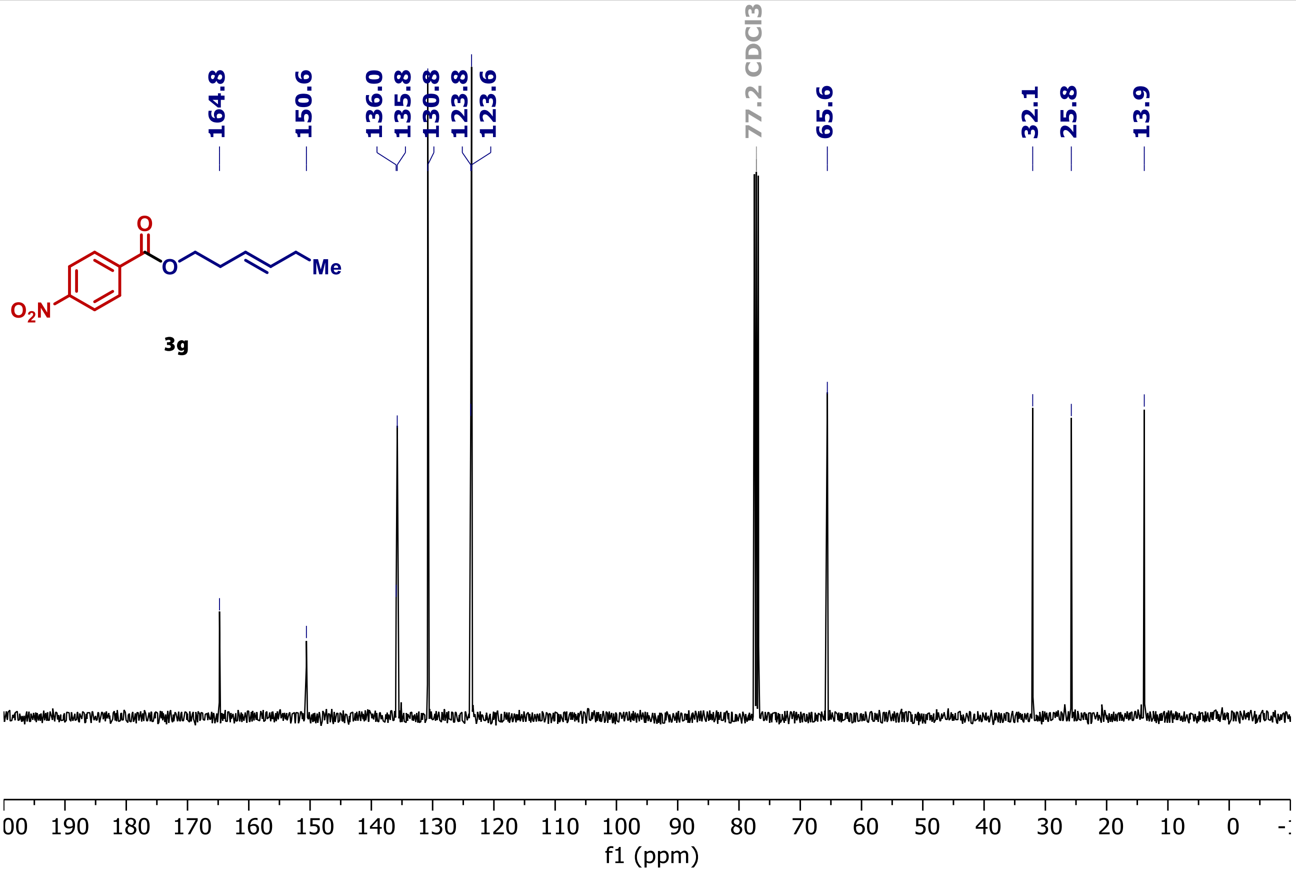
164.8
150.6
136.0
135.8
130.8
123.8
123.6
77.2 CDCl3
65.6
32.1
25.8
13.9
O
O
O2N
Me
3g
00
190
180
170
160
150
140
130
120
110
100
90
80
70
60
50
40
30
20
10
0
f1 (ppm)

2,2,2-Trichloro-4'-fluoroacetophenone, **6a**

^{19}F NMR (376 MHz, $CDCl_3$)

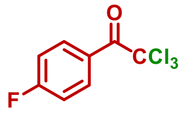

6a

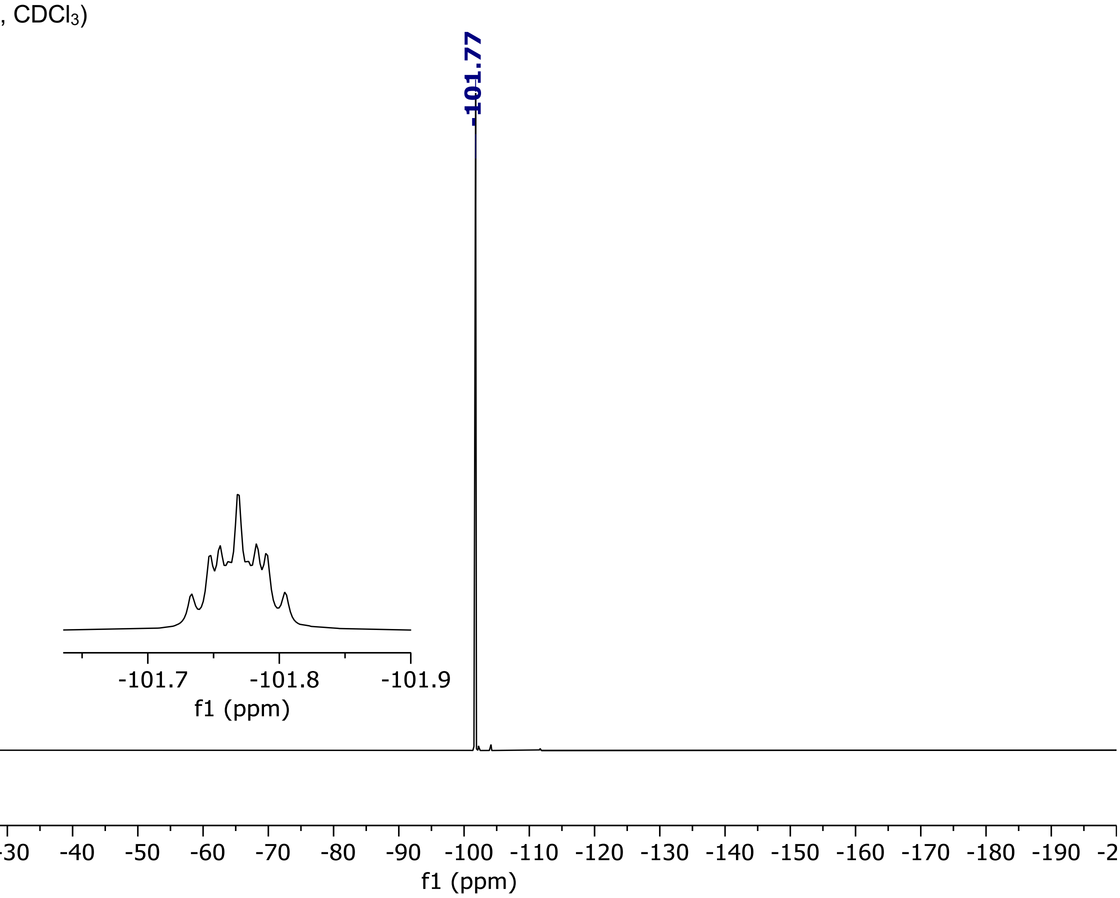

2,2-Diiodo-1-(4-fluorophenyl)ethanol

^{1}H NMR (400 MHz, $CDCl_3$)

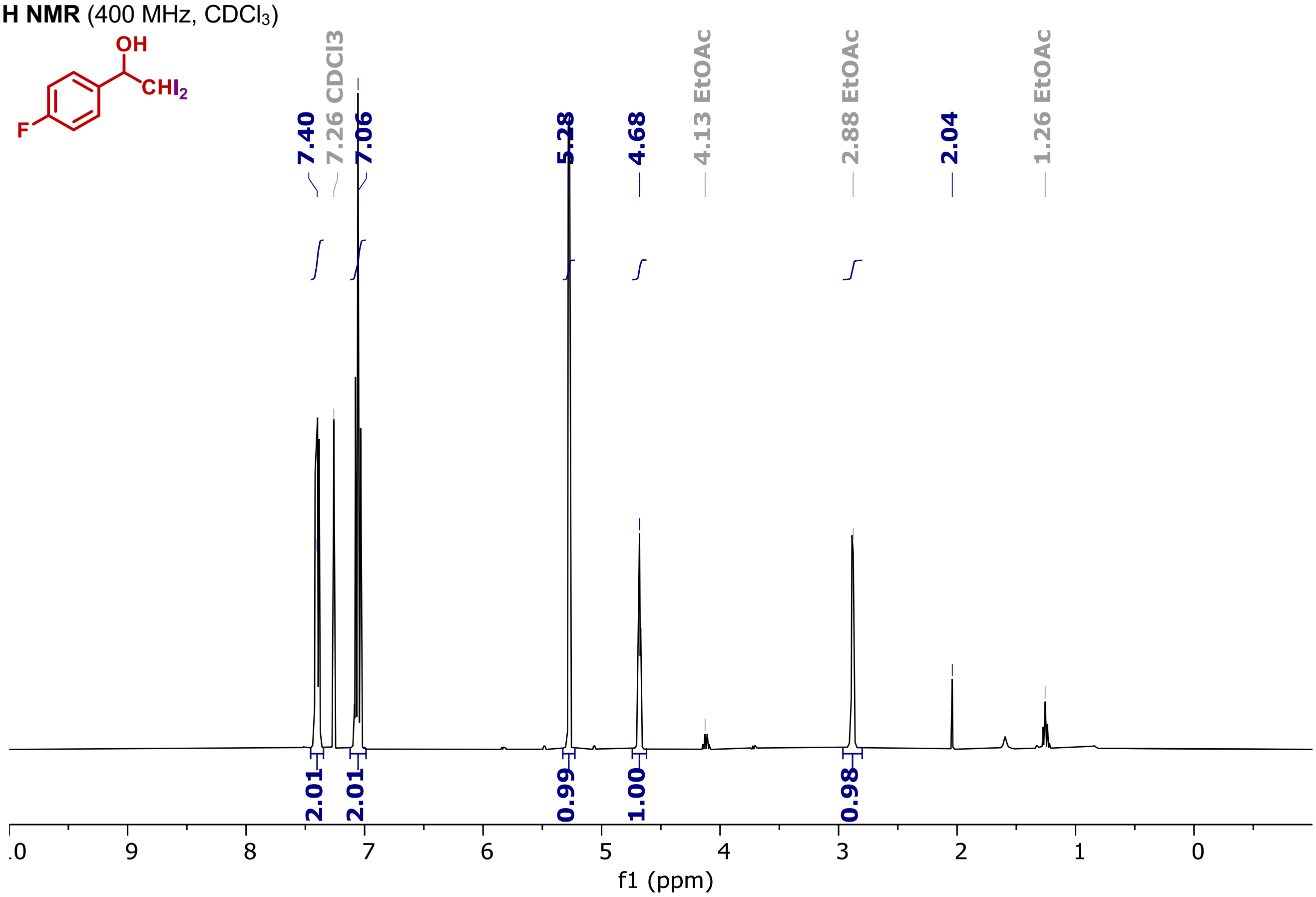

^{13}C NMR (101 MHz, $CDCl_3$)

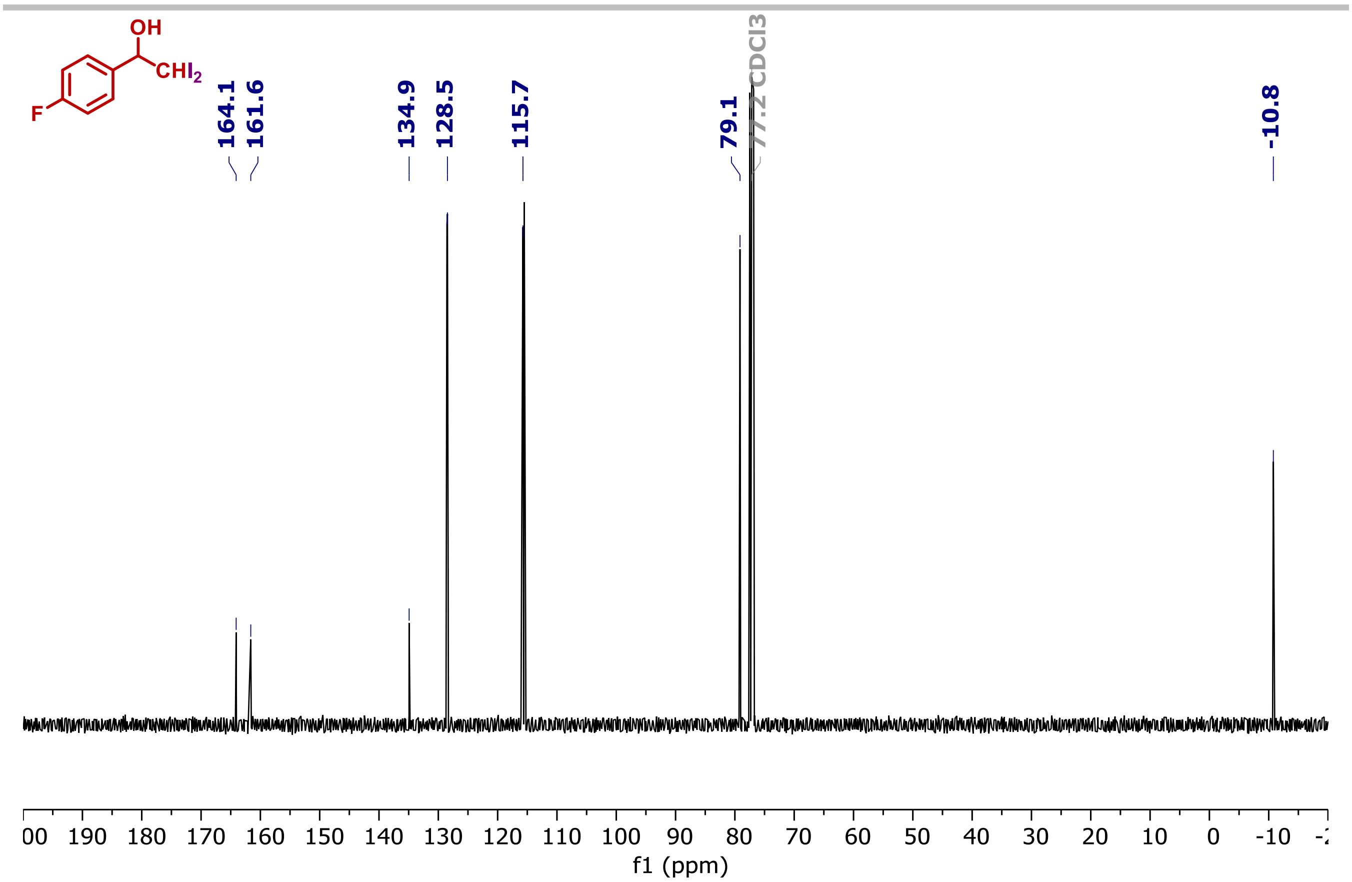

^{19}F NMR (376 MHz, $CDCl_3$)

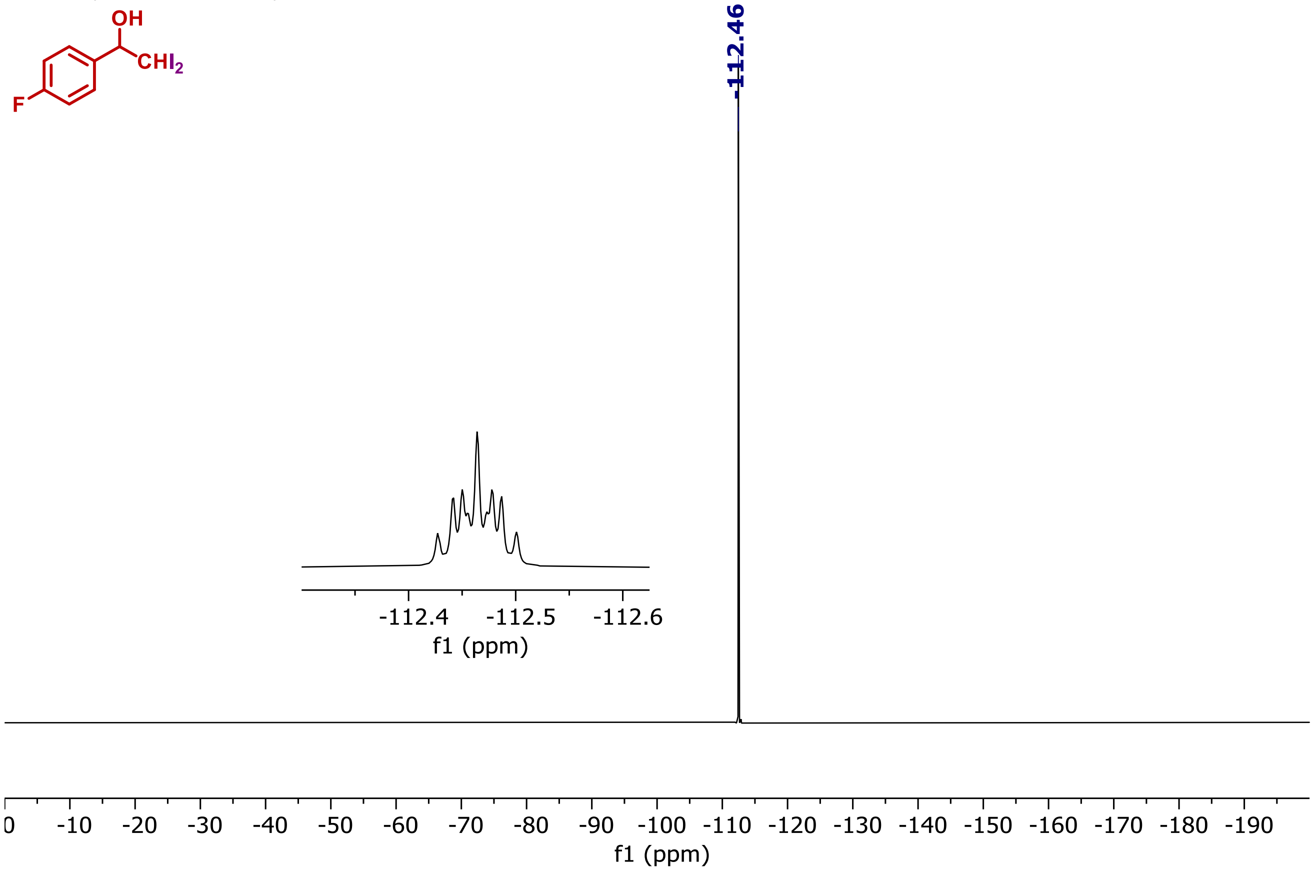

2,2-Diiodo-4'-fluoroacetophenone, **8a**

^{1}H NMR (400 MHz, $CDCl_3$)

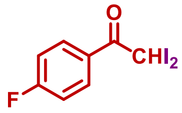

8a

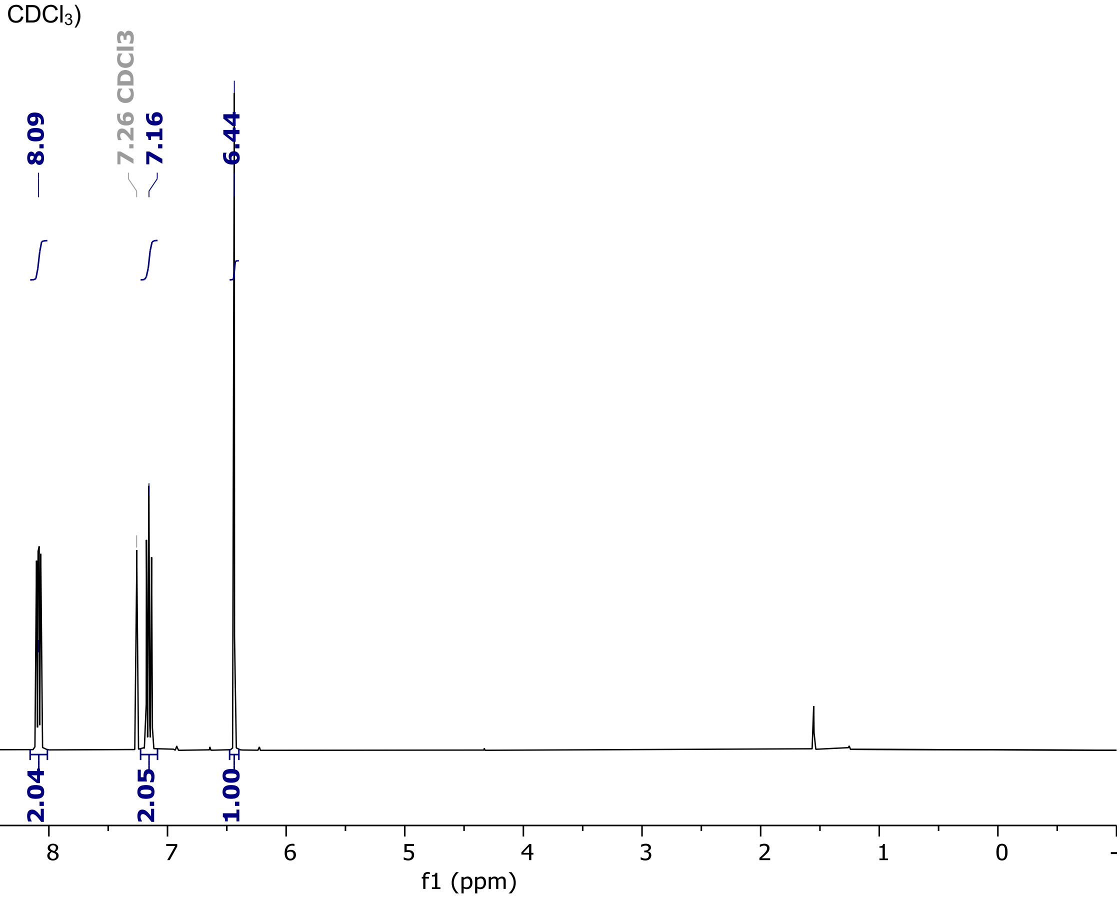

^{13}C NMR (101 MHz, $CDCl_3$)

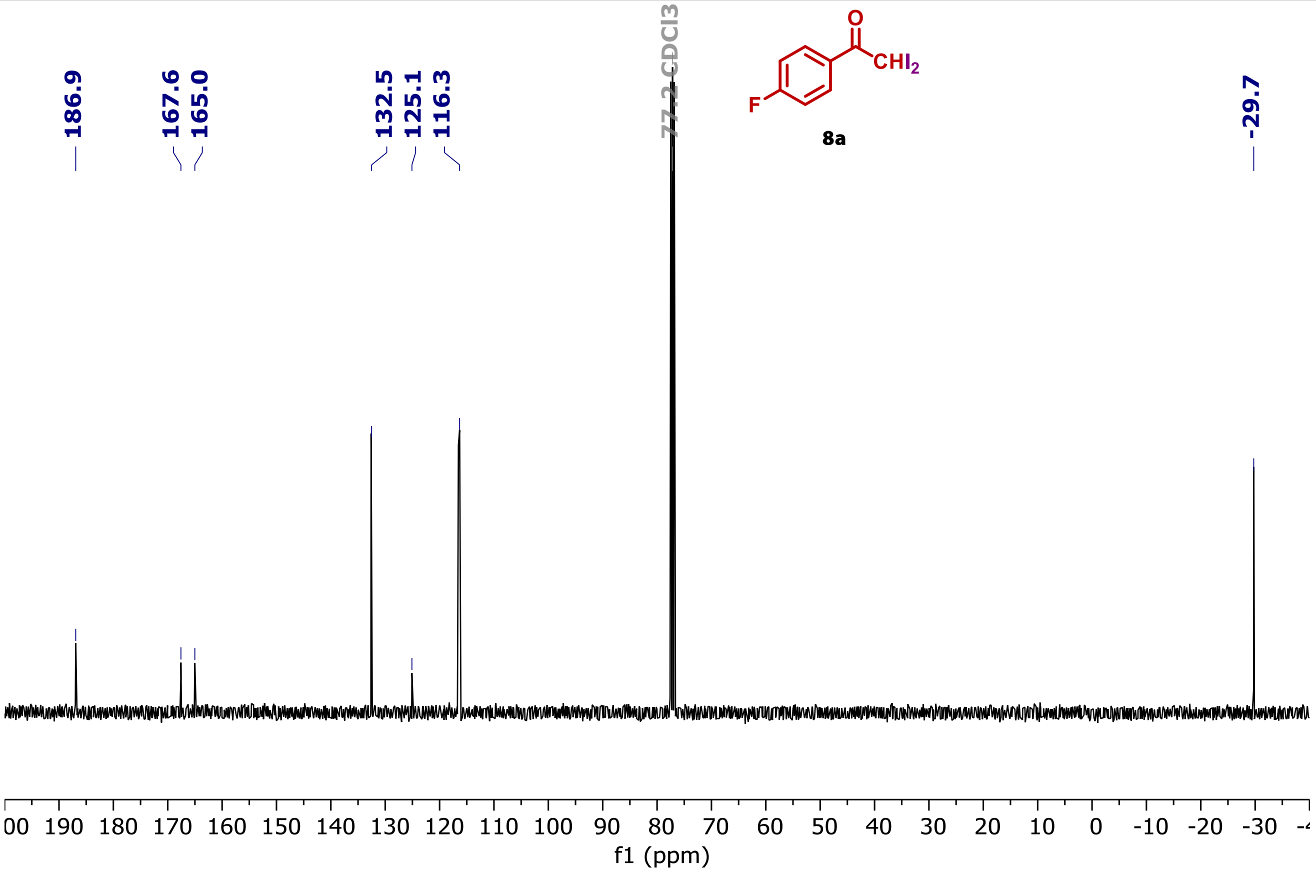

^{19}F NMR (376 MHz, $CDCl_3$)

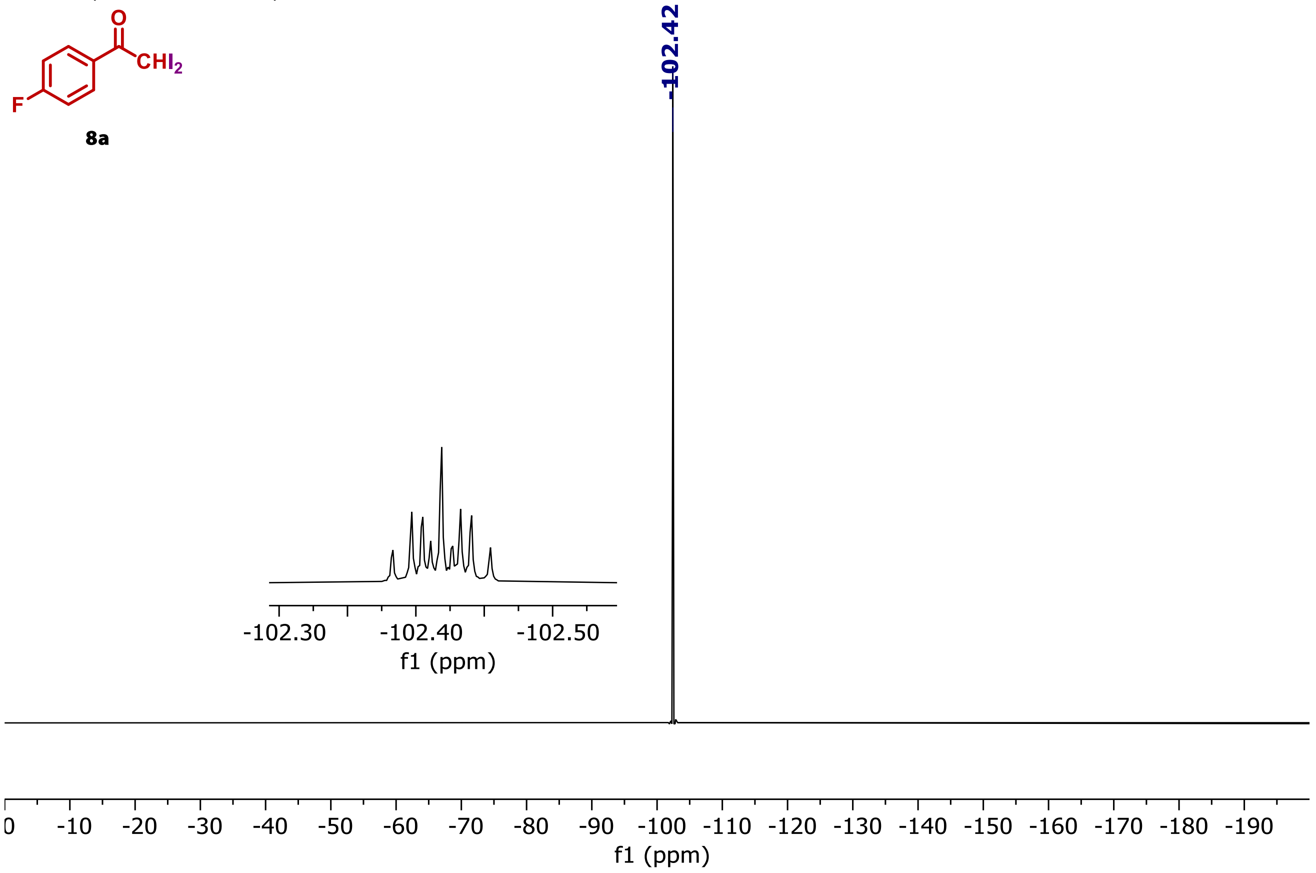

2,2-Dichloro-4'-fluoroacetophenone

^{19}F NMR (376 MHz, $CDCl_3$)

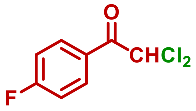

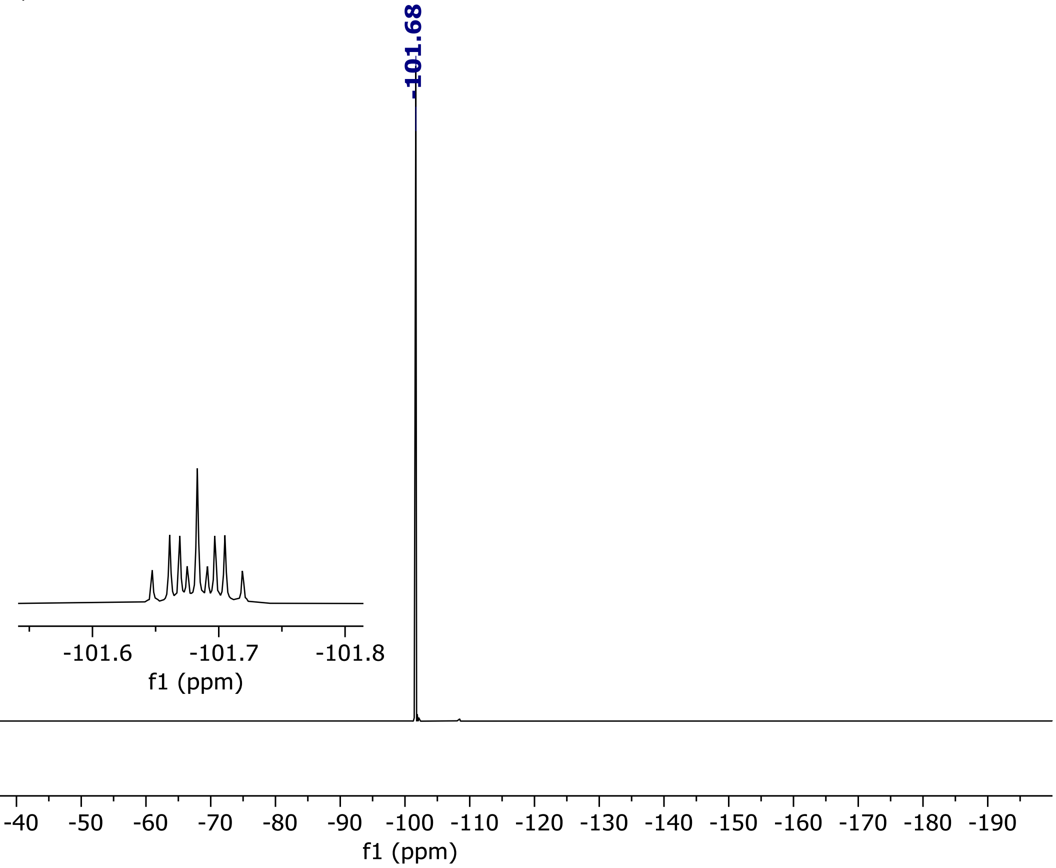

2,2,2-Tribromo-4'-fluoroacetophenone

^{19}F NMR (376 MHz, $CDCl_3$)

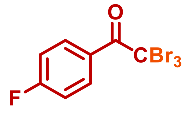

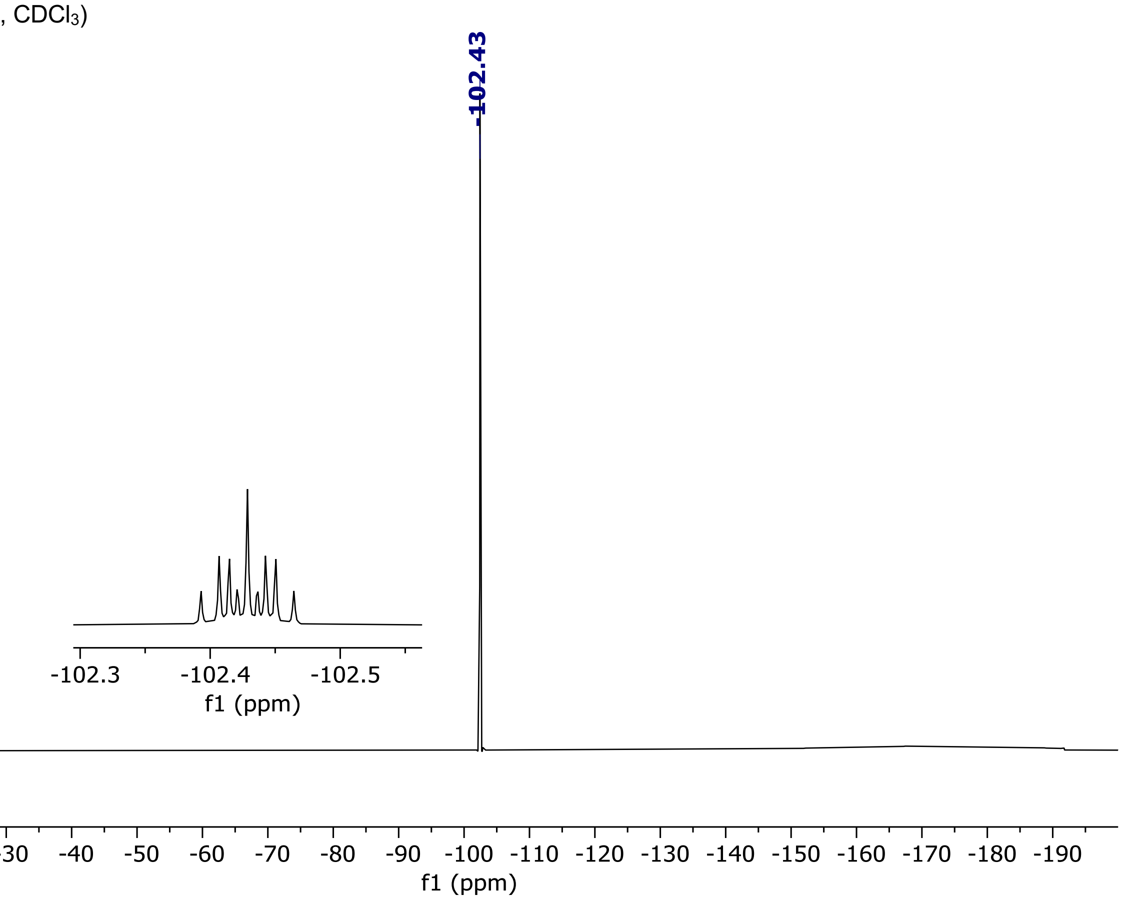

1,8-Diazabicyclo[5.4.0]undec-7-enium tetrafluoroborate

^{19}F NMR (376 MHz, $CDCl_3$)

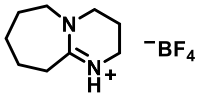

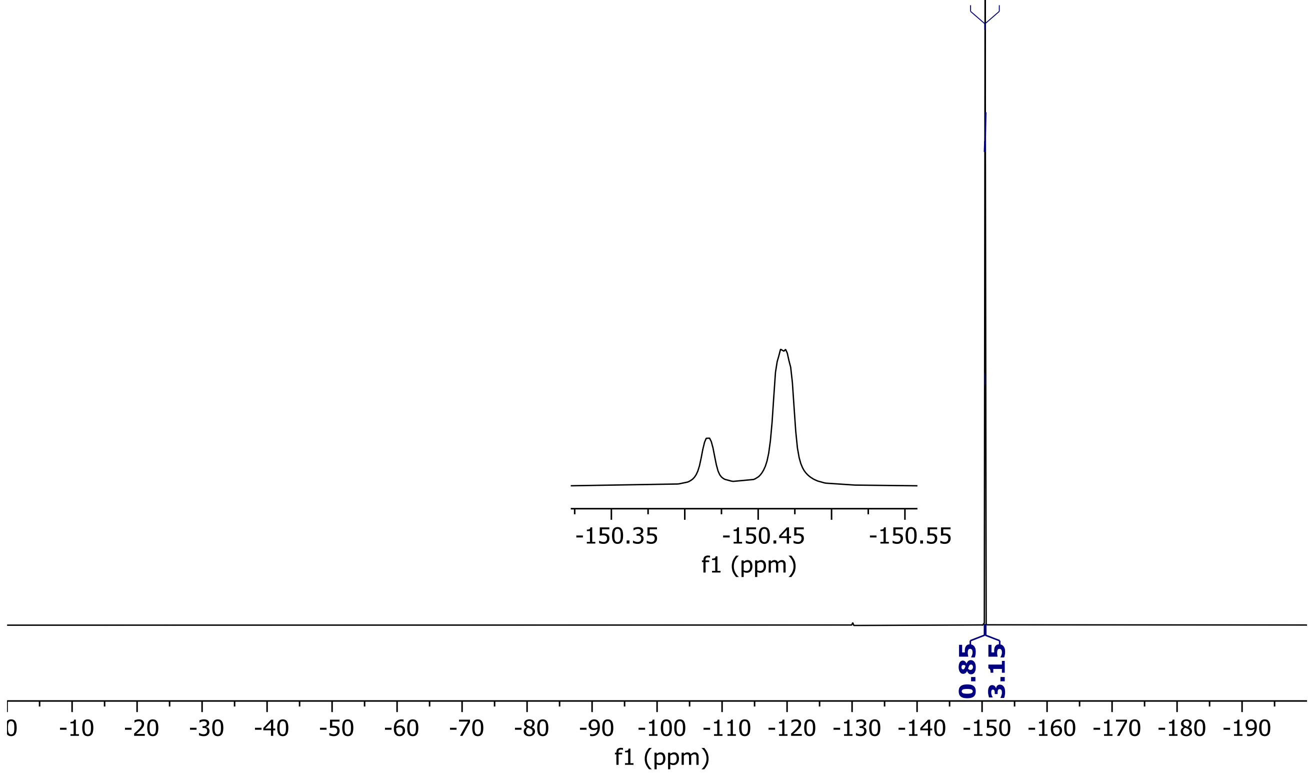

1-(2-Fluorophenyl)ethyl 2-fluorobenzoate

^{1}H NMR (400 MHz, $CDCl_3$)

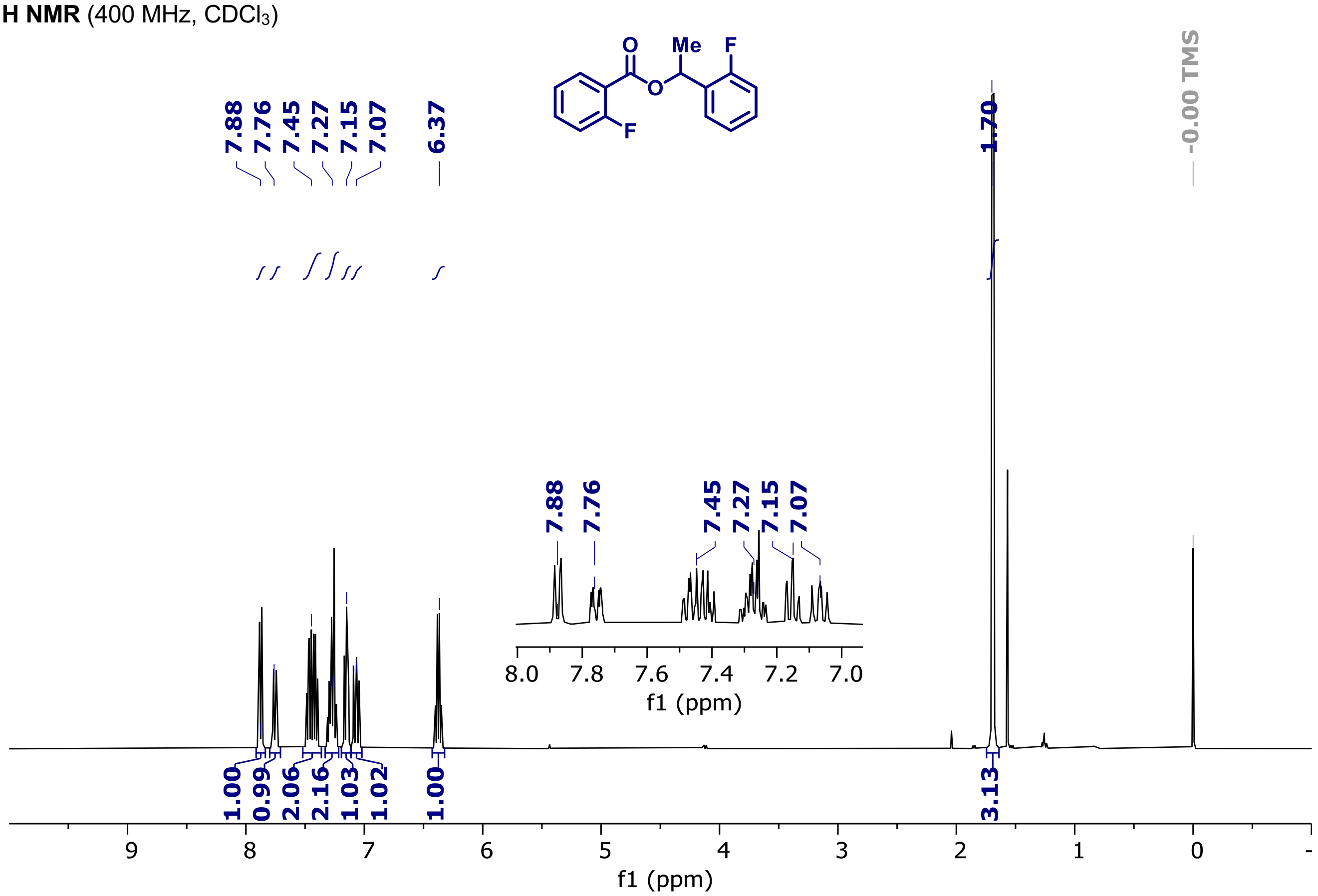

^{13}C NMR (101 MHz, $CDCl_3$)

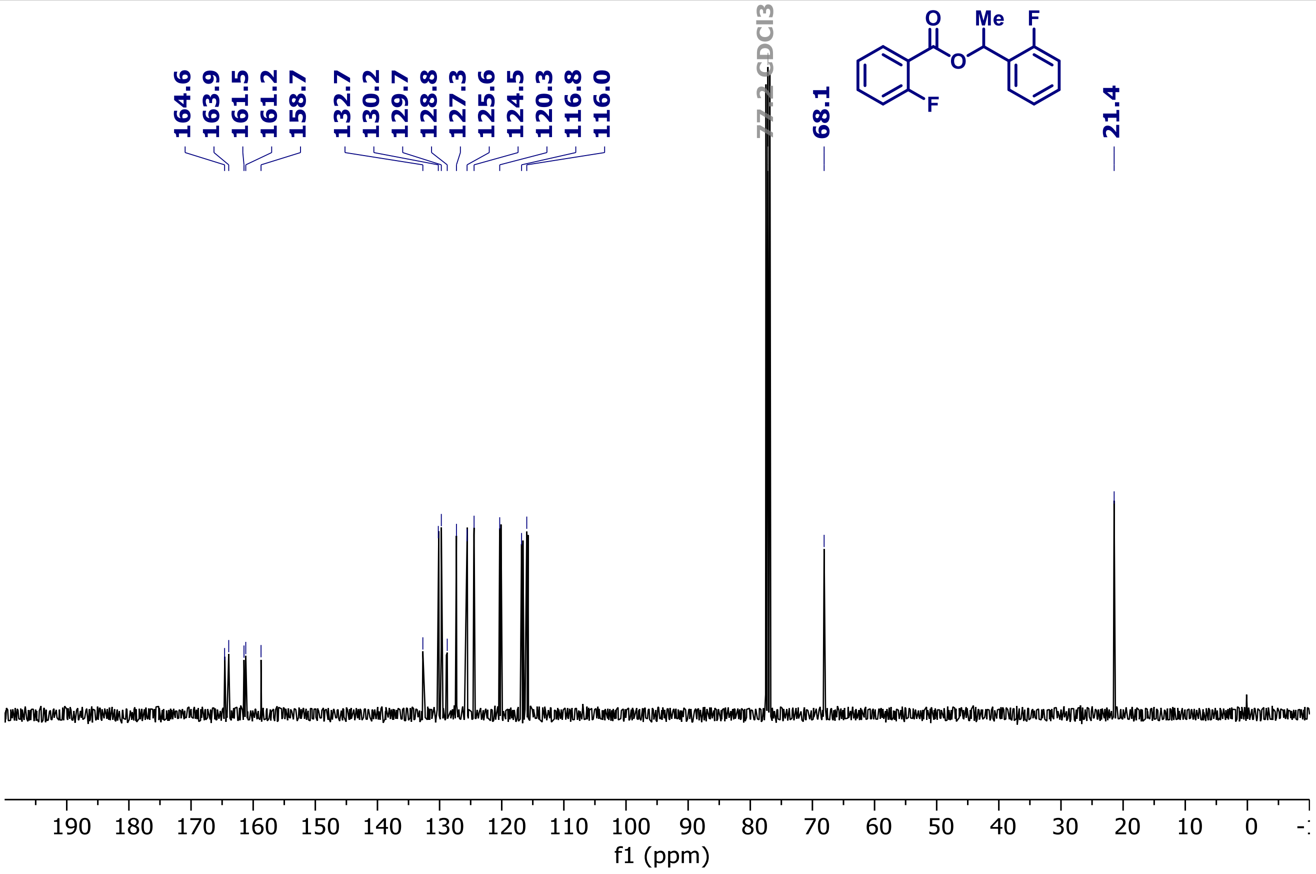

^{19}F NMR (376 MHz, $CDCl_3$)

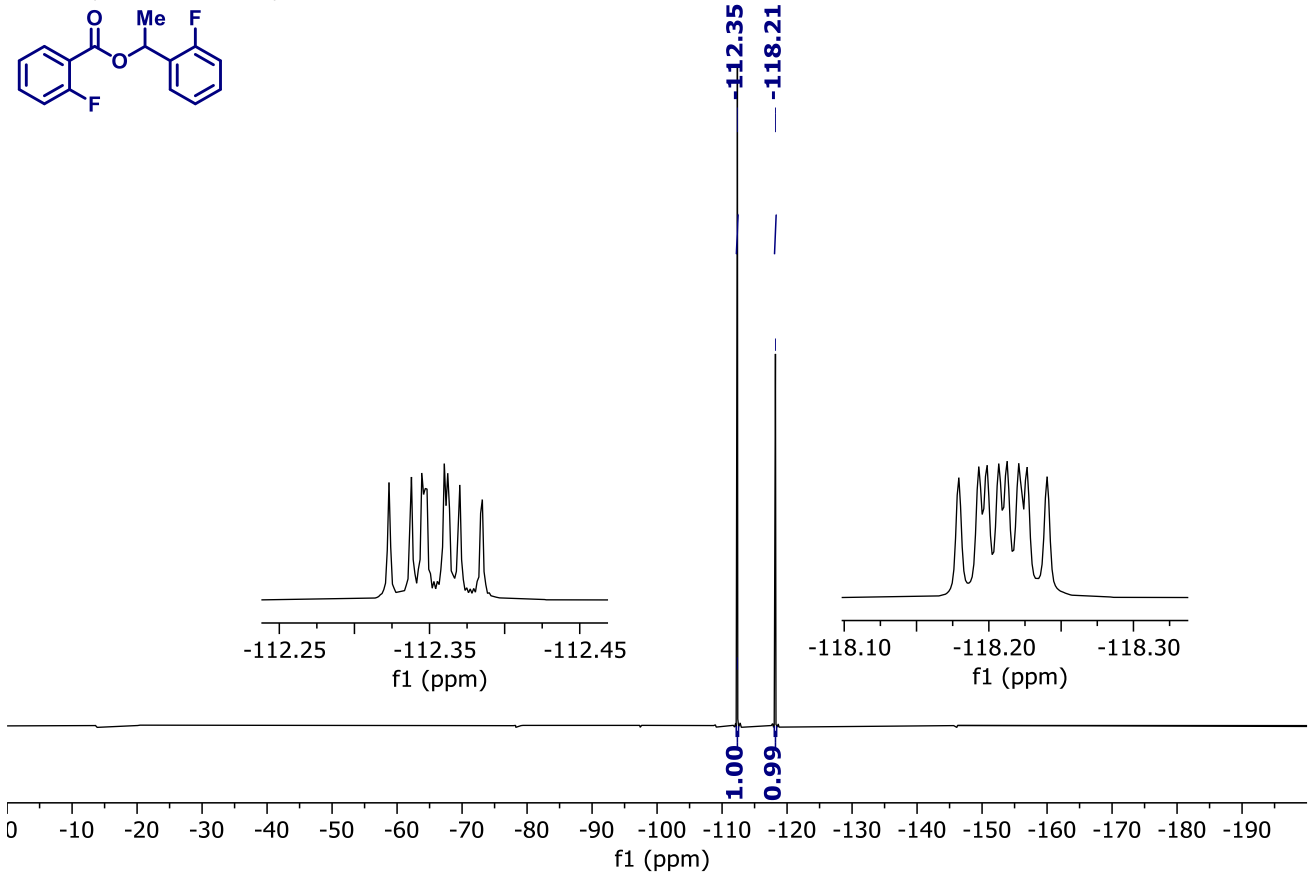

5-Acetyl-1-methyl-1H-pyrrole-2-carbonitrile

^{1}H NMR (400 MHz, $CDCl_3$)

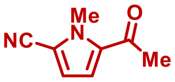

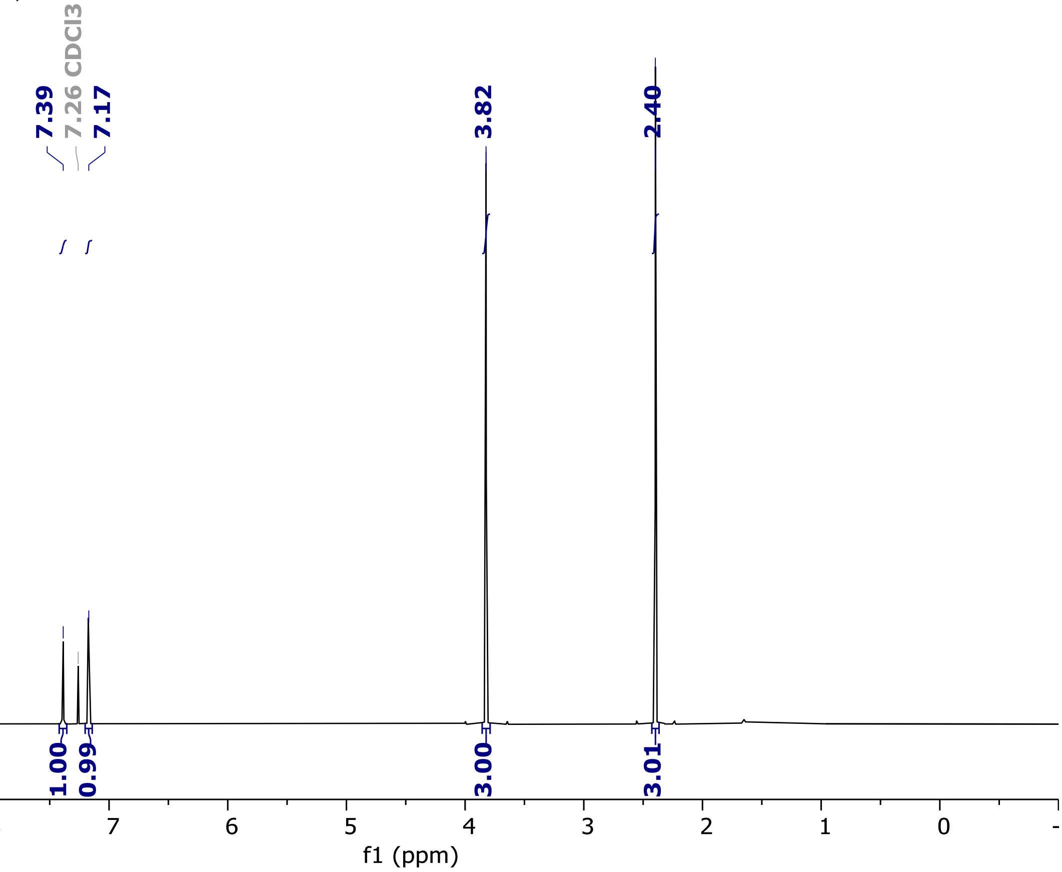

^{13}C NMR (101 MHz, $CDCl_3$)

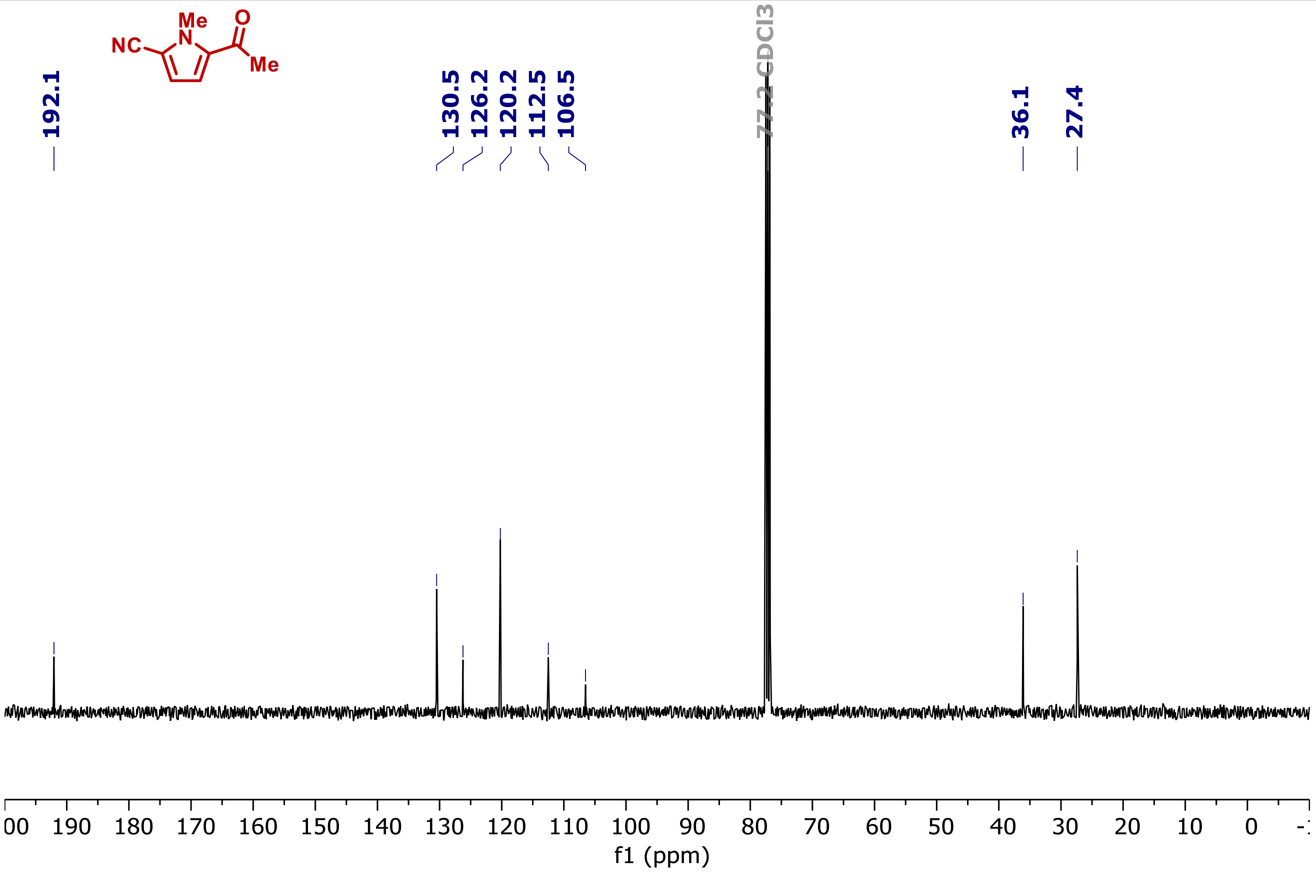
NC
Me
N
O
Me
192.1
130.5
126.2
120.2
112.5
106.5
77.2 CDCl3
36.1
27.4
00
190
180
170
160
150
140
130
120
110
100
90
80
70
60
50
40
30
20
10
0
f1 (ppm)

1-(4'-Fluoro-[1,1'-biphenyl]-4-yl)ethanol

^{19}F NMR (376 MHz, $CDCl_3$)

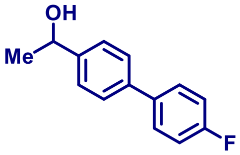

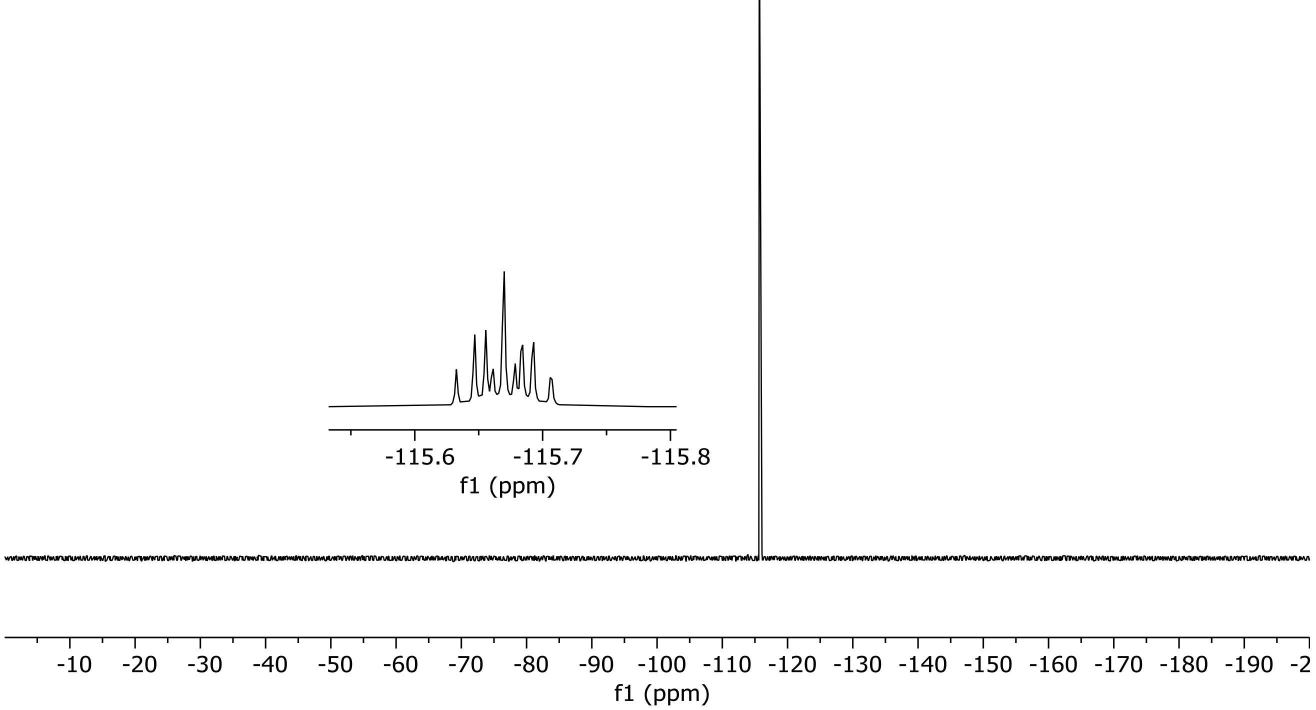

*1-(2-Fluorophenyl)ethyl 4-fluorobenzoate, **5a***

^{1}H NMR (400 MHz, $CDCl_3$)

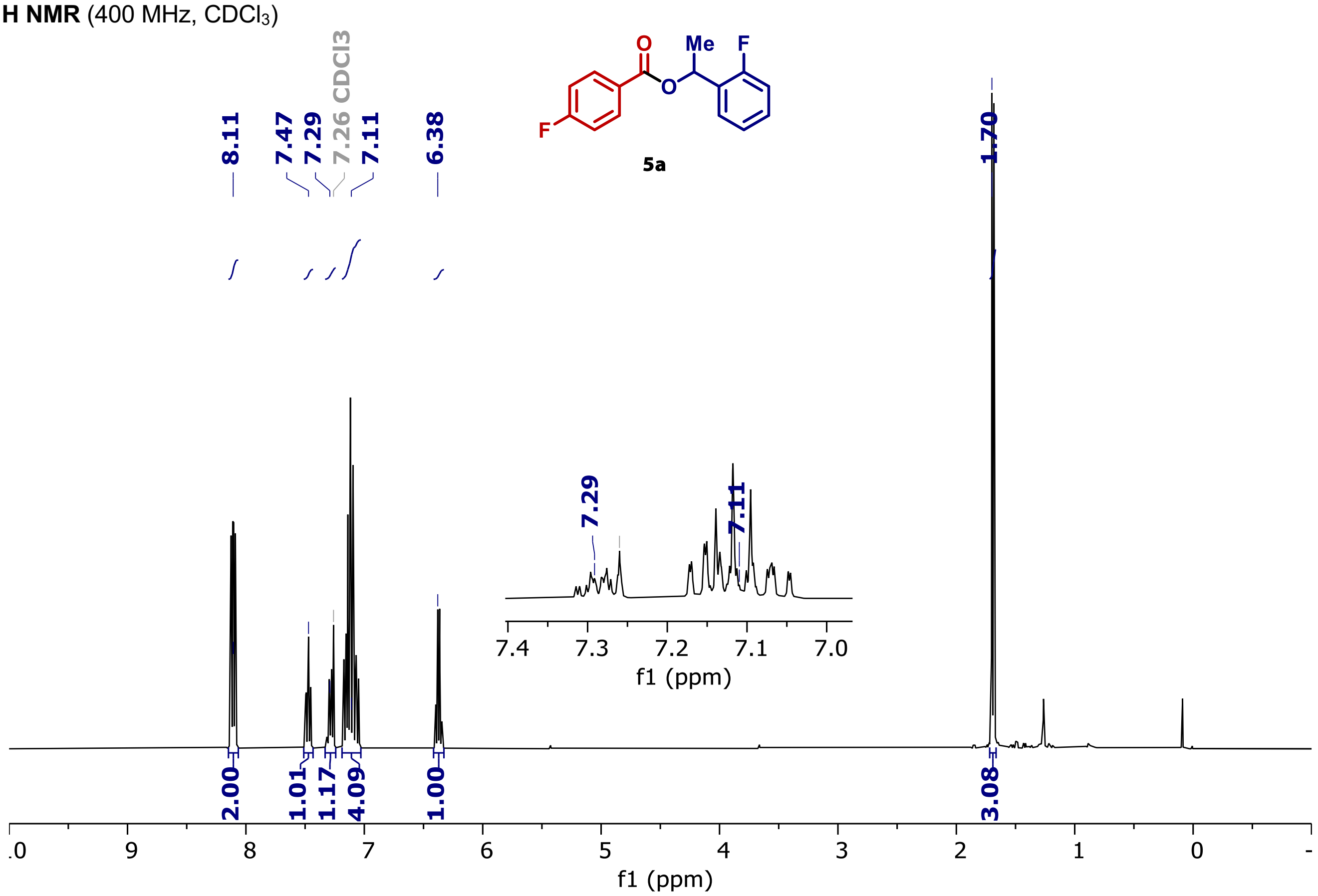

^{13}C NMR (101 MHz, $CDCl_3$)

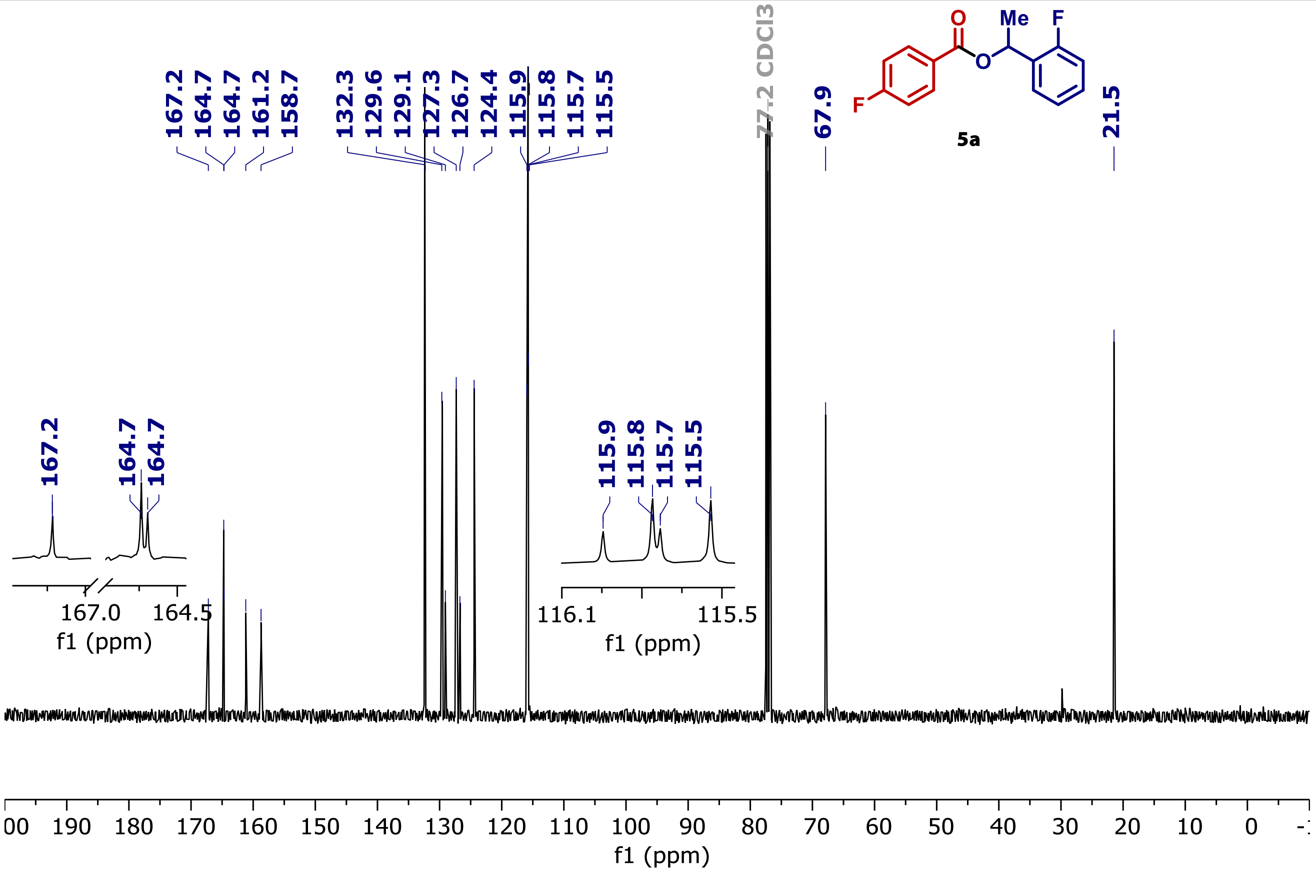

^{19}**F NMR** (376 MHz, $CDCl_3$)

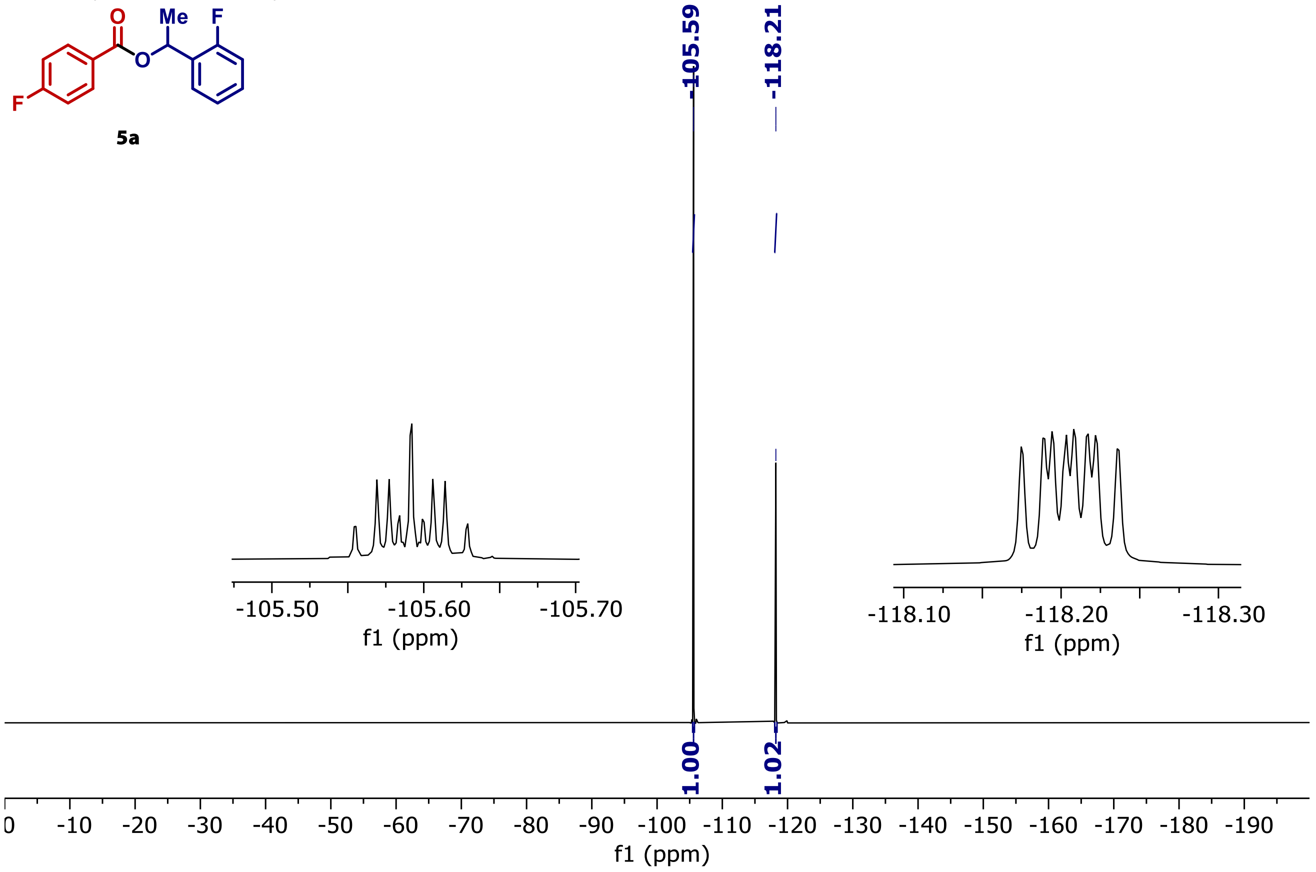

*1-(2-Fluorophenyl)ethyl 4-nitrobenzoate, **5b***

^{1}H NMR (400 MHz, $CDCl_3$)

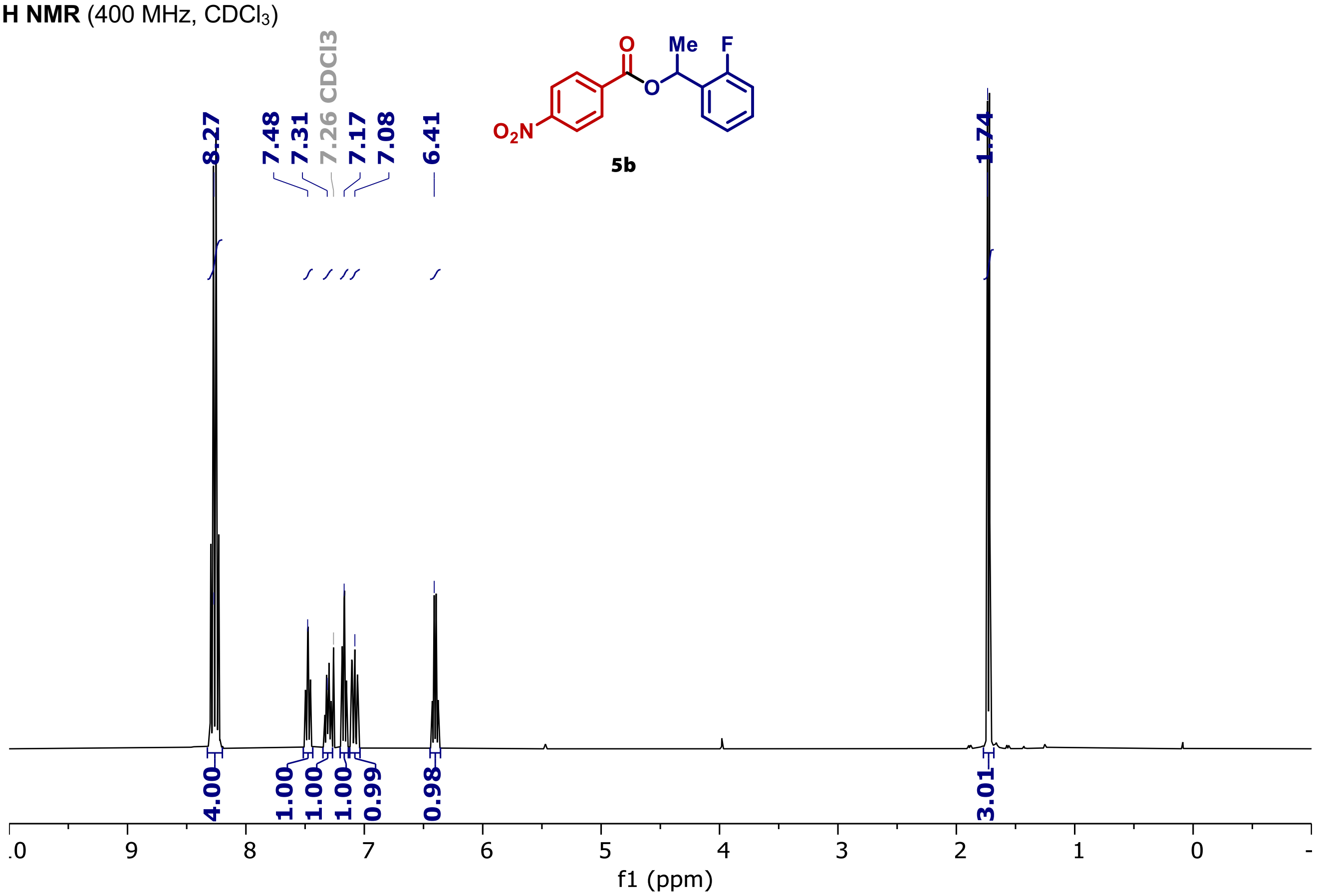

^{13}C NMR (101 MHz, $CDCl_3$)

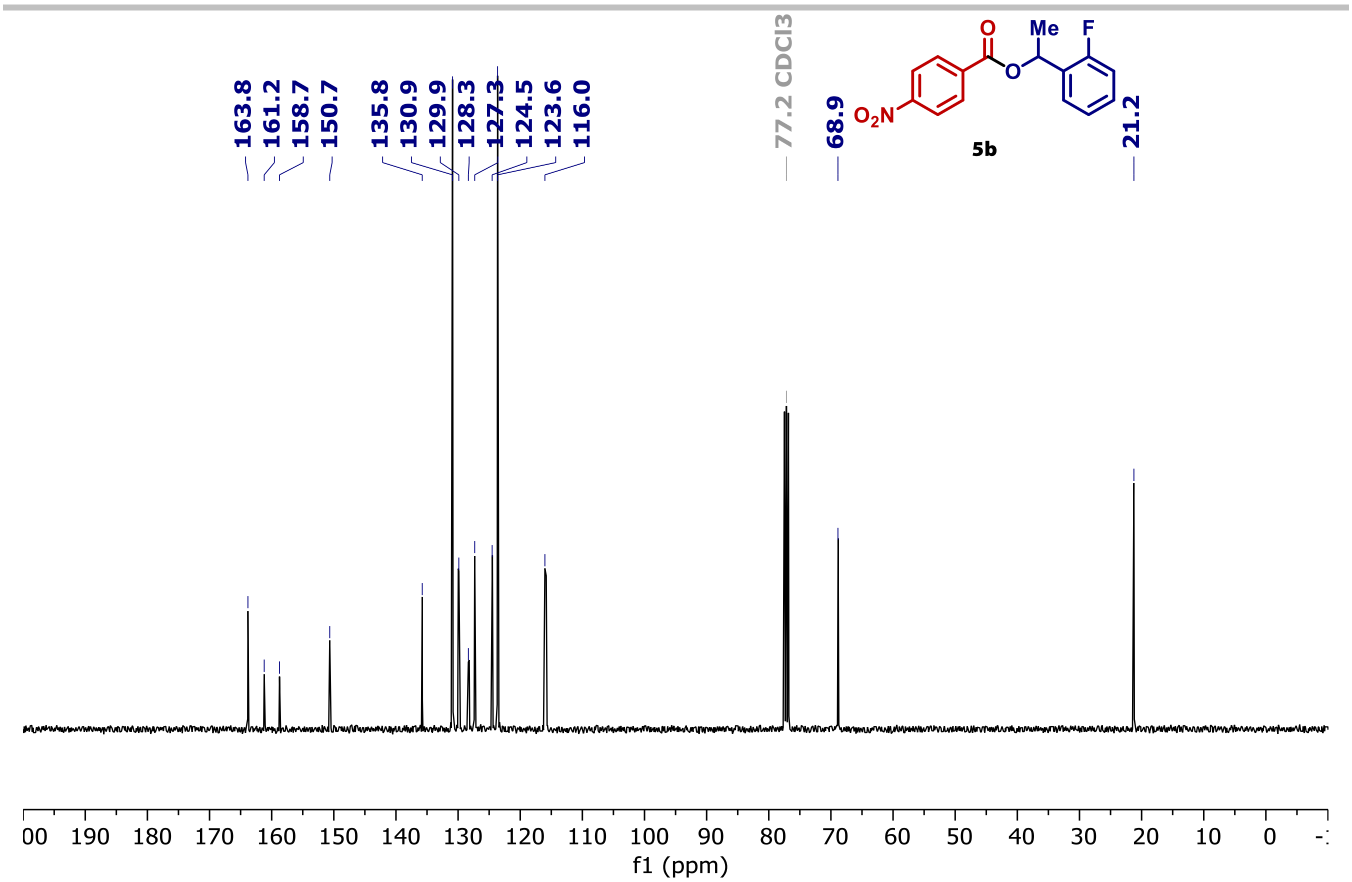

^{19}**F NMR** (376 MHz, $CDCl_3$)

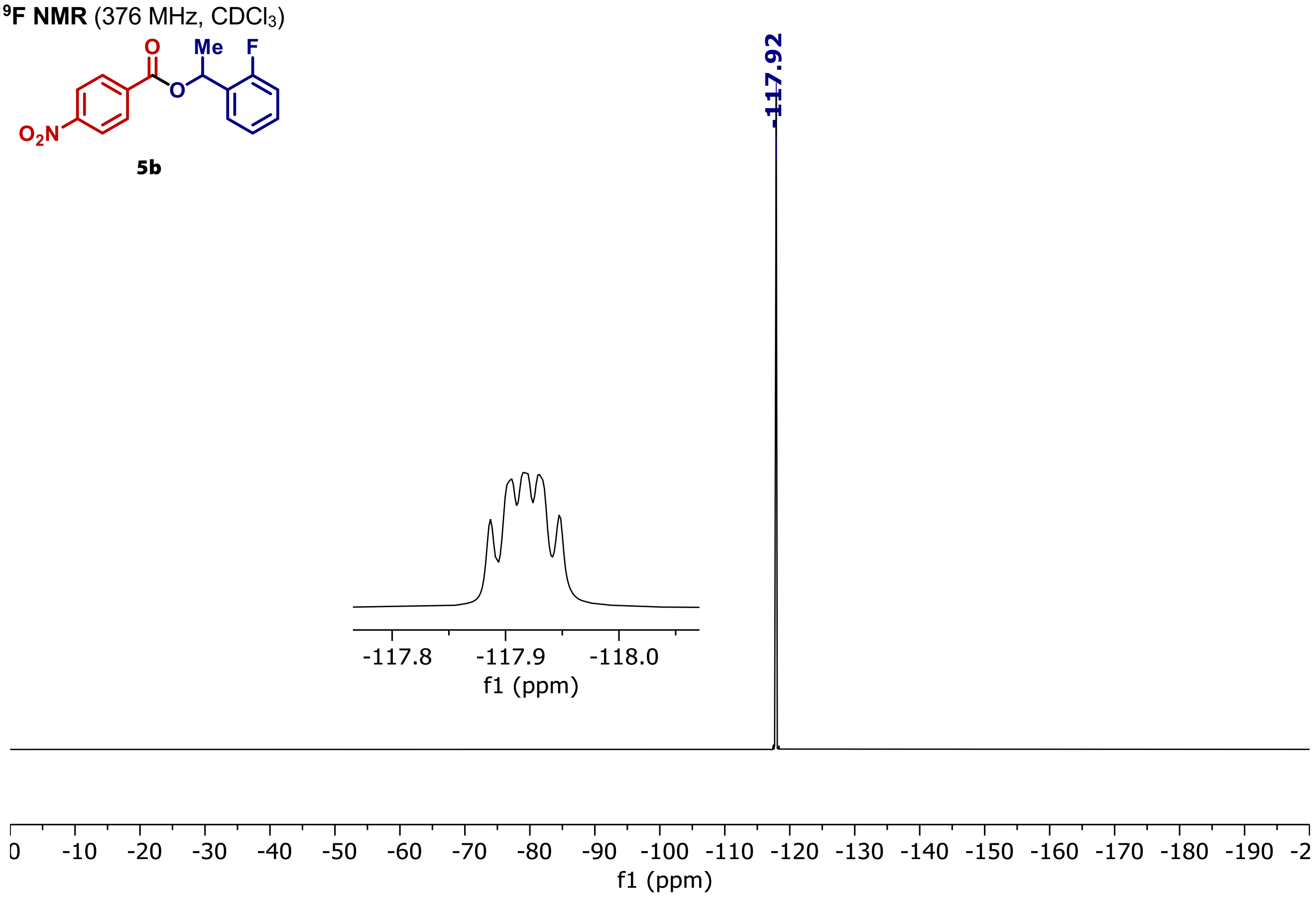

1-(2-Fluorophenyl)ethyl 4-methoxybenzoate, ***5c***

^{1}H NMR (400 MHz, $CDCl_3$)

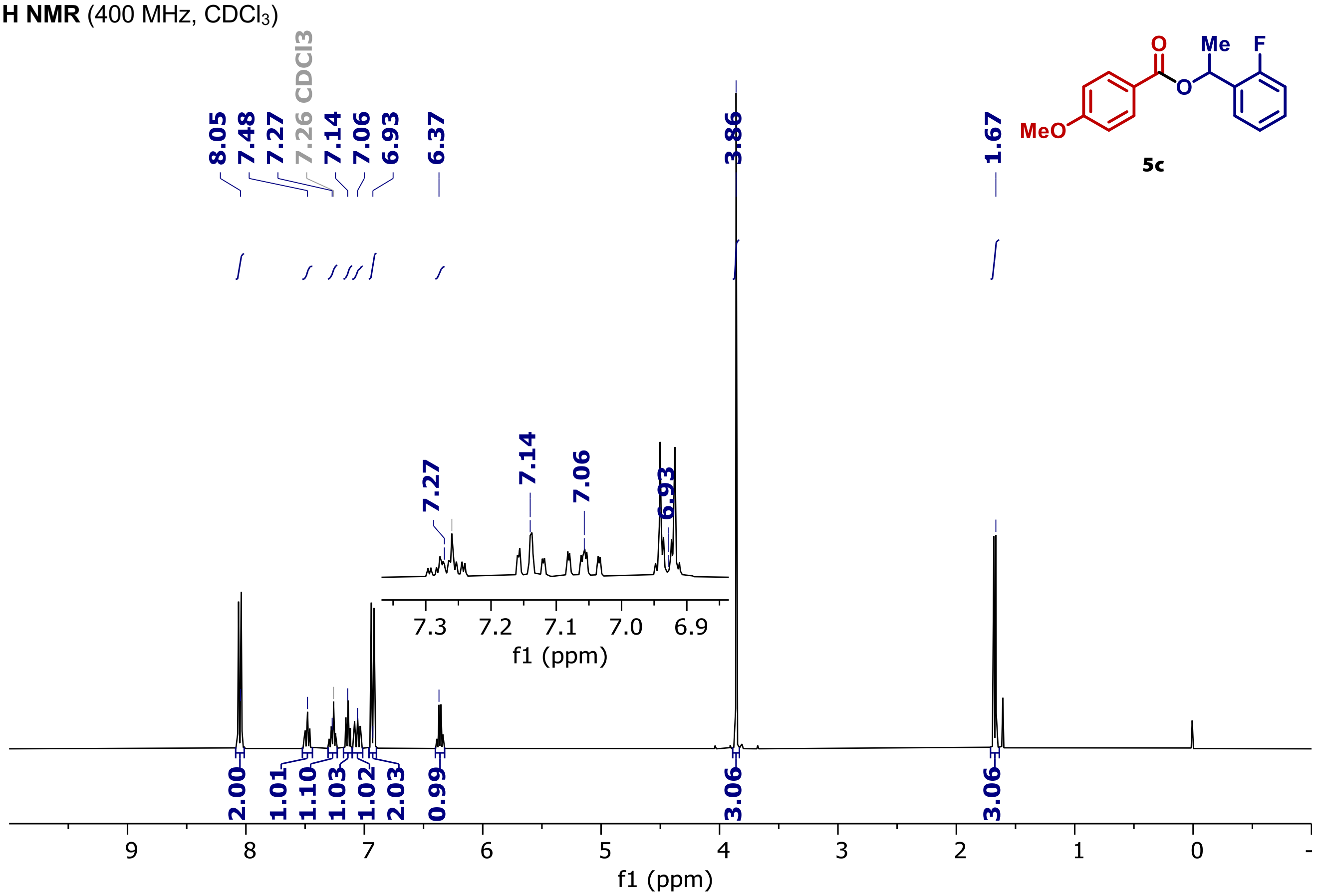

^{13}C NMR (101 MHz, $CDCl_3$)

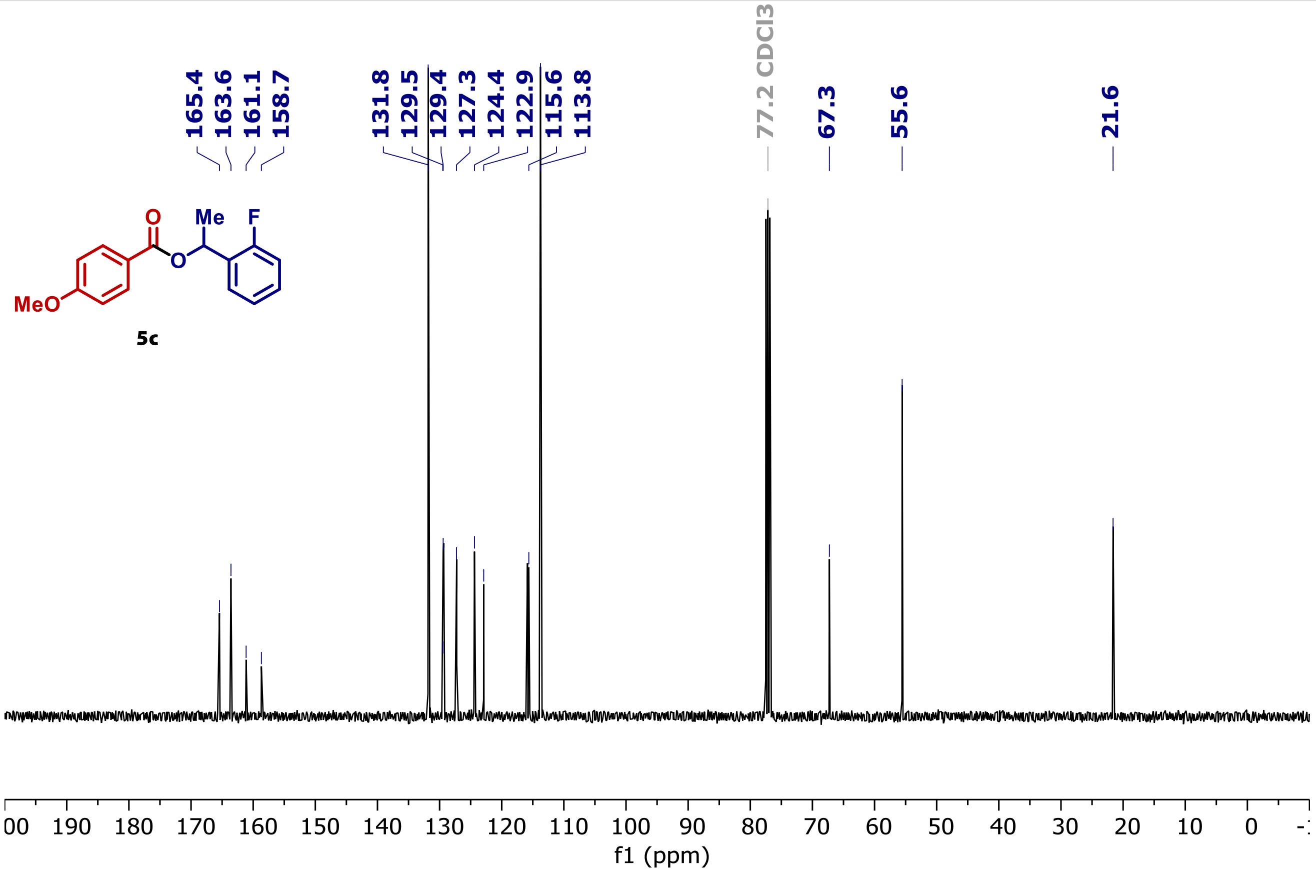

^{19}F NMR (376 MHz, $CDCl_3$)

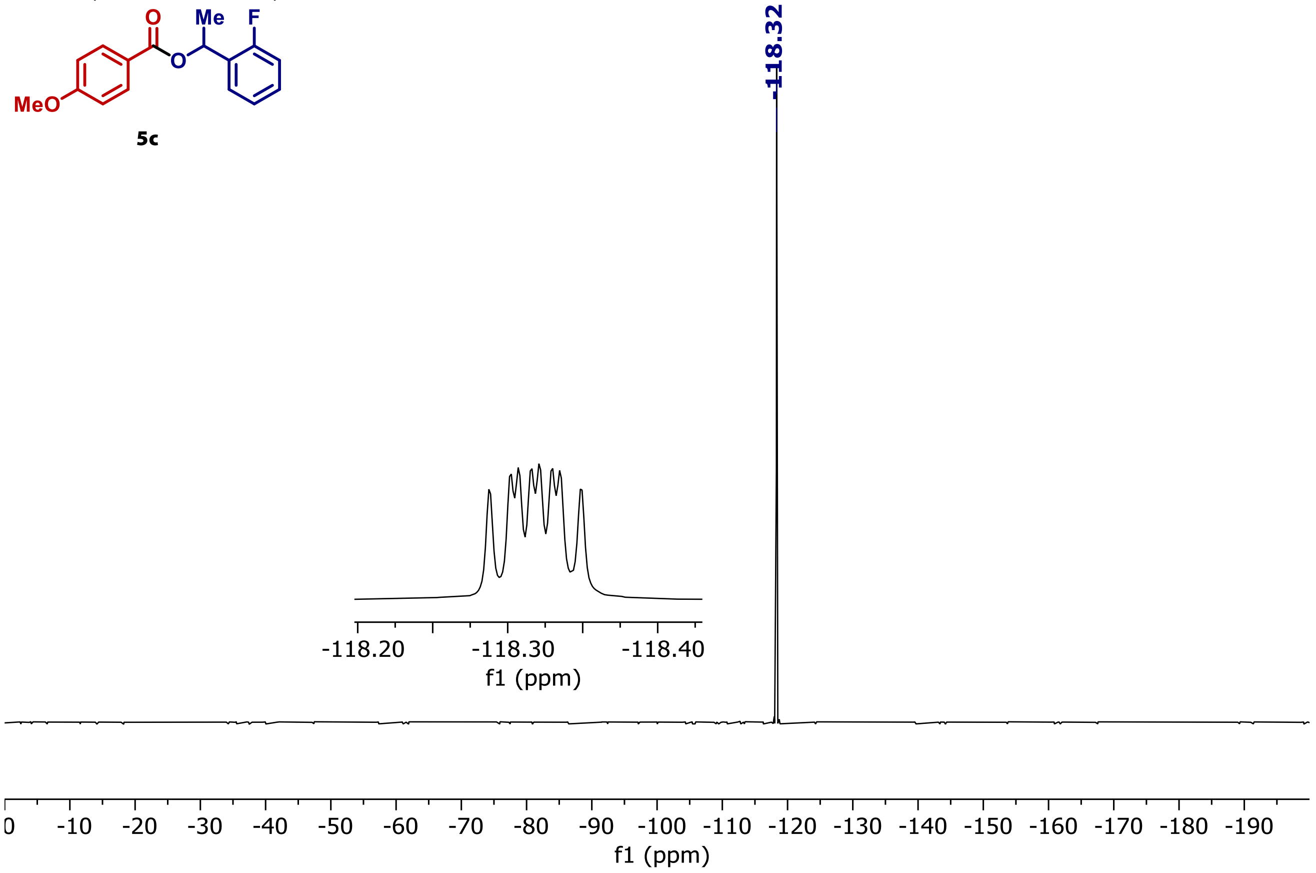

*1-(2-Fluorophenyl)ethyl picolinate, **5d***

^{1}H NMR (400 MHz, $CDCl_3$)

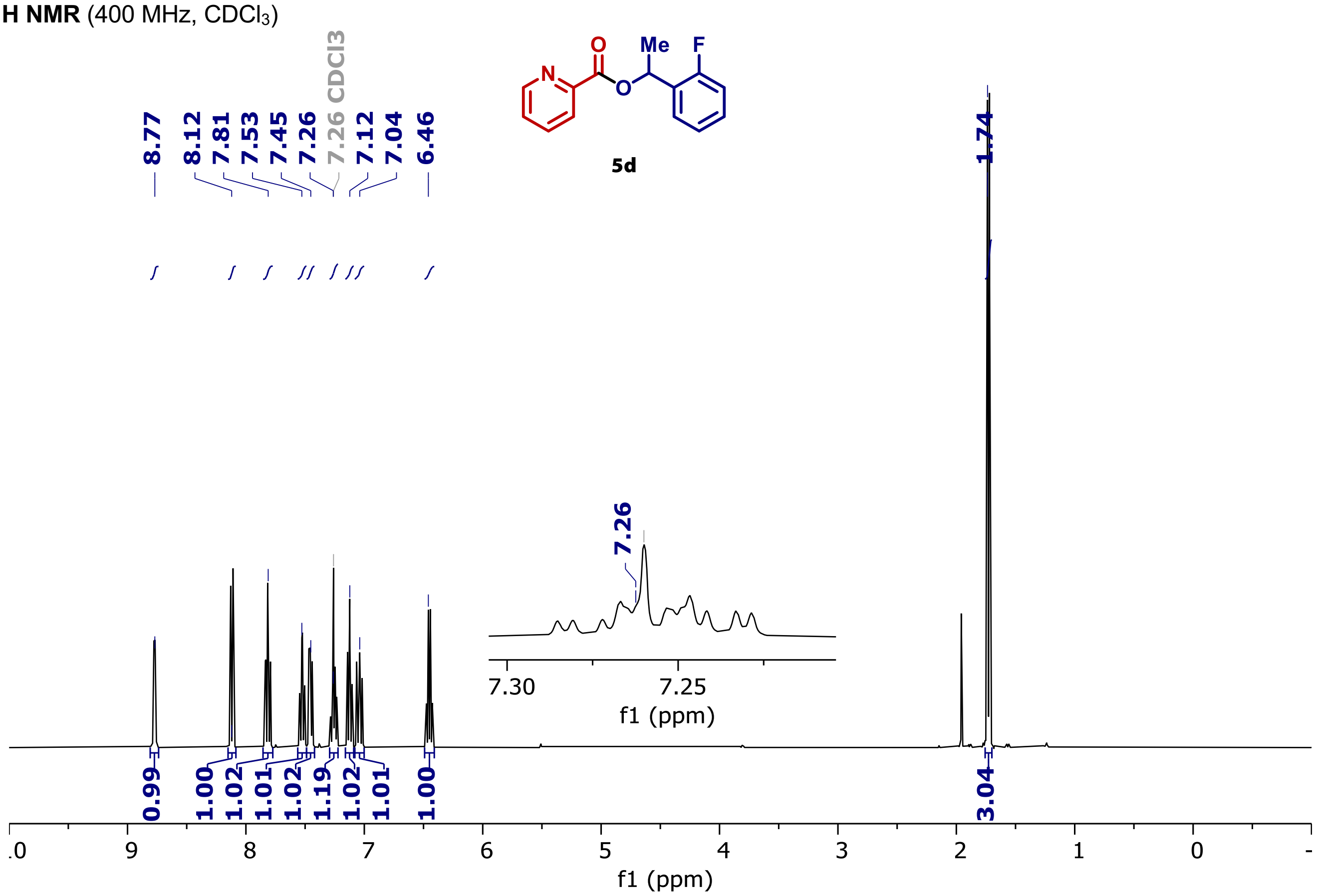

^{13}C NMR (101 MHz, $CDCl_3$)

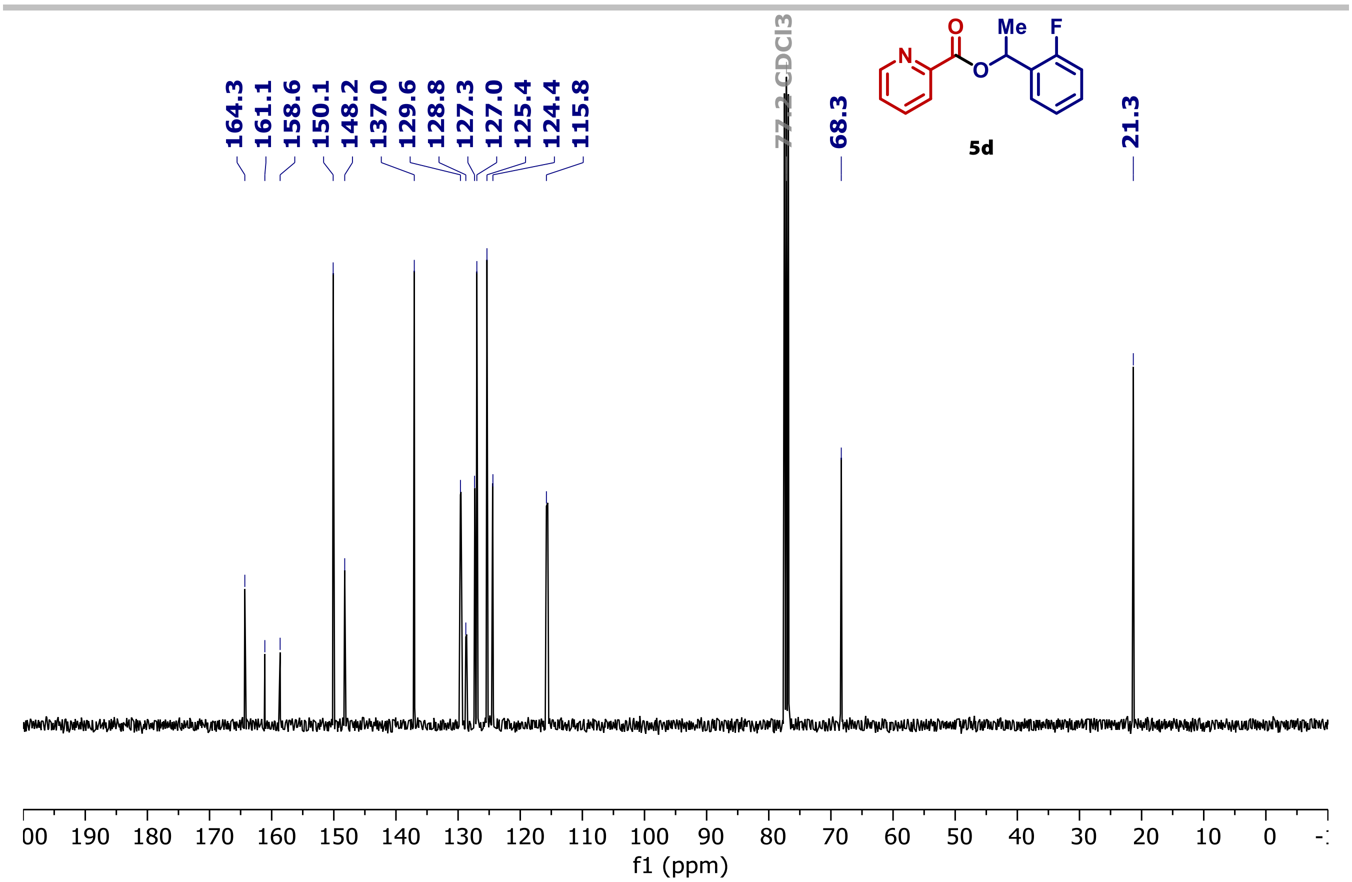

^{19}F NMR (376 MHz, $CDCl_3$)

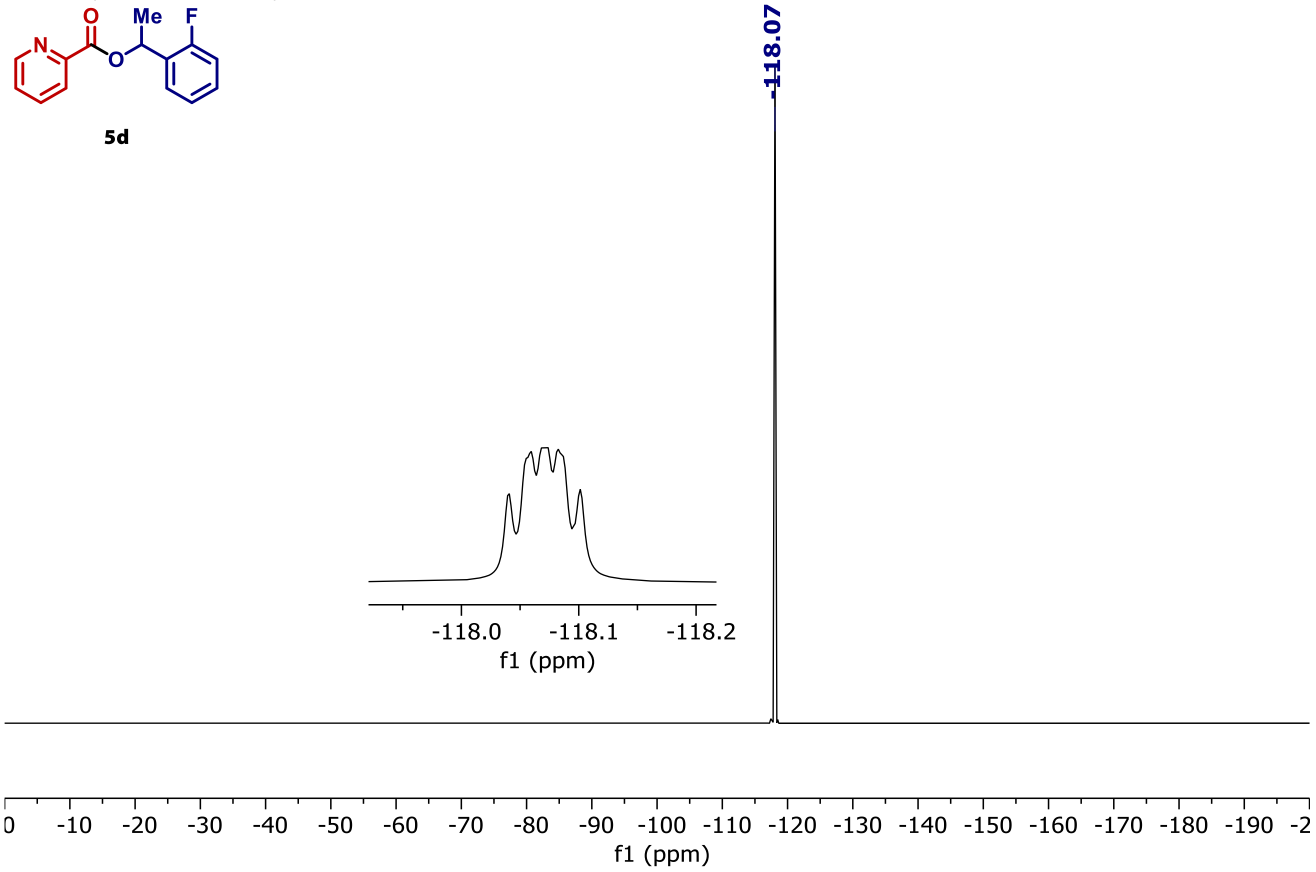

1-(2-Fluorophenyl)ethyl 5-cyano-1-methyl-1H-pyrrole-2-carboxylate, ***5e***

^{1}H NMR (400 MHz, $CDCl_3$)

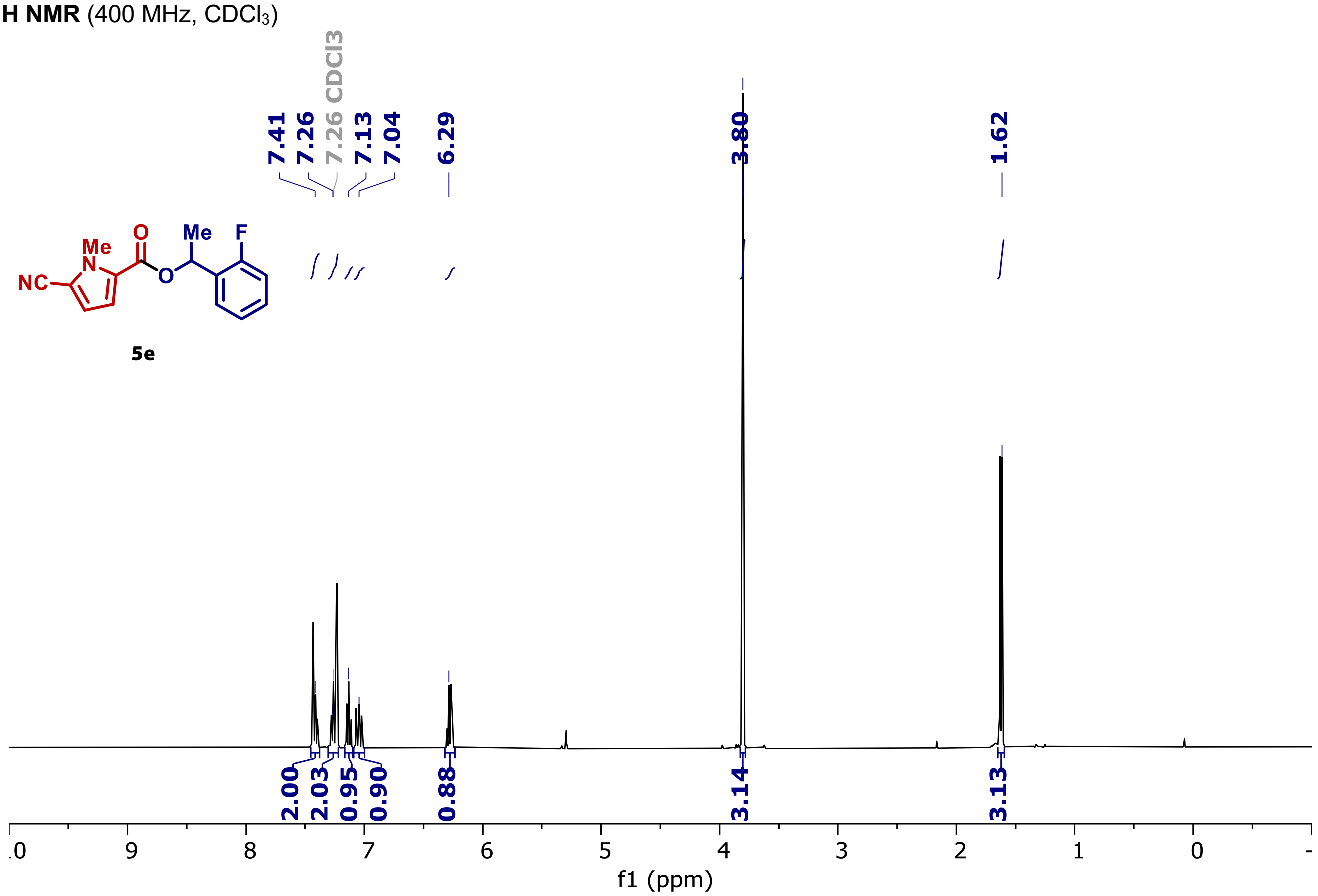

^{13}C NMR (101 MHz, $CDCl_3$)

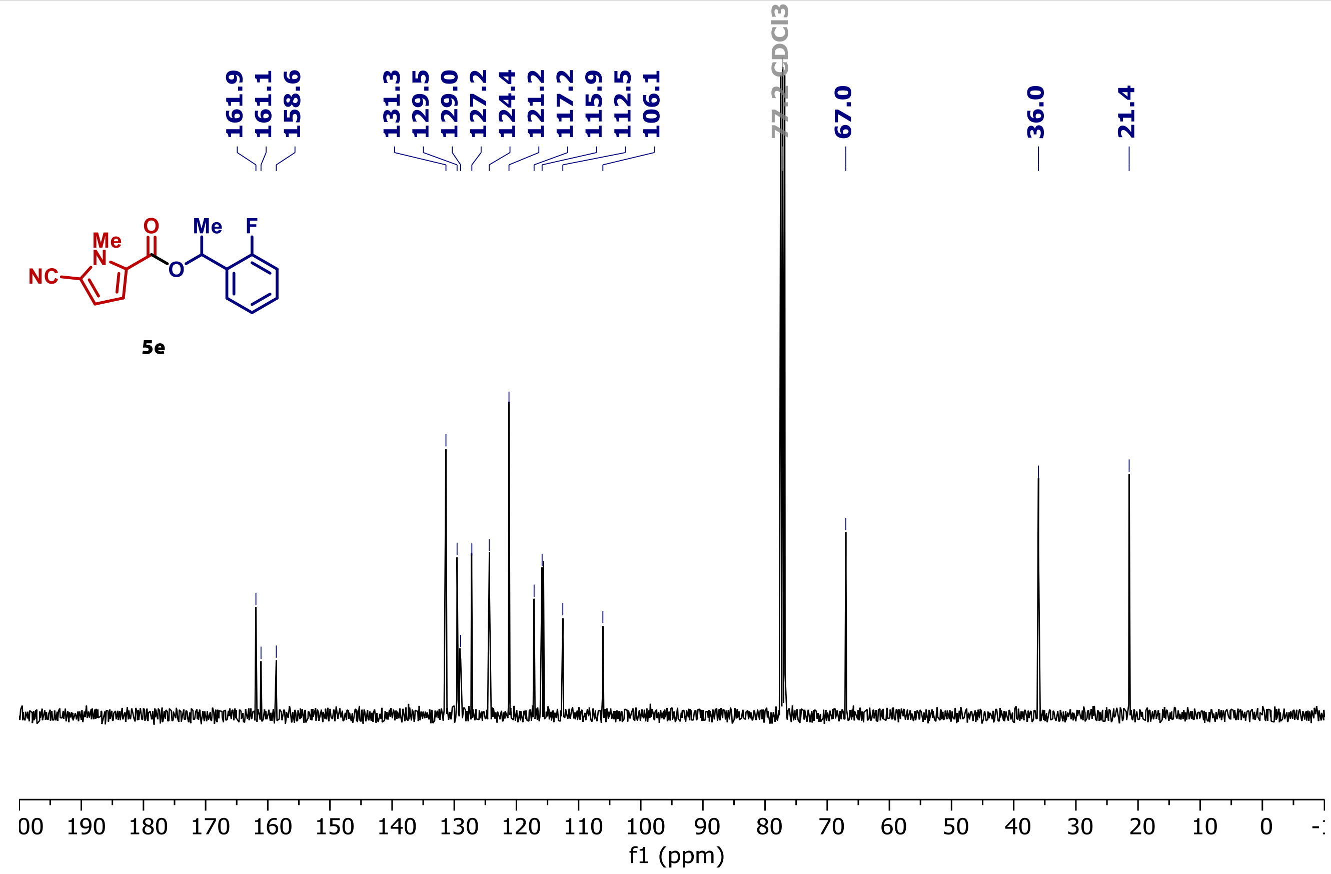

^{19}F NMR (376 MHz, $CDCl_3$)

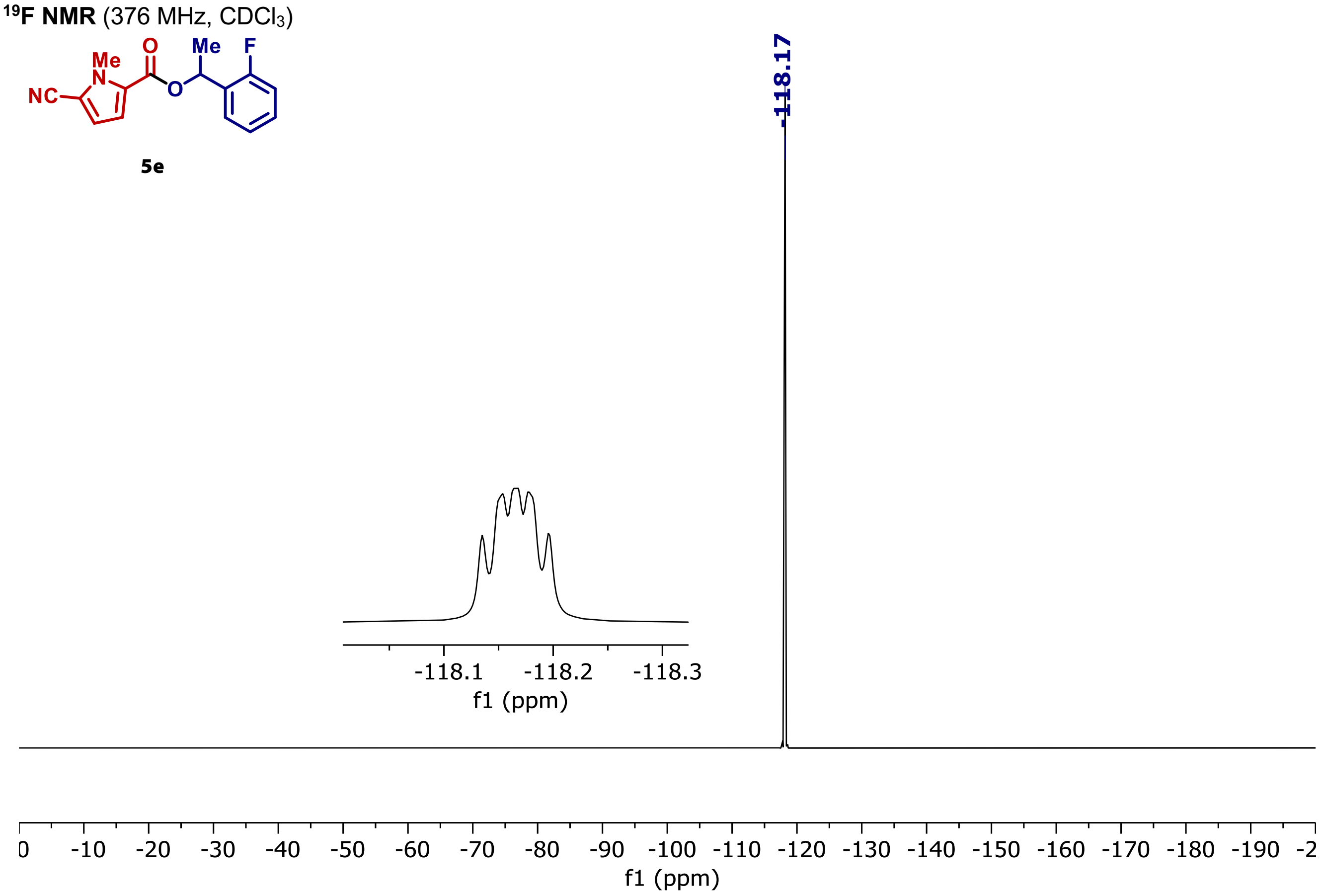

*1-(2-Fluorophenyl)ethyl 3-methylbenzoate, **5f***

^{1}H NMR (400 MHz, $CDCl_3$)

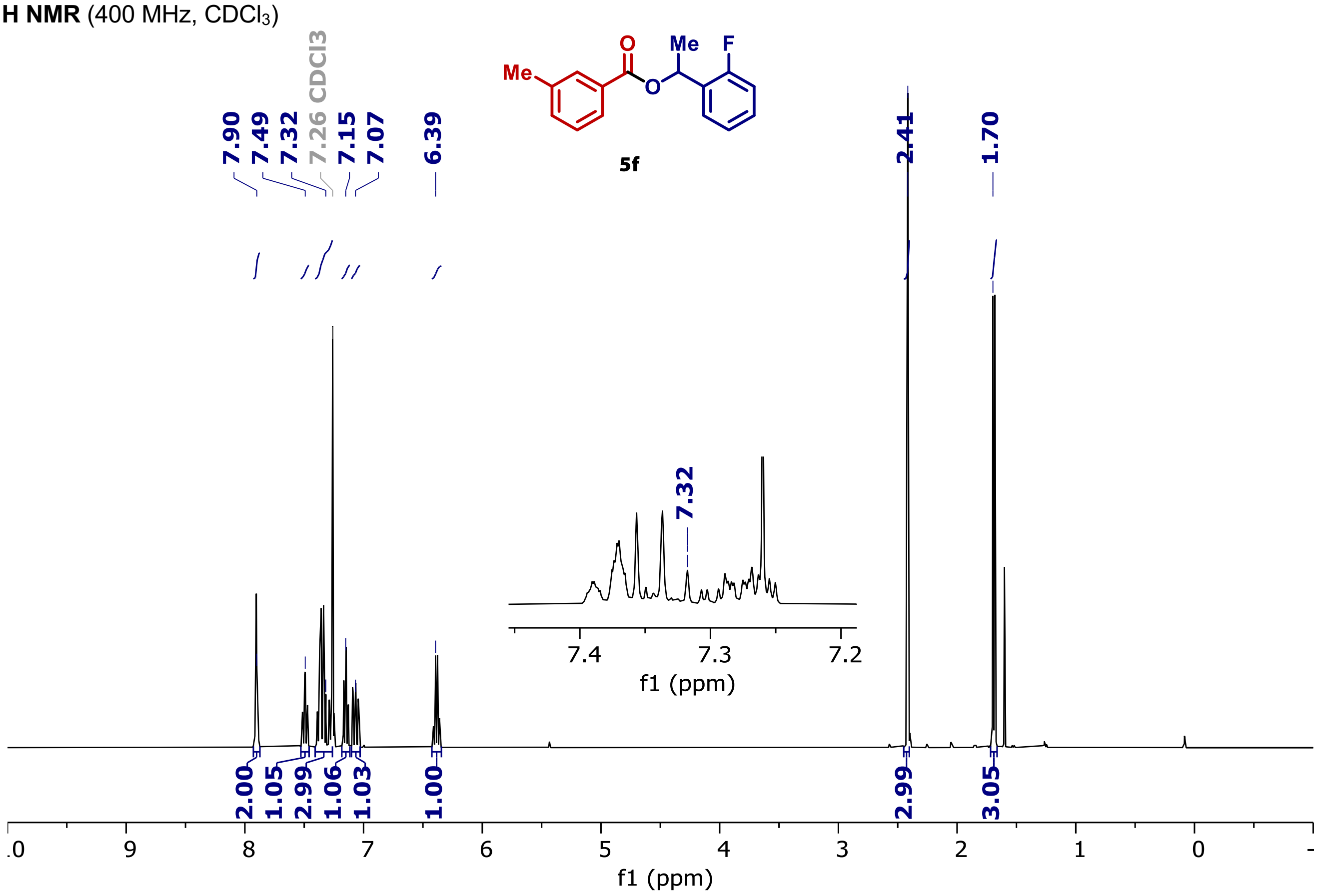

^{13}C NMR (101 MHz, $CDCl_3$)

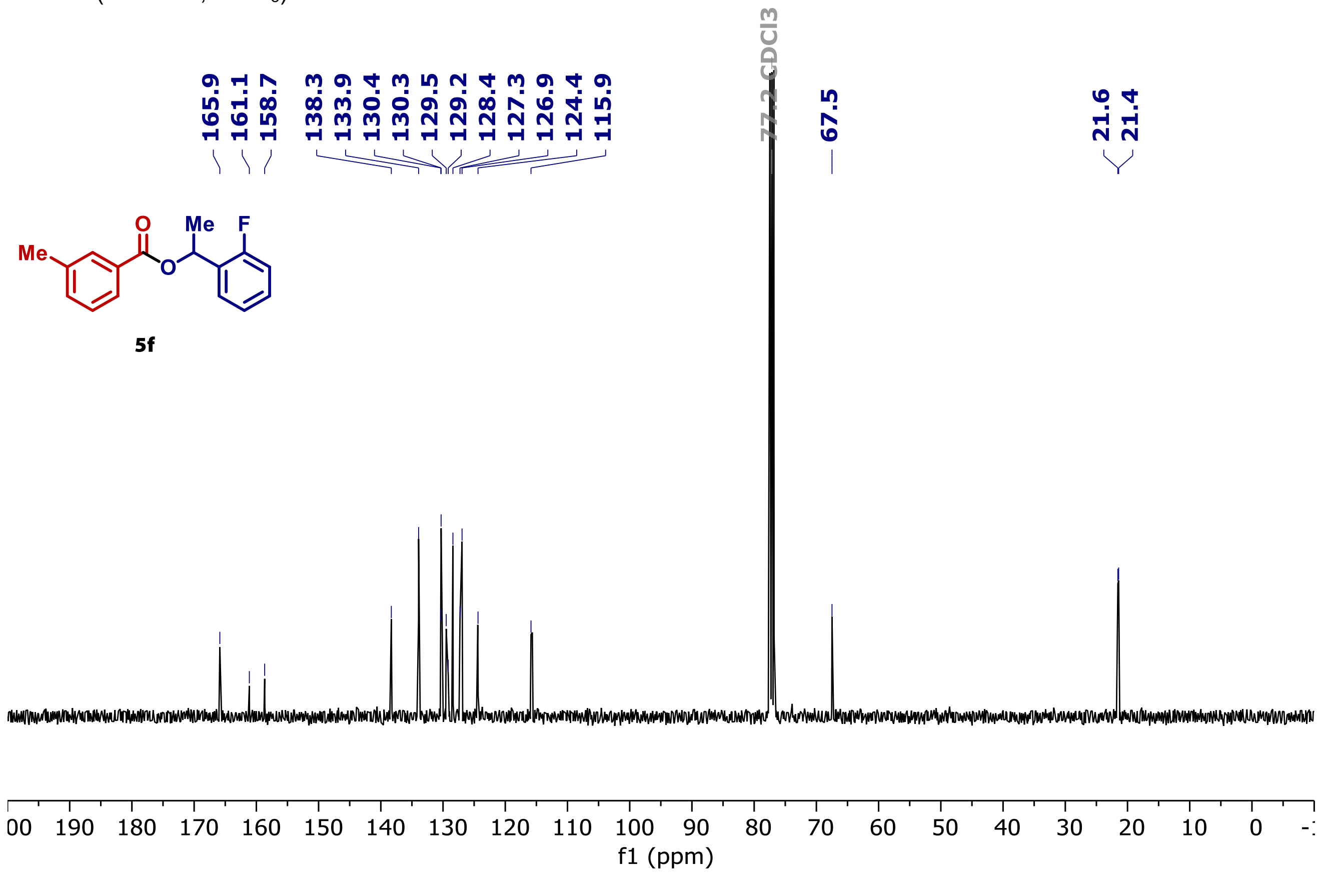

^{19}F NMR (376 MHz, $CDCl_3$)

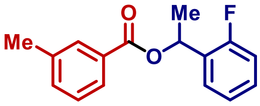

5f

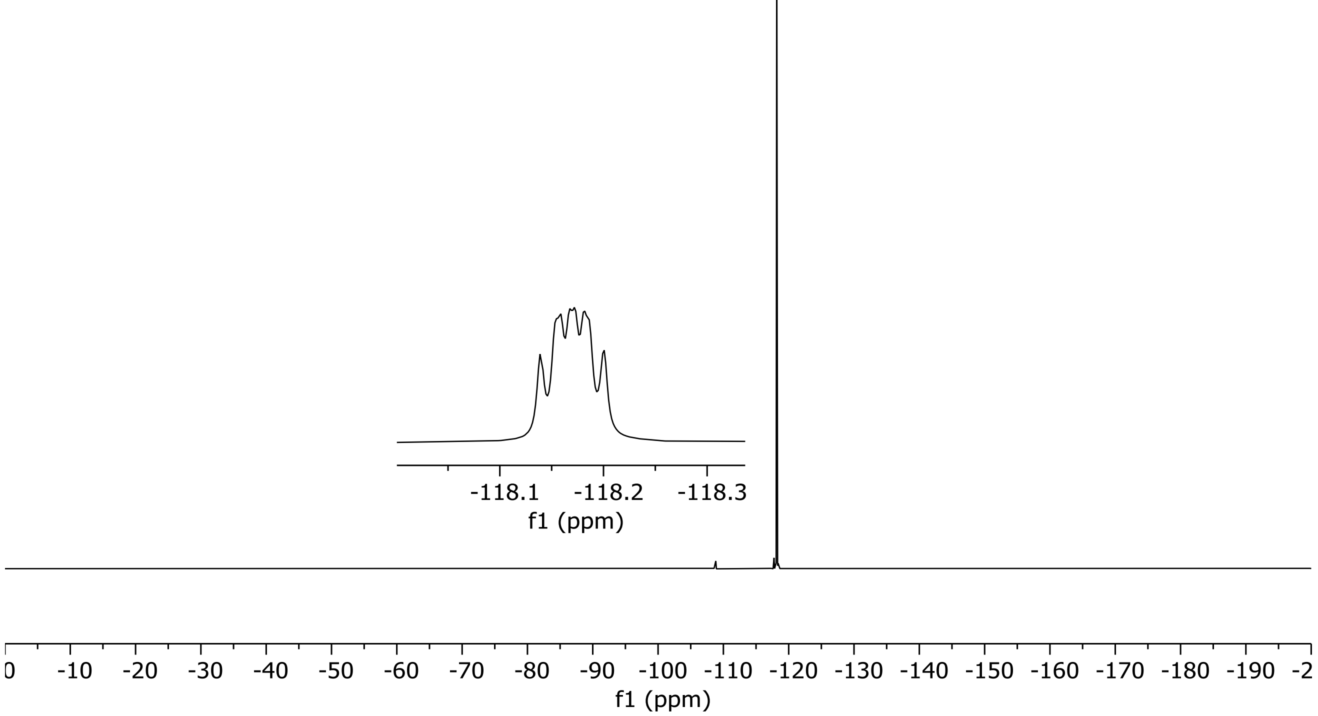

1-(4-Nitrophenyl)ethyl 4-nitrobenzoate, ***5g***

^{1}H NMR (400 MHz, $CDCl_3$)

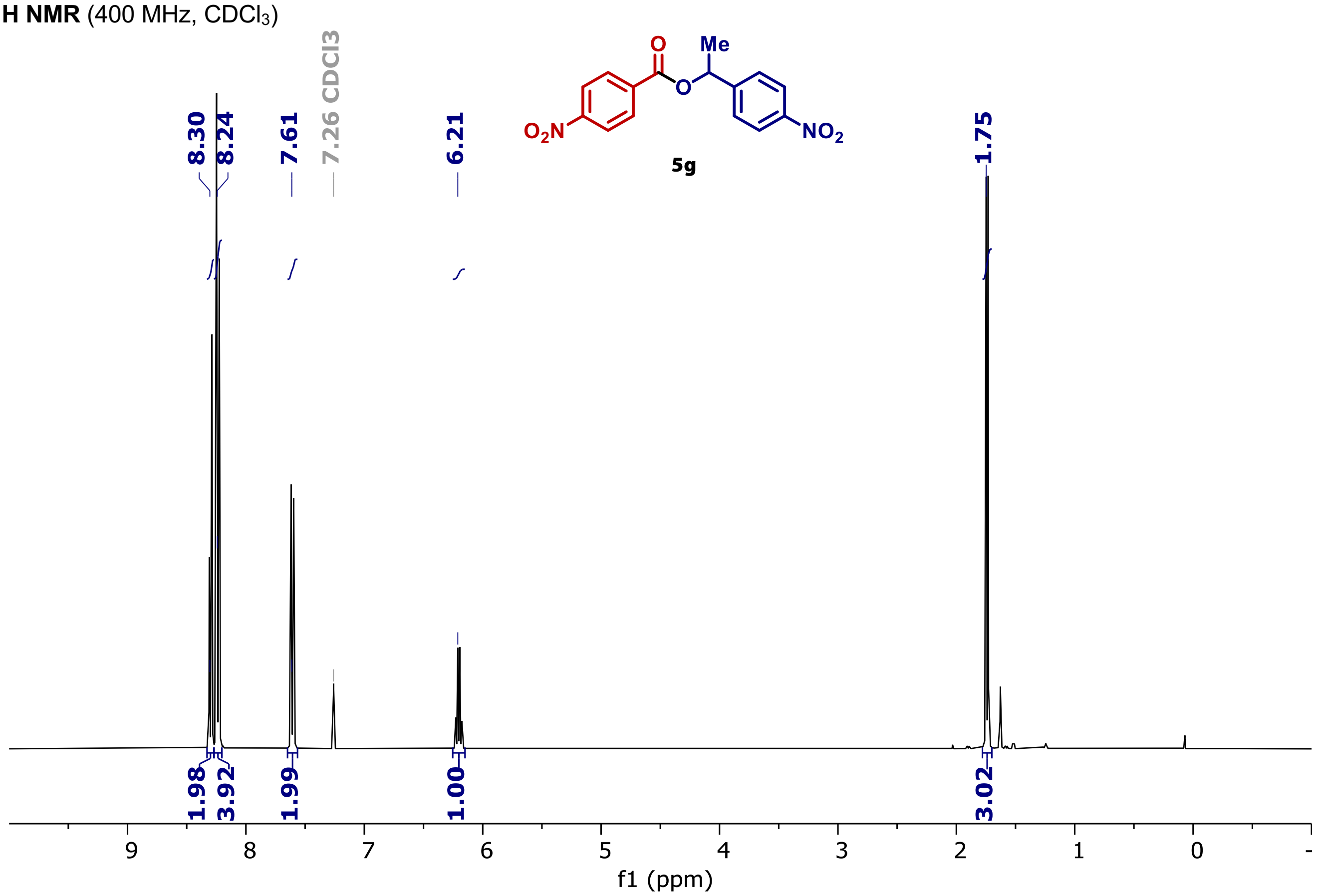

^{13}C NMR (101 MHz, $CDCl_3$)

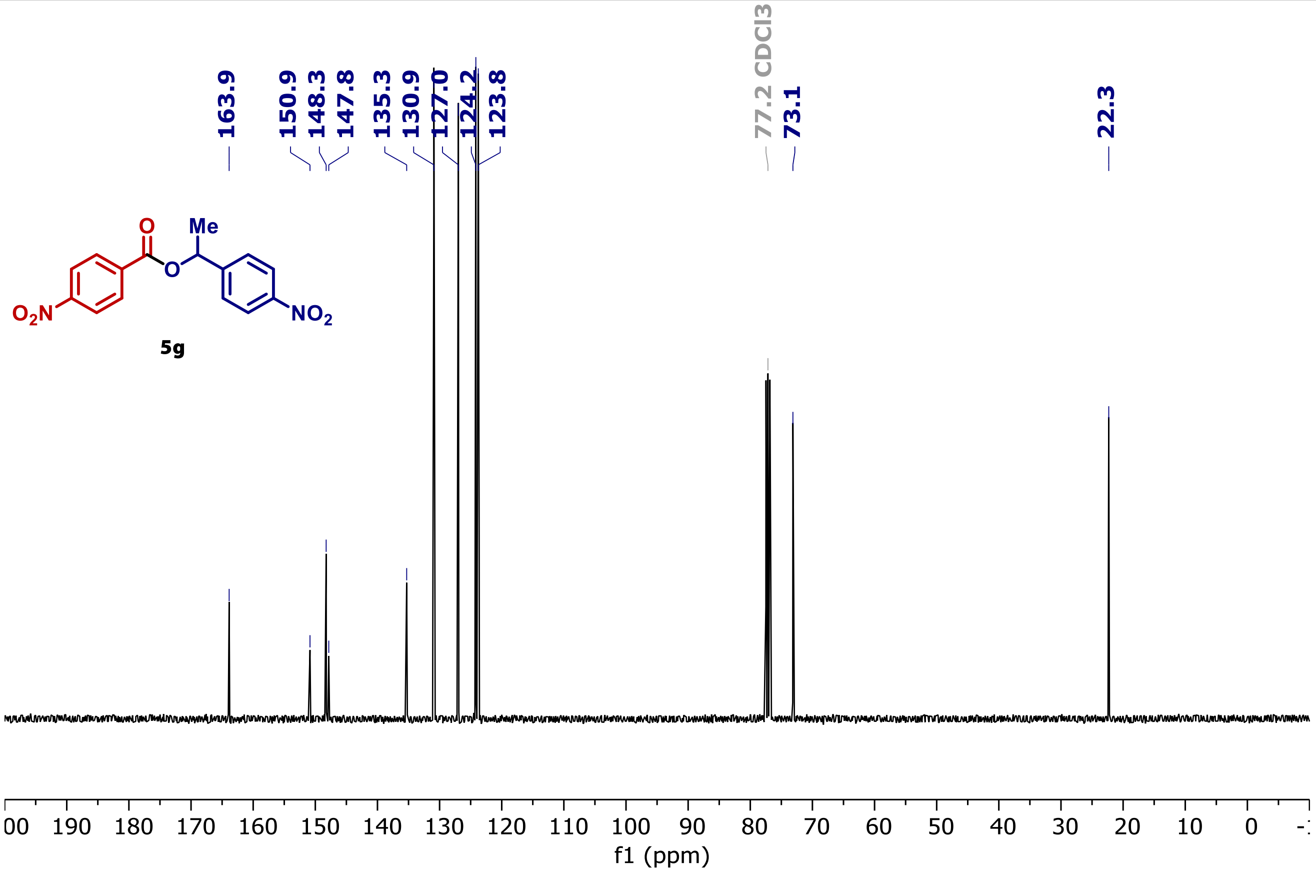
163.9
150.9
148.3
147.8
135.3
130.9
127.0
124.2
123.8
77.2 CDCl3
73.1
22.3
O
Me
O
O_2N
NO_2
5g
f1 (ppm)

SUPPORTING INFORMATION

1-(4-Methylphenyl)ethyl 4-nitrobenzoate, **5h**

^{1}H NMR (400 MHz, $CDCl_3$)

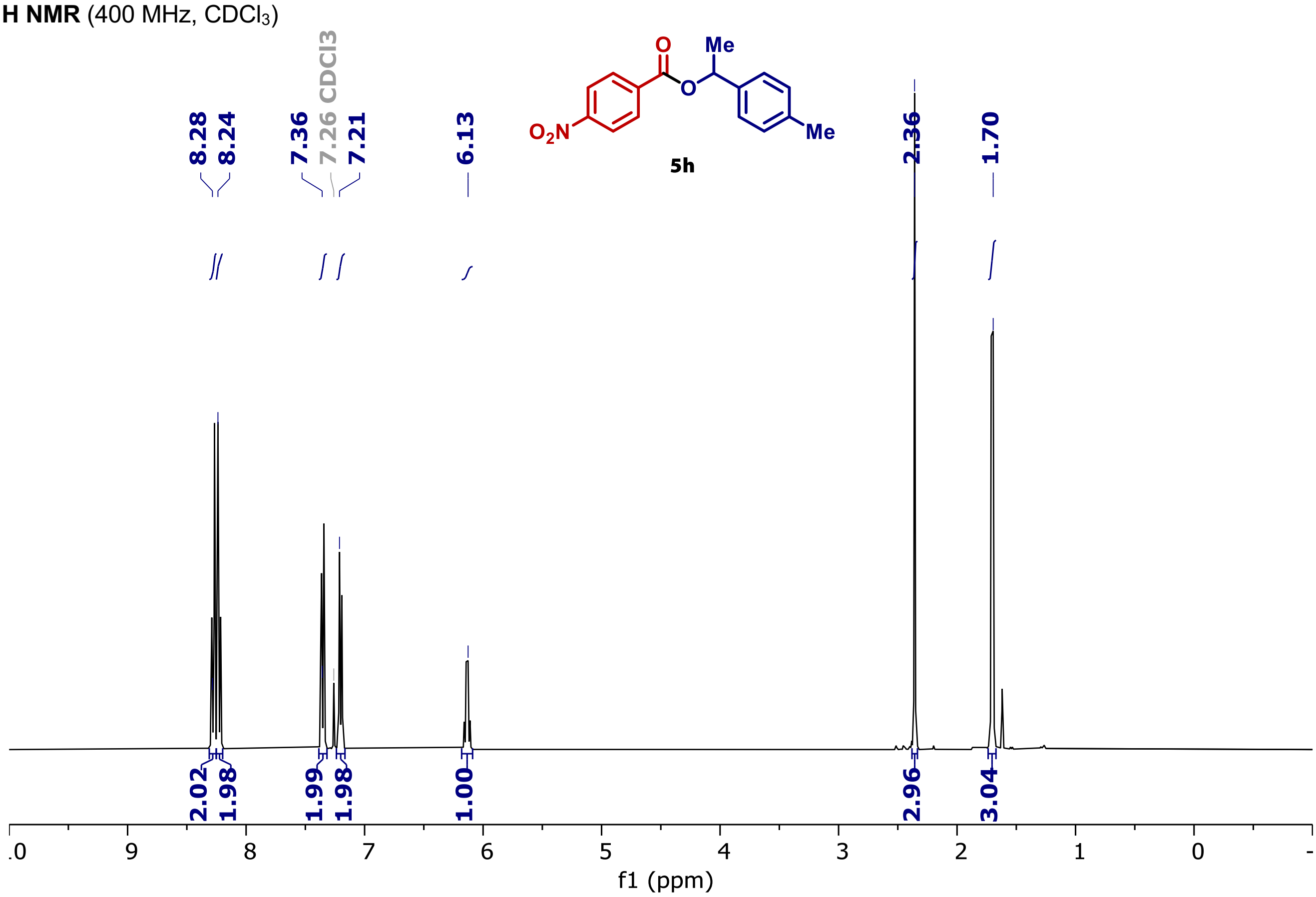

^{13}C NMR (101 MHz, $CDCl_3$)

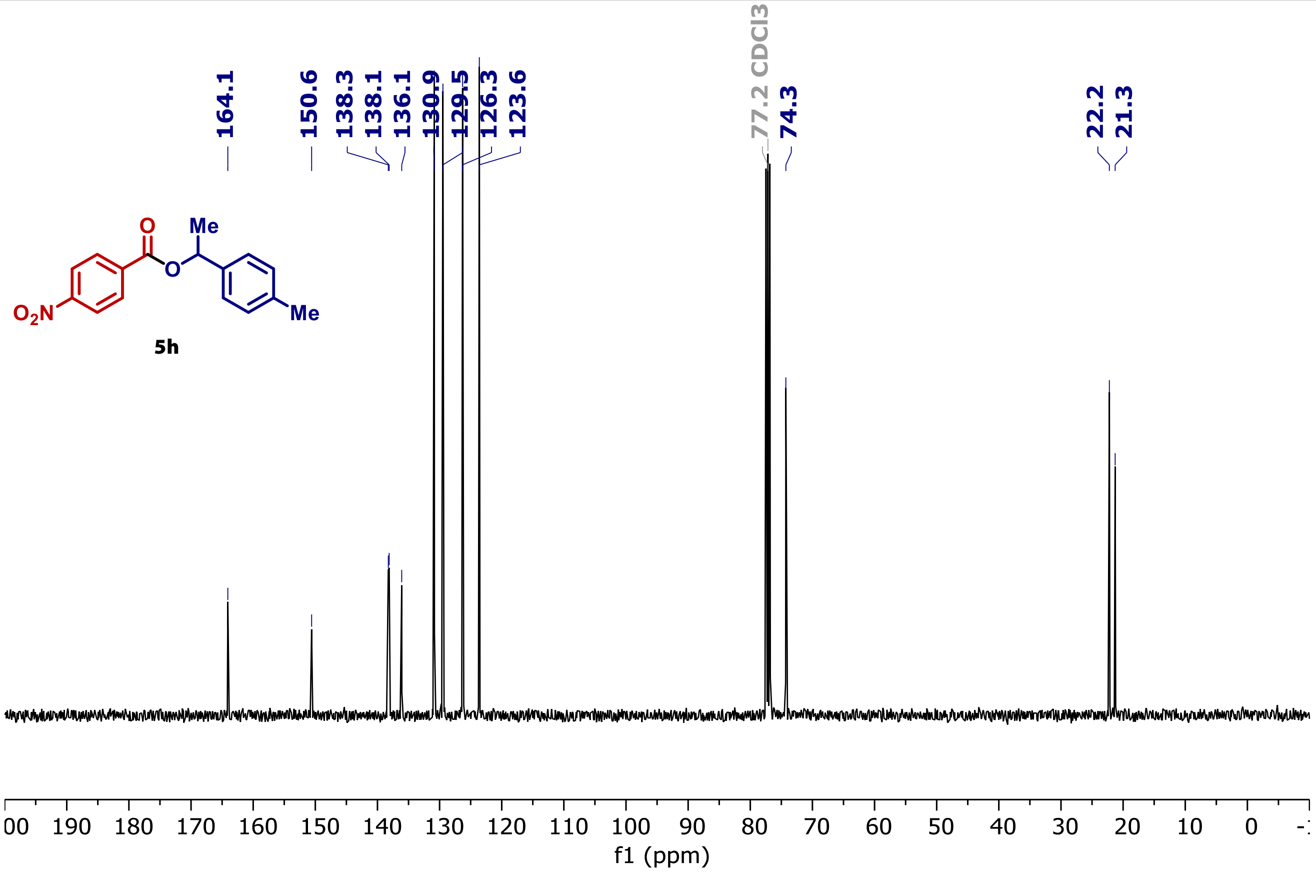
164.1
150.6
138.3
138.1
136.1
130.9
129.5
126.3
123.6
77.2 CDCl3
74.3
22.2
21.3
O
Me
O
O_2N
Me
5h
00
190
180
170
160
150
140
130
120
110
100
90
80
70
60
50
40
30
20
10
0
f1 (ppm)

*1-(2-Methylphenyl)ethyl 4-nitrobenzoate, **5i***

^{1}H NMR (400 MHz, $CDCl_3$)

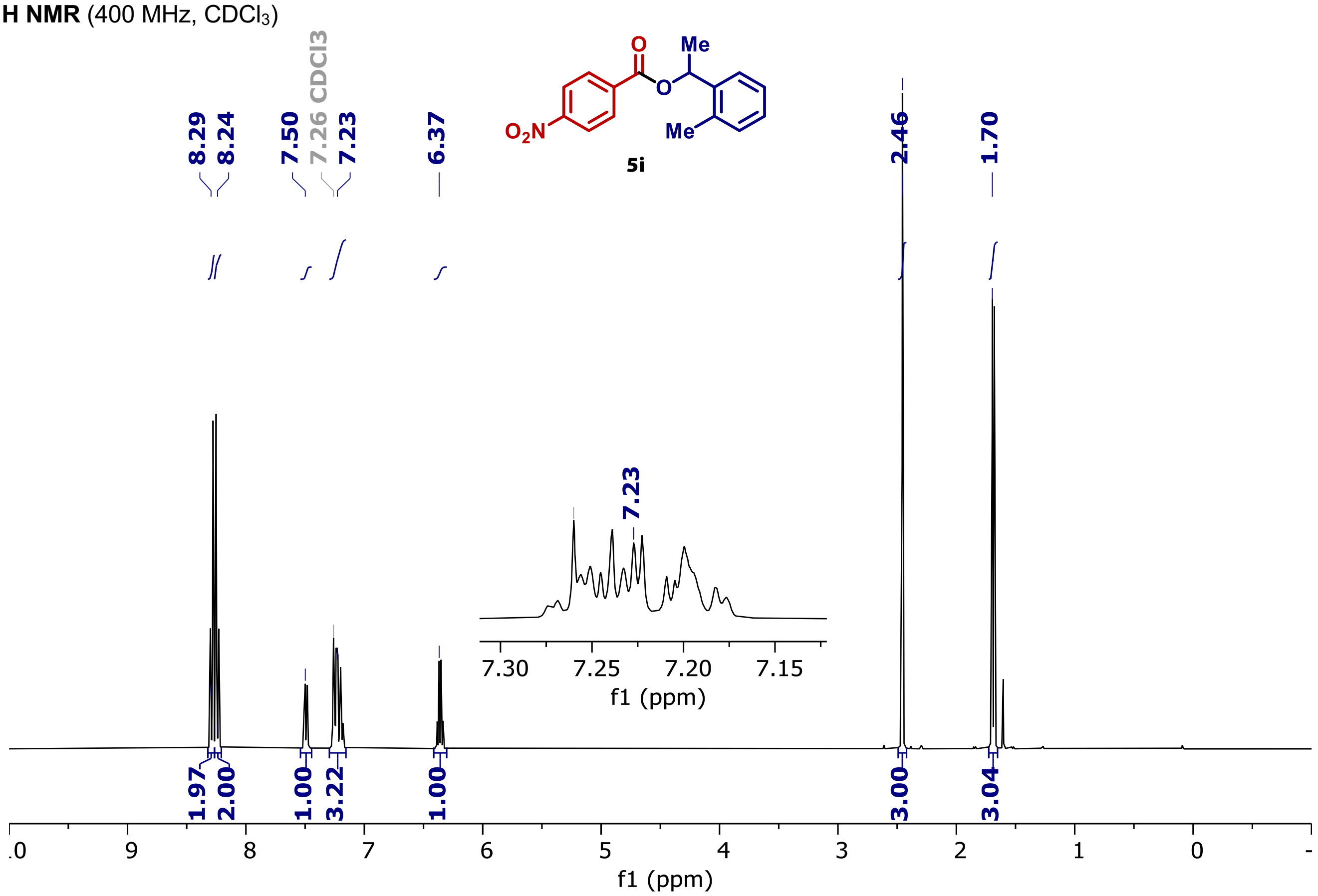

^{13}C NMR (101 MHz, $CDCl_3$)

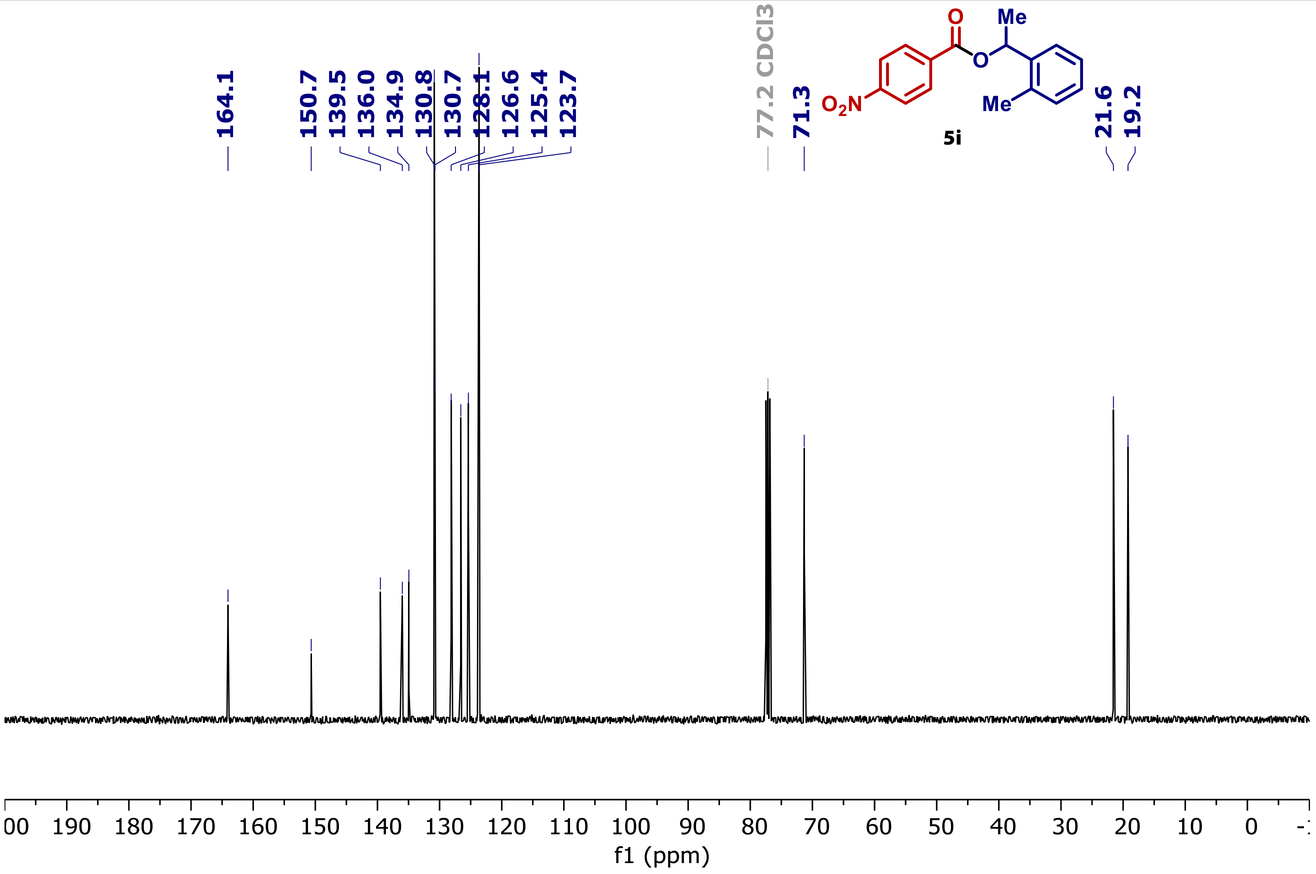
164.1
150.7
139.5
136.0
134.9
130.8
130.7
128.1
126.6
125.4
123.7
77.2 CDCl3
71.3
O_2N
O
O
Me
Me
5i
21.6
19.2
00 190 180 170 160 150 140 130 120 110 100 90 80 70 60 50 40 30 20 10 0
f1 (ppm)

*1-(2,6-Dimethylphenyl)ethyl 4-nitrobenzoate, **5j***

^{1}H NMR (400 MHz, $CDCl_3$)

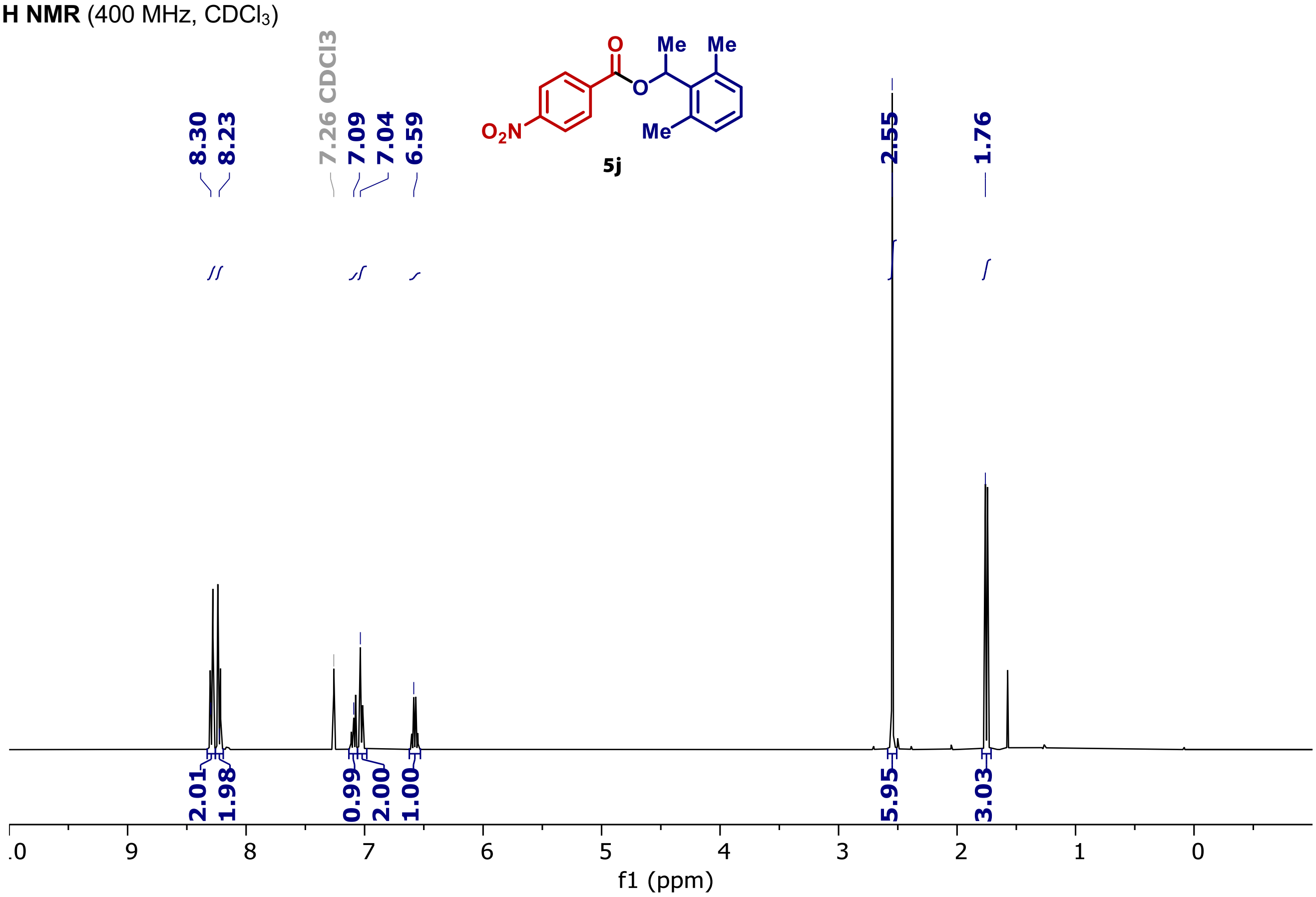

^{13}C NMR (101 MHz, $CDCl_3$)

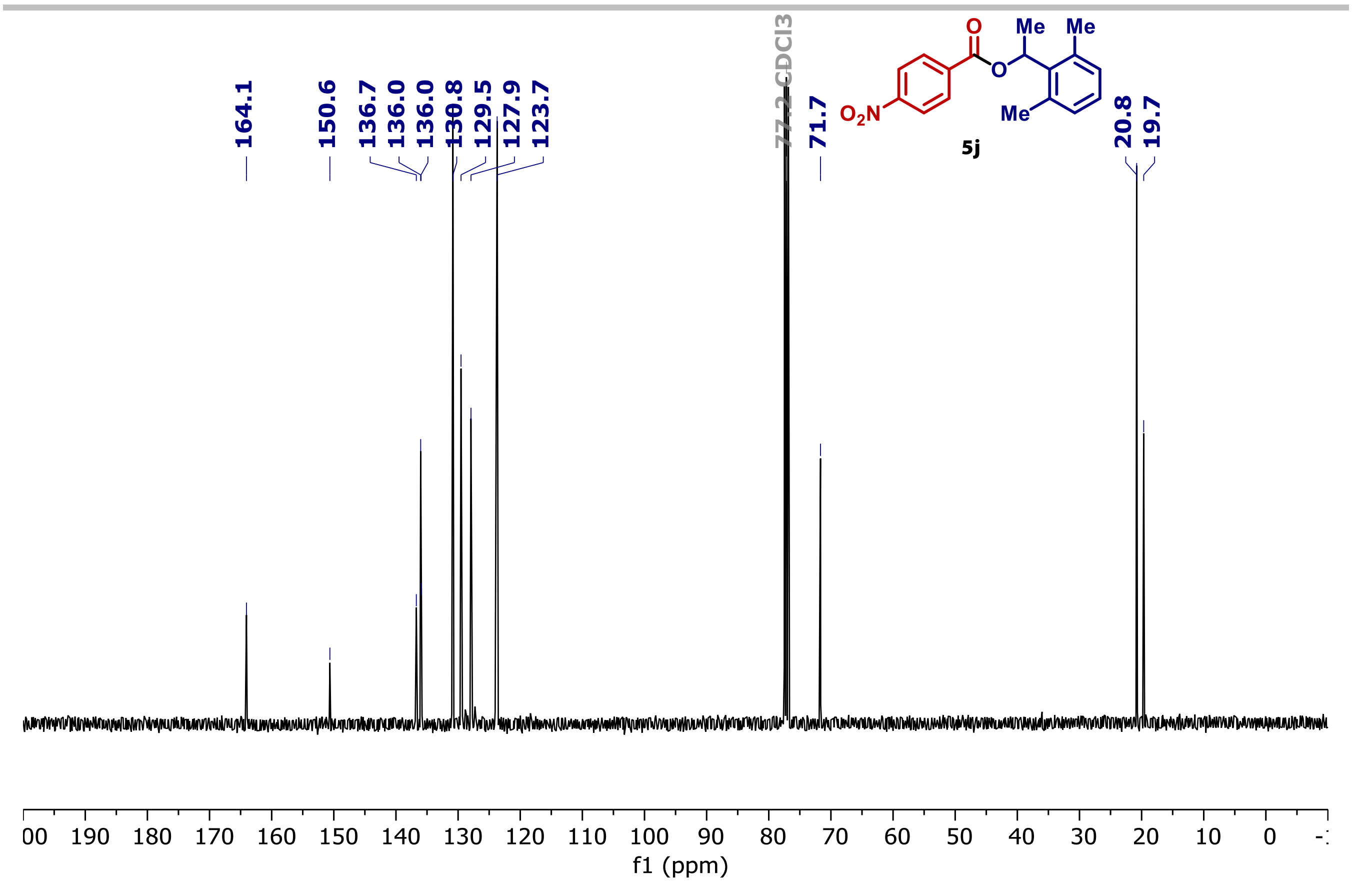
164.1
150.6
136.7
136.0
136.0
130.8
129.5
127.9
123.7
77.2 CDCl3
71.7
O
Me
Me
O
O_2N
Me
5j
20.8
19.7
00
190
180
170
160
150
140
130
120
110
100
90
80
70
60
50
40
30
20
10
0
f1 (ppm)

1-(4'-Fluoro-[1,1'-biphenyl]-4-yl)ethyl 4-nitrobenzoate, ***5l***

^{1}H NMR (400 MHz, $CDCl_3$)

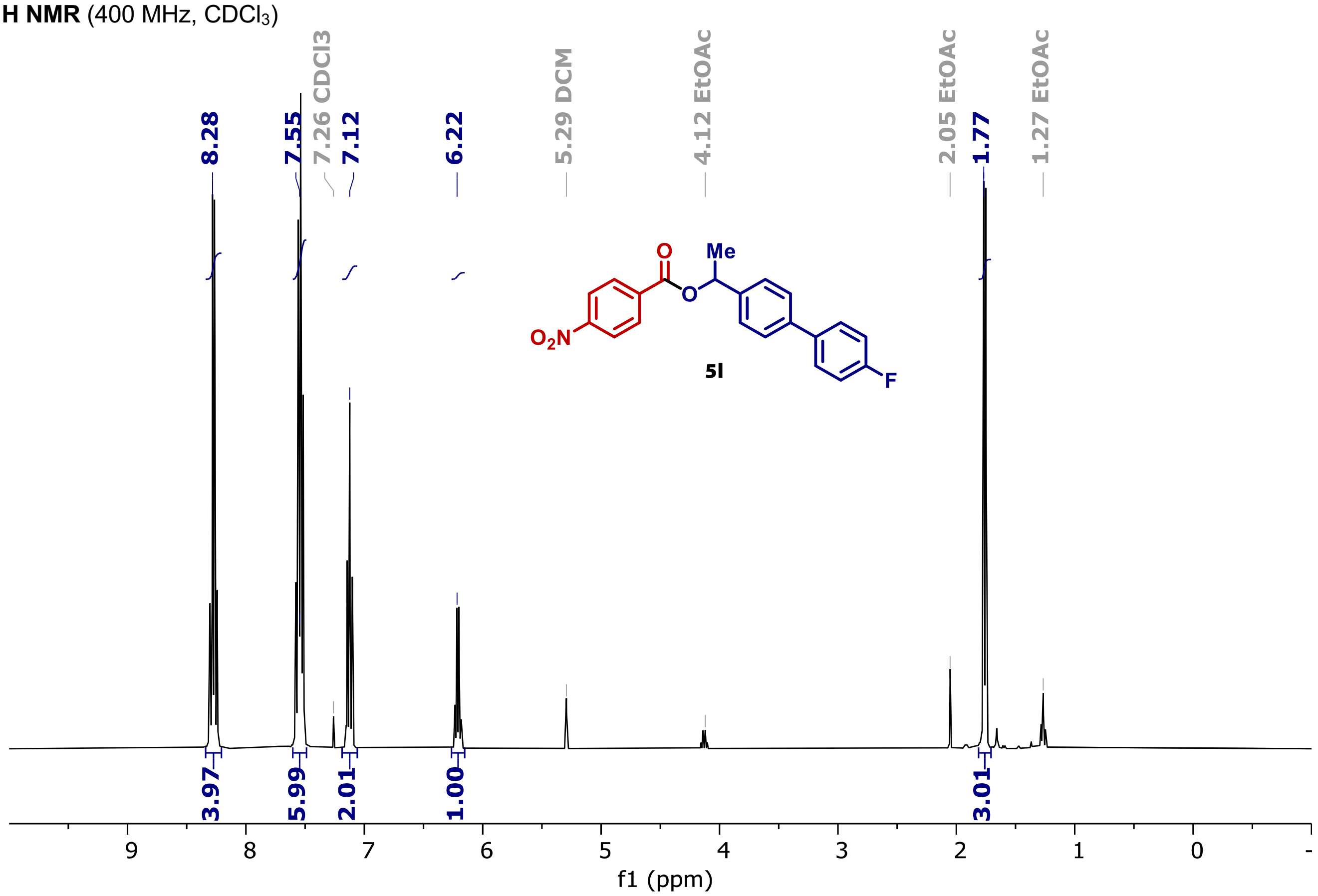

^{13}C NMR (101 MHz, $CDCl_3$)

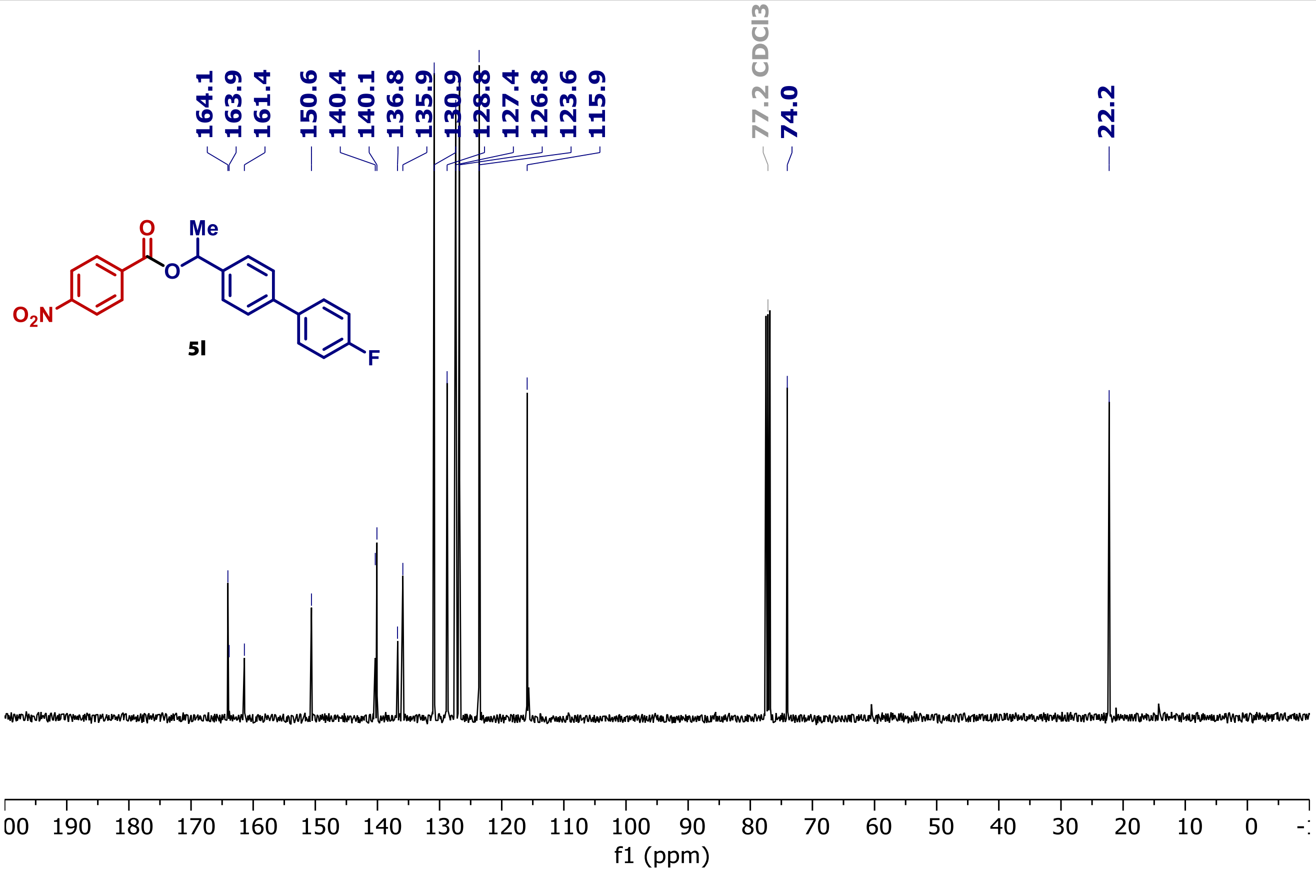

^{19}**F NMR** (376 MHz, $CDCl_3$)

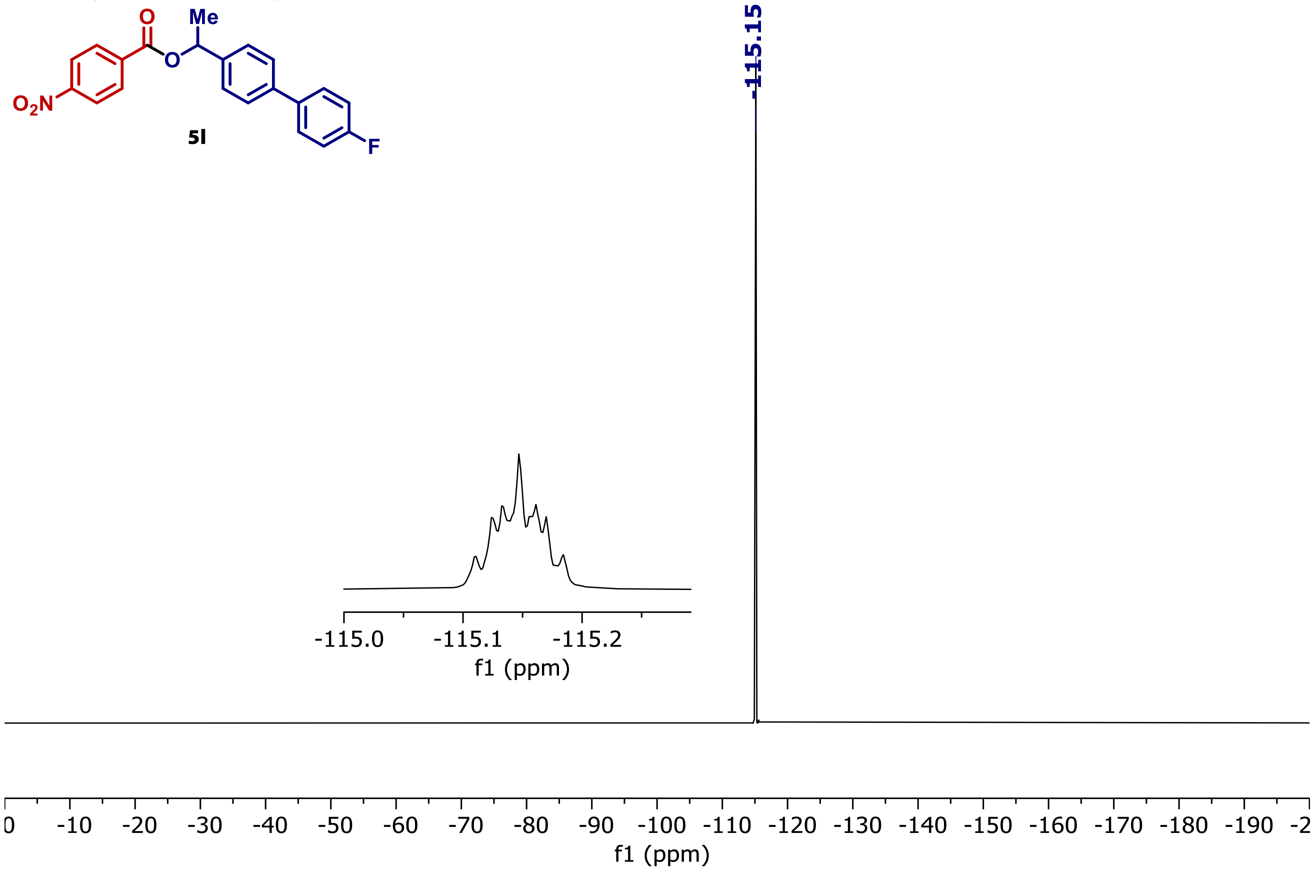

*Chroman-4-yl 4-nitrobenzoate, **5m***

^{1}H NMR (400 MHz, $CDCl_3$)

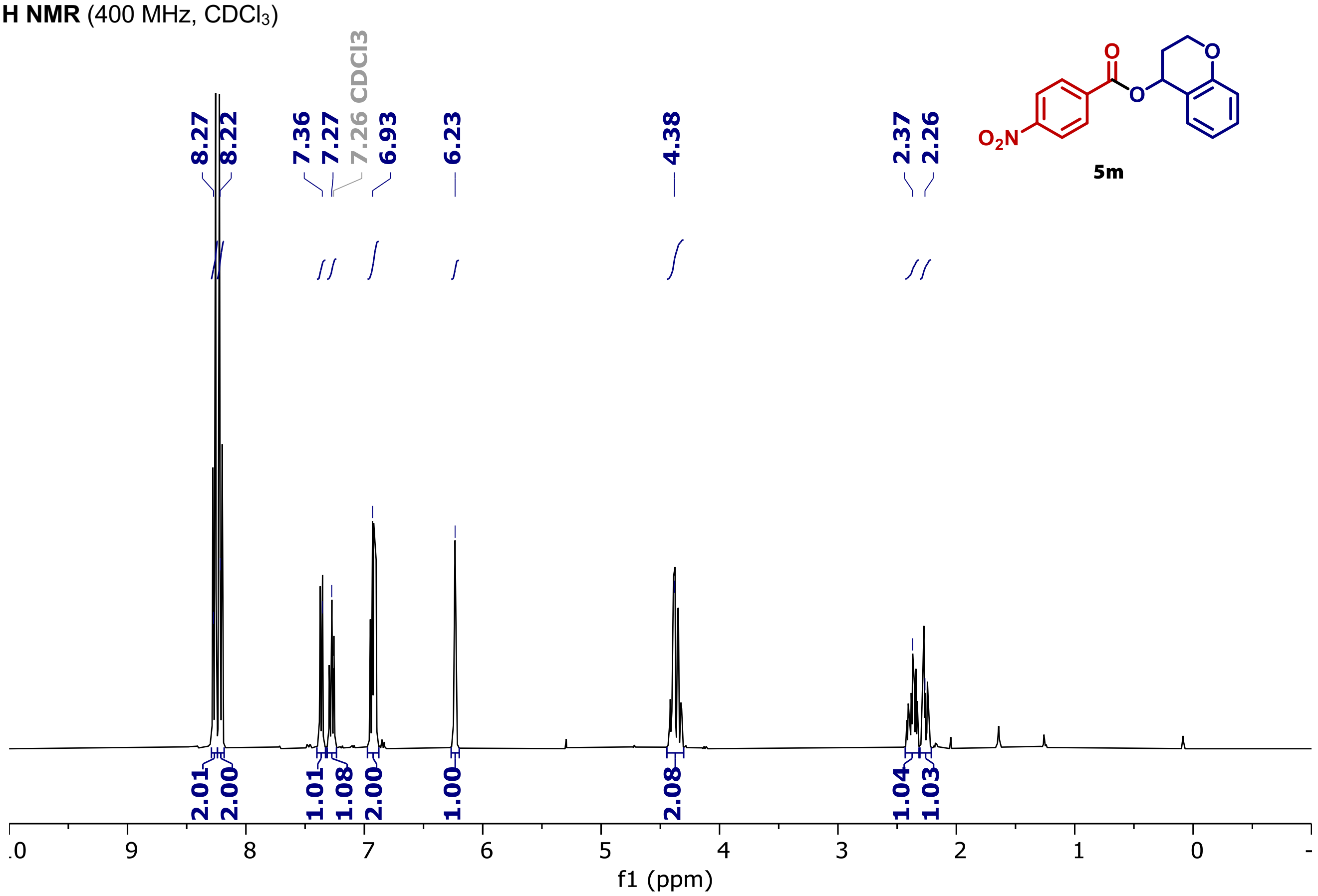

^{13}C NMR (101 MHz, $CDCl_3$)

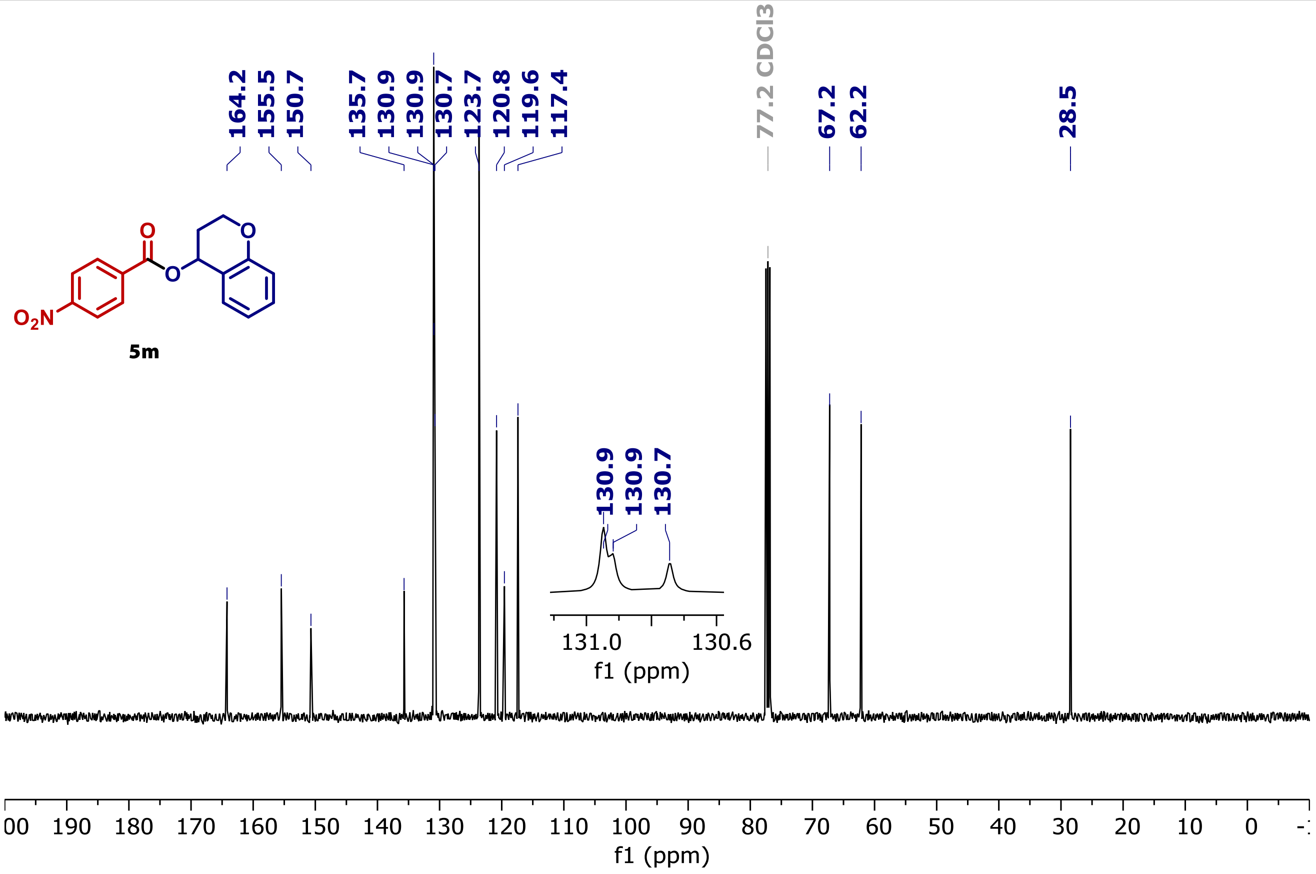
164.2
155.5
150.7
135.7
130.9
130.9
130.7
123.7
120.8
119.6
117.4
77.2 CDCl3
67.2
62.2
28.5
O
O
O
O2N
5m
130.9
130.9
130.7
131.0
130.6
f1 (ppm)
f1 (ppm)

2-Benzyl 1-(tert-butyl) (2S,4R)-4-((4-nitrobenzoyl)oxy)pyrrolidine-1,2-dicarboxylate, ***5p***

^{1}**H NMR** (400 MHz, CDCl3)

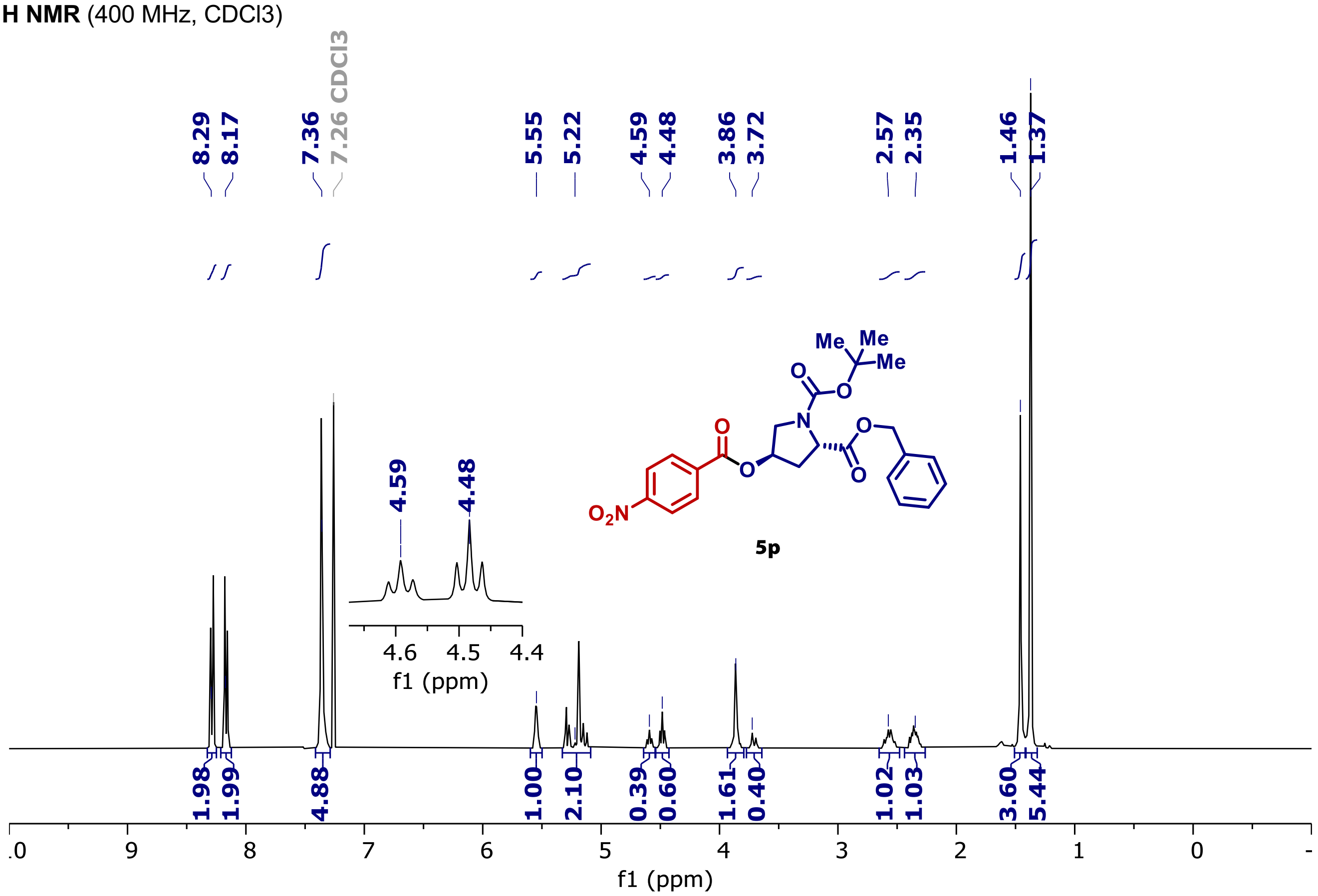

^{13}**C NMR** (101 MHz, $CDCl_3$)

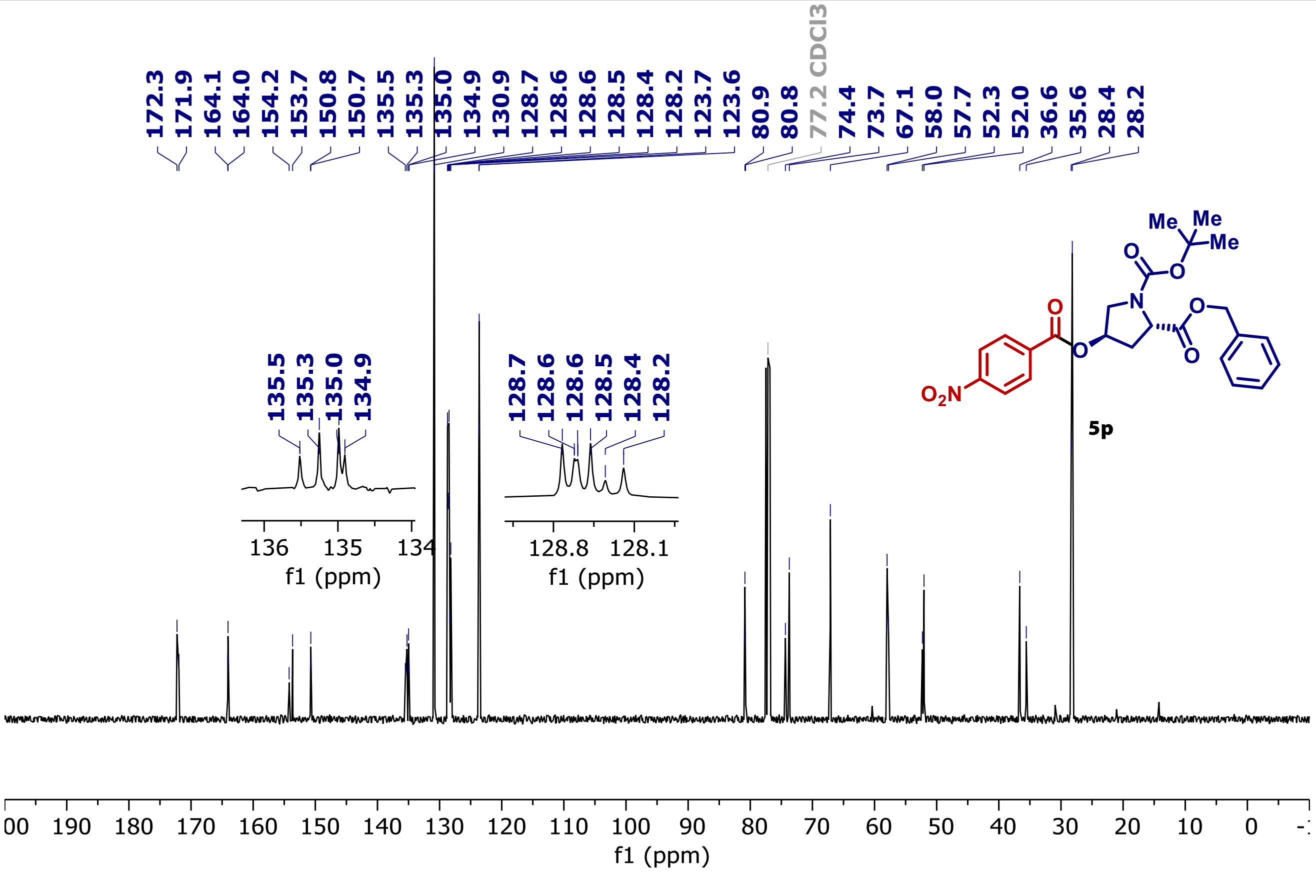
172.3
171.9
164.1
164.0
154.2
153.7
150.8
150.7
135.5
135.3
135.0
134.9
130.9
128.7
128.6
128.6
128.5
128.4
128.2
123.7
123.6
80.9
80.8
77.2 CDCl3
74.4
73.7
67.1
58.0
57.7
52.3
52.0
36.6
35.6
28.4
28.2
5p
f1 (ppm)

*O-(4-Nitrobenzoyl)-D-pantolactone, **5s***
^{1}H NMR (400 MHz, $CDCl_3$)

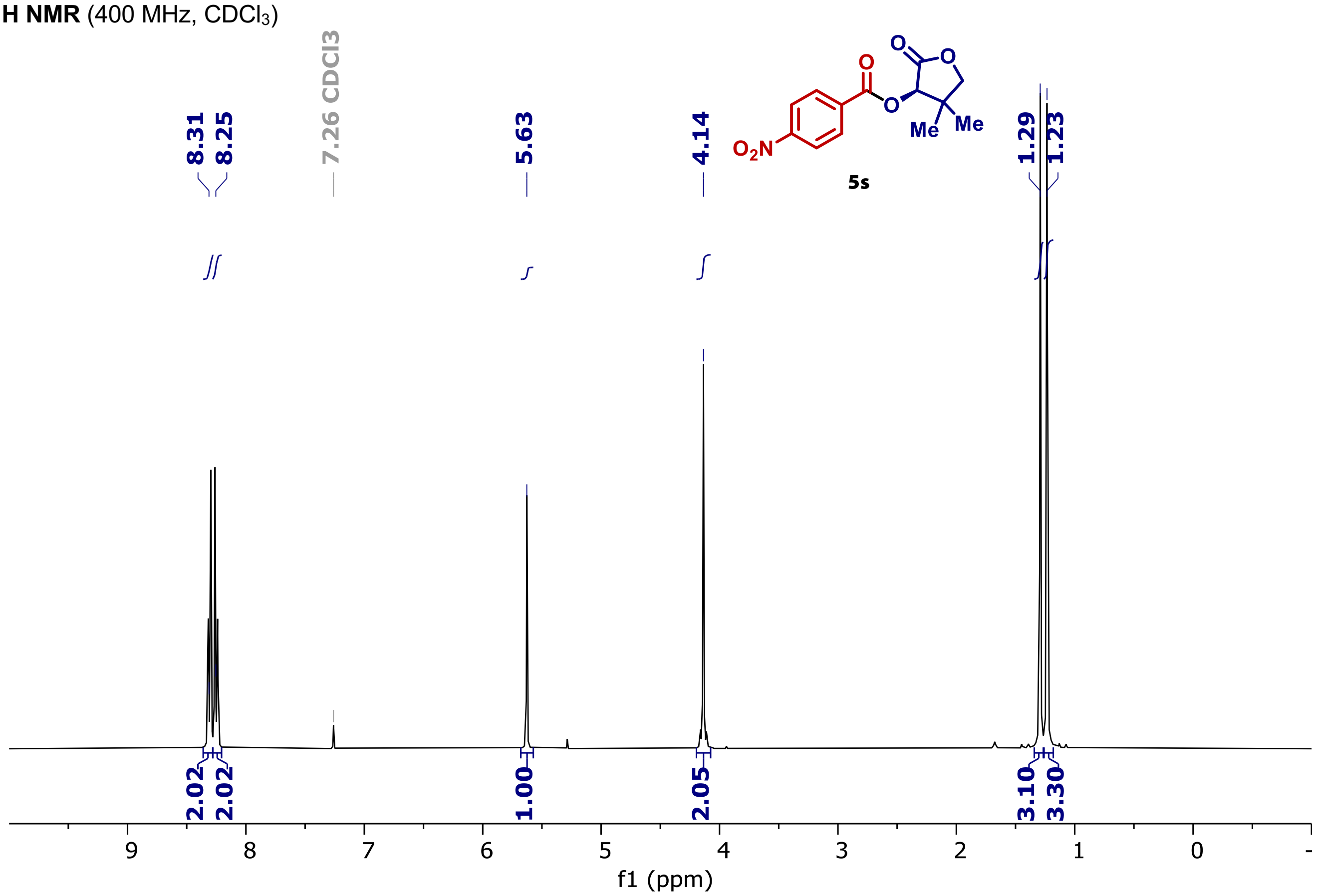

^{13}C NMR (101 MHz, $CDCl_3$)

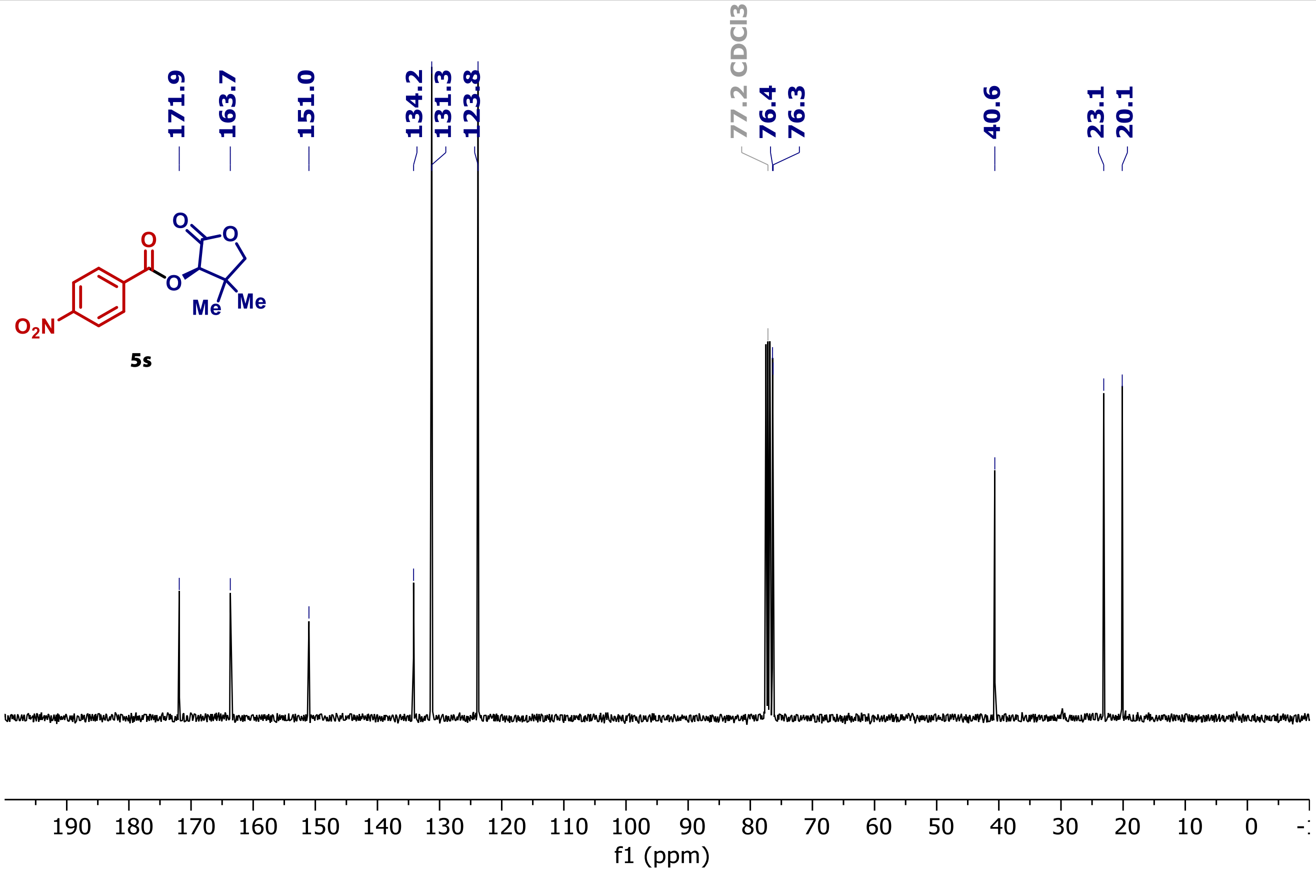
171.9
163.7
151.0
134.2
131.3
123.8
77.2 CDCl3
76.4
76.3
40.6
23.1
20.1
O2N
O
O
O
O
Me
Me
5s
190 180 170 160 150 140 130 120 110 100 90 80 70 60 50 40 30 20 10 0
f1 (ppm)

References

[1] R. T. Arnold, R. Buckles, J. Stoltenberg, *J. Am. Chem. Soc.* **1944**, *66*, 208–210.

[2] K. E. Harding, K. S. Clement, J. C. Gilbert, B. Wiechman, *J. Org. Chem.* **1984**, *49*, 2049–2050.

[3] P. Boehm, S. Roediger, A. Bismuto, B. Morandi, *Angew. Chem. Int. Ed.* **2020**, *59*, 17887–17896.

[4] B. Xing, C. Ni, J. Hu, *Angew. Chem. Int. Ed.* **2018**, *57*, 9896–9900.

[5] S. H. Kavya, V. Vijaya Kumar, C. Ramesh Kumar, *Indian J. Chem. - Sect. A Inorganic, Phys. Theor. Anal. Chem.* **2018**, *57*, 1112–1120.

[6] A. McGrath, R. Zhang, K. Shafiq, T. Cernak, *Chem. Commun.* **2023**, *59*, 1026–1029.

[7] H. Liu, C. Dong, Z. Zhang, P. Wu, X. Jiang, *Angew. Chem. Int. Ed.* **2012**, *51*, 12570–12574.

[8] X. Zhu, Z. Wang, L. Zha, Y. Zhang, Y. Qi, Q. Yuan, S. Zhou, S. Wang, *Organometallics* **2022**, *41*, 1087–1098.

[9] X.-L. Luo, D. Ge, Z.-L. Yu, X.-Q. Chu, P. Xu, *RSC Adv.* **2021**, *11*, 30937–30942.

[10] G. Blessley, P. Holden, M. Walker, J. M. Brown, V. Gouverneur, *Org. Lett.* **2012**, *14*, 2754–2757.

[11] Z. Chen, Y. Wen, Y. Fu, H. Chen, M. Ye, G. Luo, *Synlett* **2017**, *28*, 981–985.

[12] F. Xie, F. Yan, M. Chen, M. Zhang, *RSC Adv.* **2014**, *4*, 29502–29508.

[13] H. Liu, G. Shi, S. Pan, Y. Jiang, Y. Zhang, *Org. Lett.* **2013**, *15*, 4098–4101.

[14] S. A. Yakukhnov, V. P. Ananikov, *Adv. Synth. Catal.* **2019**, *361*, 4781–4789.

[15] H. Y. Bi, F. P. Liu, C. Liang, G. F. Su, D. L. Mo, *Adv. Synth. Catal.* **2018**, *360*, 1510–1516.

[16] V. K. Aggarwal, A. Mereu, *J. Org. Chem.* **2000**, *65*, 7211–7212.

[17] B. Hasimujiang, J. Zeng, Z. Yanhui, A. Abudu Rexit, *Synth. Commun.* **2018**, *48*, 887–891.

[18] A. Gulizhabaier, A. A. Rexit, *Russ. J. Org. Chem.* **2021**, *57*, 809–815.

[19] N. Aoyagi, Y. Furusho, T. Endo, *Tetrahedron* **2019**, *75*, 130781.

[20] G. Yin, M. Gao, N. She, S. Hu, A. Wu, Y. Pan, *Synthesis* **2007**, 3113–3116.

[21] J. Zhang, S. Li, G.-J. Deng, H. Gong, *ChemCatChem* **2018**, *10*, 376–380.

[22] A. Zall, D. Bensinger, B. Schmidt, *Eur. J. Org. Chem.* **2012**, *2012*, 1439–1447.

[23] X. Zhang, Y. Wu, Y. Zhang, H. Liu, Z. Xie, S. Fu, F. Liu, *Tetrahedron* **2017**, *73*, 4513–4518.

[24] Z. Zheng, B. Han, P. Cheng, J. Niu, A. Wang, *Tetrahedron* **2014**, *70*, 9814–9818.

[25] Y. Jing, C. G. Daniliuc, A. Studer, *Org. Lett.* **2014**, *16*, 4932–4935.

[26] C. Zheng, X. Zhang, M. Ijaz Hussain, M. Huang, Q. Liu, Y. Xiong, X. Zhu, *Tetrahedron Lett.* **2017**, *58*, 574–577.

[27] L. Xie, Y. Wu, W. Yi, L. Zhu, J. Xiang, W. He, *J. Org. Chem.* **2013**, *78*, 9190–9195.

[28] J. Teske, B. Plietker, *Org. Lett.* **2018**, *20*, 2257–2260.

[29] D. Wang, Z. Wan, H. Zhang, A. Lei, *Adv. Synth. Catal.* **2021**, *363*, 1022–1027.

[30] A. Jayaraman, E. Cho, J. Kim, S. Lee, *Adv. Synth. Catal.* **2018**, *360*, 3978–3989.

[31] A. Cândido, T. Rozada, A. Rozada, J. Souza, E. Pilau, F. Rosa, E. Basso, G. Gauze, *J. Braz. Chem. Soc.* **2020**, *31*, 1796–1804.

[32] Y. Zhao, D. G. Truhlar, *Theor. Chem. Acc.* **2008**, *120*, 215–241.

[33] D. Rappoport, F. Furche, *J. Chem. Phys.* **2010**, *133*, 134105.

[34] F. Weigend, F. Furche, R. Ahlrichs, *J. Chem. Phys.* **2003**, *119*, 12753–12762.

[35] K. A. Peterson, D. Figgen, E. Goll, H. Stoll, M. Dolg, *J. Chem. Phys.* **2003**, *119*, 11113–11123.

[36] B. P. Pritchard, D. Altarawy, B. Didier, T. D. Gibson, T. L. Windus, *J. Chem. Inf. Model.* **2019**, *59*, 4814–4820.

[37] M. J. Frisch, G. W. Trucks, H. B. Schlegel, G. E. Scuseria, M. A. Robb, J. R. Cheeseman, G. Scalmani, V. Barone, G. A. Petersson, H. Nakatsuji, X. Li, M. Caricato, A. V. Marenich, J. Bloino, B. G. Janesko, R. Gomperts, B. Mennucci, H. P. Hratchian, J. V. Ortiz, A. F. Izmaylov, J. L. Sonnenberg, D. Williams-Young, F. Ding, F. Lipparini, F. Egidi, J. Goings, B. Peng, A. Petrone, T. Henderson, D. Ranasinghe, V. G. Zakrzewski, J. Gao, N. Rega, G. Zheng, W. Liang, M. Hada, M. Ehara, K. Toyota, R. Fukuda, J. Hasegawa, M. Ishida, T. Nakajima, Y. Honda, O. Kitao, H. Nakai, T. Vreven, K. Throssell, J. Montgomery, J. A., J. E. Peralta, F. Ogliaro, M. J. Bearpark, J. J. Heyd, E. N. Brothers, K. N. Kudin, V. N. Staroverov, T. A. Keith, R. Kobayashi, J. Normand, K. Raghavachari, A. P. Rendell, J. C. Burant, S. S. Iyengar, J. Tomasi, M. Cossi, J. M. Millam, M. Klene, C. Adamo, R. Cammi, J. W. Ochterski, R. L. Martin, K. Morokuma, O. Farkas, J. B. Foresman, D. J. Fox, *Gaussian 16*, Gaussian Inc., Wallingford, CT, USA, **2016**.

[38] R. Dennington, T. A. Keith, J. M. Millam, *GaussView 6*, Semichem Inc., Shawnee Mission, KS, USA, **2016**.

[39] S. Miertuš, E. Scrocco, J. Tomasi, *Chem. Phys.* **1981**, *55*, 117–129.

[40] S. Hoops, S. Sahle, R. Gauges, C. Lee, J. Pahle, N. Simus, M. Singhal, L. Xu, P. Mendes, U. Kummer, *Bioinformatics* **2006**, *22*, 3067–3074.

[41] K. D. Collins, A. Rühling, F. Glorius, *Nat. Protoc.* **2014**, *9*, 1348–1353.

[42] K. D. Collins, F. Glorius, *Nat. Chem.* **2013**, *5*, 597–601.

[43] A. C. Spivey, T. Fekner, S. E. Spey, *J. Org. Chem.* **2000**, *65*, 3154–3159.

[44] L. Adak, N. Yoshikai, *J. Org. Chem.* **2011**, *76*, 7563–7568.

[45] F. Li, X.-X. Guo, G.-Z. Zeng, W.-W. Qin, B. Zhang, N.-H. Tan, *Bioorg. Med. Chem. Lett.* **2018**, *28*, 2523–2527.

[46] T. Kinoshita, K. Shibayama, K. Ikai, K. Okamoto, *Bull. Chem. Soc. Jpn.* **1988**, *61*, 2917–2922.

[47] M. T. La, H.-K. Kim, *Tetrahedron Lett.* **2018**, *59*, 1855–1859.

[48] E. Kleinpeter, P. Werner, T. Linker, *Tetrahedron* **2017**, *73*, 3801–3809.

[49] T. Zhong, M. K. Pang, Z. Da Chen, B. Zhang, J. Weng, G. Lu, *Org. Lett.* **2020**, *22*, 3072–3078.

[50] J. A. Hodges, R. T. Raines, *J. Am. Chem. Soc.* **2003**, *125*, 9262–9263.

[51] A. S. Sokolova, K. S. Kovaleva, S. O. Kuranov, N. I. Bormotov, S. S. Borisevich, A. A. Zhukovets, O. I. Yarovaya, O. A. Serova, M. B. Nawrozkij, A. A. Vernigora, A. V. Davidenko, E. M. Khamitov, R. Y. Peshkov, L. N. Shishkina, R. A. Maksuytov, N. F. Salakhutdinov, *ChemMedChem* **2022**, *17*, e202100771.

[52] S. Maity, A. Roy, S. Duari, S. Biswas, A. M. Elsharif, S. Biswas, *Eur. J. Org. Chem.* **2021**, *2021*, 3569–3572.
